# Computationally Efficient Yet Quantitatively Accurate Scaled MP2 Protocols for the Prediction of Weak Interaction Energies in Complex Biological Systems

**DOI:** 10.1021/acsomega.5c07079

**Published:** 2025-08-20

**Authors:** Luis Soriano-Agueda, Anaid Flores, Paulino Zeron, Marco Franco-Pérez

**Affiliations:** † Departamento de Física y Química Teórica, 164178 Facultad de Química, Universidad Nacional Autónoma de México, Cd. Universitaria, 04510 Ciudad de Mexico, Mexico; ‡ Departamento de Química, 27788 Universidad Autónoma Metropolitana-Iztapalapa, Av. San Rafael Atlixco 186, 09340 Ciudad de México, Mexico

## Abstract

In this study, we introduce a set of novel computational strategies based on second-order Mo̷ller–Plesset perturbation theory (MP2), enhanced through acceleration techniques, such as the resolution of the identity (RI). These approaches are further refined via spin-component scaling (SCS), following Grimme’s methodology, and are specifically calibrated for the quantitatively accurate prediction of weak interaction energies—interactions that play a critical role in biological systems. Among the developed methods, three variants exhibit outstanding performance, surpassing the accuracy of several state-of-the-art, nondynamical electronic structure techniques. Benchmarking against a comprehensive data set of 274 dimerization energies, computed at the CCSD­(T)/CBS level of theory, reveals that these models deliver quantitatively accurate interaction energies. In particular, the RIJCOSX-SCS-MP2^BWI‑DZ^ method, employing uniquely optimized scaling parameters, demonstrates exceptional accuracy (errors below 1 kcal/mol) while maintaining computational efficiency superior to widely used hybrid and meta-GGA density functional approximations (DFAs). This method reliably captures a range of biologically relevant interactions, including π–π stacking between nucleotide base pairs, halogen bonding, and dissociation energy profiles, showcasing its robustness and predictive power. Given its accuracy, efficiency, and versatility, RI-SCS-MP2^BWI‑DZ^, RIJK-SCS-MP2^BWI‑DZ^, and RIJCOSX-SCS-MP2^BWI‑DZ^ emerge as promising and reliable alternatives for modeling weak interactions in complex biological environments.

## Graphical abstract

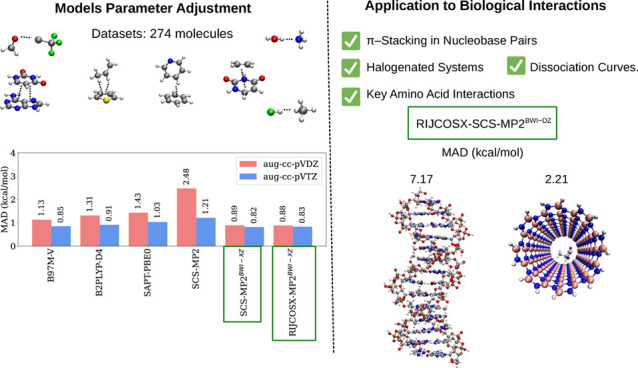

## 1 Introduction

Weak interactions are fundamental to virtually all biochemical processes, and they can be considered, as the invisible architects of life.^1–5^ With bond energies of about 0.1–5 kcal/mol, these reversible and dynamic forces govern protein folding, stabilize DNA and RNA structures as well as lipid bilayers, and enable molecular recognition in enzyme–substrate, antibody–antigen, and signaling complexes.^6–13^ Reversibility of weak interactions allows biomolecules and macromolecular complexes to dynamically transition between different conformational states, each with specific metabolic or functional significance.^14^ In general, a comprehensive understanding of biological processes requires an in-depth analysis of the weak interactions involved, as they directly influence the structural and functional dynamics of biomolecules.

The most important types of weak interactions in biological systems include hydrogen bonding, electrostatic interactions (such as ion–dipole and dipole–dipole forces), van der Waals forces, hydrophobic effects, halogen bonding, and induction interactions.^6,11,15–17^ Stacking interactions, which are primarily electrostatic in nature, were long believed to be significantly weaker than hydrogen bonds.^18,19^ However, recent findings underscore their importance in molecular recognition and structural stability.^20–22^ Given their ubiquitous role in biochemistry, weak interactions represent one of the most pressing challenges in contemporary molecular science. Accurately modeling weak interactions remains a formidable task due to their subtle energetic contributions, which fall within the accuracy limits of even the most sophisticated quantum chemical protocols. The complexity of biochemical systems further exacerbates this challenge as the sheer number of correlated electrons makes high-level electronic structure calculations computationally prohibitive. Weak interactions operate through cooperative effects, where small errors in energy calculations may propagate into unphysical results.^23,24^

Currently, there is an increasing interest in the quantum chemistry community for the development of new electronic structure methods that significantly improve the accuracy of interaction energy estimations. In the framework of density functional theory (DFT),^25,26^ double-hybrid density functional approximations (DFAs), such as B2PLYP and DSD-BLYP with empirical dispersion corrections (D3BJ), have demonstrated superior performance in describing weak interactions.^27–30^ Furthermore, rank-separated hybrid DFAs, particularly those developed by Head-Gordon et al.—including ωB97M-V, ωB97X-V, and ωB97X-D3—have shown excellent predictive capabilities.^31–34^ Beyond DFT, symmetry-adapted perturbation theory^35–38^ (SAPT) provides a powerful approach for accurately decomposing intermolecular interaction energies into fundamental components,^39–41^ such as electrostatics, induction, dispersion, and exchange. The development and refinement of SAPT-DFT methodologies offer promising avenues for efficient and reliable modeling of weak interactions in complex biochemical environments.^36,37,42,43^ Despite these tools, the field faces a paradox: weak interactions are individually feeble but collectively decisive; their accurate description demands computationally efficient methods with rigorous treatment of electron correlation, keeping at bay the propagation of a possible source of errors.

Among quantum-based methodologies, traditional second-order Mo̷ller–Plesset perturbation theory^44^ (MP2) stands out for its ability to naturally account for weak interactions, providing a reasonable description of electron correlation. However, MP2 describes chemical properties such as atomization energies, biradicals, transition states, and interactions involving π-systems inaccurately.^45–48^ Beyond standard MP2, it has been extensively demonstrated that scaling the spin energy components—same-spin energy contribution (ESS) and opposite-spin energy contribution (EOS)—by empirical coefficients (*C*_OS_ and *C*_SS_, respectively) leads to quantitatively accurate predictions of various types of weak interaction energies.^49–51^ This refinement, known as Spin-Component Scaled MP2 ^52,53^ (SCS-MP2), was originally proposed by Grimme and later extended by other researchers for modeling specific weak interactions, including the SCS-MP2-vdW,^54^ SCS­(MI)-MP2,^55^ and SCSN-MP2 ^56^ strategies, conjointly with the SCS-MP2-hal parametrization recently developed by us for halogen containing compounds (this work is under review).^57^

Other quantum chemistry approaches based on MP2 theory have been developed under local electron correlation arguments to reduce computational cost, enabling the study of larger molecules. These methods approximate the correlation energy by considering only interactions between localized molecular orbitals (LMOs) within a certain spatial range.^58,59^ On the other hand, the MP2C (Hesselmann) method is a computational chemistry technique that improves the accuracy of second-order Mo̷ller–Plesset perturbation theory (MP2) calculations. It achieves this by correcting the MP2 energy, which often underestimates these interactions, with a “coupled” dispersion energy calculated in a monomer-centered basis set.^60,61^ Finally, regularized MP2, as proposed by Shee et al., is a method for improving the accuracy of second-order Mo̷ller–Plesset perturbation theory (MP2) calculations, by addressing issues like overestimated dispersion interactions and dative bonds.^62^ It involves introducing a regularization term to the MP2 energy expression, effectively damping the effects of large correlation energies that arise from near-degeneracy or strong interactions.

Despite its improved accuracy, the high computational cost of MP2 remains a limiting factor for its application to large molecular systems, particularly those of biological relevance. Recent advances in algorithmic acceleration—most notably those leveraging the resolution of the identity (RI)^63,64^ approximation—have considerably increased the practicality of MP2-based methods.^65^ Two prominent implementations are the RI-JK method, which applies the RI approximation to both Coulomb (J) and exchange (K) integrals, and the RIJ-COSX method,^66^ which combines RI treatment of Coulomb terms with numerical integration of exchange contributions over predefined grids. These approaches, often termed RIJK-MP2 and RIJCOSX-MP2, respectively, offer substantial reductions in computational cost without compromising accuracy.

While these accelerated methods are compatible with the energy decomposition framework of standard MP2—and thus with spin-component scaling strategies—they have not been widely explored in this context. Particularly, RI, RI-JK, and RIJ-COSX methods show excellent performance in calculating energies, with considerable time savings.^67–69^ In contrast to the popularity of DFAs for modeling large systems, RI-accelerated MP2 techniques remain underutilized for studying noncovalent interactions involving molecular systems from medium to large sizes, despite their theoretical advantages. Moreover, as far as we are aware, no prior efforts have been made to recalibrate spin-component scaling parameters specifically for RI-MP2 variants. This represents a missed opportunity to enhance both the accuracy and efficiency of MP2 for applications requiring the precise treatment of weak interactions.

The present work addresses this gap by developing and calibrating new spin-component-scaled RI-MP2 methodologies, aiming to deliver computationally efficient yet quantitatively accurate protocols for the prediction of weak interaction energies in biologically related weak interactions. To validate these new approaches, we conduct rigorous benchmarking against state-of-the-art reference data, including coupled cluster CCSD­(T)/CBS values. By doing so, we aim to establish RI-accelerated SCS-MP2 protocols as viable and competitive, and even more accurate alternatives to current DFA-based methods in the modeling of noncovalent interactions. To achieve this goal, we follow a structured approach: (i) data set selection—we curated a diverse set of 274 molecular systems (see the Data Set Description section), encompassing hydrogen bonds, π-stacking interactions, London dispersion forces, halogen-bonding, and complexes combining these effects. The interaction energies for these systems, previously reported at the CCSD­(T)/CBS level (using full basis set extrapolation), serve as benchmark values for evaluating our methodology. (ii) Validation on biological systems—we apply our methodology to some relevant cases, specifically analyzing 10 DNA base pair sequences; 10 halogenated complexes; 24 biologically relevant dimers; two molecular systems with a considerable number of electrons, a boron–nitrogen cluster (381 atoms), and the B-DNA fragment (910 atoms). All geometries considered in this stage are at equilibrium. (iii) Dissociation curve analysis—to assess the performance of our method beyond equilibrium geometries, we investigate the dissociation curves of key molecular pairs, including HF–CH_3_NH_2_, H_2_O–CH_3_NH_2_, C_4_H_4_N_2_O_2_–C_4_H_4_N_2_O_2_ (BP), and C_5_H_5_N–C_4_H_4_N_2_O_2_ (π–π interaction). This analysis provides insight into the method’s accuracy in capturing interaction strength across a range of intermolecular distances, where electron correlation effects are crucial, representing long-range interactions found, for instance, in proteins, enzymes, and DNA helices.

## 2 Data Sets Description

Our benchmark set comprises 274 chemical systems; we selected molecular systems with weak interactions of different nature, i.e., hydrogen bonds, mixed electrostatics/dispersion, and dispersion-dominated interactions (including π–π stacking). Molecular structures include atoms that participate in weak interactions, particularly in biological systems. The atoms considered are O, N, S, P, Cl, and F. The constituent data sets include:

### 2.1 A24

A set of 24 noncovalent complexes featuring interactions of a different nature, covering hydrogen bonds, mixed electrostatics/dispersion, and dispersion-dominated interactions (including π–π stacking).^70^ The complexes contain up to four second-group element atoms. CCSD­(T) interaction energies were extrapolated from a series of aug-cc-pV­(T,Q,5) basis sets.

### 2.2 HB300SPX­(S,P)

This database was built by the authors of this manuscript. We selected 50 dimers from the HB300SPX database,^71^ those systems present S and P atoms. Electrostatics/dispersion, and dispersion-dominated interactions are covered. The CCSD­(T)/CBS results are obtained using a composite scheme, where the MP2 correlation energy is extrapolated to the complete basis set limit from large basis sets.

### 2.3 HB300SPX­(Cl,F)

We considered 30 molecular systems, collected from the HB300SPX data set.^71^ Dimers contain halogen atoms (Cl, F). Electrostatics/dispersion, and dispersion-dominated interactions are included. The CCSD­(T)/CBS results are obtained using a composite scheme.

### 2.4 JSCH-2005*

82 nucleic base complexes were selected from 124 systems containing the original version.^72^ We do not consider artificial molecules (systems that have not been synthesized). We chose the dimers for which interaction energies at the CCSD­(T)/CBS level are reported and calculated with an extrapolation scheme. The systems in this data set are DNA and RNA base pairs, amino acid pairs, and S22 set.^72^

### 2.5 S66

The set contains 23 hydrogen bonds featuring all possible combinations of the most common donor and acceptor groups;^73^ 23 dispersion-dominated complexes covering π–π, aliphatic–aliphatic, and π–aliphatic interactions; and 20 complexes with mixed electrostatic/dispersion interaction. The benchmark CCSD­(T)/CBS interaction energies are based on MP2/CBS calculations in aug-cc-pVTZ and aug-cc-pVQZ basis sets and the CCSD­(T) correction calculated in the aug-cc-pVDZ basis set.

### 2.6 X40*

A set of 40 noncovalent complexes of organic halides, halohydrides, and halogen molecules where the halogens participate in a variety of interaction types.^74^ The set, named X40, covers electrostatic interactions, London dispersion, hydrogen bonds, halogen bonding, halogen−π interactions, and stacking of halogenated aromatic molecules. Interaction energies were calculated using a composite CCSD­(T)/CBS scheme where the CCSD­(T) contribution is calculated using triple-ζ basis sets with diffuse functions on all atoms but hydrogen. It should be noted that we considered only 22 dimers, excluding systems with Br and I atoms.

## 3 Theoretical Methodology

### 3.1 General Protocol

Interaction energies, *E*_int_, were computed using the supermolecule approximation,

$$E_{\mathrm{int}}=E_{\dim\mathrm{er}}-E_{\mathrm{monomer}-1}-E_{\mathrm{monomer}-2}$$ 1

where *E*_dimer_ is the electronic energy of the dimeric complex, while *E*_monomer‑1_ and *E*_monomer‑2_ correspond to the energies of the constituting monomers.

Prior to applying eq 1, we conducted geometry optimizations for all monomers at the MP2/cc-pVTZ^75^ level, a method that has shown good performance in predicting molecular structures.^76^ All optimized geometries were confirmed as true potential energy surface minima through vibrational frequency analysis (the absence of imaginary frequencies). For dimer configurations, we employed geometries from previously validated calculations, keeping these fixed during subsequent electronic structure computations to specifically isolate the interaction energy effects. We then performed single-point energy evaluations using multiple quantum chemical approaches: (1) various DFAs, (2) SAPT-DFT strategies, and (3) MP2-based methods (including our accelerated-calibrated variants), all employing correlation-consistent basis sets (aug-cc-pVXZ, where X = D, T). The MP2 and RI-MP2-based calculations, conjointly with the DFT and DFT-D computations, were done with the Orca v.4.0.2 software.^77,78^ In the case of RIJCOSX-MP2, we used a large grid in the SCF procedure. SAPT-DFT data were computed using the Psi4 quantum chemistry package.^79^ Calibration of the new RI-MP2-based methodologies was performed using a homemade script (SI) implemented in the Python 3.5 programming language.^80^

### 3.2 MP2 and RI-MP2 Calculations

The MP2 electronic energy was decomposed into three terms

$$E_{\cos,\mathrm{Css}}^{X}=E_{0}+C_{\mathrm{OS}}E_{C}^{\mathrm{OS}}+C_{\mathrm{SS}}E_{C}^{\mathrm{SS}}$$ 2

*E*_0_ is the Hartree–Fock energy, while *E*_C_^OS^ and *E*_C_^SS^ are the opposite-spin and same-spin correlation energy contributions, respectively. Each electron-correlation term is accompanied by corresponding scaling factors, *C*_OS_ and *C*_SS_, which, according to Grimme’s methodology, can be tuned up to improve the accuracy of computed interaction energies (*C*_OS_ = 1.20 and *C*_SS_ = 0.33 in the original Grimme’s formulation). Different combinations of the spin-scaling coefficients yield distinct Spin-Component-Scaled MP2 (SCS-MP2) approaches, each tailored to specific reference data sets. However, to the best of our knowledge, no dedicated calibrations have been developed for accelerated MP2 methodologies based on the RI approximation. Instead, the standard spin-scaling parameters originally proposed by Grimme are typically applied by default to RI-MP2 variants (i.e., RI-SCS-MP2).^81–83^ In addition to the MP2 and SCS-MP2 methodologies, in this work, we consider the SCS­(MI)-MP2, SCSN-MP2 and SCSC-MP2-vdW standard MP2 scaled strategies, each of them owing different pairs of scaling coefficients, conjointly with the aug-cc-pVXZ (X = D, T) basis sets for the computation of the interaction energies (eq 1) through eq 2. The accelerated approaches RI-MP2, RIJK-MP2, and RIJCOSX-MP2 were also evaluated, setting the scaling coefficients equal to one (no scaling), but also using the optimized scaling factors proposed by Grimme, i.e., RI-SCS-MP2, RIJK-SCS-MP2, and RIJCOSX-SCS-MP2. For these methodologies, we used the same basis sets as for the standard MP2 procedures; notably, they require auxiliary precalibrated basis sets for the computation of the Coulomb and/or exchange integrals; the selected auxiliary basis sets were: (1) RI-MP2: aug-cc-pVXZ/C; (2) RIJK-MP2: aug-cc-pVXZ/C RIJK def2/JK, and (3) RIJCOSX-MP2: def2/J aug-cc-pVDZ/C.

We consider it unnecessary to include basis set superposition error (BSSE) corrections in our calculations, as a substantial body of literature supports the conclusion that BSSE has a minimal impact on interaction energies—particularly for weakly bound systems and when using medium to large basis sets.^84–88^ This is confirmed in Section 1 of the SI, which clearly shows that the BSSE does not enhance the performance of spin-scaling. Based on these results, the minimization procedure appears to mitigate the influence of BSSE. Furthermore, it is important to emphasize that the methodologies proposed in this work are designed for application to biologically relevant systems. In such cases, BSSE corrections entail significant additional computational cost without yielding a corresponding improvement in the accuracy of the interaction energy descriptions. Also, Alvarez-Idaboy and Galano demonstrated that BSSE does not universally enhance interaction energies, as is commonly assumed.^84^ They concluded that BSSE corrections were not essential for qualitative or even semiquantitative accuracy. Forni et al. investigated a series of complexes (similar to those studied here) using MP2/aug-cc-pVDZ and aug-cc-pVTZ levels of theory.^85^ Their analysis revealed that BSSE contributed less than 0.5 kcal/mol on average—an effect considered negligible for typical benchmarking purposes. Furthermore, we want to emphasize the fact that the deviations were evaluated with respect to the CCSD­(T)/CBS data, the latter may incorporate the CBS effect after the optimization procedure, so the parametrized versions of our approaches indirectly include this effect, saving calculation time for further studies, since to include the BSSE it is necessary to carry out the monomer calculations with the complete dimer basis set.

In alignment with the primary objective of this study—to develop an efficient and quantitatively accurate spin-component scaled MP2 (SCS-MP2) framework tailored for the computation of weak interaction energies typically found in biological complexes—we undertook a systematic calibration of the accelerated RI-MP2 variants using our curated benchmark data set (as described in the previous section). The goal was to determine the optimal spin-scaling coefficients (*C*_OS_ and *C*_SS_) that minimize the mean absolute deviation (MAD) with respect to high-level reference values obtained at the CCSD­(T)/CBS level of theory. The MAD minimization procedure comprises the following computation scheme

$$\min_{C_{\mathrm{OS}},C_{\mathrm{SS}}}\;\frac{1}{n}\sum_{i=1}^{n}|E_{\mathrm{int}}-E_{\mathrm{ref}}|$$ 3

where *E*_int_ is the interaction energy computed through eqs 1 and 2, while *E*_ref_ is the reference energy previously obtained at the Coupled Cluster level; *n* is the size of our sample (274 species). The resulting calibrated approaches were denoted as RI-SCS-MP2^BWI‑XZ^, RIJK-SCS-MP2^BWI‑XZ^, and RIJCOSX-SCS-MP2^BWI‑XZ^, where the superscript BWI refers to Biological Weak Interactions, highlighting the specific context for which these methods were optimized, while XZ indicates the basis set used during the calibration procedure, DZ for aug-cc-pVDZ and TZ for aug-cc-pVTZ. The obtained *C*_OS_ and *C*_SS_ from this BWI study should be useful in similar molecules for the description of weak interactions, due to its diverse selection of compounds in the data set. To ensure a comprehensive and fair comparison between our proposed methods and the broader range of theoretical models considered in this study, additional comparative measures were evaluated. These included the Root Mean Squared Deviation (RMSD) as a measure of overall accuracy and the Max (MAD) descriptor, which quantifies the largest absolute deviation observed between predicted and reference interaction energies—thus capturing the worst-case performance scenario for each method.

### 3.3 DFT and SAPT-DFT Calculations

Approximations to the exact density functional are nowadays the workhorse of quantum computational chemistry owing to their excellent balance between computational cost and accuracy. Recent advances in DFAs have been focused on improving the description of weak interaction energies,^27–29,31–33^ leading to the development of a select group of functionals with enhanced performance. Several benchmarking studies have identified B97M-V,^89–91^ ωB97X-V,^92–95^ ωB97M-V,^34^ ωB97X-D3,^92^ B2PLYP-D3BJ,^96^ and DSD-BLYP-D3BJ^97^ as particularly effective, and we therefore selected them as reference methodologies for comparison with our accelerated MP2 approaches. It is worth noting that in some cases, empirical dispersion corrections were included—specifically, the D3 version of Grimme’s dispersion with the original damping function,^98^ and the D3BJ variant with Becke–Johnson damping.^99^ Besides, dispersion effects were accounted for using the D4 correction scheme;^100^ specifically, we employed the ωB97X-D4 and B2PLYP-D4 density functional methods.

Previously, it has been shown that when using Kohn–Sham orbitals, the BSSE is not significant.^101^ In addition to the previously cited evidence, we analyzed the effect of including BSSE on the estimation of interaction energies. As shown in Table S1, when using the aug-cc-pVDZ basis set, the inclusion of BSSE does not lead to a significant improvement in the deviation metrics—namely the mean absolute deviation (MAD), maximum MAD (Max­(MAD)), and root-mean-square deviation (RMSD)—compared to results obtained without BSSE correction. Moreover, upon increasing the basis set size to aug-cc-pVTZ, the inclusion of BSSE actually results in a detriment of these metrics, indicating that the BSSE correction in this context may be counterproductive. So this contribution was not considered for these calculations.

Within the Symmetry-Adapted Perturbation Theory (SAPT) framework, the total interaction energy between molecules is expressed as the sum of first- and second-order interaction components (eq 4), including electrostatic *E*_pol_^1^, induction *E*_ind_^2^, and dispersion *E*_dis_^2^ contributions, along with their respective exchange counterparts, *E*_exch_^1^, *E*_exch‑ind_^2^, and *E*_exch‑disp_^2^. These exchange terms, which arise from the quantum mechanical requirement of antisymmetry due to electron exchange between monomers at short distances, are often collectively termed Pauli repulsion. Among the various SAPT variants, SAPT-DFT offers a favorable balance between computational cost and accuracy for the study of large systems. By leveraging density-fitting techniques to circumvent the direct evaluation of four-center electron repulsion integrals, the computational scaling of SAPT-DFT can be reduced to approximately O­(*N* ^5^), where *N* is the number of electrons. The total interaction energy in SAPT-DFT is then computed accordingly to the following expression

$$E_{\mathrm{tot}}^{\mathrm{SAPT}-\mathrm{DFT}}=E_{\mathrm{pol}}^{1}+E_{\mathrm{exch}}^{1}+E_{\mathrm{ind}}^{2}+E_{\mathrm{exch}-\mathrm{ind}}^{2}+E_{\mathrm{disp}}^{2}+E_{\mathrm{exch}-\mathrm{disp}}^{2}+\delta \mathrm{HF}$$ 4

where

$$\delta \mathrm{HF}=E_{\mathrm{int}}^{\mathrm{HF}}(\mathrm{cp})-(E_{\mathrm{pol}}^{\mathrm{SAPT}0}+E_{\mathrm{exch}}^{\mathrm{SAPT}0}+E_{\mathrm{ind}}^{\mathrm{SAPT}0}+E_{\mathrm{exch}-\mathrm{ind}}^{\mathrm{SAPT}0})$$ 5

and *E*_int_^HF^(cp) is the counterpoise-corrected HF interaction energy, which is assumed to consist of electrostatics, exchange-repulsion, and induction (plus its exchange term) components. The remaining terms are the analogous interaction components calculated at the HF-SAPT (SAPT0) level with the same basis set. δHF is an approximation for higher-order induction terms and is thus added to the induction energy.

### 3.4 Cases of Study

Four cases of study were considered in this work to prove the reliability of our calibrated-accelerated methods for reproducing weak interacting energies representative of biological systems under different circumstances. First, we studied nucleobase stacking on all ten canonical B-DNA base-pair steps (Figure 1), employing the notation XY:ZW where XY and ZW represent Watson–Crick base pairs (CG or AT) in the 5′ → 3′ direction. This includes all possible combinations: AA:TT, AC:GT, AG:CT, AT:AT, CC:GG, CG:CG, GC:GC, TA:TA, TC:GA, and TG:CA. The representative AA:TT stacking geometry (adenine–thymine base pair stacked with another adenine–thymine pair) in Figure 1 illustrates the characteristic π–π interactions central to our investigation.

**Figure 1:**
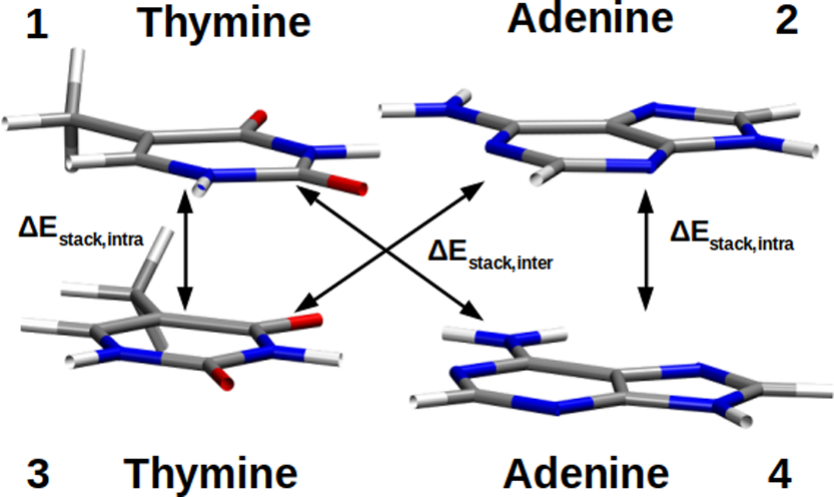
Molecular structure of the AA:TT sequence. White sticks: hydrogen atoms; gray sticks: carbon atoms; blue sticks: nitrogen atoms; red sticks: oxygen atoms.

Particularly, we computed the two-body stacking energies for each of the aforementioned base-pairs, accordingly to the following formula

$$\Delta E_{\mathrm{stack}}=\Delta E_{1,3}+\Delta E_{2,4}+\Delta E_{1,4}+\Delta E_{2,3}$$ 6

where Δ*E*_*X*,*Y*_ corresponds to the interaction energy between the two nucleobases *X* and *Y*, i.e.

$$\Delta E_{X,Y}=E_{X,Y}-\sum_{i}^{X,Y}E_{i}$$ 7

In addition, the four-body stacking energy Δ*E*_4stack_ (using the pair approximation for many-body terms) was estimated with

$$\Delta E_{4\mathrm{stack}}=E_{1,2,3,4}-E_{1,2}-E_{3,4}$$ 8

It should be noted that calculating the quantities defined in eqs 6 and 8 involves multiple computational steps, each of which may either amplify error propagation or lead to partial error cancellation.

In the second case study, we investigated the stabilization (eq 1) of benzene complexes with halogens (F_2_ and Cl_2_). We have selected these systems because halogenated complexes play an important role in many fields, such as biological systems,^9,102–104^ drug design,^105^ and other applications in engineering.^106–111^

For the third case study, we considered the database “Representative Amino-acid Side Chain Interactions” (SCAI),^112^ a set of 24 pairs of amino acid side chain interactions based on X-ray crystal structures. The pairs have been selected to represent the most typical interactions between side chains in proteins. We also studied the performance of our developed approaches in the study of a boron–nitrogen cluster and in the interaction energetics of B-DNA.

As a final benchmark, we examined the dissociation profiles of weakly bound dimer systems selected to model biologically relevant noncovalent interactions. The selected representative complexes were: (i) two hydrogen-bonded systems^74^ (HF–CH_3_NH_2_ and H_2_O–CH_3_NH_2_) and (ii) two stacked aromatic systems (C_4_H_4_N_2_O_2_–C_4_H_4_N_2_O_2_ and C_5_H_5_N–C_4_H_4_N_2_O_2_).^73,113^ The dissociation curves of hydrogen-bonded dimers were generated by scaling the equilibrium intermolecular distance (optimized at MP2/cc-pVTZ) by factors ranging from 0.90 to 2.00. The aromatic systems employed a broader scaling range (0.70–2.00) to fully characterize both short-range repulsion and long-range dispersion effects. All dissociation profiles were rigorously benchmarked against the CCSD­(T)/CBS reference data.

## 4 Results and Discussion

### 4.1 Calibration of the RI-MP2-Based Strategies, the RI-SCS-MP2^BWI‑XZ^, RIJK-SCS-MP2^BWI‑XZ^, and RIJCOSX-SCS-MP2^BWI‑XZ^ Methods

In Figure 2, we depict three heat maps as graphical representations of the dependence of each of the three comparative measurements considered in this work, with the values of the *C*_OS_ (*x*-axis) and *C*_SS_ (*y*-axis) coefficients, for one of the methods here proposed; the RIJK-MP2/aug-cc-pVDZ. Color gradients range from favorable yellow regions (lower values) to penalized dark-blue zones (higher values). The heat map in Figure 2a shows that the MAD index monotonically increases with the *C*_OS_ coefficient, indicating that the optimal value for this quantity must be zero. Conversely, there is a range where the *C*_SS_ coefficient displays low MAD values (keeping *C*_OS_ equal to zero), approximately between 1.20 and 1.80. The optimal values for these parameters found after our minimization procedure were *C*_OS_ = 0.00 and *C*_SS_ = 1.50 with a MAD value equal to 0.88 kcal/mol (RIJK-SCS-MP2^BWI‑DZ^). The broad yellow region in Figure 2a reveals significant parameter flexibility, with multiple (*C*_OS_, *C*_SS_) combinations producing chemically accurate predictions (MAD < 1 kcal/mol). This parameter robustness suggests that the RI-MP2 methodology would maintain its predictive power even when recalibrated using different reference data, if the new optimal coefficients remain within this optimal (yellow) zone. Figure 2b,c demonstrates similar parameter dependence for MAD­(MAX) and RMSD metrics, confirming the consistency of these optimal parameters across all evaluation criteria. Results for the other proposed methodologies, i.e., RIJK-SCS-MP2^BWI‑TZ^, RI-SCS-MP2^BWI‑DZ^, RI-SCS-MP2^BWI‑TZ^, RIJCOSX-SCS-MP2^BWI‑DZ^, and RIJCOSX-SCS-MP2^BWI‑TZ^, with their corresponding auxiliary basis sets, are available in the Supporting Information (Figures S1, S2, S4, S5, S7, S8, S10, S11, S13, S14, S16, and S17).

**Figure 2:**
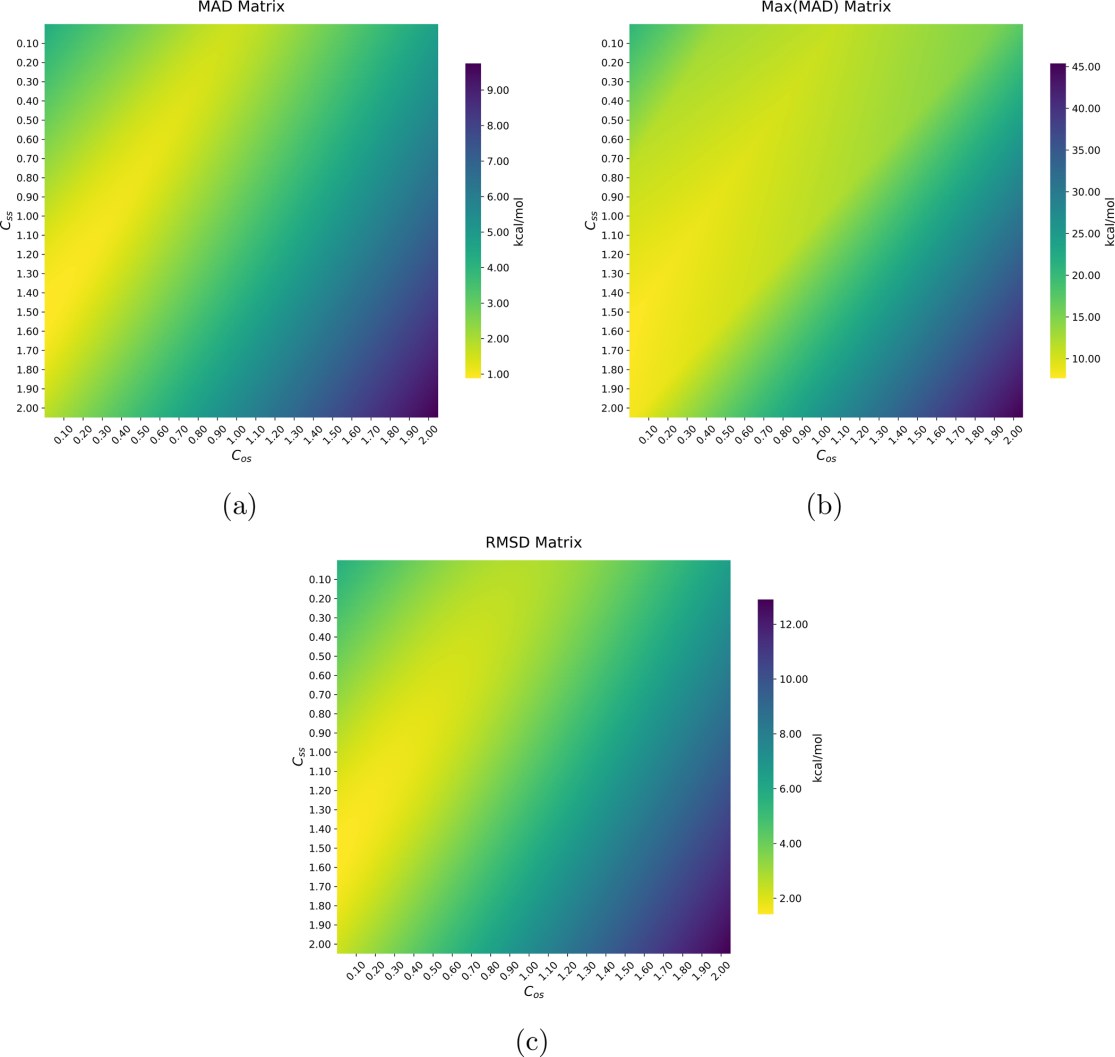
Evaluation of MADs (2a), Max­(MADs) (2b), and RMSDs (2c) in a grid of values *C*_OS_ and *C*_SS_. The optimal coefficients that minimize the MAD of the 274 molecules are *C*_OS_ = 0.00 and *C*_SS_ = 1.50. The theory level used is RIJK-MP2/aug-cc-pVDZ.

Our systematic calibration reveals a basis-set-dependent yet remarkably consistent pattern in optimal parameters across all methods. For calculations employing the aug-cc-pVDZ basis set, we observe notable parameter consistency across methods: SCS-MP2^BWI‑DZ^, RI-SCS-MP2^BWI‑DZ^, and RIJK-SCS-MP2^BWI‑DZ^ all converge to identical coefficients (*C*_OS_ = 0.00, *C*_SS_ = 1.50), with only marginal variation for RIJCOSX-SCS-MP2^BWI‑DZ^ (*C*_SS_ = 1.47). Our results suggest that, for the class of biologically relevant weakly bound systems studied, the contribution of the opposite-spin (OS) correlation energy within the MP2 framework is comparatively small in achieving agreement with CCSD­(T)/CBS reference interaction energies. It is important to note that the low or even vanishing scaling coefficients obtained for the OS component should not be interpreted as a dismissal of its physical relevance. Instead, this attenuation can be understood as an effective empirical correction that compensates for the known overestimation of the correlation energy in conventional MP2, thereby enhancing the overall predictive accuracy of the method for these systems. In contrast, the aug-cc-pVTZ basis set yields slightly modified optimal parameters (*C*_OS_ = 0.27, *C*_SS_ = 1.38) for the first three variants, while RIJCOSX-SCS-MP2^BWI‑TZ^ shows, as before, distinct values (*C*_OS_ = 0.17, *C*_SS_ = 1.59). This basis-set-dependent behavior carries important theoretical implications: the near-zero *C*_OS_ values with aug-cc-pVDZ indicate that weak interactions in these systems are governed almost entirely by long-range nondynamical correlation, with negligible short-range dynamical correlation contributions. The modest parameter shifts observed with aug-cc-pVTZ may reflect enhanced basis set sensitivity to short-range effects. Remarkably, all six methodological variants maintain MAD < 1 kcal/mol, demonstrating the robustness of our parametrization scheme across both methodological implementations and basis set choices. This comprehensive validation establishes our approach as reliable for modeling diverse noncovalent interactions in chemical and biological systems.

### 4.2 Comparative Performance Assessment

In this stage, we benchmarked the performance of our six calibrated models against a range of sophisticated and quantitatively accurate computational methods for predicting weak interaction energies. This evaluation was carried out using our curated training data set, comprising 274 interaction energies, as described in the Theoretical Methodology section. A summary of the comparative metrics for all tested computational protocols is presented in Figure 3.

**Figure 3:**
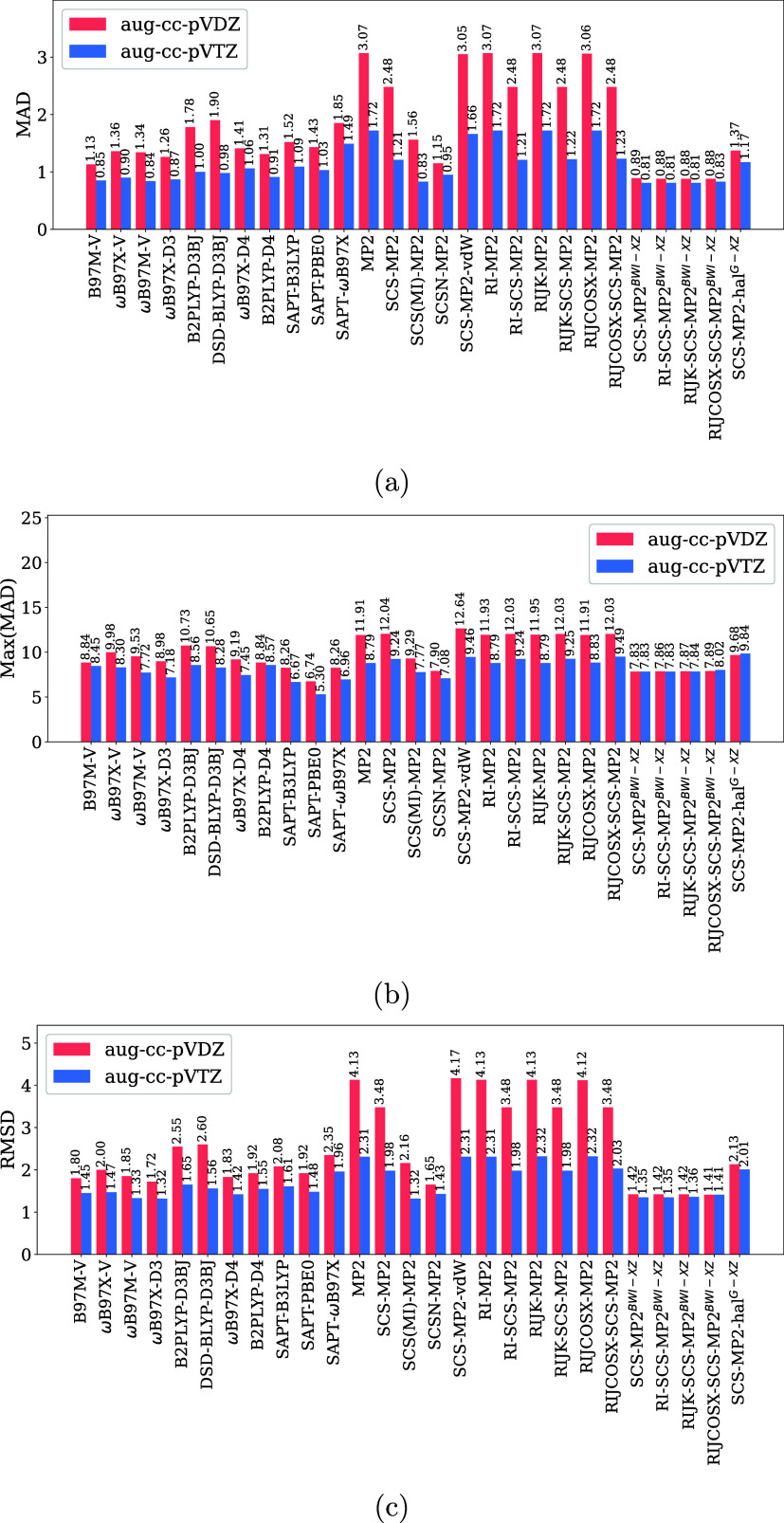
(a) MAD, (b) Max­(MAD), and (c) RMSD obtained for methodologies employed in this work with respect to CCSD­(T)/CBS. Data: 274 interaction energies. MP2: *C*_OS_ = *C*_SS_ = 1.00; SCS-MP2: *C*_OS_ = 1.20, *C*_SS_ = 0.33; SCS­(MI)-MP2: *C*_OS_ = 0.40, *C*_SS_ = 1.29; SCSN-MP2: *C*_OS_ = 0.00, *C*_SS_ = 1.76; SCS-MP2-vdW: *C*_OS_ = 1.28, *C*_SS_ = 0.50; SCS-MP2-hal^G‑DZ^: *C*_OS_ = 0.60 *C*_SS_ = 0.80; SCS-MP2^BWI‑DZ^, RI-SCS-MP2^BWI‑DZ^, RIJK-SCS-MP2^BWI‑DZ^: *C*_OS_ = 0.00, *C*_SS_ = 1.50; RIJCOSX-SCS-MP2^BWI‑DZ^: *C*_OS_ = 0.00, *C*_SS_ = 1.47; SCS-MP2-hal^G‑TZ^: *C*_OS_ = 1.20 *C*_SS_ = 0.20; SCS-MP2^BWI‑TZ^, RI-SCS-MP2^BWI‑TZ^, RIJK-SCS-MP2^BWI‑TZ^: *C*_OS_ = 0.27, *C*_SS_ = 1.38; RIJCOSX-SCS-MP2^BWI‑TZ^: *C*_OS_ = 0.17, *C*_SS_ = 1.59. All values in kcal/mol.

We first analyzed results obtained with the aug-cc-pVDZ basis (represented by pink bars in Figure 3) to analyze the effect of increasing the basis set on the performance of the methodologies under scrutiny. Unscaled MP2 methods showed the highest MAD and RMSD values (Figure 3a,c), respectively. The maximum absolute deviation (Max­(MAD); Figure 3b) was also notably high for unscaled MP2, although in some cases, it was comparable to those observed with DFAs. Interestingly, accelerated MP2 variants demonstrated performance nearly equivalent to standard MP2. For instance, RIJCOSX-MP2 deviated by only 0.01 kcal/mol in MAD compared to conventional MP2. When Grimme’s global scaling was applied to MP2-based methods, both standard and accelerated variants benefited equally, resulting in a 0.59 kcal/mol reduction in MAD. Further improvements were observed with spin-component scaling approaches. Specifically, SCS­(MI)-MP2 and SCSN-MP2 reduced the MAD by 1.51 and 1.92 kcal/mol, respectively, with respect to MP2. Similar trends were observed in RMSD and Max­(MAD).

Results with the aug-cc-pVDZ basis also indicate that among the tested DFAs, several demonstrated outstanding accuracy—often outperforming MP2-based methods and closely matching the results obtained with SAPT-DFT. Particularly, the B97M-V and ωB97X-D3 functionals yielded MAD values of 1.13 and 1.26 kcal/mol, and RMSD values of 1.80 and 1.72 kcal/mol, respectively. These were followed closely by ωB97X-V, ωB97M-V, and ωB97X-D4. B97M-V is a local meta-GGA functional augmented with nonlocal VV10 correlation, while the ωB97X-V, ωB97M-V, and ωB97X-D3 functionals incorporate different rank-separated hybrid schemes—RSH GGA VV10, RSH meta-GGA VV10, and RSH GGA with conventional hybrid exchange, respectively. The data suggest that introducing RSH effects can have a slightly detrimental effect on MAD values for the systems examined. This may also be due, in part, to the fact that ωB97X-V and ωB97M-V do not satisfy the uniform electron gas (UEG) limit, a condition met by B97M-V and ωB97X-D3. Meeting the UEG limit is essential for accurately describing noncovalent interactions using DFAs. This condition guarantees the correct asymptotic and bulk behavior of the exchange–correlation (XC) energy in regions of nearly constant or slowly varying electron density—scenarios commonly encountered in noncovalent systems such as dispersion-bound complexes, hydrogen bonds, and π–π interactions. By satisfying the UEG limit, DFAs better capture the subtle correlation effects critical to these weak interactions, thereby improving both the accuracy and transferability across diverse chemical environments. If a DFA fails to recover the UEG limit, it can over- or underestimate correlation energy, leading to significant errors in binding energies, especially in noncovalently bound systems where energetic contributions are small and highly sensitive to XC treatment. Surprisingly, the double-hybrid functionals evaluated generally performed worse than those of the tested GGA and RSH-DFAs. It is commonly assumed that climbing Jacob’s ladder of DFAs yields systematically improved predictions of weak interaction energies. However, in practice, this trend is often disrupted when dispersion corrections are added externally, as these corrections are not inherently part of the Jacob’s ladder framework. In such cases, the performance of different rungs may become flattened or even inverted due to the nonlocal nature of dispersion and the empirical tuning of the correction parameters. Our results reflect this well-established behavior, rather than being an artifact of basis set incompleteness.

Meanwhile, SAPT-DFT, although not the best in terms of average error metrics, consistently yielded the lowest Max­(MAD) values, indicating a more uniform predictive behavior. These findings suggest that combining Hartree–Fock exchange with semilocal exchange functionals and MP2 correlation with semilocal correlation functionals—as is typical in double-hybrids—can negatively affect MAD. However, SAPT-DFT methodologies appear to mitigate large errors more effectively, likely due to their distinct energy decomposition scheme, which helps isolate and control dominant sources of error.

The pink bars in Figure 3 demonstrate that our recalibrated RI-SCS-MP2^BWI‑DZ^ methods achieve superior performance in predicting the 274 weak interaction energies when using the double-ζ basis set. All RI-SCS-MP2^BWI‑DZ^ variants yield exceptional accuracy, with the lowest MAD (0.88 kcal/mol) and RMSD (1.42 kcal/mol) values among the tested methods. Notably, these implementations also match SAPT-DFT’s reliability in maximum error control (Max­(MAD)), while even the RIJCOSX-SCS-MP2^BWI‑DZ^ version outperforms most alternative approaches. This consistent performance across our recalibrated methods reveals a fundamental characteristic of biologically relevant weak interactions: their energies exhibit an evident systematic dependence on spin-component correlation patterns. Crucially, these results validate accelerated MP2 methodologies as practical tools for obtaining CCSD­(T)/CBS-quality interaction energies in large systems, combining computational efficiency with chemical accuracy.

Our analysis of the results obtained with the larger aug-cc-pVTZ basis set (represented by blue bars in Figure 3) reveals the anticipated improvement in accuracy across most methods while preserving the general trends observed with double-ζ calculations. As expected, unscaled MP2 methodologies continue to demonstrate the weakest performance, exhibiting the highest values across all comparative metrics. While the application of Grimme’s scaling coefficients yields measurable improvements for these MP2 approaches, they remain uncompetitive compared to other methodologies in the study.

The B97 family of DFAs demonstrates superior performance compared to both double-hybrid functionals and SAPT-DFT methodologies, with the B97M-V and ωB97M-V approximations showing particularly excellent accuracy. While these top-performing DFAs generally provide comparable results for weak interaction energies, our recalibrated spin-component-scaled MP2 methods achieve even better performance. Notably, these optimized MP2 approaches surpass even the quantitatively accurate B97M-V and ωB97M-V functionals in overall accuracy, while maintaining competitive maximum deviations, in some cases exhibiting smaller extreme errors than the DFA benchmarks.

A key advantage of our recalibrated methods is their remarkable insensitivity to the basis set size. The performance metrics show minimal variation when expanding from double-ζ to triple-ζ basis sets, with two particularly noteworthy findings: (1) results obtained using the smaller aug-cc-pVDZ basis are comparable to—and in some cases superior to—those from other methods employing the larger aug-cc-pVTZ basis, and (2) no loss of accuracy consistency is observed (Figure 3b). This robustness is exemplified by the RIJK-SCS-MP2^BWI‑DZ^ method, which, with the smaller basis set, achieves near-identical mean accuracy (ΔMAD = 0.04 kcal/mol) to the quantitatively accurate B97M-V/aug-cc-pVTZ method while demonstrating significantly better error uniformity (ΔMax­(MAD) > 0.5 kcal/mol). This exceptional performance highlights three key advantages of our approach: (i) the ability to obtain high-quality results with computationally economical basis sets, (ii) superior reliability across diverse weak interaction types compared to even the most sophisticated alternative methods, and (iii) inherently lowering computational costs.

Our computational timing analysis (Figure 4) reveals several important trends regarding method efficiency. Among all methods, canonical MP2/aug-cc-pVDZ exhibits the highest computational cost (122.00 s), though this is dramatically reduced through resolution-of-the-identity techniques. The RI-MP2 implementation cuts computation time to 32.86 s, with the RIJK-MP2/aug-cc-pVDZ approach achieving a remarkable 1613.76% reduction relative to RI-MP2 while maintaining accuracy. Notably, RIJK-MP2 outperforms several DFAs (ωB97X-V, ωB97M-V, ωB97X-D3, B2PLYP-D3BJ, DSD-BLYP-D3BJ) in both speed and accuracy, while RIJCOSX-MP2 offers intermediate performance between RI-MP2 and RIJK-MP2. The B97M-V functional emerges as the most computationally efficient approach, requiring significantly less time than other DFAs. The ωB97X-V, ωB97M-V, ωB97X-D3, and ωB97X-D4 functionals show computational demands similar to each other, while double-hybrid functionals (B2PLYP-D3BJ and DSD-BLYP-D3BJ) prove more efficient than the range-separated counterparts. When expanding to the aug-cc-pVTZ basis set, B97M-V remains the most efficient method (4.71 s), followed by RIJCOSX-MP2 (22.26 s), with all methods showing the expected increase in computational demand while preserving their relative performance rankings.

**Figure 4:**
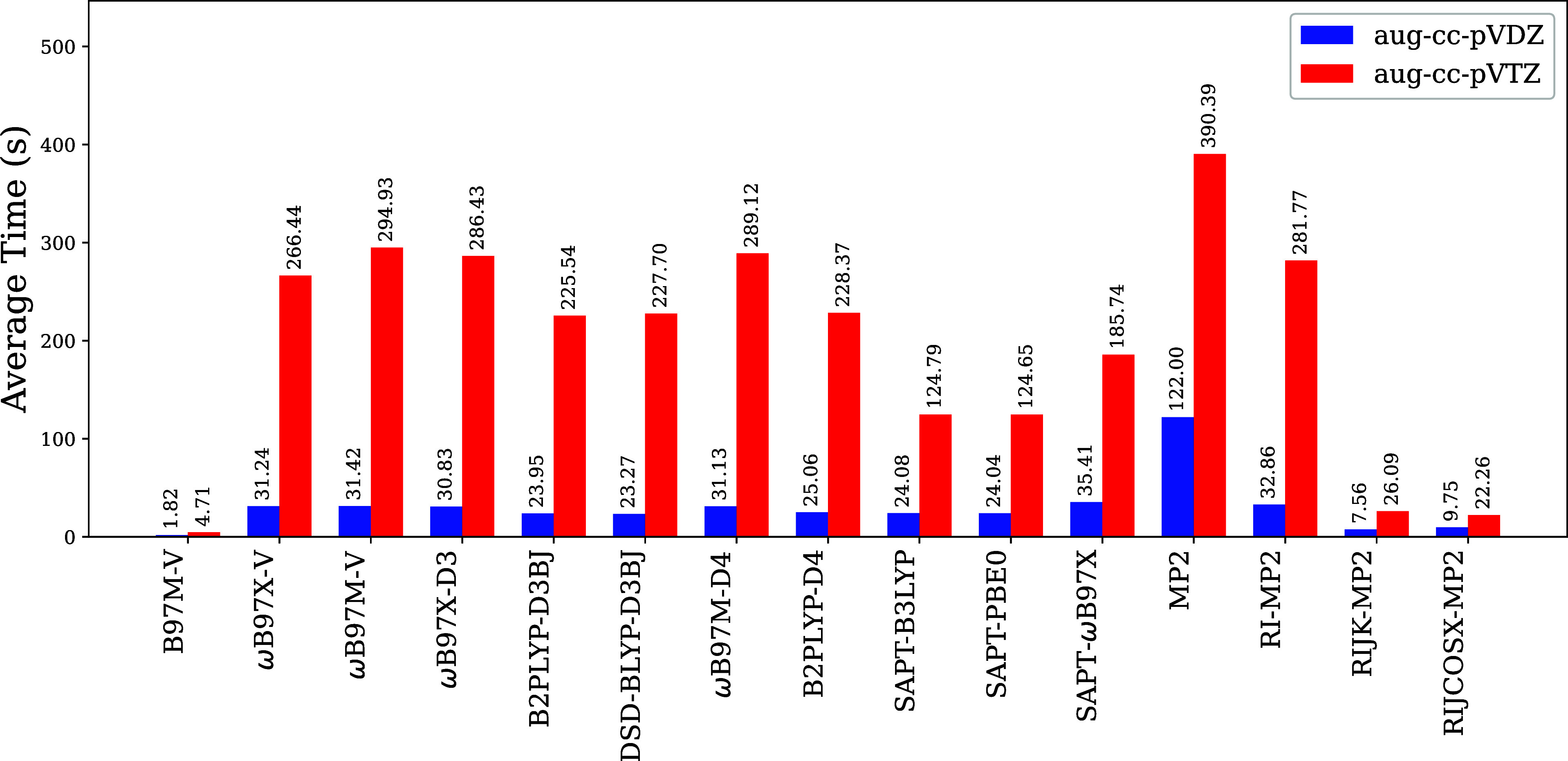
Comparison of computational times (total run time) for different methods and basis sets in the 274 molecules. Estimated time per processor (Intel Xeon Platinum 8168). All values in seconds. The RAM to all processes was 60 GB.

Based on our comprehensive analysis, we strongly recommend the RIJCOSX-SCS-MP2^BWI‑DZ^ approach as an optimal balance between accuracy and computational efficiency for studying weak interactions. This methodology demonstrates several key advantages: (1) it delivers accuracy comparable to or exceeding that of the most precise methods employing the larger aug-cc-pVTZ basis set; (2) it maintains superior reliability across diverse molecular systems; (3) it achieves this performance at computational costs similar to, or even lesser than conventional density functional approaches; and (4) unlike DFAs, which often require extensive parametrization, our approach achieves comparable or superior accuracy through calibration of just two key parameters (*C*_OS_ and *C*_SS_ coefficients). The reduced parameter space not only enhances the method’s extensibility to diverse chemical systems but also improves its reliability by minimizing potential overfitting artifacts. This unique combination of features enables the computation of interaction energies with near-CCSD­(T)/CBS accuracy while maintaining DFT-level computational efficiency, making it particularly valuable for quantitatively accurate studies of a broad spectrum of weak interaction types present in biological molecular systems from medium to large size.

## 5 Applications

### 5.1 Case Study 1: π-Stacking Interaction Energies between DNA Nucleobases

Our first case study was nucleobase π-stacking, which is crucial for stabilizing DNA structure.^114–117^ The B-DNA double helix features ten unique base-pair step sequences, where consecutive stacked base pairs (e.g., AA:TT, CG:CG) exhibit distinct stacking geometries and interaction energies.^118^ These stacking interactions critically influence DNA conformation and dynamics, yet despite decades of research, their energetic landscape remains incompletely understood.^114^ nucleobase stacking inherently presents significant challenges for DFT, given that the exponential decay of electron density requires either (1) proper asymptotic behavior of the exchange–correlation kernel (rarely achieved in standard functionals) and/or (2) explicit dispersion corrections.^119^ Even advanced functionals like M06-2X require additional long-range dispersion treatments for reliable performance.^73,113^ As mentioned in our Theoretical Methodology section, we computed the two and four-body stacking interaction energies (eqs 6 and 8, respectively) between DNA Nucleobases. Results were confronted with reference data computed at the DLPNO-CCSD­(T)/CBS level of theory.

Building upon the analysis presented in the previous section, where functionals such as B97M-V, ωB97X-V, ωB97M-V, and ωB97X-D3, along with the newly developed RI-SCS-MP2^BWI‑DZ^, RIJK-SCS-MP2^BWI‑DZ^, and RIJCOSX-SCS-MP2^BWI‑DZ^ methods, demonstrated excellent accuracy for weakly interacting systems at a modest computational cost, we now test these methods on biologically relevant systems. According to Table 1, B97M-V achieved the best performance among the tested DFAs for Δ*E*_stack_, with a MAD of 2.21 kcal/mol. Despite the inclusion of range-separated Hartree–Fock exchange in ωB97X-V, ωB97M-V, and ωB97X-D3, these functionals showed inferior accuracy compared to B97M-V. This suggests that for π-stacking interactions between DNA base pairs, the inclusion of both long- and short-range HF exchange does not necessarily lead to improved performance. Notably, the best-performing DFAs—B97M-V and ωB97X-D3—satisfy the UEG limit, unlike ωB97X-V and ωB97M-V, which may partly explain their improved performance. One may observe that the RMSD metric showed a trend consistent with MAD.

**Table 1:** Deviations Obtained for DFT, MP2 and Its Variants with Respect to DLPNO-CCSD­(T)/CBS^120^ (Δ*E*_stack_ and Δ*E*_4stack_) and CCSD­(T)/CBS^121^ (Halogenated Systems and SCAI database)^a^

method	MAD	Max(MAD)	RMSD
Δ*E*_stack_			
B97M-V	2.21	2.33	2.21
ωB97X-V	2.87	3.10	2.88
ωB97M-V	3.43	3.70	3.44
ωB97X-D3	2.71	3.04	2.72
RI-SCS-MP2^BWI‑DZ^	1.96	2.26	1.96
RIJK-SCS-MP2^BWI‑DZ^	1.98	2.30	1.99
RIJCOSX-SCS-MP2^BWI‑DZ^	1.97	2.30	1.98
Δ*E*_4stack_			
B97M-V	2.89	3.16	2.89
ωB97X-V	3.60	3.86	3.60
ωB97M-V	4.25	4.56	4.25
ωB97X-D3	3.37	3.79	3.37
RI-SCS-MP2^BWI‑DZ^	2.87	3.05	2.87
RIJK-SCS-MP2^BWI‑DZ^	2.90	3.09	2.91
RIJCOSX-SCS-MP2^BWI‑DZ^	2.88	3.07	2.86
Δ*E*_int_—halogenated systems			
B97M-V	0.68	1.38	0.84
ωB97X-V	0.39	0.48	0.39
ωB97M-V	0.51	0.80	0.54
ωB97X-D3	0.32	0.66	0.43
RI-SCS-MP2^BWI‑DZ^	0.22	0.49	0.27
RIJK-SCS-MP2^BWI‑DZ^	0.22	0.48	0.27
RIJCOSX-SCS-MP2^BWI‑DZ^	0.22	0.48	0.27
Δ*E*_int_—SCAI database			
B97M-V	2.33	21.12	10.48
ωB97X-V	1.92	11.55	6.66
ωB97M-V	1.95	11.69	6.60
ωB97X-D3	2.04	11.15	6.47
RI-SCS-MP2^BWI‑DZ^	1.08	8.57	4.04
RIJK-SCS-MP2^BWI‑DZ^	1.09	8.62	4.05
RIJCOSX-SCS-MP2^BWI‑DZ^	1.08	8.56	4.04

a For Δ*E*_stack_ and Δ*E*_4stack_, 10 nucleobase pairs were considered (arithmetic mean: Δ*E*_stack_ = −14.36 and Δ*E*_4__stack_= −12.77 kcal/mol); for Δ*E*_int_ in halogenated systems, we considered 10 systems. There are 24 dimers in the SCAI database. All values in kcal/mol.

The Max­(MAD) value in all of the DFAs in Table 1 was observed for the GC:GC system, consisting of two guanine–cytosine base pairs stacked atop one another (Figure 5a). In contrast, our RI-SCS-MP2^BWI‑DZ^, RIJK-SCS-MP2^BWI‑DZ^, and RIJCOSX-SCS-MP2^BWI‑DZ^ methods outperformed the best DFA (B97M-V), reducing the MAD by 0.25, 0.23, and 0.24 kcal/mol, respectively. This improvement indicates that our methods recover a portion of the interaction energy not captured by B97M-V. Interestingly, the largest error for both of our MP2-based approaches occurred in the AA:TT system, which involves two stacked adenine–thymine base pairs (Figure 5b). For the Δ*E*_4stack_ energies, B97M-V, RI-SCS-MP2^BWI‑DZ^, RIJK-SCS-MP2^BWI‑DZ^, and RIJCOSX-SCS-MP2^BWI‑DZ^ all yielded nearly identical MAD values, indicating robust performance across these methods. In contrast, ωB97M-V showed significantly larger deviations from the DLPNO-CCSD­(T)/CBS reference data. Among DFAs, the CG:CG (Figure 5c) system contributed the largest Max­(MAD), whereas for both of our MP2-based approaches, the AA:TT configuration once again showed the largest deviation. Despite using only two adjustable parameters—*C*_OS_ and *C*_SS_—our RI-SCS-MP2^BWI‑DZ^, RIJK-SCS-MP2^BWI‑DZ^, and RIJCOSX-SCS-MP2^BWI‑DZ^ methodologies delivered predictions that match or surpass the accuracy of state-of-the-art DFAs, including those positioned at the top of Perdew’s “Jacob’s Ladder” of DFT. These findings highlight the potential of our refined RI-MP2-based strategies for studying biologically relevant, noncovalent interactions with both accuracy and efficiency.

**Figure 5:**
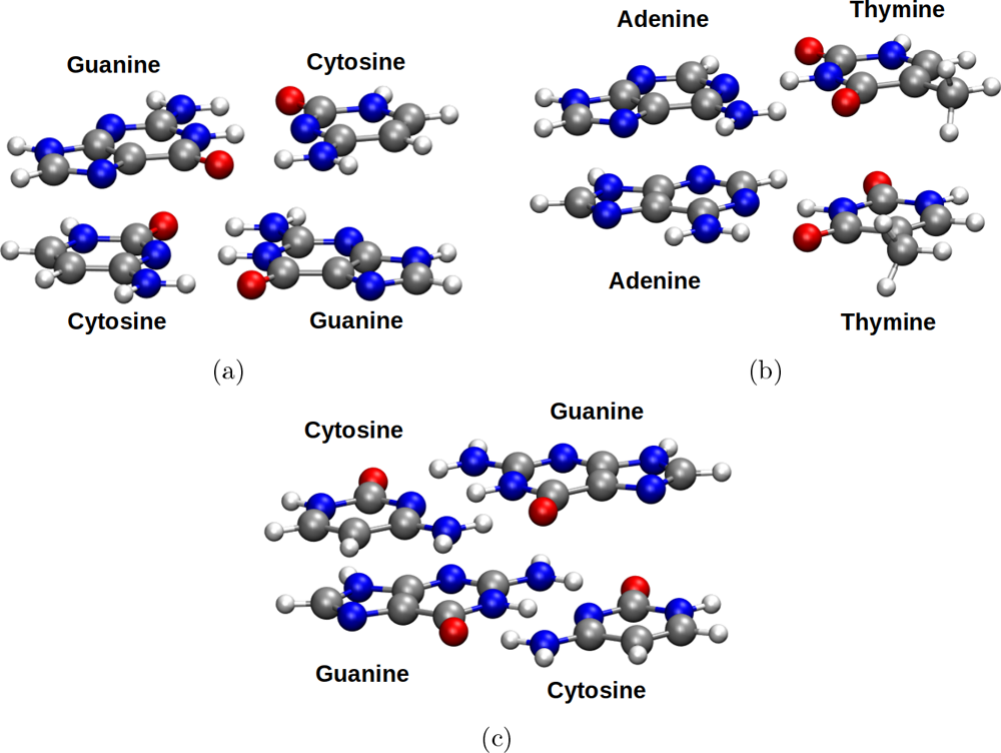
Molecular structures of (a) GC:GC, (b) AA:TT, and (c) CG:CG. White spheres: hydrogen atoms; gray spheres: carbon atoms; blue spheres: nitrogen atoms; red spheres: oxygen atoms.

### 5.2 Case Study 2: Halogenated Molecular Systems

In the second case study, we evaluated the interaction energies of halogenated molecular systems to account for the role of halogen bonding in biological interactions. It is known that these interactions critically enhance both the binding selectivity and the affinity between guests and receptors. Halogenated compounds leverage this phenomenon to achieve targeted molecular recognition, making them promising molecular entities in life sciences—from pharmaceutical design to biomolecular engineering. As shown in Table 1, all methods under consideration exhibited excellent predictive performance, with MADs below 1.00 kcal/mol. Notably, the RI-SCS-MP2^BWI‑DZ^, RIJK-SCS-MP2^BWI‑DZ^, and RIJCOSX-SCS-MP2^BWI‑DZ^ approaches developed in this work achieved superior accuracy, reducing the MAD by 0.22 kcal/mol with respect to reference data. This represents a significant improvement over the tested DFAs, underscoring the effectiveness of our methods in capturing the subtle energetics of halogen bonding.

Note that for B97M-V, ωB97M-V, and our MP2-based approaches, the system contributing the highest Max­(MAD) was C_6_H_6_–Cl_2_–Ta, where the Cl_2_ molecule is oriented perpendicular to the benzene ring (Figure 6). For ωB97X-D3, the largest deviation occurs for C_6_H_6_–Cl_2_–Sb, with a similar perpendicular geometry. Meanwhile, ωB97X-V shows its largest deviation in the C_6_H_6_–Cl_2–_Tb system, again with Cl_2_ situated out of plane relative to the aromatic ring. These trends suggest that the nature and spatial orientation of the halogen interactions significantly influence the accuracy of the DFAs. In contrast, the RI-SCS-MP2^BWI‑DZ^, RIJK-SCS-MP2^BWI‑DZ^, and RIJCOSX-SCS-MP2^BWI‑DZ^ methods consistently provide more reliable interaction energies, regardless of the specific molecular geometry or spatial orientation.

**Figure 6:**
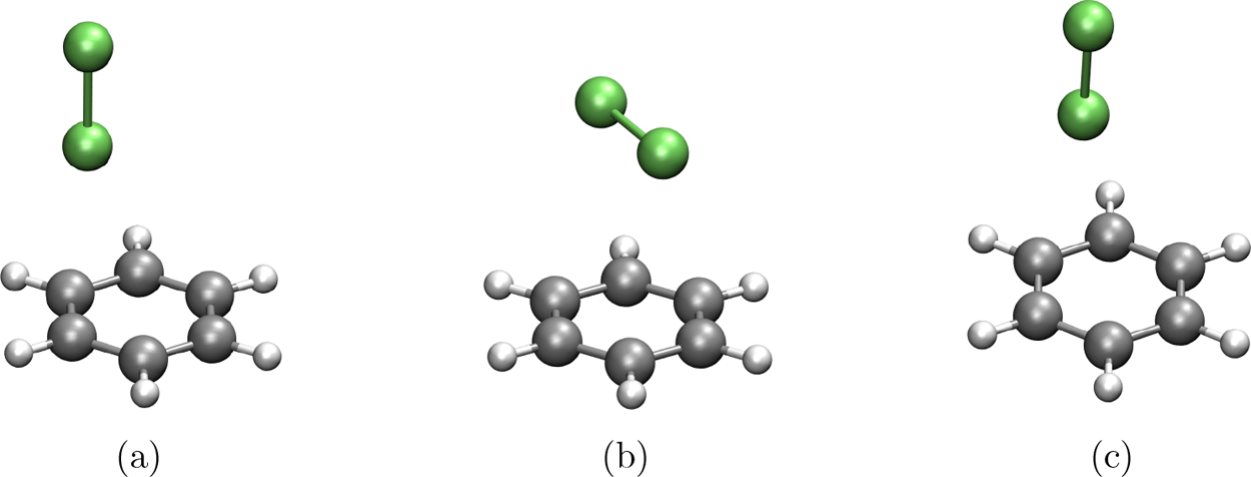
Molecular structures of (a) C_6_H_6_–Cl_2_–Ta, (b) C_6_H_6_–Cl_2_–Sb, and (c) C_6_H_6_–Cl_2_–Tb. Ta and Tb are two different T-shaped structures, and Sb is a sandwich structure. White spheres: hydrogen atoms; gray spheres: carbon atoms; green spheres: chlorine atoms.

### 5.3 Case Study 3: SCAI Data Set

The third test case involves determining the interaction energies for the Representative Amino-acid Side Chain Interactions (SCAI) database, which consists of 24 biologically relevant dimers. This data set provides an appropriate benchmark for assessing the performance of the proposed methodologies on systems resembling real biological interactions. As shown in Table 1, the deviation metrics for RI-SCS-MP2^BWI‑DZ^ (MAD = 1.08, Max­(MAD) = 8.57, RMSD = 4.04 kcal/mol), RIJK-SCS-MP2^BWI‑DZ^ (MAD = 1.09, Max­(MAD) = 8.62, RMSD = 4.05 kcal/mol), and RIJCOSX-SCS-MP2^BWI‑DZ^ (MAD = 1.08 kcal/mol, Max­(MAD) = 8.56 kcal/mol, RMSD = 4.04 kcal/mol) demonstrate superior performance compared to those of the other evaluated methods. In contrast, the B97M-V DFA exhibits the poorest performance with a Max­(MAD) of 21.12 kcal/mol, corresponding to the 703-KE1 molecular system (Figure 7a). For RI-SCS-MP2^BWI‑DZ^, RIJK-SCS-MP2^BWI‑DZ^, RIJCOSX-SCS-MP2^BWI‑DZ^, and the remaining DFAs, Max­(MAD) is associated with the 705-DH complex (Figure 7b). Overall, the methodologies developed in this work show excellent performance on the SCAI data set, reliably capturing the interaction energies of weakly bound, biologically inspired molecular systems.

**Figure 7:**
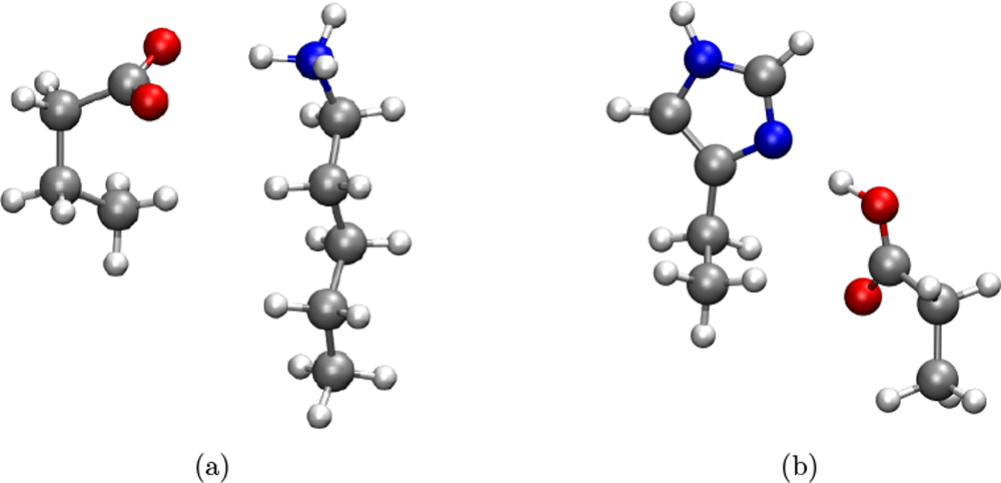
Molecular structures of (a) 703-KE1, and (b) 705-DH. White spheres: hydrogen atoms; gray spheres: carbon atoms; blue spheres: nitrogen atoms; red spheres: oxygen atoms.

Finally, for the systems shown in Figure 8, we evaluated the performance of the RIJCOSX-SCS-MP2^BWI‑DZ^ approach in predicting the interaction energies. For the absorption cluster depicted in Figure 8a, which contains a total of 381 atoms, the reference interaction energy obtained at the CIM-DLPNO-CCSD­(T)//RI-MP2 level is −17.83 kcal/mol. The RIJCOSX-SCS-MP2^BWI‑DZ^ method yields an interaction energy of −15.62 kcal/mol for the boron–nitrogen cluster, showing a deviation of 2.21 kcal/mol from the reference value. In contrast, the RIJCOSX-SCS-MP2^BWI‑TZ^ approach provides a slightly improved result of −16.02 kcal/mol with a reduced deviation of 1.81 kcal/mol. For the B-DNA double helix system (910 atoms, Figure 8b), the reference interaction energy is −416.08 kcal/mol. The RIJCOSX-SCS-MP2^BWI‑DZ^ and RIJCOSX-SCS-MP2^BWI‑DZ^ methods yield interaction energies of −408.91 and −409.83 kcal/mol, corresponding to deviations of 7.17 and 6.25 kcal/mol, respectively. These results highlight the capability of the proposed method to reliably estimate interaction energies in large, biologically relevant systems with acceptable accuracy and significantly reduced computational cost compared to high-level wave function methods.

**Figure 8:**
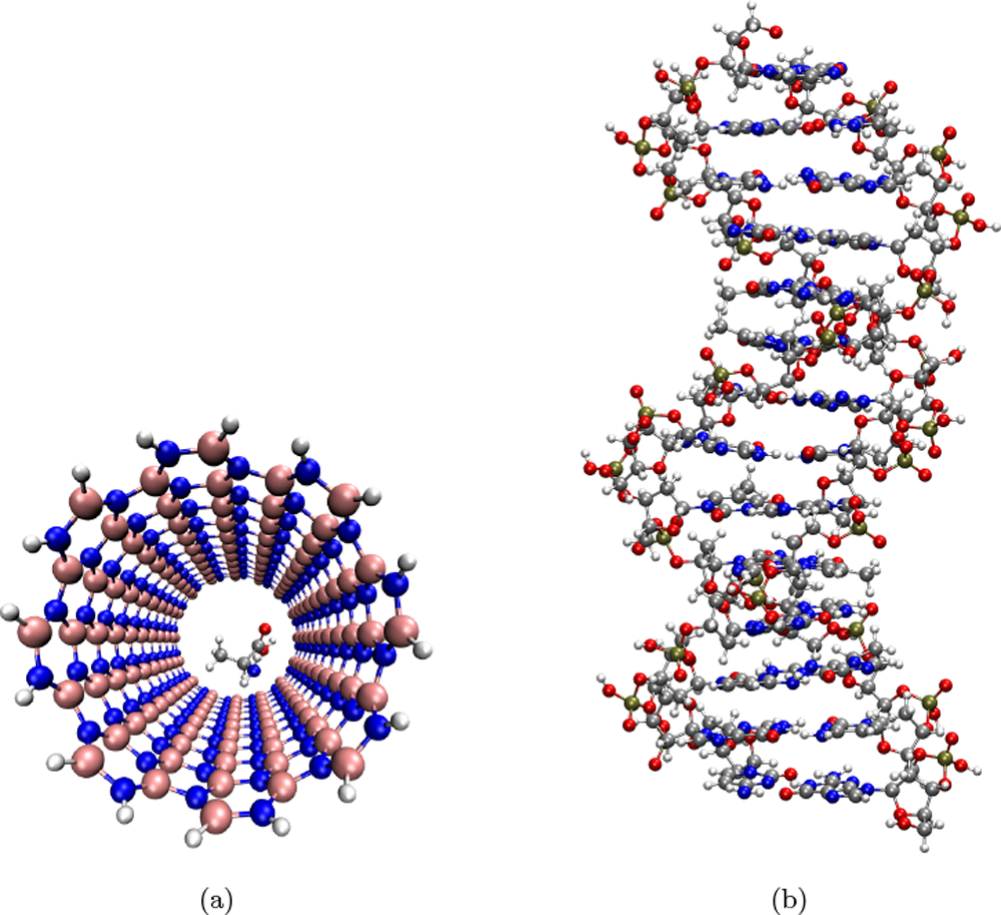
Molecular structures of (a) B–N cluster absorption, and (b) B-DNA. White spheres: hydrogen atoms; pink spheres: boron atoms; gray spheres: carbon atoms; blue spheres: nitrogen atoms; red spheres: oxygen atoms; brown spheres: phosphorus atoms.

### 5.4 Case Study 4: Non-Equilibrium Interaction Energies: Dissociation Curves

As a fourth case study, we extend our analysis beyond equilibrium geometries to investigate the performance of the proposed methodologies in nonequilibrium regimes. Specifically, we examine the dissociation energy profiles for a series of representative molecular dimers: HF–CH_3_NH_2_, H_2_O–CH_3_NH_2_, C_4_H_4_N_2_O_2_–C_4_H_4_N_2_O_2_ (BP), and C_5_H_5_N–C_4_H_4_N_2_O_2_ (π–π). Accurately reproducing dissociation curves presents a significant challenge for electronic structure methods, as the balance between dynamic and static electron correlation varies substantially across the potential energy surface. Such systems are particularly important for understanding biological processes in which cooperative, long-range interactions between molecular fragments can drive conformational changes. Dissociation curves were computed using conventional MP2, spin-component scaled MP2 (SCS-MP2), and our RIJK-SCS-MP2^BWI‑DZ^ method, with reference data taken from high-level CCSD­(T)/CBS calculations previously reported in the literature (refs 58 and 96 ; results are summarized in Figure 9.

**Figure 9:**
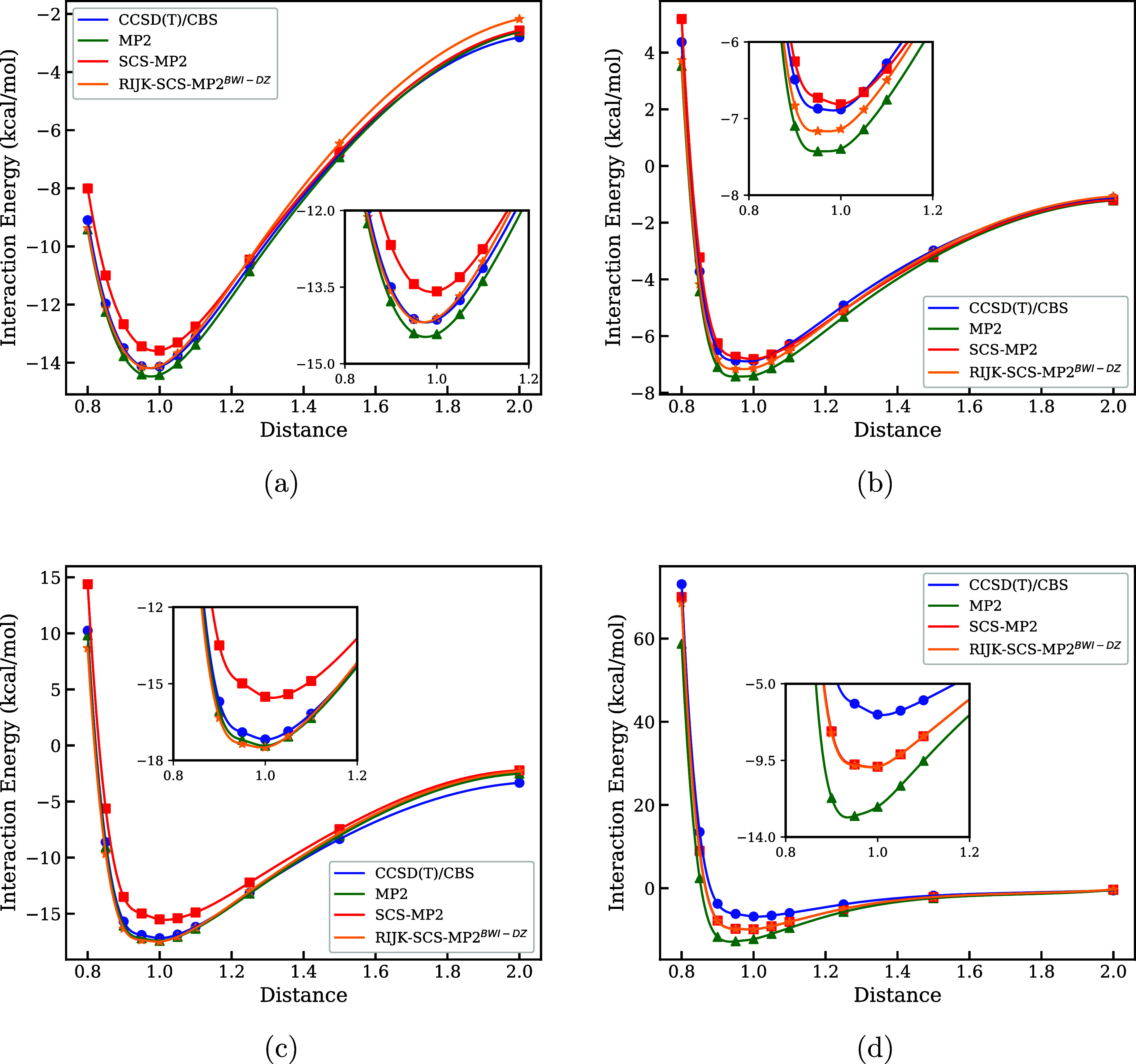
Dissociation curves for (a) HF–CH_3_NH_2_, (b) H_2_O–CH_3_NH_2_, (c) C_4_H_4_N_2_O_2_–C_4_H_4_N_2_O_2_-BP, and (d) C_5_H_5_N–C_4_H_4_N_2_O_2_–π–π obtained for CCSD­(T)/CBS and MP2, SCS-MP2/aug-cc-pVDZ and RIJK-SCS-MP2^BWI-DZ^.

In the case of the HF-CH_3_NH_2_ dimer (Figure 9a), RIJK-SCS-MP2^BWI‑DZ^ closely reproduces the reference CCSD­(T)/CBS curve at short intermolecular distances, capturing the interaction energy profile near equilibrium with high accuracy. In contrast, MP2 tends to overestimate, while SCS-MP2 underestimates the interaction energy in this region. As the monomers move further apart, both MP2 and SCS-MP2 converge toward the reference curve, whereas RIJK-SCS-MP2^BWI‑DZ^ slightly diverges at larger separations, indicating that static correlation is not being optimally scaled, although the difference between the reference CC data is less than 0.8 kcal/mol. For the H_2_O–CH_3_NH_2_ dimer (Figure 9a), both SCS-MP2 and RIJK-SCS-MP2^BWI‑DZ^ maintain excellent agreement with CCSD­(T)/CBS across the full dissociation curve, while MP2 consistently overestimates the interaction strength. Notably, despite significant differences in the scaling coefficients—*C*_OS_ = 1.20 and *C*_SS_ = 0.33 for SCS-MP2 versus *C*_OS_ = 0.00 and *C*_SS_ = 1.50 for RIJK-SCS-MP2^BWI‑DZ^—the performance of both methods remains comparable, which can be a consequence of what our heatmaps in Figure 9 indicated, different calibrations may show quantitatively accurate results if their “optimal” zones (set of coefficients pairs) traslape. The dissociation curve for uracil dimer C_4_H_4_N_2_O_2_–C_4_H_4_N_2_O_2_ (BP, Figure 9c) further supports the robustness of our method. Both MP2 and RIJK-SCS-MP2^BWI‑DZ^ provide accurate descriptions near the equilibrium and at a long-range separation distance up to 0.5 (scaled), successfully recovering the expected interaction energy behavior. In contrast, SCS-MP2 struggles near the equilibrium geometry, deviating from the CCSD­(T)/CBS reference. This behavior underscores the sensitivity of SCS-MP2 to the balance of correlation components in near-equilibrium geometries. For higher distances, all the methods, including canonical MP2, consistently deviates from the reference curve, although the difference is less than 1 kcal/mol, this result indicates that this ill behavior is directly inherited from standard MP2 theory and will be roughly corrected by a simple scaling optimization, unless each point of the dissociation curve owns a particular pair of optimized coefficients. Finally, in the π–π stacked system C_5_H_5_N–C_4_H_4_N_2_O_2_ (Figure 9d), both SCS-MP2 and RIJK-SCS-MP2^BWI‑DZ^ delivered a similar agreement with CCSD­(T)/CBS near the energy minimum and correctly described the infinite dissociation limit behavior. MP2 shows the weakest performance, overestimating binding. These results suggest that enhancing short-range dynamical correlation (as in SCS-MP2 with *C*_OS_ = 1.20) and simultaneously suppressing long-range nondynamical correlation (*C*_SS_ = 0.33) yields the most quantitatively accurate description of π–π interactions near equilibrium. However, at extended distances, all methods converge and exhibit comparable performance, highlighting minimal differences under “infinite″ dissociation conditions for this system.

It is important to emphasize that while the present SCS-MP2 parametrization provides reliable results for the weakly bound biological systems studied, its performance cannot be generalized to systems of fundamentally different nature. For instance, dissociation profiles of covalently bonded species (where spin-symmetry breaking may occur) or dispersion-only interactions such as those present in rare-gas dimers (such as Ar_2_,^122^ where orbital overlap is negligible) may require a more balanced or system-specific treatment of spin correlation components and the description of those phenomena is beyond the scope of the this work.

## 6 Conclusions

Weak interactions play a pivotal role in numerous biological processes, and their quantitatively accurate description continues to represent a significant challenge for electronic structure methods. In this work, we introduced a series of MP2-based spin-component-scaled (SCS-MP2) methodologies aimed at reliably quantifying interaction energies. These approaches were applied within the supermolecular framework and extended to compute two- and four-body interaction energies—particularly relevant for decomposing stacking contributions to the overall interaction energy. To achieve computational efficiency without compromising accuracy, we incorporated several acceleration techniques, including RI approximation and predefined integration grids. These enhancements were employed to evaluate key energy components, namely, the Hartree–Fock term and same- and opposite-spin correlation energies.

Following systematic calibration, two of the developed methodologies demonstrated an outstanding performance. These models consistently outperformed other high-level approaches used for comparison—both in the data sets used for training and in three biologically relevant case studies. Notably, the RIJCOSX-SCS-MP2^BWI‑DZ^ method offers a remarkable balance between accuracy and efficiency, delivering interaction energies close to CCSD­(T)/CBS quality at a computational cost comparable to DFT. For applications requiring even greater accuracy, the RIJCOSX-SCS-MP2^BWI‑TZ^ method stands out. While it entails a higher computational demand, it still operates within the cost range of hybrid or meta-GGA functionals and achieves exceptional accuracy in predicting weak interaction energies.

It is important to emphasize that our calibrated methodologies demonstrated excellent predictive capabilities for systems outside of the training set. In particular, the RIJCOSX-SCS-MP2^BWI‑DZ^ method quantitatively accurately reproduced both two-body and four-body stacking interactions between nucleotide base pairs. These interactions are highly sensitive to spatial rearrangements due to the complex interplay between overlapping π-clouds, yet our method captured these effects with impressive precision. This approach also yielded the best statistical performance in modeling halogen bonding—an interaction type increasingly recognized for its relevance in biological systems, especially in the context of halogenated compounds interacting with protein residues. Despite their significance in drug design, the interaction of halogens with residues has not received in-depth attention; in this context, our method presents a valuable tool for bridging this gap with accurate and efficient modeling. Furthermore, the RIJCOSX-SCS-MP2^BWI‑DZ^ method provided reliable predictions of potential energy dissociation curves, highlighting its applicability for modeling long-range weak interactions commonly encountered in biological assemblies.

Overall, our findings support the recommendation of the RIJCOSX-SCS-MP2^BWI‑DZ^ variant as a highly versatile, accurate, and computationally efficient strategy for studying weak interactions present in biological assemblies. In addition to its performance, this method exhibits a notable extensivity. As shown in Figure 3, a broad range of *C*_OS_ and *C*_SS_ coefficient pairs yield similarly accurate results for a given test set. This overlap suggests the possibility of developing transferable parametrizations applicable across different data sets and molecular systems. Unlike DFAs, which often require extensive parametrization, the SCS-RI-MP2 framework relies on just two parameters. This simplicity, combined with potential extensibility, makes the method particularly well-suited for investigating noncovalent interactions in biologically relevant complexes of medium to large size.

## Supplementary Material



## Notes

The online version contains Supporting Information available at Supporting Information. The file includes the evaluation of mean absolute deviation in a grid of *C*_OS_ and *C*_SS_ values, interaction energies and absolute deviations of molecular systems for the A24, HB3000PX­(S,P), HB3000PX­(Cl,F), JSCH-2005*, S66, and X40* databases. Finally, orca input for RIJK-MP2/aug-cc-PVDZ calculations. The data are presented for aug-cc-pVDZ and aug-cc-pVTZ basis sets (PDF)
