# Supplementary material for: Computationally Efficient Yet Quantitatively Accurate Scaled MP2 Protocols for the Prediction of Weak Interaction Energies in Complex Biological Systems
Source: ACS Omega. 2025 Aug 20;10(34):39292–308. doi: 10.1021/acsomega.5c07079 (PMC12409536; doi:10.1021/acsomega.5c07079)
Supplement: Supplementary file 1 [file ao5c07079_si_001.pdf]

Supporting Information: Computationally efficient yet quantitatively accurate scaled MP2 protocols for the prediction of weak interaction energies in complex biological systems

Luis Soriano-Agueda,*,† Anaid Flores,‡ Paulino Zeron,‡ and Marco Franco Pérez*,†

†*Departamento de Física y Química Teórica, Facultad de Química, Universidad Nacional Autónoma de México, Cd. Universitaria, 04510 Ciudad de Mexico, México.*

‡*Departamento de Química, Universidad Autónoma Metropolitana-Iztapalapa, Av. San Rafael Atlixco 186, Ciudad de México 09340, México.*

E-mail: lsorianoagueda@gmail.com; qimfranco@gmail.com

Contents

1 BSSE contribution **S-5**

1.1 Kohn-Sham orbitals S-5

1.2 MP2 . . . . S-5

2 A24 dataset **S-7**

2.1 aug-cc-pVDZ basis set data. S-7

2.1.1 Ionization Potentials and HOMO values for SAPT-DFT calculations . S-7
2.1.2 Evaluation of mean absolute deviation in a grid of C_{OS} and C_{SS} values S-8
2.1.3 Interaction energies S-9
2.1.4 Absolute deviations of molecular systems. S-12
2.2 aug-cc-pVTZ basis set data. S-15
2.2.1 Ionization Potentials and HOMO values for SAPT-DFT calculations . S-15
2.2.2 Evaluation of mean absolute deviation in a grid of C_{OS} and C_{SS} values S-16
2.2.3 Interaction energies S-17
2.2.4 Absolute deviations of molecular systems. S-20
2.3 Mean calculation times S-23

3 HB300SPX(S,P) S-24

3.1 aug-cc-pVDZ basis set data. S-24
3.1.1 Ionization Potentials and HOMO values for SAPT-DFT calculations . S-24
3.1.2 Evaluation of mean absolute deviation in a grid of C_{OS} and C_{SS} values S-25
3.1.3 Interaction energies S-26
3.1.4 Absolute deviations of molecular systems. S-29
3.2 aug-cc-pVTZ basis set data. S-32
3.2.1 Ionization Potentials and HOMO values for SAPT-DFT calculations . S-32
3.2.2 Evaluation of mean absolute deviation in a grid of C_{OS} and C_{SS} values S-33
3.2.3 Interaction energies S-34
3.2.4 Absolute deviations of molecular systems. S-37
3.3 Mean calculation times S-40

4 HB300SPX(Cl,F) S-41

4.1 aug-cc-pVDZ basis set data. S-41
4.1.1 Ionization Potentials and HOMO values for SAPT-DFT calculations . S-41
4.1.2 Evaluation of mean absolute deviation in a grid of C_{OS} and C_{SS} values S-42

4.1.3 Interaction energies S-43

4.1.4 Absolute deviations of molecular systems. S-46

4.2 aug-cc-pVTZ basis set data. S-49

4.2.1 Ionization Potentials and HOMO values for SAPT-DFT calculations . S-49

4.2.2 Evaluation of mean absolute deviation in a grid of C_{OS} and C_{SS} values S-50

4.2.3 Interaction energies S-51

4.2.4 Absolute deviations of molecular systems. S-54

4.3 Mean calculation times S-57

5 JSCH-2005* dataset S-58

5.1 aug-cc-pVDZ basis set data. S-58

5.1.1 Ionization Potentials and HOMO values for SAPT-DFT calculations . S-58

5.1.2 Evaluation of mean absolute deviation in a grid of C_{OS} and C_{SS} values S-59

5.1.3 Interaction energies S-60

5.1.4 Absolute deviations of molecular systems. S-63

5.2 aug-cc-pVTZ basis set data. S-66

5.2.1 Ionization Potentials and HOMO values for SAPT-DFT calculations . S-66

5.2.2 Evaluation of mean absolute deviation in a grid of C_{OS} and C_{SS} values S-67

5.2.3 Interaction energies S-68

5.2.4 Absolute deviations of molecular systems. S-71

5.3 Mean calculation times S-74

6 S66 dataset S-75

6.1 aug-cc-pVDZ basis set data. S-75

6.1.1 Ionization Potentials and HOMO values for SAPT-DFT calculations . S-75

6.1.2 Evaluation of mean absolute deviation in a grid of C_{OS} and C_{SS} values S-76

6.1.3 Interaction energies S-77

6.1.4 Absolute deviations of molecular systems. S-80

6.2 aug-cc-pVTZ basis set data. S-83

6.2.1 Ionization Potentials and HOMO values for SAPT-DFT calculations . S-83

6.2.2 Evaluation of mean absolute deviation in a grid of C_{OS} and C_{SS} values S-84

6.2.3 Interaction energies S-85

6.2.4 Absolute deviations of molecular systems. S-88

6.3 Mean calculation times S-91

7 X40* dataset S-92

7.1 aug-cc-pVDZ basis set data. S-92

7.1.1 Ionization Potentials and HOMO values for SAPT-DFT calculations . S-92

7.1.2 Evaluation of mean absolute deviation in a grid of C_{OS} and C_{SS} values S-93

7.1.3 Interaction energies S-94

7.1.4 Absolute deviations of molecular systems. S-97

7.2 aug-cc-pVTZ basis set data. S-100

7.2.1 Ionization Potentials and HOMO values for SAPT-DFT calculations . S-100

7.2.2 Evaluation of mean absolute deviation in a grid of C_{OS} and C_{SS} values S-101

7.2.3 Interaction energies S-102

7.2.4 Absolute deviations of molecular systems. S-105

7.3 Mean calculation times S-108

8 Orca input for RIJK-MP2/aug-cc-pVDZ calculations S-109

9 Python script to minimize MAD S-110

1 BSSE contribution

1.1 Kohn-Sham orbitals

Table S1: Deviation metrics obtained for the B97M-V DFA. We have considered the X40* database. All values in kcal/mol.

Deviation	Without BSSE	
MAD	0.30	0.18
$Max(MAD)$	1.21	0.98
$RMSD$	1.29	1.22
	With BSSE	
MAD	0.22	0.26
$Max(MAD)$	0.99	1.04
$RMSD$	1.21	1.20

1.2 MP2

In order to explore the dependence of the optimal parameters (C_{OS} and C_{SS}) by considered the BSSE. We searched for the coefficients that minimize MAD considering the BSSE. The methodologies that emerge from this process are named SCS-MP2$^{BWI-DZ-BSSE}$ and SCS-MP2$^{BWI-TZ-BSSE}$. The optimal SCS-MP2$^{BWI-DZ-BSSE}$ coefficients are C_{OS}=0.1 and C_{SS}=1.9; MAD=0.35 kcal/mol, RMSD=0.55 kcal/mol and Max(MAD)=1.98 kcal/mol. Compared with the SCS-MP2^{BWI-DZ} approach, where the optimal parameters are C_{OS}=0.00 and C_{SS}=1.50, we obtain: MAD=0.36 kcal/mol, RMSD=0.63 kcal/mol, and Max(MAD)=2.21 kcal/mol. For SCS-MP2$^{BWI-TZ-BSSE}$, the optimal parameters are C_{OS}=0.30 and C_{SS}=1.64, with MAD=0.32 kcal/mol, RMSD=0.47 kcal/mol, and Max(MAD)=1.42 kcal/mol. In the case of SCS-MP2^{BWI-TZ}, the optimal coefficient pairs are C_{OS}=0.27 and C_{SS}=1.38; MAD=0.36 kcal/mol, RMSD=0.51 kcal/mol, and Max(MAD)=1.50 kcal/mol. For both sets of bases, we highlight that:

"Both coefficients, C_{OS} and C_{SS}, increase when we consider the BSSE. However, when looking at the deviation metrics, we see that in both cases there are no significant changes, so

minimizing the MAD without considering the BSSE is an appropriate strategy."

The optimal parameters depend on the basis set and the BSSE. However, considering the BSSE does not improve spin scaling performance. Furthermore, we cannot forget that the methodologies proposed in our work are applicable to biological systems, so calculating the BSSE requires a computational effort that does not improve the description of the systems.

2 A24 dataset

2.1 aug-cc-pVDZ basis set data.

2.1.1 Ionization Potentials and HOMO values for SAPT-DFT calculations

Table S2: Experimental ionization potentials (IP) of molecules comprising dimers in the A24 database. IP obtained from NIST (https://webbook.nist.gov/chemistry). Kohn-Sham energies of HOMO orbitals were calculated at the DFA/aug-cc-pVDZ level of theory (DFA=B3LYP, PBE0, and ωB97X). Ionization energies in ev and HOMO energies in atomic units.

			HOMO energies (eV)			$\Delta_{XC} = \epsilon_{HOMO} - (-IP)$		
Molecule	Formula	IP	B3LYP	PBE0	ωB97X	B3LYP	PBE0	ωB97X
ammonia	NH_3	10.07	-7.43	-7.76	-10.37	0.10	0.08	0.01
argon	Ar	15.76	-11.58	-12.00	-14.58	0.15	0.14	0.04
borane	BH_3	12.03	-9.64	-9.91	-12.49	0.09	0.08	0.02
ethane	C_2H_6	11.52	-9.30	-9.58	-12.09	0.08	0.07	0.02
ethene	C_2H_4	10.51	-7.54	-7.86	-10.29	0.11	0.10	0.01
ethyne	C_2H_2	11.40	-8.05	-8.39	-10.86	0.12	0.11	0.02
formaldehyde	CH_2O	10.88	-7.54	-7.85	-10.33	0.12	0.11	0.02
hydrocyanicacid	HCN	13.60	-9.97	-10.32	-12.87	0.13	0.12	0.03
hydrogenfluoride	HF	16.03	-11.45	-11.85	-14.47	0.17	0.15	0.06
methane	CH_4	12.61	-10.67	-10.98	-13.57	0.07	0.06	0.04
water	H_2O	12.62	-8.72	-9.07	-11.69	0.14	0.13	0.03

2.1.2 Evaluation of mean absolute deviation in a grid of C_{OS} and C_{SS} values

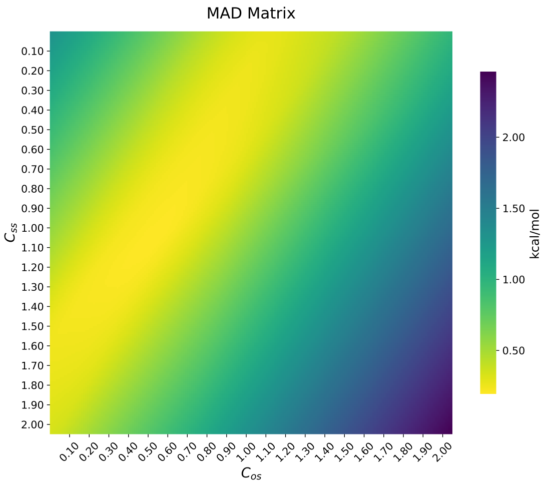

(a)

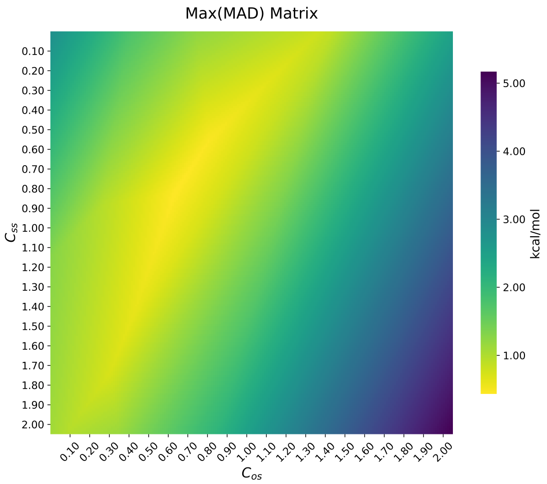

(b)

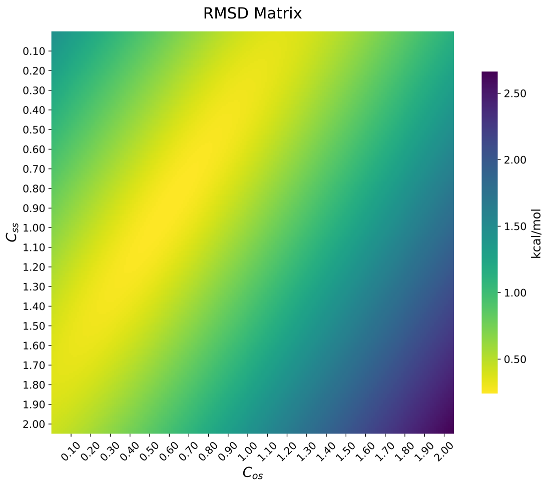

(c)

Figure S1: Evaluation of MADs (S1a), Max(MADs) (S1b) and RMSDs (S1c) in grid of values C_{OS} and C_{SS}. The optimal coefficients are C_{OS}=0.00 and C_{SS}=1.50. The theory level used is RIJK-MP2/aug-cc-pVDZ.

2.1.3 Interaction energies

MP2 with C_{OS} y C_{SS} values

Table S3: Interaction energies for CCSD(T)/CBS, MP2, SCS-MP2, SCS(MI)-MP2, SCSN-MP2, SCS-MP2-vdW, RI-MP2, RI-SCS-MP2, RIJK-MP2, RIJK-SCS-MP2, RIJCOSX-MP2, RIJCOSX-SCS-MP2, SCS-MP2^{BWI-DZ}, RI-SCS-MP2^{BWI-DZ}, RIJK-SCS-MP2^{BWI-DZ}, RIJCOSX-SCS-MP2^{BWI-DZ} and SCS-MP2-hal^{G-XZ}. Data: 24 interaction energies. MP2: $C_{OS} = C_{SS}$ =1.00; SCS-MP2: C_{OS} =1.20, C_{SS} =0.33; SCS(MI)-MP2: C_{OS} =0.40, C_{SS} =1.29; SCSN-MP2: C_{OS} =0.00, C_{SS} =1.76; SCS-MP2-vdW: C_{OS} =1.28, C_{SS} =0.50; SCS-MP2^{BWI-DZ} RI-SCS-MP2^{BWI-DZ} , RIJK-SCS-MP2^{BWI-DZ} : C_{OS} =0.00, C_{SS} =1.50; RIJCOSX-SCS-MP2^{BWI-DZ} : C_{OS} =0.00, C_{SS} =0.17. All values in kcal/mol.

Dimer	Formula	CCSD(T)/CBS	MP2	SCS-MP2	SCS(MI)-MP2	SCSN-MP2	SCS-MP2-vdW	RI-MP2	RI-SCS-MP2	RIJK-MP2	RIJK-SCS-MP2	RIJCOSX-MP2	RIJCOSX-SCS-MP2	SCS-MP2^{BWI-DZ}	RI-SCS-MP2^{BWI-DZ}	RIJK-SCS-MP2^{BWI-DZ}	RIJCOSX-SCS-MP2^{BWI-DZ}	SCS-MP2-hal^{G-XZ}
ammonia − ammonia	NH_3-NH_3	-3.14	-3.51	-3.15	-3.17	-3.18	-3.37	-3.51	-3.15	-3.51	-3.14	-3.51	-3.14	-2.96	-2.96	-2.95	-2.95	-2.96
ammonia − methane	NH_3-CH_4	-0.77	-1.32	-1.17	-1.05	-0.98	-1.30	-1.32	-1.17	-1.32	-1.17	-1.31	-1.16	-0.87	-0.87	-0.87	-0.87	-0.97
borane − methane	BH_3-CH_4	-1.49	-1.88	-1.41	-1.24	-1.15	-1.74	-1.88	-1.42	-1.87	-1.41	-1.89	-1.42	-0.84	-0.84	-0.83	-0.86	-0.99
ethene − ammonia	$C_2H_4-NH_3$	-1.37	-2.28	-1.99	-1.75	-1.63	-2.23	-2.28	-1.99	-2.28	-1.99	-2.28	-1.99	-1.42	-1.42	-1.41	-1.42	-1.61
ethene − argon	C_2H_4-Ar	-0.36	-0.61	-0.49	-0.33	-0.25	-0.60	-0.61	-0.49	-0.60	-0.48	-0.62	-0.50	-0.15	-0.15	-0.15	-0.16	-0.28
ethene − ethene	$C_2H_4-C_2H_4$	-1.09	-2.30	-1.92	-1.50	-1.28	-2.26	-2.30	-1.92	-2.30	-1.92	-2.31	-1.92	-0.98	-0.99	-0.98	-1.00	-1.32
ethene − ethene	$C_2H_4-C_2H_4$	0.93	-0.18	0.18	0.98	1.40	-0.25	-0.19	0.18	-0.18	0.19	-0.19	0.19	1.74	1.73	1.74	1.72	1.11
ethene − ethyne	$C_2H_4-C_2H_2$	0.82	0.15	0.48	0.95	1.20	0.15	0.14	0.48	0.16	0.50	0.14	0.48	1.47	1.46	1.48	1.45	1.10
ethene − formaldehyde	$C_2H_4-CH_2O$	-1.62	-2.32	-1.97	-1.91	-1.86	-2.20	-2.32	-1.97	-2.31	-1.96	-2.31	-1.96	-1.64	-1.65	-1.64	-1.65	-1.71
ethene − water	$C_2H_4-H_2O$	-2.56	-3.42	-3.00	-2.89	-2.82	-3.29	-3.42	-3.00	-3.42	-3.00	-3.39	-2.98	-2.55	-2.55	-2.55	-2.54	-2.66
ethyne − ethyne	$C_2H_2-C_2H_2$	-1.52	-2.09	-1.87	-1.93	-1.95	-1.99	-2.09	-1.87	-2.09	-1.87	-2.09	-1.86	-1.83	-1.83	-1.83	-1.83	-1.79
ethyne − ethyne	$C_2H_2-C_2H_2$	1.12	0.80	1.11	1.32	1.44	0.87	0.80	1.11	0.83	1.13	0.80	1.10	1.66	1.65	1.68	1.64	1.48
formaldehyde − formaldehyde	CH_2O	-4.55	-4.81	-4.09	-4.79	-5.12	-4.36	-4.82	-4.09	-4.80	-4.07	-4.81	-4.09	-4.79	-4.79	-4.78	-4.80	-4.29
hydrogencyanide − hydrogencyanide	$HCN-HCN$	-4.75	-4.59	-4.44	-5.41	-5.90	-4.28	-4.59	-4.44	-4.59	-4.44	-4.59	-4.44	-5.96	-5.95	-5.95	-5.95	-5.22
hydrogenfluoride − hydrogenfluoride	$HF-HF$	-4.58	-4.66	-4.38	-4.60	-4.71	-4.50	-4.66	-4.38	-4.65	-4.37	-4.64	-4.36	-4.58	-4.58	-4.57	-4.56	-4.42
methane − argon	CH_4-Ar	-0.41	-0.66	-0.55	-0.42	-0.35	-0.65	-0.66	-0.55	-0.65	-0.54	-0.66	-0.55	-0.27	-0.27	-0.26	-0.27	-0.37
methane − ethane	$CH_4-C_2H_6$	-0.83	-1.39	-1.25	-0.89	-0.70	-1.43	-1.39	-1.25	-1.39	-1.24	-1.40	-1.25	-0.56	-0.56	-0.56	-0.57	-0.84
methane − ethane	$CH_4-C_2H_6$	-0.61	-1.68	-1.45	-1.09	-0.90	-1.68	-1.68	-1.45	-1.68	-1.44	-1.67	-1.44	-0.70	-0.70	-0.70	-0.70	-0.99
methane − ethene	$CH_4-C_2H_4$	-0.50	-1.28	-1.15	-0.84	-0.67	-1.31	-1.28	-1.15	-1.28	-1.14	-1.29	-1.15	-0.54	-0.55	-0.54	-0.56	-0.79
methane − hydrogenfluoride	CH_4-HF	-1.65	-1.97	-1.68	-1.63	-1.59	-1.88	-1.97	-1.68	-1.97	-1.68	-1.96	-1.67	-1.41	-1.41	-1.41	-1.40	-1.47
methane − methane	CH_4-CH_4	-0.53	-1.17	-1.03	-0.76	-0.62	-1.18	-1.17	-1.03	-1.16	-1.03	-1.16	-1.03	-0.50	-0.50	-0.49	-0.50	-0.71
methane − water	CH_4-H_2O	-0.66	-1.02	-0.90	-0.83	-0.80	-0.99	-1.02	-0.90	-1.03	-0.90	-1.02	-0.90	-0.71	-0.71	-0.72	-0.71	-0.77
water − ammonia	H_2O-NH_3	-6.49	-7.00	-6.44	-6.65	-6.74	-6.73	-7.00	-6.45	-7.00	-6.44	-6.96	-6.41	-6.44	-6.43	-6.43	-6.41	-6.31
water − water	H_2O-H_2O	-5.01	-5.25	-4.86	-5.10	-5.22	-5.04	-5.25	-4.86	-5.25	-4.86	-5.23	-4.84	-5.02	-5.01	-5.01	-4.99	-4.85

DFT

Table S4: Interaction energies for CCSD(T)/CBS, B97M-V, ωB97X-V, ωB97M-V, ωB97X-D3, B2PLYP-D3BJ, DSD-BLYP-D3BJ, ωB97X-D4 and B2PLYP-D4 . Data: 24 interaction energies. All values in kcal/mol.

Dimer	Formula	CCSD(T)/CBS	B97M-V	ωB97X-V	ωB97M-V	ωB97X-D3	B2PLYP-D3BJ	DSD-BLYP-D3BJ	ωB97X-D4	B2PLYP-D4
ammonia − ammonia	NH_3−NH_3	-3.14	-3.17	-3.37	-3.40	-3.37	-3.46	-3.45	-3.93	-3.37
ammonia − methane	NH_3−CH_4	-0.77	-1.03	-1.19	-1.09	-1.18	-1.16	-1.17	-1.47	-1.16
borane − methane	BH_3−CH_4	-1.49	-2.24	-1.93	-1.99	-2.03	-1.83	-1.79	-2.79	-1.73
ethene − ammonia	C_2H_4−NH_3	-1.37	-1.51	-1.73	-1.70	-1.85	-1.87	-1.92	-2.06	-1.82
ethene − argon	C_2H_4−Ar	-0.36	-0.44	-0.57	-0.54	-0.59	-0.45	-0.45	-0.70	-0.40
ethene − ethene	C_2H_4−C_2H_4	-1.09	-1.38	-1.54	-1.51	-1.68	-1.70	-1.75	-2.04	-1.65
ethene − ethene	C_2H_4−C_2H_4	0.93	0.83	0.58	0.47	0.25	0.56	0.49	0.02	0.59
ethene − ethyne	C_2H_4−C_2H_2	0.82	0.76	0.57	0.46	0.31	0.68	0.63	0.00	0.71
ethene − formaldehyde	C_2H_4−CH_2O	-1.62	-1.86	-2.08	-2.04	-2.14	-2.03	-2.06	-2.54	-1.96
ethene − water	C_2H_4−H_2O	-2.56	-2.75	-2.96	-2.96	-3.03	-3.08	-3.11	-3.35	-2.98
ethyne − ethyne	C_2H_2−C_2H_2	-1.52	-1.85	-1.98	-2.01	-2.02	-1.91	-1.93	-2.35	-1.87
ethyne − ethyne	C_2H_2−C_2H_2	1.12	1.09	0.95	0.84	0.76	1.15	1.11	0.38	1.16
formaldehyde − formaldehyde	CH_2O−CH_2O	-4.55	-5.38	-4.99	-5.10	-4.87	-4.96	-4.93	-5.74	-4.90
hydrogencyanide − hydrogencyanide	HCN−HCN	-4.75	-5.28	-5.66	-5.73	-5.75	-5.22	-5.15	-6.27	-5.18
hydrogenfluoride − hydrogenfluoride	HF−HF	-4.58	-4.97	-4.77	-4.79	-4.57	-4.89	-4.85	-5.26	-4.85
methane − argon	CH_4−Ar	-0.41	-0.49	-0.63	-0.59	-0.64	-0.52	-0.54	-0.76	-0.46
methane − ethane	CH_4−C_2H_6	-0.83	-0.83	-1.16	-1.08	-1.23	-1.11	-1.12	-1.44	-1.07
methane − ethane	CH_4−C_2H_6	-0.61	-1.10	-1.43	-1.34	-1.52	-1.38	-1.39	-1.78	-1.33
methane − ethene	CH_4−C_2H_4	-0.50	-0.71	-0.87	-0.79	-0.93	-0.91	-0.96	-1.08	-0.90
methane − hydrogenfluoride	CH_4−HF	-1.65	-1.97	-1.97	-1.94	-1.84	-1.97	-1.93	-2.32	-1.89
methane − methane	CH_4−CH_4	-0.53	-0.77	-1.04	-0.98	-1.13	-0.97	-0.97	-1.32	-0.93
methane − water	CH_4−H_2O	-0.66	-0.79	-0.92	-0.83	-0.82	-0.87	-0.90	-1.07	-0.88
water − ammonia	H_2O−NH_3	-6.49	-6.53	-7.01	-6.97	-7.22	-7.15	-7.05	-7.68	-7.08
water − water	H_2O−H_2O	-5.01	-5.09	-5.23	-5.24	-5.26	-5.29	-5.27	-5.84	-5.27

SAPT-DFT

Table S5: Interaction energies for CCSD(T)/CBS and SAPT-DFT. For SAPT-DFT method we used the B3LYP, PBE0 and ωB97X DFA's in conjunction with the aug-cc-pVDZ basis set. Data: 24 interaction energies. All values in kcal/mol.

Dimer	Formula	CCSD(T)/CBS	SAPT-B3LYP	SAPT-PBE0	SAPT-ωB97X
ammonia − ammonia	NH_3−NH_3	-3.14	-2.20	-2.29	-2.09
ammonia − methane	NH_3−CH_4	-0.77	-0.48	-0.51	-0.44
borane − methane	BH_3−CH_4	-1.49	-0.21	-0.23	-0.19
ethene − ammonia	C_2H_4−NH_3	-1.37	-1.02	-1.07	-0.87
ethene − argon	C_2H_4−Ar	-0.36	-0.16	-0.15	-0.04
ethene − ethene	C_2H_4−C_2H_4	-1.09	-0.80	-0.78	-0.42
ethene − ethene	C_2H_4−C_2H_4	0.93	1.25	1.31	1.84
ethene − ethyne	C_2H_4−C_2H_2	0.82	1.15	1.21	1.67
ethene − formaldehyde	C_2H_4−CH_2O	-1.62	-1.22	-1.25	-0.99
ethene − water	C_2H_4−H_2O	-2.56	-1.93	-2.06	-1.80
ethyne − ethyne	C_2H_2−C_2H_2	-1.52	-1.28	-1.33	-1.14
ethyne − ethyne	C_2H_2−C_2H_2	1.12	1.47	1.54	1.96
formaldehyde − formaldehyde	CH_2O−CH_2O	-4.55	-3.17	-3.24	-2.94
hydrocyanicacid − hydrocyanicacid	HCN−HCN	-4.75	-4.23	-4.25	-4.17
hydrogenfluoride − hydrogenfluoride	HF−HF	-4.58	-3.47	-3.59	-3.48
methane − argon	CH_4−Ar	-0.41	-0.17	-0.16	-0.09
methane − ethane	CH_4−C_2H_6	-0.61	-0.31	-0.28	-0.23
methane − ethane	CH_4−C_2H_6	-0.50	-0.42	-0.39	-0.29
methane − ethene	CH_4−C_2H_4	-0.50	-0.35	-0.36	-0.29
methane − hydrogenfluoride	CH_4−HF	-1.65	-0.78	-0.93	-0.90
methane − methane	CH_4−CH_4	-0.53	-0.25	-0.22	-0.18
methane − water	CH_4−H_2O	-0.66	-0.43	-0.45	-0.39
water − ammonia	H_2O−NH_3	-6.49	-4.92	-5.18	-4.89
water − water	H_2O−H_2O	-5.01	-3.83	-3.99	-3.79

2.1.4 Absolute deviations of molecular systems.

MP2 with C_{OS} y C_{SS} values

Table S6: Absolute deviations for MP2, SCS-MP2, SCS(MI)-MP2, SCSN-MP2, SCS-MP2-vdW, RI-MP2, RI-SCS-MP2, RIJK-MP2, RIJK-SCS-MP2, RIJCOSX-MP2, RIJCOSX-SCS-MP2, SCS-MP2^{BWI-DZ}, RI-SCS-MP2^{BWI-DZ}, RIJK-SCS-MP2^{BWI-DZ}, RIJCOSX-SCS-MP2^{BWI-DZ} and SCS-MP2-hal^{G-XZ}. Data: 24 interaction energies. MP2: $C_{OS} = C_{SS}$ =1.00; SCS-MP2: C_{OS} =1.20, C_{SS} =0.33; SCS(MI)-MP2: C_{OS} =0.40, C_{SS} =1.29; SCSN-MP2: C_{OS} =0.00, C_{SS} =1.76; SCS-MP2-vdW: C_{OS} =1.28, C_{SS} =0.50; SCS-MP2^{BWI-DZ} RI-SCS-MP2^{BWI-DZ} , RIJK-SCS-MP2^{BWI-DZ} : C_{OS} =0.00, C_{SS} =1.50; RIJCOSX-SCS-MP2^{BWI-DZ} : C_{OS} =0.00, C_{SS} =0.17. All values in kcal/mol.

Dimer	Formula	MP2	SCS-MP2	SCS(MI)-MP2	SCSN-MP2	SCS-MP2-vdW	RI-MP2	RI-SCS-MP2	RIJK-MP2	RIJK-SCS-MP2	RIJCOSX-MP2	RIJCOSX-SCS-MP2	SCS-MP2^{BWI-DZ}	RI-SCS-MP2^{BWI-DZ}	RIJK-SCS-MP2^{BWI-DZ}	RIJCOSX-SCS-MP2^{BWI-DZ}	SCS-MP2-hal^{G-XZ}
ammonia − ammonia	NH_3	0.37	0.01	0.03	0.04	0.23	0.37	0.01	0.37	0.00	0.37	0.00	0.18	0.18	0.19	0.19	0.18
ammonia − methane	NH_3	0.55	0.40	0.28	0.21	0.53	0.55	0.40	0.55	0.40	0.54	0.39	0.10	0.10	0.10	0.10	0.20
borane − methane	BH_3	0.39	0.08	0.25	0.34	0.25	0.39	0.07	0.38	0.08	0.40	0.07	0.65	0.65	0.66	0.63	0.50
ethene − ammonia	C_2H_4	0.91	0.62	0.38	0.26	0.86	0.91	0.62	0.91	0.62	0.91	0.62	0.05	0.05	0.04	0.05	0.24
ethene − argon	C_2H_4	0.25	0.13	0.03	0.11	0.24	0.25	0.13	0.24	0.12	0.26	0.14	0.21	0.21	0.21	0.20	0.08
ethene − ethene	C_2H_4	1.21	0.83	0.41	0.19	1.17	1.21	0.83	1.21	0.83	1.22	0.83	0.11	0.10	0.11	0.09	0.23
ethene − ethene	C_2H_4	1.11	0.75	0.05	0.47	1.18	1.12	0.75	1.11	0.74	1.12	0.74	0.81	0.80	0.81	0.79	0.18
ethene − ethyne	C_2H_4	0.67	0.34	0.13	0.38	0.67	0.68	0.34	0.66	0.32	0.68	0.34	0.65	0.64	0.66	0.63	0.28
ethene − formaldehyde	C_2H_4	0.70	0.35	0.29	0.24	0.58	0.70	0.35	0.69	0.34	0.69	0.34	0.02	0.03	0.02	0.03	0.09
ethene − water	C_2H_4	0.86	0.44	0.33	0.26	0.73	0.86	0.44	0.86	0.44	0.83	0.42	0.01	0.01	0.01	0.02	0.10
ethyne − ethyne	C_2H_2	0.57	0.35	0.41	0.43	0.47	0.57	0.35	0.57	0.35	0.57	0.34	0.31	0.31	0.31	0.31	0.27
ethyne − ethyne	C_2H_2	0.32	0.01	0.20	0.32	0.25	0.32	0.01	0.29	0.01	0.32	0.02	0.54	0.53	0.56	0.52	0.36
formaldehyde − formaldehyde	CH_2O	0.26	0.46	0.24	0.57	0.19	0.27	0.46	0.25	0.48	0.26	0.46	0.24	0.24	0.23	0.25	0.26
hydrocyanicacid − hydrocyanicacid	HCN	0.16	0.31	0.66	1.15	0.47	0.16	0.31	0.16	0.31	0.16	0.31	1.21	1.20	1.20	1.20	0.47
hydrogenfluoride − hydrogenfluoride	HF	0.08	0.20	0.02	0.13	0.08	0.08	0.20	0.07	0.21	0.06	0.22	0.00	0.00	0.01	0.02	0.16
methane − argon	CH_4	0.25	0.14	0.01	0.06	0.24	0.25	0.14	0.24	0.13	0.25	0.14	0.14	0.14	0.15	0.14	0.04
methane − ethane	CH_4	0.56	0.42	0.06	0.13	0.60	0.56	0.42	0.56	0.41	0.57	0.42	0.27	0.27	0.27	0.26	0.01
methane − ethane	CH_4	1.07	0.84	0.48	0.29	1.07	1.07	0.84	1.07	0.83	1.06	0.83	0.09	0.09	0.09	0.09	0.38
methane − ethene	CH_4	0.78	0.65	0.34	0.17	0.81	0.78	0.65	0.78	0.64	0.79	0.65	0.04	0.05	0.04	0.06	0.29
methane − hydrogenfluoride	CH_4	0.32	0.03	0.02	0.06	0.23	0.32	0.03	0.32	0.03	0.31	0.02	0.24	0.24	0.24	0.25	0.18
methane − methane	CH_4	0.64	0.50	0.23	0.09	0.65	0.64	0.50	0.63	0.50	0.63	0.50	0.03	0.03	0.04	0.03	0.18
methane − water	CH_4	0.36	0.24	0.17	0.14	0.33	0.36	0.24	0.37	0.24	0.36	0.24	0.05	0.05	0.06	0.05	0.11
water − ammonia	H_2O	0.51	0.05	0.16	0.25	0.24	0.51	0.04	0.51	0.05	0.47	0.08	0.05	0.06	0.06	0.08	0.18
water − water	H_2O	0.24	0.15	0.09	0.21	0.03	0.24	0.15	0.24	0.15	0.22	0.17	0.01	0.00	0.00	0.02	0.16

DFT

Table S7: Absolute deviations for B97M-V, ωB97X-V, ωB97M-V, ωB97X-D3, B2PLYP-D3BJ, DSD-BLYP-D3BJ, ωB97X-D4 and B2PLYP-D4. Data: 24 interaction energies. All values in kcal/mol.

Dimer	Formula	B97M-V	ωB97X-V	ωB97M-V	ωB97X-D3	B2PLYP-D3BJ	DSD-BLYP-D3BJ	ωB97X-D4	B2PLYP-D4
ammonia − ammonia	NH_3−NH_3	0.03	0.24	0.26	0.23	0.32	0.31	0.79	0.24
ammonia − methane	NH_3−CH_4	0.27	0.42	0.33	0.42	0.40	0.41	0.71	0.39
borane − methane	BH_3−CH_4	0.75	0.45	0.50	0.54	0.34	0.30	1.31	0.25
ethene − ammonia	C_2H_4−NH_3	0.13	0.35	0.33	0.47	0.50	0.54	0.68	0.44
ethene − argon	C_2H_4−Ar	0.08	0.21	0.18	0.22	0.09	0.09	0.33	0.04
ethene − ethene	C_2H_4−C_2H_4	0.29	0.45	0.42	0.59	0.61	0.66	0.95	0.56
ethene − ethene	C_2H_4−C_2H_4	0.10	0.35	0.46	0.68	0.37	0.45	0.91	0.35
ethene − ethyne	C_2H_4−C_2H_2	0.06	0.25	0.36	0.51	0.14	0.20	0.82	0.11
ethene − formaldehyde	C_2H_4−CH_2O	0.24	0.45	0.42	0.51	0.41	0.44	0.91	0.34
ethene − water	C_2H_4−H_2O	0.19	0.40	0.40	0.47	0.53	0.55	0.79	0.43
ethyne − ethyne	C_2H_2−C_2H_2	0.32	0.45	0.49	0.50	0.39	0.41	0.82	0.35
ethyne − ethyne	C_2H_2−C_2H_2	0.03	0.17	0.28	0.36	0.03	0.00	0.74	0.05
formaldehyde − formaldehyde	CH_2O−CH_2O	0.82	0.44	0.54	0.31	0.41	0.38	1.19	0.35
hydrogencyanide − hydrogencyanide	HCN−HCN	0.54	0.92	0.98	1.01	0.48	0.41	1.53	0.43
hydrogenfluoride − hydrogenfluoride	HF−HF	0.39	0.19	0.21	0.01	0.31	0.26	0.68	0.27
methane − Ar	CH_4−Ar	0.08	0.22	0.18	0.23	0.12	0.13	0.35	0.06
methane − ethane	CH_4−C_2H_6	0.01	0.33	0.25	0.41	0.28	0.30	0.62	0.24
methane − ethane	CH_4−C_2H_6	0.49	0.82	0.73	0.91	0.77	0.78	1.17	0.73
methane − ethene	CH_4−C_2H_4	0.21	0.37	0.29	0.43	0.41	0.46	0.58	0.40
methane − hydrogenfluoride	CH_4−HF	0.32	0.31	0.29	0.18	0.31	0.27	0.67	0.24
methane − methane	CH_4−CH_4	0.23	0.51	0.45	0.60	0.43	0.44	0.78	0.39
methane − water	CH_4−H_2O	0.12	0.25	0.17	0.16	0.21	0.23	0.41	0.21
water − ammonia	H_2O−NH_3	0.04	0.52	0.48	0.72	0.66	0.55	1.19	0.58
water − water	H_2O−H_2O	0.09	0.23	0.24	0.26	0.28	0.27	0.84	0.26

SAPT-DFT

Table S8: Absolute deviations for SAPT-DFT with respect to CCSD(T)/CBS. In SAPT-DFT method we used the B3LYP, PBE0 and ωB97X DFA's in conjunction with the aug-cc-pVDZ basis set. Data: 24 interaction energies. All values in kcal/mol.

Dimer	Formula	SAPT-B3LYP	SAPT-PBE0	SAPT-ωB97X
ammonia − ammonia	NH_3−NH_3	0.94	3.23	5.32
ammonia − methane	NH_3−CH_4	0.28	0.79	1.23
borane − methane	BH_3−CH_4	1.27	1.50	1.69
ethene − ammonia	C_2H_4−NH_3	0.36	1.43	2.29
ethene − Ar	C_2H_4−Ar	0.20	0.36	0.39
ethene − ethene	C_2H_4−C_2H_4	0.29	1.07	1.50
ethene − ethene	C_2H_4−C_2H_4	0.31	1.00	0.84
ethene − ethyne	C_2H_4−C_2H_2	0.33	0.88	0.79
ethene − formaldehyde	C_2H_4−CH_2O	0.40	1.65	2.64
ethene − wat	C_2H_4−H_2O	0.63	2.69	4.49
ethyne − ethyne	C_2H_2−C_2H_2	0.25	1.58	2.72
ethyne − ethyne	C_2H_2−C_2H_2	0.35	1.19	0.77
formaldehyde − formaldehyde	CH_2O−CH_2O	1.38	4.62	7.56
hydrocyanicacid − hydrocyanicacid	HCN−HCN	0.51	4.76	8.93
hydrogenfluoride − hydrogenfluoride	HF−HF	1.11	4.70	8.19
methane − Ar	CH_4−Ar	0.24	0.39	0.49
methane − ethane	CH_4−C_2H_6	0.29	0.58	0.80
methane − ethane	CH_4−C_2H_6	0.08	0.47	0.76
methane − ethene	CH_4−C_2H_4	0.15	0.51	0.79
methane − hydrogenfluoride	CH_4−HF	0.87	1.80	2.69
methane − methane	CH_4−CH_4	0.29	0.50	0.69
methane − water	CH_4−H_2O	0.23	0.69	1.08
water − ammonia	H_2O−NH_3	1.57	6.75	11.64
water − water	H_2O−H_2O	1.17	5.17	8.95

2.2 aug-cc-pVTZ basis set data.

2.2.1 Ionization Potentials and HOMO values for SAPT-DFT calculations

Table S9: Experimental ionization potentials (IP) of molecules comprising dimers in the A24 database. IP obtained from NIST (https://webbook.nist.gov/chemistry). Kohn-Sham energies of HOMO orbitals were calculated at the DFA/aug-cc-pVTZ level of theory (DFA=B3LYP, PBE0, and ωB97X). Ionization energies in ev and HOMO energies in atomic units.

			HOMO energies (eV)			$\Delta_{XC} = \epsilon_{HOMO} - (-IP)$		
		IP	B3LYP	PBE0	ωB97X	B3LYP	PBE0	ωB97X
ammonia	NH_3	10.07	-7.45	-7.78	-10.36	0.10	0.08	0.01
argon	Ar	15.76	-11.59	-11.99	-14.56	0.15	0.14	0.04
borane	BH_3	12.03	-9.66	-9.94	-12.53	0.09	0.08	0.02
ethane	C_2H_6	11.52	-9.32	-9.59	-12.10	0.08	0.07	0.02
ethene	C_2H_4	10.51	-7.56	-7.89	-10.30	0.11	0.10	0.01
ethyne	C_2H_2	11.40	-8.07	-8.40	-10.85	0.12	0.11	0.02
formaldehyde	CH_2O	10.88	-7.58	-7.88	-10.35	0.12	0.11	0.02
hydrogencyanide	HCN	13.60	-9.98	-10.33	-12.85	0.13	0.12	0.03
hydrogenfluoride	HF	16.03	-11.46	-11.84	-14.45	0.17	0.15	0.06
methane	CH_4	12.61	-10.69	-11.00	-13.58	0.07	0.06	0.04
water	H_2O	12.62	-8.74	-9.08	-11.70	0.14	0.13	0.03

2.2.2 Evaluation of mean absolute deviation in a grid of C_{OS} and C_{SS} values

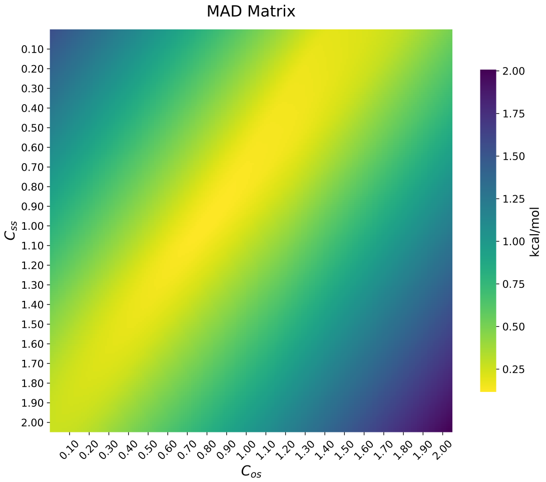

(a)

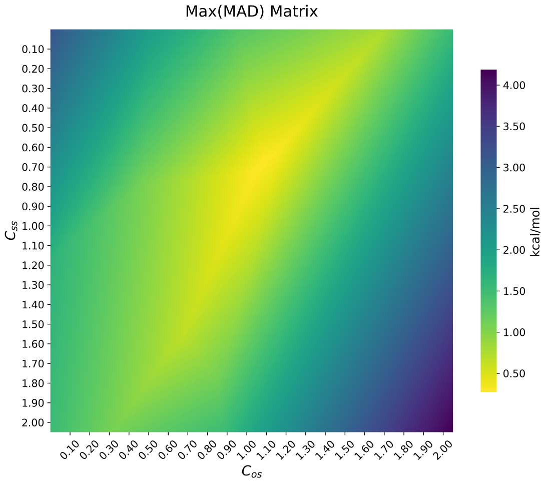

(b)

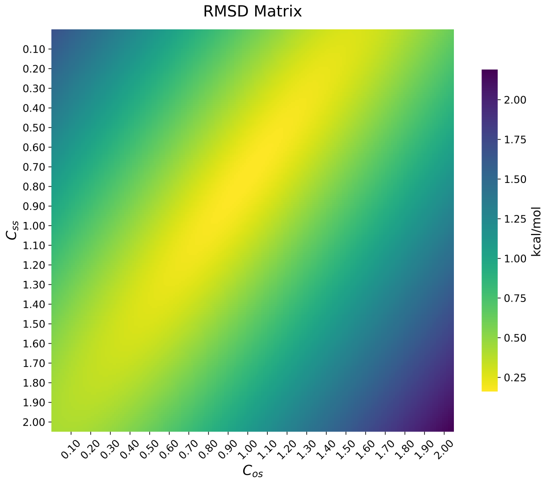

(c)

Figure S2: Evaluation of MADs (S2a), Max(MADs) (S2b) and RMSDs (S2c) in grid of values C_{OS} and C_{SS}. The optimal coefficients are C_{OS}=0.27 and C_{SS}=1.38. The theory level used is RIJK-MP2/aug-cc-pVDZ.

2.2.3 Interaction energies

MP2 with C_{OS} y C_{SS} values

Table S10: Interaction energies for CCSD(T)/CBS, MP2, SCS-MP2, SCS(MI)-MP2, SCSN-MP2, SCS-MP2-vdW, RI-MP2, RI-SCS-MP2, RIJK-MP2, RIJK-SCS-MP2, RIJCOSX-MP2, RIJCOSX-SCS-MP2, SCS-MP2^{BWI-TZ}, RI-SCS-MP2^{BWI-TZ}, RIJK-SCS-MP2^{BWI-TZ}, RIJCOSX-SCS-MP2^{BWI-DZ} and SCS-MP2-hal^{G-XZ}. Data: 24 interaction energies. MP2: $C_{OS} = C_{SS}$ =1.00; SCS-MP2: C_{OS} =1.20, C_{SS} =0.33; SCS(MI)-MP2: C_{OS} =0.40, C_{SS} =1.29; SCSN-MP2: C_{OS} =0.00, C_{SS} =1.76; SCS-MP2-vdW: C_{OS} =1.28, C_{SS} =0.50; SCS-MP2^{BWI-TZ} RI-SCS-MP2^{BWI-TZ} , RIJK-SCS-MP2^{BWI-TZ} : C_{OS} =0.27, C_{SS} =1.38; RIJCOSX-SCS-MP2^{BWI-TZ} : C_{OS} =0.17, C_{SS} =1.59. All values in kcal/mol.

Dimer	Formula	CCSD(T)/CBS	MP2	SCS-MP2	SCS(MI)-MP2	SCSN-MP2	SCS-MP2-vdW	RI-MP2	RI-SCS-MP2	RIJK-MP2	RIJK-SCS-MP2	RIJCOSX-MP2	RIJCOSX-SCS-MP2	SCS-MP2^{BWI-TZ}	RI-SCS-MP2^{BWI-TZ}	RIJK-SCS-MP2^{BWI-TZ}	RIJCOSX-SCS-MP2^{BWI-TZ}	SCS-MP2-hal^{G-XZ}
ammonia − ammonia	NH_3	-3.14	-3.29	-2.90	-2.96	-2.99	-3.13	-3.29	-2.90	-3.29	-2.90	-3.27	-2.88	-2.92	-2.92	-2.91	-2.91	-2.79
ammonia − methane	NH_3	-0.77	-0.82	-0.66	-0.63	-0.61	-0.76	-0.82	-0.66	-0.81	-0.65	-0.80	-0.64	-0.60	-0.60	-0.60	-0.59	-0.61
borane − methane	BH_3	-1.49	-1.51	-0.98	-0.95	-0.92	-1.31	-1.51	-0.98	-1.50	-0.97	-1.51	-0.97	-0.86	-0.86	-0.85	-0.87	-0.81
ethene − ammonia	C_2H_4	-1.37	-1.77	-1.47	-1.35	-1.29	-1.69	-1.77	-1.47	-1.77	-1.47	-1.76	-1.45	-1.28	-1.28	-1.28	-1.27	-1.37
ethene − argon	C_2H_4	-0.36	-0.51	-0.37	-0.24	-0.17	-0.49	-0.51	-0.37	-0.50	-0.36	-0.50	-0.36	-0.20	-0.20	-0.18	-0.18	-0.32
ethene − ethene	C_2H_4	-1.09	-1.52	-1.11	-0.88	-0.76	-1.42	-1.52	-1.11	-1.52	-1.11	-1.48	-1.07	-0.78	-0.78	-0.77	-0.75	-0.97
ethene − ethene	C_2H_4	0.93	0.48	0.89	1.49	1.80	0.49	0.48	0.89	0.49	0.90	0.52	0.93	1.67	1.67	1.68	1.69	1.06
ethene − ethyne	C_2H_4	0.82	0.37	0.77	1.08	1.26	0.44	0.37	0.77	0.39	0.78	0.39	0.78	1.21	1.21	1.22	1.21	0.91
ethene − formaldehyde	C_2H_4	-1.62	-1.88	-1.49	-1.55	-1.56	-1.72	-1.88	-1.49	-1.87	-1.49	-1.86	-1.48	-1.50	-1.50	-1.49	-1.49	-1.38
ethene − water	C_2H_4	-2.56	-3.07	-2.63	-2.60	-2.58	-2.91	-3.07	-2.63	-3.07	-2.63	-3.05	-2.62	-2.53	-2.53	-2.53	-2.52	-2.50
ethyne − ethyne	C_2H_2	-1.52	-1.87	-1.61	-1.82	-1.92	-1.71	-1.87	-1.61	-1.87	-1.61	-1.86	-1.60	-1.82	-1.82	-1.82	-1.82	-1.55
ethyne − ethyne	C_2H_2	1.12	0.70	1.07	1.16	1.22	0.81	0.70	1.07	0.72	1.09	0.70	1.07	1.24	1.24	1.27	1.24	1.19
formaldehyde − formaldehyde	CH_2O	-4.55	-4.70	-3.90	-4.66	-5.03	-4.20	-4.70	-3.90	-4.68	-3.89	-4.70	-3.91	-4.69	-4.69	-4.68	-4.72	-3.72
hydrogencyanide − hydrogencyanide	HCN	-4.75	-5.03	-4.86	-5.83	-6.31	-4.72	-5.03	-4.86	-5.03	-4.86	-5.02	-4.85	-6.00	-6.00	-5.99	-6.00	-4.88
hydrogenfluoride − hydrogenfluoride	HF	-4.58	-4.70	-4.40	-4.59	-4.67	-4.54	-4.70	-4.40	-4.69	-4.39	-4.66	-4.37	-4.58	-4.58	-4.57	-4.55	-4.33
methane − argon	CH_4	-0.41	-0.52	-0.39	-0.31	-0.26	-0.49	-0.52	-0.39	-0.50	-0.37	-0.52	-0.39	-0.27	-0.27	-0.25	-0.27	-0.34
methane − ethane	CH_4	-0.83	-0.68	-0.52	-0.33	-0.23	-0.67	-0.68	-0.52	-0.68	-0.51	-0.68	-0.51	-0.27	-0.27	-0.26	-0.26	-0.45
methane − ethane	CH_4	-0.61	-0.97	-0.71	-0.52	-0.42	-0.92	-0.97	-0.71	-0.96	-0.71	-0.95	-0.69	-0.44	-0.44	-0.44	-0.43	-0.62
methane − ethene	CH_4	-0.50	-0.70	-0.55	-0.37	-0.28	-0.68	-0.70	-0.55	-0.69	-0.54	-0.68	-0.53	-0.31	-0.31	-0.31	-0.30	-0.49
methane − hydrogenfluoride	CH_4	-1.65	-1.80	-1.49	-1.48	-1.47	-1.68	-1.80	-1.49	-1.80	-1.48	-1.80	-1.49	-1.43	-1.43	-1.43	-1.43	-1.39
methane − methane	CH_4	-0.53	-0.59	-0.44	-0.30	-0.23	-0.57	-0.59	-0.44	-0.59	-0.43	-0.59	-0.43	-0.25	-0.25	-0.24	-0.25	-0.38
methane − water	CH_4	-0.66	-0.74	-0.61	-0.60	-0.59	-0.69	-0.74	-0.61	-0.74	-0.61	-0.73	-0.60	-0.58	-0.58	-0.58	-0.57	-0.57
water − ammonia	H_2O	-6.49	-6.72	-6.15	-6.38	-6.48	-6.45	-6.72	-6.15	-6.72	-6.15	-6.68	-6.10	-6.33	-6.33	-6.33	-6.30	-5.99
water − water	H_2O	-5.01	-5.19	-4.78	-5.01	-5.12	-4.97	-5.19	-4.78	-5.18	-4.77	-5.15	-4.74	-5.00	-5.00	-4.99	-4.97	-4.67

DFT

Table S11: Interaction energies for CCSD(T)/CBS, B97M-V, ωB97X-V, ωB97M-V, ωB97X-D3, B2PLYP-D3BJ, DSD-BLYP-D3BJ ωB97X-D4 and B2PLYP-D4. Data: 24 interaction energies. All values in kcal/mol.

Dimer	Formula	CCSD(T)/CBS	B97M-V	ωB97X-V	ωB97M-V	ωB97X-D3	B2PLYP-D3BJ	DSD-BLYP-D3BJ	ωB97X-D4	B2PLYP-D4
ammonia − ammonia	$NH_3{-}NH_3$	-3.14	-3.08	-3.17	-3.16	-3.15	-3.24	-3.21	-3.73	-3.15
ammonia − methane	$NH_3{-}CH_4$	-0.77	-0.73	-0.86	-0.76	-0.87	-0.75	-0.74	-1.14	-0.74
borane − methane	$BH_3{-}CH_4$	-1.49	-1.70	-1.49	-1.55	-1.61	-1.29	-1.30	-2.29	-1.19
ethene − ammonia	$C_2H_4{-}NH_3$	-1.37	-1.29	-1.47	-1.41	-1.57	-1.52	-1.52	-1.79	-1.46
ethene − Ar	$C_2H_4{-}Ar$	-0.36	-0.28	-0.41	-0.35	-0.38	-0.32	-0.33	-0.51	-0.27
ethene − ethene	$C_2H_4{-}C_2H_4$	-1.09	-0.96	-1.09	-1.00	-1.16	-1.14	-1.13	-1.54	-1.07
ethene − ethene	$C_2H_4{-}C_2H_4$	0.93	1.21	1.07	1.09	0.83	1.06	1.03	0.56	1.09
ethene − ethyne	$C_2H_4{-}C_2H_2$	0.82	0.88	0.78	0.79	0.60	0.83	0.80	0.26	0.87
ethene − formaldehyde	$C_2H_4{-}CH_2O$	-1.62	-1.54	-1.76	-1.69	-1.80	-1.63	-1.65	-2.22	-1.57
ethene − water	$C_2H_4{-}H_2O$	-2.56	-2.55	-2.73	-2.69	-2.78	-2.80	-2.80	-3.12	-2.69
ethyne − ethyne	$C_2H_2{-}C_2H_2$	-1.52	-1.81	-1.92	-1.87	-1.95	-1.78	-1.78	-2.27	-1.74
ethyne − ethyne	$C_2H_2{-}C_2H_2$	1.12	1.01	0.95	0.95	0.82	1.04	1.02	0.40	1.06
formaldehyde − formaldehyde	$CH_2O{-}CH_2O$	-4.55	-5.10	-4.74	-4.81	-4.59	-4.70	-4.69	-5.49	-4.65
hydrogencyanide − hydrogencyanide	$HCN{-}HCN$	-4.75	-5.63	-5.92	-5.86	-5.93	-5.50	-5.47	-6.50	-5.45
hydrogenfluoride − hydrogenfluoride	$HF{-}HF$	-4.58	-4.82	-4.67	-4.67	-4.49	-4.81	-4.79	-5.18	-4.76
methane − Ar	$CH_4{-}Ar$	-0.41	-0.31	-0.44	-0.36	-0.41	-0.37	-0.39	-0.52	-0.31
methane − ethane	$CH_4{-}C_2H_6$	-0.83	-0.41	-0.68	-0.50	-0.67	-0.52	-0.52	-0.87	-0.48
methane − ethane	$CH_4{-}C_2H_6$	-0.61	-0.61	-0.91	-0.74	-0.93	-0.75	-0.75	-1.18	-0.69
methane − ethene	$CH_4{-}C_2H_4$	-0.50	-0.42	-0.57	-0.46	-0.62	-0.50	-0.50	-0.76	-0.47
methane − hydrogenfluoride	$CH_4{-}HF$	-1.65	-1.69	-1.73	-1.68	-1.63	-1.69	-1.68	-2.13	-1.62
methane − methane	$CH_4{-}CH_4$	-0.53	-0.38	-0.59	-0.44	-0.60	-0.46	-0.45	-0.77	-0.42
methane − water	$CH_4{-}H_2O$	-0.66	-0.60	-0.72	-0.63	-0.63	-0.62	-0.64	-0.87	-0.62
water − ammonia	$H_2O{-}NH_3$	-6.49	-6.31	-6.64	-6.55	-6.85	-6.80	-6.71	-7.33	-6.71
water − water	$H_2O{-}H_2O$	-5.01	-4.97	-5.08	-5.07	-5.11	-5.18	-5.17	-5.71	-5.15

SAPT-DFT

Table S12: Interaction energies for CCSD(T)/CBS and SAPT-DFT. For SAPT-DFT method we used the B3LYP, PBE0 and ωB97X DFA's in conjunction with the aug-cc-pVDZ basis set. Data: 24 interaction energies. All values in kcal/mol.

Dimer	Formula	CCSD(T)/CBS	SAPT-B3LYP	SAPT-PBE0	SAPT-ωB97X
ammonia − ammonia	NH_3−NH_3	-3.14	-2.52	-2.59	-2.37
ammonia − methane	NH_3−CH_4	-0.77	-0.55	-0.58	-0.49
borane − methane	BH_3−CH_4	-1.49	-0.62	-0.63	-0.56
ethene − ammonia	C_2H_4−NH_3	-1.37	-1.19	-1.23	-1.01
ethene − argon	C_2H_4−Ar	-0.36	-0.29	-0.27	-0.14
ethene − ethene	C_2H_4−C_2H_4	-1.09	-0.98	-0.95	-0.57
ethene − ethene	C_2H_4−C_2H_4	0.93	1.05	1.11	1.62
ethene − ethyne	C_2H_4−C_2H_2	0.82	0.93	1.00	1.45
ethene − formaldehyde	C_2H_4−CH_2O	-1.62	-1.45	-1.46	-1.17
ethene − water	C_2H_4−H_2O	-2.56	-2.26	-2.38	-2.07
ethyne − ethyne	C_2H_2−C_2H_2	-1.52	-1.42	-1.48	-1.27
ethyne − ethyne	C_2H_2−C_2H_2	1.12	1.26	1.34	1.74
formaldehyde − formaldehyde	CH_2O−CH_2O	-4.55	-3.71	-3.71	-3.33
hydrogencyanide − hydrogencyanide	HCN−HCN	-4.75	-4.42	-4.45	-4.32
hydrogenfluoride − hydrogenfluoride	HF−HF	-4.58	-3.82	-3.90	-3.73
methane − argon	CH_4−Ar	-0.41	-0.28	-0.26	-0.19
methane − ethane	CH_4−C_2H_6	-0.61	-0.41	-0.38	-0.30
methane − ethane	CH_4−C_2H_6	-0.50	-0.56	-0.52	-0.41
methane − ethene	CH_4−C_2H_4	-0.50	-0.41	-0.41	-0.33
methane − hydrogenfluoride	CH_4−HF	-1.65	-1.21	-1.33	-1.25
methane − methane	CH_4−CH_4	-0.53	-0.34	-0.31	-0.26
methane − water	CH_4−H_2O	-0.66	-0.50	-0.52	-0.45
water − ammonia	H_2O−NH_3	-6.49	-5.46	-5.69	-5.32
water − water	H_2O−H_2O	-5.01	-4.23	-4.35	-4.08

2.2.4 Absolute deviations of molecular systems.

MP2 with C_{OS} y C_{SS} values

Table S13: Absolute deviations for MP2, SCS-MP2, SCS(MI)-MP2, SCSN-MP2, SCS-MP2-vdW, RI-MP2, RI-SCS-MP2, RIJK-MP2, RIJK-SCS-MP2, RIJCOSX-MP2, RIJCOSX-SCS-MP2, SCS-MP2^{BWI-TZ}, RI-SCS-MP2^{BWI-TZ}, RIJK-SCS-MP2^{BWI-TZ}, RIJCOSX-SCS-MP2^{BWI-DZ} and SCS-MP2-hal^{G-XZ}. Data: 24 interaction energies. MP2: $C_{OS} = C_{SS}$ =1.00; SCS-MP2: C_{OS} =1.20, C_{SS} =0.33; SCS(MI)-MP2: C_{OS} =0.40, C_{SS} =1.29; SCSN-MP2: C_{OS} =0.00, C_{SS} =1.76; SCS-MP2-vdW: C_{OS} =1.28, C_{SS} =0.50; SCS-MP2^{BWI-TZ} RI-SCS-MP2^{BWI-TZ} , RIJK-SCS-MP2^{BWI-TZ} : C_{OS} =0.27, C_{SS} =1.38; RIJCOSX-SCS-MP2^{BWI-TZ} : C_{OS} =0.17, C_{SS} =1.59. All values in kcal/mol.

Dimer	Formula	MP2	SCS-MP2	SCS(MI)-MP2	SCSN-MP2	SCS-MP2-vdW	RI-MP2	RI-SCS-MP2	RIJK-MP2	RIJK-SCS-MP2	RIJCOSX-MP2	RIJCOSX-SCS-MP2	SCS-MP2^{BWI-TZ}	RI-SCS-MP2^{BWI-TZ}	RIJK-SCS-MP2^{BWI-TZ}	RIJCOSX-SCS-MP2^{BWI-TZ}	SCS-MP2-hal^{G-XZ}
ammonia − ammonia	NH_3	0.15	0.24	0.18	0.15	0.01	0.15	0.24	0.15	0.24	0.13	0.26	0.22	0.22	0.23	0.23	0.35
ammonia − methane	NH_3	0.05	0.11	0.14	0.16	0.01	0.05	0.11	0.04	0.12	0.03	0.13	0.17	0.17	0.17	0.18	0.16
borane − methane	BH_3	0.02	0.51	0.54	0.57	0.18	0.02	0.51	0.01	0.52	0.02	0.52	0.63	0.63	0.64	0.62	0.68
ethene − ammonia	C_2H_4	0.40	0.10	0.02	0.08	0.32	0.40	0.10	0.40	0.10	0.39	0.08	0.09	0.09	0.09	0.10	0.00
ethene − argon	C_2H_4	0.15	0.01	0.12	0.19	0.13	0.15	0.01	0.14	0.00	0.14	0.00	0.16	0.16	0.18	0.18	0.04
ethene − ethene	C_2H_4	0.43	0.02	0.21	0.33	0.33	0.43	0.02	0.43	0.02	0.39	0.02	0.31	0.31	0.32	0.34	0.12
ethene − ethene	C_2H_4	0.45	0.04	0.56	0.87	0.44	0.45	0.04	0.44	0.03	0.41	0.00	0.74	0.74	0.75	0.76	0.13
ethene − ethyne	C_2H_4	0.45	0.05	0.26	0.44	0.38	0.45	0.05	0.43	0.04	0.43	0.04	0.39	0.39	0.40	0.39	0.09
ethene − formaldehyde	C_2H_4	0.26	0.13	0.07	0.06	0.10	0.26	0.13	0.25	0.13	0.24	0.14	0.12	0.12	0.13	0.13	0.24
ethene − water	C_2H_4	0.51	0.07	0.04	0.02	0.35	0.51	0.07	0.51	0.07	0.49	0.06	0.03	0.03	0.03	0.04	0.06
ethyne − ethyne	C_2H_2	0.35	0.09	0.30	0.40	0.19	0.35	0.09	0.35	0.09	0.34	0.08	0.30	0.30	0.30	0.30	0.03
ethyne − ethyne	C_2H_2	0.42	0.05	0.04	0.10	0.31	0.42	0.05	0.40	0.03	0.42	0.05	0.12	0.12	0.15	0.12	0.07
formaldehyde − formaldehyde	CH_2O	0.15	0.65	0.11	0.48	0.35	0.15	0.65	0.13	0.66	0.15	0.64	0.14	0.14	0.13	0.17	0.83
hydrogencyanide − hydrogencyanide	HCN	0.28	0.11	1.08	1.56	0.03	0.28	0.11	0.28	0.11	0.27	0.10	1.25	1.25	1.24	1.25	0.13
hydrogenfluoride − hydrogenfluoride	HF	0.12	0.18	0.01	0.09	0.04	0.12	0.18	0.11	0.19	0.08	0.21	0.00	0.00	0.01	0.03	0.25
methane − argon	CH_4	0.11	0.02	0.10	0.15	0.08	0.11	0.02	0.09	0.04	0.11	0.02	0.14	0.14	0.16	0.14	0.07
methane − ethane	CH_4	0.15	0.31	0.50	0.60	0.16	0.15	0.31	0.15	0.32	0.15	0.32	0.56	0.56	0.57	0.57	0.38
methane − ethane	CH_4	0.36	0.10	0.09	0.19	0.31	0.36	0.10	0.35	0.10	0.34	0.08	0.17	0.17	0.17	0.18	0.01
methane − ethene	CH_4	0.20	0.05	0.13	0.22	0.18	0.20	0.05	0.19	0.04	0.18	0.03	0.19	0.19	0.19	0.20	0.01
methane − hydrogenfluoride	CH_4	0.15	0.16	0.17	0.18	0.03	0.15	0.16	0.15	0.17	0.15	0.16	0.22	0.22	0.22	0.22	0.26
methane − methane	CH_4	0.06	0.09	0.23	0.30	0.04	0.06	0.09	0.06	0.10	0.06	0.10	0.28	0.28	0.29	0.28	0.15
methane − water	CH_4	0.08	0.05	0.06	0.07	0.03	0.08	0.05	0.08	0.05	0.07	0.06	0.08	0.08	0.08	0.09	0.09
water − ammonia	H_2O	0.23	0.34	0.11	0.01	0.04	0.23	0.34	0.23	0.34	0.19	0.39	0.16	0.16	0.16	0.19	0.50
water − water	H_2O	0.18	0.23	0.00	0.11	0.04	0.18	0.23	0.17	0.24	0.14	0.27	0.01	0.01	0.02	0.04	0.34

DFT

Table S14: Absolute deviations for B97M-V, ωB97X-V, ωB97M-V, ωB97X-D3, B2PLYP-D3BJ, DSD-BLYP-D3BJ, ωB97X-D4 and B2PLYP-D4. Data: 24 interaction energies. All values in kcal/mol.

Dimer	Formula	B97M-V	ωB97X-V	ωB97M-V	ωB97X-D3	B2PLYP-D3BJ	DSD-BLYP-D3BJ	ωB97X-D4	B2PLYP-D4
ammonia − ammonia	NH_3−NH_3	0.06	0.03	0.02	0.01	0.10	0.08	0.60	0.01
ammonia − methane	NH_3−CH_4	0.03	0.10	0.00	0.10	0.02	0.03	0.38	0.03
borane − methane	BH_3−CH_4	0.22	0.00	0.06	0.12	0.20	0.18	0.81	0.29
ethene − ammonia	C_2H_4−NH_3	0.08	0.10	0.03	0.20	0.14	0.15	0.42	0.08
ethene − argon	C_2H_4−Ar	0.08	0.04	0.01	0.02	0.05	0.04	0.15	0.10
ethene − ethene	C_2H_4−C_2H_4	0.13	0.00	0.09	0.07	0.05	0.04	0.45	0.02
ethene − ethene	C_2H_4−C_2H_4	0.27	0.13	0.15	0.10	0.12	0.10	0.37	0.16
ethene − ethyne	C_2H_4−C_2H_2	0.06	0.04	0.03	0.22	0.01	0.02	0.57	0.05
ethene − formaldehyde	C_2H_4−CH_2O	0.08	0.14	0.07	0.18	0.01	0.03	0.60	0.05
ethene − water	C_2H_4−H_2O	0.01	0.18	0.13	0.23	0.24	0.25	0.57	0.14
ethyne − ethyne	C_2H_2−C_2H_2	0.29	0.40	0.35	0.42	0.26	0.26	0.74	0.22
ethyne − ethyne	C_2H_2−C_2H_2	0.11	0.16	0.16	0.29	0.07	0.10	0.71	0.05
formaldehyde − formaldehyde	CH_2O−CH_2O	0.55	0.19	0.25	0.04	0.15	0.14	0.94	0.09
hydrogencyanide − hydrogencyanide	HCN−HCN	0.88	1.17	1.11	1.19	0.76	0.72	1.76	0.71
hydrogenfluoride − hydrogenfluoride	HF−HF	0.24	0.08	0.09	0.09	0.23	0.21	0.60	0.18
methane − argon	CH_4−Ar	0.10	0.04	0.05	0.01	0.04	0.02	0.12	0.10
methane − ethane	CH_4−C_2H_6	0.42	0.15	0.33	0.15	0.31	0.31	0.04	0.35
methane − ethane	CH_4−C_2H_6	0.01	0.30	0.13	0.33	0.14	0.14	0.58	0.08
methane − ethene	CH_4−C_2H_4	0.09	0.07	0.04	0.12	0.01	0.00	0.26	0.03
methane − hydrogenfluoride	CH_4−HF	0.03	0.08	0.03	0.03	0.03	0.03	0.47	0.04
methane − methane	CH_4−CH_4	0.16	0.06	0.10	0.07	0.08	0.08	0.24	0.12
methane − water	CH_4−H_2O	0.06	0.05	0.03	0.03	0.04	0.02	0.21	0.04
water − ammonia	H_2O−NH_3	0.18	0.15	0.05	0.35	0.30	0.22	0.84	0.22
water − water	H_2O−H_2O	0.03	0.08	0.06	0.11	0.17	0.16	0.70	0.14

SAPT-DFT

Table S15: Absolute deviations for SAPT-DFT with respect to CCSD(T)/CBS. In SAPT-DFT method we used the B3LYP, PBE0 and ωB97X DFA's in conjunction with the aug-cc-pVTZ basis set. Data: 24 interaction energies. All values in kcal/mol.

Dimer	Formula	SAPT-B3LYP	SAPT-PBE0	SAPT-ωB97X
ammonia − ammonia	NH_3-NH_3	2.52	5.11	7.48
ammonia − methane	NH_3-CH_4	0.55	1.13	1.61
borane − methane	BH_3-CH_4	0.62	1.25	1.81
ethene − ammonia	$C_2H_4-NH_3$	1.19	2.42	3.43
ethene − argon	C_2H_4-Ar	0.29	0.56	0.71
ethene − ethene	$C_2H_4-C_2H_4$	0.98	1.93	2.50
ethene − ethene	$C_2H_4-C_2H_4$	1.05	0.07	1.55
ethene − ethyne	$C_2H_4-C_2H_2$	0.93	0.07	1.38
ethene − formaldehyde	$C_2H_4-CH_2O$	1.45	2.91	4.07
ethene − water	$C_2H_4-H_2O$	2.26	4.64	6.71
ethyne − ethyne	$C_2H_2-C_2H_2$	1.42	2.91	4.18
ethyne − ethyne	$C_2H_2-C_2H_2$	1.26	0.08	1.66
formaldehyde − formaldehyde	CH_2O-CH_2O	3.71	7.42	10.75
hydrogencyanide − hydrogencyanide	$HCN-HCN$	4.42	8.87	13.19
hydrogenfluoride − hydrogenfluoride	$HF-HF$	3.82	7.72	11.45
methane − argon	CH_4-Ar	0.28	0.54	0.73
methane − ethane	$CH_4-C_2H_6$	0.41	0.79	1.09
methane − ethane	$CH_4-C_2H_6$	0.56	1.08	1.49
methane − ethene	$CH_4-C_2H_4$	0.41	0.82	1.16
methane − hydrogenfluoride	CH_4-HF	1.21	2.53	3.78
methane − methane	CH_4-CH_4	0.34	0.64	0.91
methane − water	CH_4-H_2O	0.50	1.03	1.47
water − ammonia	H_2O-NH_3	5.46	11.15	16.47
water − water	H_2O-H_2O	4.23	8.58	12.66

2.3 Mean calculation times

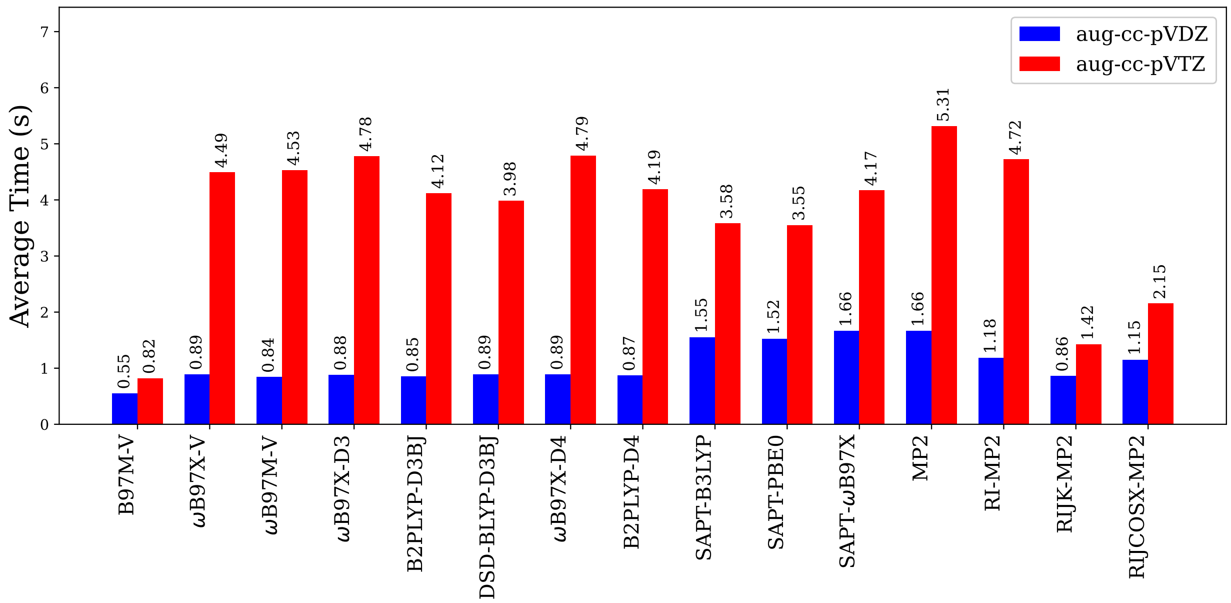

Figure S3: Comparison of computational times for differentn methods and basis sets in the dataset A24. All values in seconds.

3 HB300SPX(S,P)

3.1 aug-cc-pVDZ basis set data.

3.1.1 Ionization Potentials and HOMO values for SAPT-DFT calculations

Table S16: Experimental ionization potentials (IP) of molecules comprising dimers in the HB300SPX(S,P) database. IP obtained from NIST (https://webbook.nist.gov/chemistry). Kohn-Sham energies of HOMO orbitals were calculated at the DFA/aug-cc-pVDZ level of theory (DFA=B3LYP, PBE0, and ωB97X). Ionization energies in ev and HOMO energies in atomic units.

			HOMO energies (eV)			$\Delta_{XC} = \epsilon_{HOMO} - (-IP)$		
Molecule	Formula	IP	B3LYP	PBE0	ωB97X	B3LYP	PBE0	ωB97X
acetone	C_3H_6O	9.70	-6.91	-7.23	-9.71	0.10	0.09	0.00
ammonia	NH_3	10.07	-7.44	-7.77	-10.36	0.10	0.08	0.01
benzene	C_6H_6	9.24	-6.97	-7.28	-9.48	0.08	0.07	0.01
butadiene	C_4H_6	9.07	-6.50	-6.79	-9.00	0.09	0.08	0.00
carbondioxide	CO_2	13.78	-10.35	-10.70	-13.16	0.13	0.11	0.02
carbondisulfide	CS_2	10.07	-7.57	-7.90	-10.06	0.09	0.08	0.00
cyclobutadiene	C_4H_4	8.16	-5.25	-5.53	-7.85	0.11	0.10	0.01
cyclohexane	C_6H_{12}	9.88	-8.00	-8.27	-10.67	0.07	0.06	0.03
cyclopentadiene	C_5H_6	8.57	-6.02	-6.31	-8.59	0.09	0.08	0.00
cyclopentane	C_5H_{10}	10.33	-8.45	-8.71	-11.19	0.07	0.06	0.03
diazomethane	CH_2N_2	9.00	-6.24	-6.50	-8.73	0.10	0.09	0.01
dimethylbutane	C_6H_{14}	10.04	-8.33	-8.59	-11.02	0.06	0.05	0.04
dimethyldisulfide	$C_2H_6S_2$	7.40	-6.42	-6.73	-9.09	0.04	0.02	0.06
dimethylether	C_2H_6O	10.03	-7.13	-7.45	-9.92	0.11	0.09	0.00
dimethylperoxide	$C_2H_6O_2$	9.10	-7.03	-7.36	-9.89	0.08	0.06	0.03
dimethylsulfide	C_2H_6S	8.69	-5.98	-6.28	-8.68	0.10	0.09	0.00
diphospine	P_2H_4	8.80	-6.62	-6.90	-9.13	0.08	0.07	0.01
dithiethane	$C_4H_{10}S_2$	8.50	-6.30	-6.60	-8.99	0.08	0.07	0.02
ethane	C_2H_6	11.52	-9.30	-9.58	-12.09	0.08	0.07	0.02
ethene	C_2H_4	10.51	-7.54	-7.86	-10.29	0.11	0.10	0.01
formaldehyde	CH_2O	10.88	-7.54	-7.85	-10.33	0.12	0.11	0.02
hexane	C_6H_{14}	10.13	-8.36	-8.66	-11.07	0.07	0.05	0.03
methylamine	CH_3NH_2	8.90	-6.62	-6.93	-9.45	0.08	0.07	0.02
methylazide	CH_3N_3	9.81	-7.15	-7.43	-9.70	0.10	0.09	0.00
naphthalene	$C_{10}H_8$	8.14	-6.01	-6.30	-8.30	0.08	0.07	0.01
neohexane	C_6H_{14}	10.07	-8.37	-8.64	-11.07	0.06	0.05	0.04
neopentane	C_5H_{12}	10.30	-8.69	-8.97	-11.42	0.08	0.07	0.02
nonbornadiene	C_7H_8	8.38	-6.11	-6.40	-8.75	0.08	0.07	0.01
phosphine	PH_3	9.87	-7.53	-7.82	-10.21	0.09	0.08	0.01
phosphorine	C_5H_5P	9.00	-6.86	-7.16	-9.23	0.08	0.07	0.01
phosphorus	P_4	10.49	-7.28	-7.64	-9.76	0.12	0.10	0.03
propane	C_3H_8	10.94	-8.88	-9.14	-11.63	0.08	0.07	0.03
propene	C_3H_6	9.73	-7.04	-7.34	-9.73	0.10	0.09	0.00
thioacetone	C_3H_6S	8.60	-5.98	-6.30	-8.69	0.10	0.08	0.00
thiophene	C_4H_4S	8.86	-6.53	-6.85	-9.09	0.09	0.07	0.01
toluene	C_7H_8	8.83	-6.64	-6.94	-9.10	0.08	0.07	0.01
triazine	$C_3H_3N_3$	9.80	-7.81	-8.14	-10.63	0.07	0.06	0.03
triazole	$C_2H_3N_3$	9.80	-7.58	-7.88	-10.22	0.08	0.07	0.02

3.1.2 Evaluation of mean absolute deviation in a grid of C_{OS} and C_{SS} values

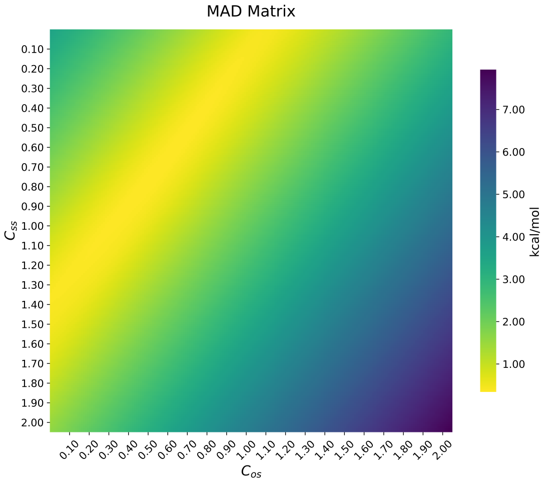

(a)

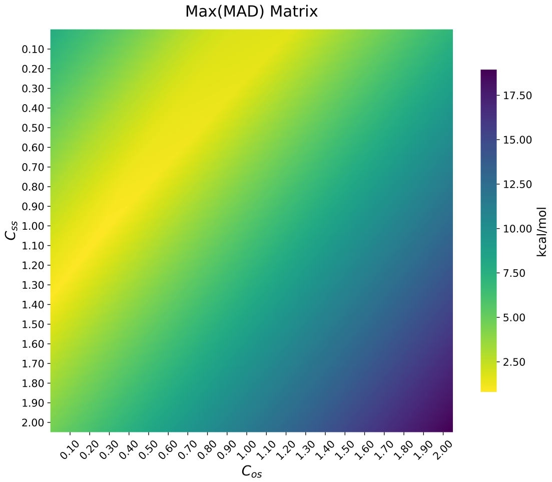

(b)

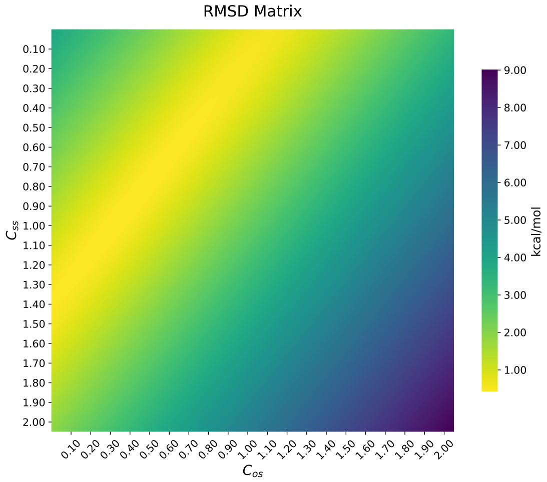

(c)

Figure S4: Evaluation of MADs (S4a), Max(MADs) (S4b) and RMSDs (S4c) in grid of values C_{OS} and C_{SS}. The optimal coefficients are C_{OS}=0.00 and C_{SS}=1.50. The theory level used is RIJK-MP2/aug-cc-pVDZ.

3.1.3 Interaction energies

MP2 with C_{OS} y C_{SS} values

Table S17: Interaction energies for CCSD(T)/CBS, MP2, SCS-MP2, SCS(MI)-MP2, SCSN-MP2, SCS-MP2-vdW, RI-MP2, RI-SCS-MP2, RIJK-MP2, RIJK-SCS-MP2, RIJCOSX-MP2, RIJCOSX-SCS-MP2, SCS-MP2^{BWI-DZ}, RI-SCS-MP2^{BWI-DZ}, RIJK-SCS-MP2^{BWI-DZ}, RIJCOSX-SCS-MP2^{BWI-DZ} and SCS-MP2-hal^{G-XZ}. Data: 50 interaction energies. MP2: $C_{OS} = C_{SS}$ =1.00; SCS-MP2: C_{OS} =1.20, C_{SS} =0.33; SCS(MI)-MP2: C_{OS} =0.40, C_{SS} =1.29; SCSN-MP2: C_{OS} =0.00, C_{SS} =1.76; SCS-MP2-vdW: C_{OS} =1.28, C_{SS} =0.50; SCS-MP2^{BWI-DZ} RI-SCS-MP2^{BWI-DZ} , RIJK-SCS-MP2^{BWI-DZ} : C_{OS} =0.00, C_{SS} =1.50; RIJCOSX-SCS-MP2^{BWI-DZ} : C_{OS} =0.00, C_{SS} =0.17. All values in kcal/mol.

Dimer	Formula	CCSD(T)/CBS	MP2	SCS-MP2	SCS(MI)-MP2	SCSN-MP2	SCS-MP2-vdW	RI-MP2	RI-SCS-MP2	RIJK-MP2	RIJK-SCS-MP2	RIJCOSX-MP2	RIJCOSX-SCS-MP2	SCS-MP2^{BWI-DZ}	RI-SCS-MP2^{BWI-DZ}	RIJK-SCS-MP2^{BWI-DZ}	RIJCOSX-SCS-MP2^{BWI-DZ}	SCS-MP2-hal^{G-XZ}
1 − 2 − 4 − triazole − thiophene	$C_2H_3N_3$	-2.98	-5.23	-4.26	-4.19	-4.13	-4.88	-5.24	-4.26	-5.24	-4.26	-5.23	-4.25	-3.53	-3.53	-3.53	-3.54	-3.63
1 − 3 − 5 − triazine − phosphine	$C_3H_3N_3$	-1.94	-2.92	-2.16	-2.28	-2.32	-2.60	-2.93	-2.16	-2.92	-2.16	-2.92	-2.15	-1.88	-1.89	-1.88	-1.90	-1.82
1 − 3 − 5 − triazine − phosphorine	$C_3H_3N_3$	-4.04	-7.67	-5.74	-5.85	-5.87	-6.90	-7.68	-5.74	-7.69	-5.75	-7.67	-5.73	-4.72	-4.73	-4.74	-4.77	-4.72
1 − 3 − 5 − triazine − thiophene	$C_3H_3N_3$	-3.41	-5.94	-4.91	-4.93	-4.91	-5.55	-5.95	-4.91	-5.95	-4.91	-5.95	-4.91	-4.29	-4.29	-4.29	-4.32	-4.32
2 − 3 − dimethylbutane − diphospine	C_6H_{14}	-2.74	-5.47	-4.45	-3.53	-3.05	-5.31	-5.47	-4.44	-5.47	-4.44	-5.45	-4.43	-2.29	-2.30	-2.29	-2.31	-3.03
acetone − phosphorine	C_3H_6O	-4.51	-7.46	-5.99	-6.04	-6.02	-6.89	-7.47	-6.00	-7.48	-6.00	-7.46	-5.99	-5.14	-5.15	-5.16	-5.17	-5.18
ammonia − phosphorine	NH_3	-2.23	-3.25	-2.55	-2.65	-2.68	-2.96	-3.25	-2.55	-3.25	-2.55	-3.24	-2.54	-2.27	-2.27	-2.27	-2.28	-2.23
ammonia − thiophene	NH_3	-2.28	-3.42	-2.97	-2.86	-2.79	-3.28	-3.42	-2.97	-3.42	-2.97	-3.41	-2.96	-2.50	-2.50	-2.49	-2.50	-2.60
benzene − carbondisulfide	C_6H_6	-3.03	-4.63	-3.18	-3.59	-3.77	-3.97	-4.65	-3.18	-4.64	-3.17	-4.64	-3.18	-2.95	-2.97	-2.96	-2.99	-2.70
benzene − phosphorine	C_6H_6	-3.48	-6.47	-5.10	-5.24	-5.28	-5.91	-6.48	-5.10	-6.48	-5.11	-6.47	-5.09	-4.47	-4.48	-4.49	-4.49	-4.42
butadinene − thiophene	C_4H_6	-2.58	-5.19	-3.95	-3.80	-3.69	-4.76	-5.20	-3.96	-5.20	-3.95	-5.21	-3.96	-2.91	-2.92	-2.91	-2.95	-3.09
carbondioxide − phosphorine	CO_2	-2.52	-2.73	-1.96	-2.77	-3.17	-2.23	-2.74	-1.97	-2.73	-1.96	-2.74	-1.97	-2.82	-2.83	-2.82	-2.84	-2.24
carbondioxide − thiophene	CO_2	-2.62	-2.80	-2.37	-2.94	-3.22	-2.49	-2.80	-2.37	-2.79	-2.35	-2.78	-2.35	-3.05	-3.06	-3.04	-3.05	-2.63
cyclobutadiene − dithiethane	C_4H_4	-4.22	-8.45	-6.26	-6.35	-6.35	-7.59	-8.47	-6.27	-8.46	-6.26	-8.45	-6.26	-5.04	-5.06	-5.05	-5.10	-5.07
cyclobutadiene − phosphorine	C_4H_4	-2.85	-5.09	-3.77	-4.07	-4.19	-4.51	-5.10	-3.78	-5.10	-3.77	-5.11	-3.78	-3.44	-3.45	-3.45	-3.47	-3.27
cyclohexane − diphosphine	C_6H_{12}	-2.35	-4.31	-3.61	-2.91	-2.54	-4.22	-4.31	-3.61	-4.31	-3.61	-4.28	-3.58	-2.01	-2.01	-2.01	-2.00	-2.58
cyclopentadiene − thiophene	C_5H_6	-2.38	-4.75	-4.10	-3.83	-3.68	-4.57	-4.76	-4.10	-4.76	-4.11	-4.75	-4.10	-3.24	-3.25	-3.25	-3.26	-3.48
cyclopentane − diphosphine	C_5H_{10}	-2.34	-4.20	-3.53	-2.87	-2.52	-4.12	-4.20	-3.53	-4.20	-3.53	-4.20	-3.53	-2.01	-2.01	-2.01	-2.03	-2.55
cyclopentane − phosphine	C_5H_{10}	-1.80	-3.15	-2.53	-1.99	-1.70	-3.05	-3.15	-2.53	-3.14	-2.52	-3.13	-2.51	-1.24	-1.25	-1.24	-1.25	-1.68
cyclopentane − phosphorus	C_5H_{10}	-3.25	-6.47	-4.96	-4.57	-4.34	-6.00	-6.48	-4.97	-6.50	-4.98	-6.47	-4.95	-3.37	-3.38	-3.40	-3.41	-3.73
diazomethane − carbondisulfide	CH_4	-2.18	-1.79	-1.01	-1.80	-2.18	-1.29	-1.80	-1.01	-1.78	-0.99	-1.80	-1.01	-1.82	-1.84	-1.82	-1.85	-1.26
diazomethane − diphosphine	CH_4	-2.96	-3.34	-2.48	-2.94	-3.14	-2.90	-3.34	-2.48	-3.33	-2.47	-3.34	-2.48	-2.69	-2.70	-2.68	-2.71	-2.39
diazomethane − phosphine	CH_4	-1.82	-1.60	-1.17	-1.68	-1.93	-1.31	-1.60	-1.17	-1.59	-1.15	-1.60	-1.16	-1.74	-1.74	-1.73	-1.74	-1.37
diazomethane − phosphorine	CH_4	-3.46	-4.64	-3.48	-4.08	-4.36	-4.04	-4.65	-3.48	-4.65	-3.48	-4.65	-3.49	-3.75	-3.76	-3.76	-3.78	-3.34
dimethylether − dimethyldisulfide	C_3H_6O	-3.75	-5.69	-4.84	-4.46	-4.25	-5.46	-5.70	-4.84	-5.69	-4.84	-5.68	-4.82	-3.67	-3.68	-3.68	-3.68	-4.00
dimethylether − thiophene	C_3H_6O	-2.82	-3.90	-3.54	-3.33	-3.21	-3.82	-3.90	-3.54	-3.89	-3.54	-3.88	-3.53	-2.96	-2.96	-2.95	-2.95	-3.14
dimethylperoxide − dimethylsulfide	$C_3H_6O_2$	-3.41	-5.10	-4.38	-4.05	-3.86	-4.91	-5.10	-4.38	-5.10	-4.38	-5.10	-4.37	-3.37	-3.37	-3.37	-3.38	-3.66
dimethylperoxide − thiophene	$C_3H_6O_2$	-2.35	-4.37	-3.86	-3.52	-3.33	-4.26	-4.37	-3.86	-4.37	-3.86	-4.35	-3.85	-2.98	-2.98	-2.97	-2.97	-3.26
Ethane − dimethyldisulfide	C_2H_6	-2.16	-3.88	-3.18	-2.71	-2.45	-3.73	-3.88	-3.18	-3.88	-3.18	-3.87	-3.17	-1.96	-1.96	-1.96	-1.97	-2.35
ethene − thiophene	C_2H_4	-2.18	-3.93	-3.28	-3.14	-3.06	-3.72	-3.93	-3.29	-3.93	-3.29	-3.92	-3.28	-2.65	-2.65	-2.66	-2.66	-2.78
formaldehyde − phosphorine	CH_2O	-3.18	-4.42	-3.22	-3.69	-3.89	-3.84	-4.43	-3.22	-4.43	-3.22	-4.44	-3.23	-3.24	-3.25	-3.25	-3.28	-2.94
formaldehyde − thiophene	CH_2O	-3.04	-4.14	-3.55	-3.79	-3.90	-3.85	-4.15	-3.55	-4.14	-3.54	-4.15	-3.55	-3.57	-3.58	-3.57	-3.59	-3.42
hexane − carbondisulfide	C_6H_{14}	-3.26	-5.57	-4.29	-3.91	-3.69	-5.18	-5.58	-4.29	-5.57	-4.28	-5.58	-4.29	-2.86	-2.89	-2.88	-2.92	-3.21
methylamine − thiophene	CH_3NH_2	-3.15	-5.39	-4.60	-4.35	-4.21	-5.15	-5.39	-4.60	-5.39	-4.60	-5.38	-4.58	-3.70	-3.70	-3.70	-3.70	-3.92
methylazide − thiophene	CH_3N_3	-4.20	-5.24	-4.36	-5.26	-5.70	-4.67	-5.24	-4.36	-5.24	-4.36	-5.24	-4.36	-5.31	-5.32	-5.31	-5.32	-4.66
naphthalene − carbondisulfide	$C_{10}H_8$	-3.25	-5.27	-3.90	-4.48	-4.74	-4.60	-5.28	-3.91	-5.28	-3.91	-5.27	-3.89	-3.99	-4.01	-4.01	-4.02	-3.62
naphthalene − dimethyldisulfide	$C_{10}H_8$	-5.03	-10.01	-7.76	-7.57	-7.42	-9.20	-10.02	-7.76	-10.03	-7.77	-9.96	-7.70	-6.03	-6.04	-6.05	-6.03	-6.28
naphthalene − diphospine	$C_{10}H_8$	-5.13	-9.03	-6.93	-6.99	-6.97	-8.22	-9.04	-6.94	-9.04	-6.94	-8.99	-6.89	-5.71	-5.72	-5.72	-5.73	-5.77
naphthalene − thioacetone	$C_{10}H_8$	-6.35	-12.01	-9.61	-9.55	-9.47	-11.11	-12.02	-9.62	-12.03	-9.63	-11.99	-9.58	-8.01	-8.02	-8.02	-8.04	-8.16
neohexane − diphospine	C_6H_{14}	-2.70	-4.91	-4.10	-3.29	-2.86	-4.80	-4.91	-4.10	-4.90	-4.10	-4.91	-4.10	-2.25	-2.26	-2.25	-2.29	-2.91
neopentane − phospine	C_5H_{12}	-1.28	-2.50	-2.10	-1.52	-1.22	-2.49	-2.50	-2.10	-2.49	-2.10	-2.47	-2.07	-0.90	-0.90	-0.90	-0.88	-1.36
nonbornadiene − dimethyldisulfide	C_7H_8	-3.20	-5.23	-4.38	-4.10	-3.94	-4.98	-5.23	-4.38	-5.23	-4.38	-5.21	-4.35	-3.39	-3.39	-3.39	-3.39	-3.64
propane − carbondisulfide	C_3H_8	-2.26	-3.53	-2.68	-2.53	-2.44	-3.24	-3.54	-2.68	-3.52	-2.67	-3.54	-2.68	-1.90	-1.92	-1.91	-1.95	-2.05
propane − diphospine	C_3H_8	-2.35	-4.20	-3.35	-2.80	-2.50	-4.02	-4.20	-3.35	-4.20	-3.35	-4.19	-3.34	-1.91	-1.91	-1.90	-1.91	-2.37
propane − phosphorine	C_3H_8	-2.92	-5.85	-4.57	-4.24	-4.04	-5.45	-5.86	-4.58	-5.87	-4.58	-5.86	-4.57	-3.21	-3.22	-3.23	-3.25	-3.53
propane − phospine	C_3H_8	-1.37	-2.41	-1.93	-1.52	-1.31	-2.33	-2.41	-1.93	-2.40	-1.92	-2.40	-1.92	-0.96	-0.96	-0.95	-0.97	-1.29
propene − thiophene	C_3H_8	-2.61	-5.16	-4.37	-4.11	-3.96	-4.93	-5.17	-4.37	-5.17	-4.37	-5.16	-4.36	-3.45	-3.45	-3.45	-3.46	-3.68
toluene − dimethylsulfide	$C_6H_5CH_3$	-4.29	-7.53	-6.08	-5.91	-5.80	-7.02	-7.53	-6.09	-7.53	-6.08	-7.52	-6.07	-4.90	-4.90	-4.90	-4.92	-5.09
toluene − diphospine	$C_6H_5CH_3$	-4.95	-8.37	-6.48	-6.33	-6.21	-7.69	-8.38	-6.49	-8.38	-6.49	-8.36	-6.47	-5.05	-5.06	-5.05	-5.09	-5.25
toluene − phosphorine	$C_6H_5CH_3$	-4.04	-9.36	-6.91	-6.71	-6.56	-8.48	-9.38	-6.92	-9.38	-6.92	-9.37	-6.92	-5.04	-5.06	-5.06	-5.11	-5.31

DFT

Table S18: Interaction energies for CCSD(T)/CBS, B97M-V, ωB97X-V, ωB97M-V, ωB97X-D3, B2PLYP-D3BJ, ωB97X-D4, B2PLYP-D4 and DSD-BLYP-D3BJ. Data: 50 interaction energies. All values in kcal/mol.

Dimer	Formula	CCSD(T)/CBS	B97M-V	ωB97X-V	ωB97M-V	ωB97X-D3	B2PLYP-D3BJ	DSD-BLYP-D3BJ	ωB97X-D4	B2PLYP-D4
$1-2-4-triazole-thiophene$	$C_2H_3N_3{-}C_4H_4S$	-2.98	-3.54	-3.84	-4.18	-4.04	-3.74	-4.01	-4.15	-3.54
$1-3-5-triazine-phosphine$	$C_3H_3N_3{-}PH_3$	-1.94	-1.95	-2.09	-2.23	-2.24	-2.21	-2.30	-2.68	-1.95
$1-3-5-triazine-phosphorine$	$C_3H_3N_3{-}PC_5H_5$	-4.04	-4.32	-4.45	-4.94	-4.96	-4.91	-5.29	-4.80	-4.32
$1-3-5-triazine-thiophene$	$C_3H_3N_3{-}C_4H_4S$	-3.41	-3.94	-4.16	-4.45	-4.44	-4.36	-4.65	-4.48	-3.94
$2-3-dimethylbutane-diphospine$	$C_6H_{14}{-}P_2H_4$	-2.74	-3.31	-3.47	-3.58	-3.68	-4.17	-4.20	-4.18	-3.31
$acetone-phosphorine$	$C_3H_6O{-}PC_5H_5$	-4.51	-5.09	-5.36	-5.67	-5.78	-5.50	-5.78	-5.83	-5.09
$ammonia-phosphorine$	$NH_3{-}PC_5H_5$	-2.23	-2.22	-2.50	-2.61	-2.82	-2.47	-2.60	-2.94	-2.22
$ammonia-thiophene$	$NH_3{-}C_4H_4S$	-2.28	-2.65	-2.93	-3.02	-3.25	-2.89	-2.98	-3.34	-2.65
$benzene-carbondisulfide$	$C_6H_6{-}CS_2$	-3.03	-2.71	-2.84	-3.27	-3.46	-2.88	-3.13	-3.42	-2.71
$benzene-phosphorine$	$C_6H_6{-}PC_5H_5$	-3.48	-3.76	-3.80	-4.20	-4.23	-4.30	-4.68	-4.39	-3.76
$butadinene-thiophene$	$C_4H_6{-}C_4H_4S$	-2.58	-3.29	-3.38	-3.93	-4.01	-3.49	-3.72	-3.91	-3.29
$carbondioxide-phosphorine$	$CO_2{-}PC_5H_5$	-2.52	-2.55	-2.59	-2.80	-2.67	-2.26	-2.39	-2.80	-2.55
$carbondioxide-thiophene$	$CO_2{-}C_4H_4S$	-2.62	-2.97	-3.08	-3.24	-2.96	-2.69	-2.79	-3.26	-2.97
$cyclobutadiene-dithiethane$	$C_4H_4{-}C_2H_4S_2$	-4.22	-6.26	-4.73	-5.49	-4.91	-6.01	-6.30	-5.19	-6.26
$cyclobutadiene-phosphorine$	$C_4H_4{-}PC_5H_5$	-2.85	-3.77	-3.26	-3.68	-3.46	-3.60	-3.86	-4.10	-3.77
$cyclohexane-diphosphine$	$C_6H_{12}{-}P_2H_4$	-2.35	-2.47	-2.76	-2.79	-2.78	-3.32	-3.38	-3.32	-2.47
$cyclopentadiene-thiophene$	$C_5H_6{-}C_4H_4S$	-2.38	-3.10	-3.33	-3.54	-3.61	-3.46	-3.71	-3.75	-3.10
$cyclopentane-diphosphine$	$C_5H_{10}{-}P_2H_4$	-2.34	-2.50	-2.81	-2.84	-2.82	-3.31	-3.36	-3.43	-2.50
$cyclopentane-phosphine$	$C_5H_{10}{-}PH_3$	-1.80	-2.18	-2.36	-2.36	-2.42	-2.65	-2.60	-3.01	-2.18
$cyclopentane-phosphorus$	$C_5H_{10}{-}P_4$	-3.25	-3.78	-4.03	-3.92	-4.04	-4.62	-4.80	-4.78	-3.78
$diazomethane-carbondisulfide$	$CH_4{-}CS_2$	-2.18	-1.82	-1.91	-2.06	-2.15	-1.45	-1.50	-2.53	-1.82
$diazomethane-diphosphine$	$CH_4{-}P_2H_4$	-2.96	-2.92	-2.95	-3.07	-2.96	-2.91	-2.95	-3.74	-2.92
$diazomethane-phosphine$	$CH_4{-}PH_3$	-1.82	-1.91	-1.97	-1.98	-2.02	-1.72	-1.68	-2.60	-1.91
$diazomethane-phosphorine$	$CH_4{-}PC_5H_5$	-3.46	-3.47	-3.59	-3.91	-4.04	-3.50	-3.67	-4.14	-3.47
$dimethylether-dimethyldisulfide$	$C_3H_6O{-}CH_3S_2CH_3$	-3.75	-4.15	-4.47	-4.40	-4.68	-4.91	-4.90	-4.99	-4.15
$dimethylether-thiophene$	$C_3H_6O{-}C_4H_4S$	-2.82	-3.03	-3.45	-3.46	-3.51	-3.45	-3.53	-3.82	-3.03
$dimethylperoxide-dimethylsulfide$	$C_3H_6O_2{-}CH_3SCH_3$	-3.41	-3.87	-4.26	-4.19	-4.42	-4.42	-4.45	-4.85	-3.87
$dimethylperoxide-thiophene$	$C_3H_6O_2{-}C_4H_4S$	-2.35	-3.24	-3.32	-3.46	-3.57	-3.59	-3.70	-3.76	-3.24
$ethane-dimethyldisulfide$	$C_2H_6{-}CH_3S_2CH_3$	-2.16	-2.59	-2.73	-2.75	-2.98	-3.11	-3.13	-3.27	-2.59
$ethene-thiophene$	$C_2H_4{-}C_4H_4S$	-2.18	-2.81	-2.92	-3.10	-3.24	-2.99	-3.14	-3.43	-2.81
$formaldehyde-phosphorine$	$CH_2O{-}PC_5H_5$	-3.18	-3.86	-3.44	-3.77	-3.63	-3.51	-3.63	-3.94	-3.86
$formaldehyde-thiophene$	$CH_2O{-}C_4H_4S$	-3.04	-3.72	-3.71	-3.88	-3.87	-3.67	-3.80	-4.15	-3.72
$hexane-carbondisulfide$	$C_6H_{14}{-}CS_2$	-3.26	-3.34	-3.57	-3.67	-4.03	-3.81	-4.02	-4.25	-3.34
$methylamine-thiophene$	$CH_3NH_2{-}C_4H_4S$	-3.15	-3.77	-4.09	-4.26	-4.39	-4.22	-4.42	-4.43	-3.77
$methylazide-thiophene$	$CH_3N_3{-}C_4H_4S$	-4.20	-4.49	-4.97	-5.16	-5.37	-4.25	-4.49	-5.53	-4.49
$naphthalene-carbondisulfide$	$C_{10}H_8{-}CS_2$	-3.25	-3.13	-3.18	-3.59	-3.55	-3.40	-3.79	-3.64	-3.13
$naphthalene-dimethyldisulfide$	$C_{10}H_8{-}CH_3S_3CH_3$	-5.03	-5.65	-5.78	-6.52	-6.47	-6.70	-7.12	-5.98	-5.65
$naphthalene-diphospine$	$C_{10}H_8{-}PSH_4$	-5.13	-5.20	-5.48	-6.07	-6.11	-6.28	-6.63	-5.92	-5.20
$naphthalene-thioacetone$	$C_{10}H_8{-}C_3H_6S$	-6.35	-7.39	-7.66	-8.32	-8.31	-8.25	-8.84	-7.84	-7.39
$neohexane-diphospine$	$C_6H_{14}{-}PSH_4$	-2.70	-2.99	-3.29	-3.31	-3.33	-3.79	-3.84	-3.93	-2.99
$neopentane-phospine$	$C_5H_{12}{-}PH_3$	-1.28	-1.69	-1.83	-1.75	-1.89	-1.99	-1.98	-2.36	-1.69
$nonbornadiene-dimethyldisulfide$	$C_7H_8{-}CH_3S_2CH_3$	-3.20	-3.68	-3.87	-4.09	-4.34	-4.33	-4.36	-4.36	-3.68
$propane-carbondisulfide$	$C_3H_8{-}CS_2$	-2.26	-2.21	-2.46	-2.49	-2.78	-2.42	-2.57	-3.03	-2.21
$propane-diphospine$	$C_3H_8{-}P_2H_4$	-2.35	-2.65	-2.81	-2.80	-2.86	-3.33	-3.35	-3.58	-2.65
$propane-phosphorine$	$C_3H_8{-}PC_5H_5$	-2.92	-3.46	-3.71	-4.00	-4.08	-3.96	-4.20	-4.20	-3.46
$propane-phospine$	$C_3H_8{-}PH_3$	-1.37	-1.70	-1.82	-1.78	-1.88	-2.00	-1.98	-2.40	-1.70
$propene-thiophene$	$C_3H_8{-}C_4H_4S$	-2.61	-3.44	-3.61	-3.79	-3.95	-3.86	-4.08	-4.05	-3.44
$toluene-dimethylsulfide$	$C_6H_5CH_3{-}CH_3SCH_3$	-4.29	-4.78	-4.91	-5.31	-5.31	-5.46	-5.76	-5.37	-4.78
$toluene-diphospine$	$C_6H_5CH_3{-}P_2H_4$	-4.95	-5.01	-5.15	-5.65	-5.44	-6.06	-6.35	-5.80	-5.01
$toluene-phosphorine$	$C_6H_5CH_3{-}PC_5H_5$	-4.04	-4.71	-4.74	-5.58	-5.48	-5.61	-6.10	-5.09	-4.71

SAPT-DFT

Table S19: Interaction energies for CCSD(T)/CBS and SAPT-DFT. For SAPT-DFT method we used the B3LYP, PBE0 and ωB97X DFA's in conjunction with the aug-cc-pVDZ basis set. Data: 50 interaction energies. All values in kcal/mol.

Dimer	Formula	CCSD(T)	SAPT-B3LYP	SAPT-PBE0	SAPT-ωB97X
$1-2-4-triazole-thiophene$	$C_2H_3N_3{-}C_4H_4S$	-2.98	-2.08	-2.00	-1.52
$1-3-5-triazine-phosphine$	$C_3H_3N_3{-}PH_3$	-1.94	-1.32	-1.28	-1.04
$1-3-5-triazine-phosphorine$	$C_3H_3N_3{-}PC_5H_5$	-4.04	-2.90	-2.85	-2.30
$1-3-5-triazine-thiophene$	$C_3H_3N_3{-}C_4H_4S$	-3.41	-2.42	-2.47	-2.13
$2-3-dimethylbutane-diphospine$	$C_6H_{14}{-}P_2H_4$	-2.74	-1.80	-1.72	-1.29
$acetone-phosphorine$	$C_3H_6O{-}PC_5H_5$	-4.51	-3.59	-3.66	-2.95
$ammonia-phosphorine$	$NH_3{-}PC_5H_5$	-2.23	-1.70	-1.75	-1.43
$ammonia-thiophene$	$NH_3{-}C_4H_4S$	-2.28	-1.76	-1.79	-1.46
$benzene-carbondisulfide$	$C_6H_6{-}CS_2$	-3.03	-2.19	-2.13	-1.55
$benzene-phosphorine$	$C_6H_6{-}PC_5H_5$	-3.48	-2.60	-2.68	-2.19
$butadinene-thiophene$	$C_4H_6{-}C_4H_4S$	-2.58	-1.87	-1.75	-1.07
$carbondioxide-phosphorine$	$CO_2{-}PC_5H_5$	-2.52	-1.85	-1.84	-1.45
$carbondioxide-thiophene$	$CO_2{-}C_4H_4S$	-2.62	-1.91	-1.91	-1.56
$cyclobutadiene-dithiethane$	$C_4H_4{-}C_2H_4S_2$	-4.22	-2.71	-2.78	-1.85
$cyclobutadiene-phosphorine$	$C_4H_4{-}PC_5H_5$	-2.85	-2.15	-2.17	-1.52
$cyclohexane-diphosphine$	$C_6H_{12}{-}P_2H_4$	-2.35	-1.65	-1.58	-1.23
$cyclopentadiene-thiophene$	$C_5H_6{-}C_4H_4S$	-2.38	-1.79	-1.80	-1.32
$cyclopentane-diphosphine$	$C_5H_{10}{-}P_2H_4$	-2.34	-1.66	-1.61	-1.29
$cyclopentane-phosphine$	$C_5H_{10}{-}PH_3$	-1.80	-1.26	-1.23	-0.88
$cyclopentane-phosphorus$	$C_5H_{10}{-}P_4$	-3.25	-2.49	-2.44	-1.80
$diazomethane-carbondisulfide$	$CH_4{-}CS_2$	-2.18	-1.53	-1.50	-1.08
$diazomethane-diphosphine$	$CH_4{-}P_2H_4$	-2.96	-2.23	-2.21	-1.76
$diazomethane-phosphine$	$CH_4{-}PH_3$	-1.82	-1.43	-1.43	-1.17
$diazomethane-phosphorine$	$CH_4{-}PC_5H_5$	-3.46	-2.65	-2.74	-2.21
$dimethylether-dimethyldisulfide$	$C_3H_6O{-}CH_3S_2CH_3$	-3.75	-2.62	-2.66	-2.45
$dimethylether-thiophene$	$C_3H_6O{-}C_4H_4S$	-2.82	-2.19	-2.23	-1.85
$dimethylperoxide-dimethylsulfide$	$C_3H_6O_2{-}CH_3SCH_3$	-3.41	-2.64	-2.65	-2.19
$dimethylperoxide-thiophene$	$C_3H_6O_2{-}C_4H_4S$	-2.35	-1.77	-1.78	-1.50
$ethane-dimethyldisulfide$	$C_2H_6{-}CH_3S_2CH_3$	-2.16	-1.41	-1.38	-1.28
$ethene-thiophene$	$C_2H_4{-}C_4H_4S$	-2.18	-1.70	-1.71	-1.25
$formaldehyde-phosphorine$	$CH_2O{-}PC_5H_5$	-3.18	-2.52	-2.56	-1.93
$formaldehyde-thiophene$	$CH_2O{-}C_4H_4S$	-3.04	-2.37	-2.44	-2.06
$hexane-carbondisulfide$	$C_6H_{14}{-}CS_2$	-3.26	-2.35	-2.28	-1.64
$methylamine-thiophene$	$CH_3NH_2{-}C_4H_4S$	-3.15	-2.37	-2.44	-2.02
$methylazide-thiophene$	$CH_3N_3{-}C_4H_4S$	-4.20	-3.21	-3.27	-2.64
$naphthalene-carbondisulfide$	$C_{10}H_8{-}CS_2$	-3.25	-2.45	-2.49	-1.99
$naphthalene-dimethyldisulfide$	$C_{10}H_8{-}CH_3S_3CH_3$	-5.03	-3.23	-3.19	-2.99
$naphthalene-diphospine$	$C_{10}H_8{-}PSH_4$	-5.13	-3.76	-3.81	-3.06
$naphthalene-thioacetone$	$C_{10}H_8{-}C_3H_6S$	-6.35	-4.99	-5.12	-4.11
$neohexane-diphospine$	$C_6H_{14}{-}PSH_4$	-2.70	-1.87	-1.80	-1.44
$neopentane-phospine$	$C_5H_{12}{-}PH_3$	-1.28	-0.93	-0.88	-0.57
$nonbornadiene-dimethyldisulfide$	$C_7H_8{-}CH_3S_2CH_3$	-3.20	-2.20	-2.29	-2.22
$propane-carbondisulfide$	$C_3H_8{-}CS_2$	-2.26	-1.61	-1.55	-1.12
$propane-diphospine$	$C_3H_8{-}P_2H_4$	-2.35	-1.63	-1.61	-1.18
$propane-phosphorine$	$C_3H_8{-}PC_5H_5$	-2.92	-2.16	-2.14	-1.62
$propane-phospine$	$C_3H_8{-}PH_3$	-1.37	-0.98	-0.94	-0.66
$propene-thiophene$	$C_3H_8{-}C_4H_4S$	-2.61	-1.92	-1.94	-1.56
$toluene-dimethylsulfide$	$C_6H_5CH_3{-}CH_3SCH_3$	-4.29	-3.34	-3.42	-2.65
$toluene-diphospine$	$C_6H_5CH_3{-}P_2H_4$	-4.95	-3.72	-3.71	-2.97
$toluene-phosphorine$	$C_6H_5CH_3{-}PC_5H_5$	-4.04	-2.95	-2.82	-1.89

3.1.4 Absolute deviations of molecular systems.

MP2 with C_{OS} y C_{SS} values

Table S20: Absolute deviations for MP2, SCS-MP2, SCS(MI)-MP2, SCSN-MP2, SCS-MP2-vdW, RI-MP2, RI-SCS-MP2, RIJK-MP2, RIJK-SCS-MP2, RIJCOSX-MP2, RIJCOSX-SCS-MP2, SCS-MP2^{BWI-DZ}, RI-SCS-MP2^{BWI-DZ}, RIJK-SCS-MP2^{BWI-DZ}, RIJCOSX-SCS-MP2^{BWI-DZ} and SCS-MP2-hal^{G-XZ}. Data: 50 interaction energies. MP2: C_{OS} = C_{SS} =1.00; SCS-MP2: C_{OS} =1.20, C_{SS} =0.33; SCS(MI)-MP2: C_{OS} =0.40, C_{SS} =1.29; SCSN-MP2: C_{OS} =0.00, C_{SS} =1.76; SCS-MP2-vdW: C_{OS} =1.28, C_{SS} =0.50; SCS-MP2^{BWI-DZ} RI-SCS-MP2^{BWI-DZ} , RIJK-SCS-MP2^{BWI-DZ} : C_{OS} =0.00, C_{SS} =1.50; RIJCOSX-SCS-MP2^{BWI-DZ} : C_{OS} =0.00, C_{SS} =0.17. All values in kcal/mol.

Dimer	Formula	MP2	SCS-MP2	SCS(MI)-MP2	SCSN-MP2	SCS-MP2-vdW	RI-MP2	RI-SCS-MP2	RIJK-MP2	RIJK-SCS-MP2	RIJCOSX-MP2	RIJCOSX-SCS-MP2	SCS-MP2^{BWI-DZ}	RI-SCS-MP2^{BWI-DZ}	RIJK-SCS-MP2^{BWI-DZ}	RIJCOSX-SCS-MP2^{BWI-DZ}	SCS-MP2-hal^{G-XZ}
1 − 2 − 4 − triazole − thiophene	$C_2H_3N_3$	2.25	1.28	1.21	1.15	1.90	2.26	1.28	2.26	1.28	2.25	1.27	0.55	0.55	0.55	0.56	0.65
1 − 3 − 5 − triazine − phosphine	$C_3H_3N_3$	0.98	0.22	0.34	0.38	0.66	0.99	0.22	0.98	0.22	0.98	0.21	0.06	0.05	0.06	0.04	0.12
1 − 3 − 5 − triazine − phosphorine	$C_3H_3N_3$	3.63	1.70	1.81	1.83	2.86	3.64	1.70	3.65	1.71	3.63	1.69	0.68	0.69	0.70	0.73	0.68
1 − 3 − 5 − triazine − thiophene	$C_3H_3N_3$	2.53	1.50	1.52	1.50	2.14	2.54	1.50	2.54	1.50	2.54	1.50	0.88	0.88	0.88	0.91	0.91
2 − 3 − dimethylbutane − diphospine	C_6H_{14}	2.73	1.71	0.79	0.31	2.57	2.73	1.70	2.73	1.70	2.71	1.69	0.45	0.44	0.45	0.43	0.29
acetone − phosphorine	C_3H_6O	2.95	1.48	1.53	1.51	2.38	2.96	1.49	2.97	1.49	2.95	1.48	0.63	0.64	0.65	0.66	0.67
ammonia − phosphorine	NH_3	1.02	0.32	0.42	0.45	0.73	1.02	0.32	1.02	0.32	1.01	0.31	0.04	0.04	0.04	0.05	0.00
ammonia − thiophene	NH_3	1.14	0.69	0.58	0.51	1.00	1.14	0.69	1.14	0.69	1.13	0.68	0.22	0.22	0.21	0.22	0.32
benzene − carbondisulfide	C_6H_6	1.60	0.15	0.56	0.74	0.94	1.62	0.15	1.61	0.14	1.61	0.15	0.08	0.06	0.07	0.04	0.33
benzene − phosphorine	C_6H_6	2.99	1.62	1.76	1.80	2.43	3.00	1.62	3.00	1.63	2.99	1.61	0.99	1.00	1.01	1.01	0.94
butadinene − thiophene	C_4H_6	2.61	1.37	1.22	1.11	2.18	2.62	1.38	2.62	1.37	2.63	1.38	0.33	0.34	0.33	0.37	0.51
carbondioxide − phosphorine	CO_2	0.21	0.56	0.25	0.65	0.29	0.22	0.55	0.21	0.56	0.22	0.55	0.30	0.31	0.30	0.32	0.28
carbondioxide − thiophene	CO_2	0.18	0.25	0.32	0.60	0.13	0.18	0.25	0.17	0.27	0.16	0.27	0.43	0.44	0.42	0.43	0.01
cyclobutadiene − dithiethane	C_4H_4	4.23	2.04	2.13	2.13	3.37	4.25	2.05	4.24	2.04	4.23	2.04	0.82	0.84	0.83	0.88	0.85
cyclobutadiene − phosphorine	C_4H_4	2.24	0.92	1.22	1.34	1.66	2.25	0.93	2.25	0.92	2.26	0.93	0.59	0.60	0.60	0.62	0.42
cyclohexane − diphosphine	C_6H_{12}	1.96	1.26	0.56	0.19	1.87	1.96	1.26	1.96	1.26	1.93	1.23	0.34	0.34	0.34	0.35	0.23
cyclopentadiene − thiophene	C_5H_6	2.37	1.72	1.45	1.30	2.19	2.38	1.72	2.38	1.73	2.37	1.72	0.86	0.87	0.87	0.88	1.10
cyclopentane − diphosphine	C_5H_{10}	1.86	1.19	0.53	0.18	1.78	1.86	1.19	1.86	1.19	1.86	1.19	0.33	0.33	0.33	0.31	0.21
cyclopentane − phosphine	C_5H_{10}	1.35	0.73	0.19	0.10	1.25	1.35	0.73	1.34	0.72	1.33	0.71	0.56	0.55	0.56	0.55	0.12
cyclopentane − phosphorus	C_5H_{10}	3.22	1.71	1.32	1.09	2.75	3.23	1.72	3.25	1.73	3.22	1.70	0.12	0.13	0.15	0.16	0.48
diazomethane − carbondisulfide	CH_4	0.39	1.17	0.38	0.00	0.89	0.38	1.17	0.40	1.19	0.38	1.17	0.36	0.34	0.36	0.33	0.92
diazomethane − diphosphine	CH_4	0.38	0.48	0.02	0.18	0.06	0.38	0.48	0.37	0.49	0.38	0.48	0.27	0.26	0.28	0.25	0.57
diazomethane − phosphine	CH_4	0.22	0.65	0.14	0.11	0.51	0.22	0.65	0.23	0.67	0.22	0.66	0.08	0.08	0.09	0.08	0.45
diazomethane − phosphorine	CH_4	1.18	0.02	0.62	0.90	0.58	1.19	0.02	1.19	0.02	1.19	0.03	0.29	0.30	0.30	0.32	0.12
dimethylether − dimethyldisulfide	C_3H_6O	1.94	1.09	0.71	0.50	1.71	1.95	1.09	1.94	1.09	1.93	1.07	0.08	0.07	0.07	0.07	0.25
dimethylether − thiophene	C_3H_6O	1.08	0.72	0.51	0.39	1.00	1.08	0.72	1.07	0.72	1.06	0.71	0.14	0.14	0.13	0.13	0.32
dimethylperoxide − dimethylsulfide	$C_3H_6O_2$	1.69	0.97	0.64	0.45	1.50	1.69	0.97	1.69	0.97	1.69	0.96	0.04	0.04	0.04	0.03	0.25
dimethylperoxide − thiophene	$C_3H_6O_2$	2.02	1.51	1.17	0.98	1.91	2.02	1.51	2.02	1.51	2.00	1.50	0.63	0.63	0.62	0.62	0.91
ethane − dimethyldisulfide	C_2H_6	1.72	1.02	0.55	0.29	1.57	1.72	1.02	1.72	1.02	1.71	1.01	0.20	0.20	0.20	0.19	0.19
ethene − thiophene	C_2H_4	1.75	1.10	0.96	0.88	1.54	1.75	1.11	1.75	1.11	1.74	1.10	0.47	0.47	0.48	0.48	0.60
formaldehyde − phosphorine	CH_2O	1.24	0.04	0.51	0.71	0.66	1.25	0.04	1.25	0.04	1.26	0.05	0.06	0.07	0.07	0.10	0.24
formaldehyde − thiophene	CH_2O	1.10	0.51	0.75	0.86	0.81	1.11	0.51	1.10	0.50	1.11	0.51	0.53	0.54	0.53	0.55	0.38
hexane − carbondisulfide	C_6H_{14}	2.31	1.03	0.65	0.43	1.92	2.32	1.03	2.31	1.02	2.32	1.03	0.40	0.37	0.38	0.34	0.05
methylamine − thiophene	CH_3NH_2	2.24	1.45	1.20	1.06	2.00	2.24	1.45	2.24	1.45	2.23	1.43	0.55	0.55	0.55	0.55	0.77
methylazide − thiophene	CH_3N_3	1.04	0.16	1.06	1.50	0.47	1.04	0.16	1.04	0.16	1.04	0.16	1.11	1.12	1.11	1.12	0.46
naphthalene − carbondisulfide	$C_{10}H_8$	2.02	0.65	1.23	1.49	1.35	2.03	0.66	2.03	0.66	2.02	0.64	0.74	0.76	0.76	0.77	0.37
naphthalene − dimethyldisulfide	$C_{10}H_8$	4.98	2.73	2.54	2.39	4.17	4.99	2.73	5.00	2.74	4.93	2.67	1.00	1.01	1.02	1.00	1.25
naphthalene − diphospine	$C_{10}H_8$	3.90	1.80	1.86	1.84	3.09	3.91	1.81	3.91	1.81	3.86	1.76	0.58	0.59	0.59	0.60	0.64
naphthalene − thioacetone	$C_{10}H_8$	5.66	3.26	3.20	3.12	4.76	5.67	3.27	5.68	3.28	5.64	3.23	1.66	1.67	1.67	1.69	1.81
neohexane − diphospine	C_6H_{14}	2.21	1.40	0.59	0.16	2.10	2.21	1.40	2.20	1.40	2.21	1.40	0.45	0.44	0.45	0.41	0.21
neopentane − phospine	C_5H_{12}	1.22	0.82	0.24	0.06	1.21	1.22	0.82	1.21	0.82	1.19	0.79	0.38	0.38	0.38	0.40	0.08
nonbornadiene − dimethyldisulfide	C_7H_8	2.03	1.18	0.90	0.74	1.78	2.03	1.18	2.03	1.18	2.01	1.15	0.19	0.19	0.19	0.19	0.44
propane − carbondisulfide	C_3H_8	1.27	0.42	0.27	0.18	0.98	1.28	0.42	1.26	0.41	1.28	0.42	0.36	0.34	0.35	0.31	0.21
propane − diphospine	C_3H_8	1.85	1.00	0.45	0.15	1.67	1.85	1.00	1.85	1.00	1.84	0.99	0.44	0.44	0.45	0.44	0.02
propane − phosphorine	C_3H_8	2.93	1.65	1.32	1.12	2.53	2.94	1.66	2.95	1.66	2.94	1.65	0.29	0.30	0.31	0.33	0.61
propane − phospine	C_3H_8	1.04	0.56	0.15	0.06	0.96	1.04	0.56	1.03	0.55	1.03	0.55	0.41	0.41	0.42	0.40	0.08
propene − thiophene	C_3H_8	2.55	1.76	1.50	1.35	2.32	2.56	1.76	2.56	1.76	2.55	1.75	0.84	0.84	0.84	0.85	1.07
toluene − dimethylsulfide	$C_6H_5CH_3$	3.24	1.79	1.62	1.51	2.73	3.24	1.80	3.24	1.79	3.23	1.78	0.61	0.61	0.61	0.63	0.80
toluene − diphospine	$C_6H_5CH_3$	3.42	1.53	1.38	1.26	2.74	3.43	1.54	3.43	1.54	3.41	1.52	0.10	0.11	0.10	0.14	0.30
toluene − phosphorine	$C_6H_5CH_3$	5.32	2.87	2.67	2.52	4.44	5.34	2.88	5.34	2.88	5.33	2.88	1.00	1.02	1.02	1.07	1.27

DFT

Table S21: Absolute deviations for B97M-V, ωB97X-V, ωB97M-V, ωB97X-D3, B2PLYP-D3BJ, DSD-BLYP-D3BJ, ωB97X-D4 and B2PLYP-D4. Data: 50 interaction energies. All values in kcal/mol.

Dimer	Formula	B97M-V	ωB97X-V	ωB97M-V	ωB97X-D3	B2PLYP-D3BJ	DSD-BLYP-D3BJ	ωB97X-D4	B2PLYP-D4
1 − 2 − 4 − triazole − thiophene	$C_2H_3N_3{-}C_4H_4S$	0.56	0.86	1.20	1.06	0.76	1.03	1.17	0.56
1 − 3 − 5 − triazine − phosphine	$C_3H_3N_3{-}PH_3$	0.01	0.15	0.29	0.30	0.27	0.36	0.74	0.01
1 − 3 − 5 − triazine − phosphorine	$C_3H_3N_3{-}PC_5H_5$	0.28	0.41	0.90	0.92	0.87	1.25	0.76	0.28
1 − 3 − 5 − triazine − thiophene	$C_3H_3N_3{-}C_4H_4S$	0.53	0.75	1.04	1.03	0.95	1.24	1.07	0.53
2 − 3 − dimethylbutane − diphospine	$C_6H_{14}{-}P_2H_4$	0.57	0.73	0.84	0.94	1.43	1.46	1.44	0.57
acetone − phosphorine	$C_3H_6O{-}PC_5H_5$	0.58	0.85	1.16	1.27	0.99	1.27	1.32	0.58
ammonia − phosphorine	$NH_3{-}PC_5H_5$	0.01	0.27	0.38	0.59	0.24	0.37	0.71	0.01
ammonia − thiophene	$NH_3{-}C_4H_4S$	0.37	0.65	0.74	0.97	0.61	0.70	1.06	0.37
benzene − carbondisulfide	$C_6H_6{-}CS_2$	0.32	0.19	0.24	0.43	0.15	0.10	0.39	0.32
benzene − phosphorine	$C_6H_6{-}PC_5H_5$	0.28	0.32	0.72	0.75	0.82	1.20	0.91	0.28
butadinene − thiophene	$C_4H_6{-}C_4H_4S$	0.71	0.80	1.35	1.43	0.91	1.14	1.33	0.71
carbondioxide − phosphorine	$CO_2{-}PC_5H_5$	0.03	0.07	0.28	0.15	0.26	0.13	0.28	0.03
carbondioxide − thiophene	$CO_2{-}C_4H_4S$	0.35	0.46	0.62	0.34	0.07	0.17	0.64	0.35
cyclobutadiene − dithiethane	$C_4H_4{-}C_2H_4S_2$	2.04	0.51	1.27	0.69	1.79	2.08	0.97	2.04
cyclobutadiene − phosphorine	$C_4H_4{-}PC_5H_5$	0.92	0.41	0.83	0.61	0.75	1.01	1.25	0.92
cyclohexane − diphosphine	$C_6H_{12}{-}P_2H_4$	0.12	0.41	0.44	0.43	0.97	1.03	0.97	0.12
cyclopentadiene − thiophene	$C_5H_6{-}C_4H_4S$	0.72	0.95	1.16	1.23	1.08	1.33	1.37	0.72
cyclopentane − diphosphine	$C_5H_{10}{-}P_2H_4$	0.16	0.47	0.50	0.48	0.97	1.02	1.09	0.16
cyclopentane − phosphine	$C_5H_{10}{-}PH_3$	0.38	0.56	0.56	0.62	0.85	0.80	1.21	0.38
cyclopentane − phosphorus	$C_5H_{10}{-}P_4$	0.53	0.78	0.67	0.79	1.37	1.55	1.53	0.53
diazomethane − carbondisulfide	$CH_4{-}CS_2$	0.36	0.27	0.12	0.03	0.73	0.68	0.35	0.36
diazomethane − diphosphine	$CH_4{-}P_2H_4$	0.04	0.01	0.11	0.00	0.05	0.01	0.78	0.04
diazomethane − phosphine	$CH_4{-}PH_3$	0.09	0.15	0.16	0.20	0.10	0.14	0.78	0.09
diazomethane − phosphorine	$CH_4{-}PC_5H_5$	0.01	0.13	0.45	0.58	0.04	0.21	0.68	0.01
dimethylether − dimethyldisulfide	$C_3H_6O{-}CH_3S_2CH_3$	0.40	0.72	0.65	0.93	1.16	1.15	1.24	0.40
dimethylether − thiophene	$C_3H_6O{-}C_4H_4S$	0.21	0.63	0.64	0.69	0.63	0.71	1.00	0.21
dimethylperoxide − dimethylsulfide	$C_3H_6O_2{-}CH_3SCH_3$	0.46	0.85	0.78	1.01	1.01	1.04	1.44	0.46
dimethylperoxide − thiophene	$C_3H_6O_2{-}C_4H_4S$	0.89	0.97	1.11	1.22	1.24	1.35	1.41	0.89
ethane − dimethyldisulfide	$C_2H_6{-}CH_3S_2CH_3$	0.43	0.57	0.59	0.82	0.95	0.97	1.11	0.43
ethene − thiophene	$C_2H_4{-}C_4H_4S$	0.63	0.74	0.92	1.06	0.81	0.96	1.25	0.63
formaldehyde − phosphorine	$CH_2O{-}PC_5H_5$	0.68	0.26	0.59	0.45	0.33	0.45	0.76	0.68
formaldehyde − thiophene	$CH_2O{-}C_4H_4S$	0.68	0.67	0.84	0.83	0.63	0.76	1.11	0.68
hexane − carbondisulfide	$C_6H_{14}{-}CS_2$	0.08	0.31	0.41	0.77	0.55	0.76	0.99	0.08
methylamine − thiophene	$CH_3NH_2{-}C_4H_4S$	0.62	0.94	1.11	1.24	1.07	1.27	1.28	0.62
methylazide − thiophene	$CH_3N_3{-}C_4H_4S$	0.29	0.77	0.96	1.17	0.05	0.29	1.33	0.29
naphthalene − carbondisulfide	$C_{10}H_8{-}CS_2$	0.12	0.07	0.34	0.30	0.15	0.54	0.39	0.12
naphthalene − dimethyldisulfide	$C_{10}H_8{-}CH_3S_3CH_3$	0.62	0.75	1.49	1.44	1.67	2.09	0.95	0.62
naphthalene − diphospine	$C_{10}H_8{-}PSH_4$	0.07	0.35	0.94	0.98	1.15	1.50	0.79	0.07
naphthalene − thioacetone	$C_{10}H_8{-}C_3H_6S$	1.04	1.31	1.97	1.96	1.90	2.49	1.49	1.04
neohexane − diphospine	$C_6H_{14}{-}PSH_4$	0.29	0.59	0.61	0.63	1.09	1.14	1.23	0.29
neopentane − phospine	$C_5H_{12}{-}PH_3$	0.41	0.55	0.47	0.61	0.71	0.70	1.08	0.41
nonbornadiene − dimethyldisulfide	$C_7H_8{-}CH_3S_2CH_3$	0.48	0.67	0.89	1.14	1.13	1.16	1.16	0.48
propane − carbondisulfide	$C_3H_8{-}CS_2$	0.05	0.20	0.23	0.52	0.16	0.31	0.77	0.05
propane − diphospine	$C_3H_8{-}P_2H_4$	0.30	0.46	0.45	0.51	0.98	1.00	1.23	0.30
propane − phosphorine	$C_3H_8{-}PC_5H_5$	0.54	0.79	1.08	1.16	1.04	1.28	1.28	0.54
propane − phospine	$C_3H_8{-}PH_3$	0.33	0.45	0.41	0.51	0.63	0.61	1.03	0.33
propene − thiophene	$C_3H_8{-}C_4H_4S$	0.83	1.00	1.18	1.34	1.25	1.47	1.44	0.83
toluene − dimethylsulfide	$C_6H_5CH_3{-}CH_3SCH_3$	0.49	0.62	1.02	1.02	1.17	1.47	1.08	0.49
toluene − diphospine	$C_6H_5CH_3{-}P_2H_4$	0.06	0.20	0.70	0.49	1.11	1.40	0.85	0.06
toluene − phosphorine	$C_6H_5CH_3{-}PC_5H_5$	0.67	0.70	1.54	1.44	1.57	2.06	1.05	0.67

SAPT-DFT

Table S22: Absolute deviations for SAPT-DFT with respect to CCSD(T)/CBS. In SAPT-DFT method we used the B3LYP, PBE0 and ωB97X DFA's in conjunction with the aug-cc-pVTZ basis set. Data: 50 interaction energies. All values in kcal/mol.

Dimer	Formula	SAPT-B3LYP	SAPT-PBE0	SAPT-ωB97X
$1-2-4-triazole-thiophene$	$C_2H_3N_3{-}C_4H_4S$	1.25	1.31	1.75
$1-3-5-triazine-phosphine$	$C_3H_3N_3{-}PH_3$	0.91	0.93	1.15
$1-3-5-triazine-phosphorine$	$C_3H_3N_3{-}PC_5H_5$	1.57	1.59	2.10
$1-3-5-triazine-thiophene$	$C_3H_3N_3{-}C_4H_4S$	1.33	1.24	1.55
$2-3-dimethylbutane-diphospine$	$C_6H_{14}{-}P_2H_4$	1.37	1.44	1.85
$acetone-phosphorine$	$C_3H_6O{-}PC_5H_5$	1.32	1.21	1.89
$ammonia-phosphorine$	$NH_3{-}PC_5H_5$	0.80	0.73	1.02
$ammonia-thiophene$	$NH_3{-}C_4H_4S$	0.81	0.74	1.04
$benzene-carbondisulfide$	$C_6H_6{-}CS_2$	1.37	1.36	1.93
$benzene-phosphorine$	$C_6H_6{-}PC_5H_5$	1.22	1.09	1.55
$butadinene-thiophene$	$C_4H_6{-}C_4H_4S$	1.09	1.18	1.86
$carbondioxide-phosphorine$	$CO_2{-}PC_5H_5$	1.05	1.02	1.37
$carbondioxide-thiophene$	$CO_2{-}C_4H_4S$	1.07	1.03	1.33
$cyclobutadiene-dithiethane$	$C_4H_4{-}C_2H_4S_2$	2.33	2.20	3.10
$cyclobutadiene-phosphorine$	$C_4H_4{-}PC_5H_5$	1.12	1.05	1.68
$cyclohexane-diphosphine$	$C_6H_{12}{-}P_2H_4$	1.06	1.12	1.45
$cyclopentadiene-thiophene$	$C_5H_6{-}C_4H_4S$	0.86	0.82	1.29
$cyclopentane-diphosphine$	$C_5H_{10}{-}P_2H_4$	1.01	1.05	1.35
$cyclopentane-phosphine$	$C_5H_{10}{-}PH_3$	0.84	0.86	1.19
$cyclopentane-phosphorus$	$C_5H_{10}{-}P_4$	1.25	1.29	1.88
$diazomethane-carbondisulfide$	$CH_4{-}CS_2$	1.08	1.08	1.48
$diazomethane-diphosphine$	$CH_4{-}P_2H_4$	1.17	1.16	1.57
$diazomethane-phosphine$	$CH_4{-}PH_3$	0.61	0.59	0.82
$diazomethane-phosphorine$	$CH_4{-}PC_5H_5$	1.17	1.04	1.54
$dimethylether-dimethyldisulfide$	$C_3H_6O{-}CH_3S_2CH_3$	1.50	1.43	1.60
$dimethylether-thiophene$	$C_3H_6O{-}C_4H_4S$	0.89	0.80	1.15
$dimethylperoxide-dimethylsulfide$	$C_3H_6O_2{-}CH_3SCH_3$	1.10	1.06	1.48
$dimethylperoxide-thiophene$	$C_3H_6O_2{-}C_4H_4S$	0.81	0.77	1.04
$ethane-dimethyldisulfide$	$C_2H_6{-}CH_3S_2CH_3$	1.06	1.08	1.15
$ethene-thiophene$	$C_2H_4{-}C_4H_4S$	0.76	0.73	1.18
$formaldehyde-phosphorine$	$CH_2O{-}PC_5H_5$	1.09	1.02	1.59
$formaldehyde-thiophene$	$CH_2O{-}C_4H_4S$	1.03	0.91	1.25
$hexane-carbondisulfide$	$C_6H_{14}{-}CS_2$	1.38	1.44	2.03
$methylamine-thiophene$	$CH_3NH_2{-}C_4H_4S$	1.16	1.07	1.46
$methylazide-thiophene$	$CH_3N_3{-}C_4H_4S$	1.42	1.31	1.91
$naphthalene-carbondisulfide$	$C_{10}H_8{-}CS_2$	1.18	1.07	1.58
$naphthalene-dimethyldisulfide$	$C_{10}H_8{-}CH_3S_3CH_3$	2.44	2.44	2.61
$naphthalene-diphospine$	$C_{10}H_8{-}PSH_4$	1.99	1.86	2.58
$naphthalene-thioacetone$	$C_{10}H_8{-}C_3H_6S$	1.92	1.73	2.70
$neohexane-diphospine$	$C_6H_{14}{-}PSH_4$	1.21	1.27	1.60
$neopentane-phospine$	$C_5H_{12}{-}PH_3$	0.58	0.62	0.91
$nonbornadiene-dimethyldisulfide$	$C_7H_8{-}CH_3S_2CH_3$	1.31	1.18	1.23
$propane-carbondisulfide$	$C_3H_8{-}CS_2$	1.02	1.06	1.48
$propane-diphospine$	$C_3H_8{-}P_2H_4$	1.12	1.13	1.53
$propane-phosphorine$	$C_3H_8{-}PC_5H_5$	1.10	1.12	1.60
$propane-phospine$	$C_3H_8{-}PH_3$	0.65	0.68	0.94
$propene-thiophene$	$C_3H_8{-}C_4H_4S$	0.97	0.93	1.29
$toluene-dimethylsulfide$	$C_6H_5CH_3{-}CH_3SCH_3$	1.38	1.26	1.99
$toluene-diphospine$	$C_6H_5CH_3{-}P_2H_4$	1.90	1.83	2.57
$toluene-phosphorine$	$C_6H_5CH_3{-}PC_5H_5$	1.64	1.73	2.65

3.2 aug-cc-pVTZ basis set data.

3.2.1 Ionization Potentials and HOMO values for SAPT-DFT calculations

Table S23: Experimental ionization potentials (IP) of molecules comprising dimers in the HB300SPX(S,P) database. IP obtained from NIST (https://webbook.nist.gov/chemistry). Kohn-Sham energies of HOMO orbitals were calculated at the DFA/aug-cc-pVTZ level of theory (DFA=B3LYP, PBE0, and ωB97X). Ionization energies in ev and HOMO energies in atomic units.

			HOMO energies (eV)			$\Delta_{XC} = \epsilon_{HOMO} - (-IP)$		
Molecule	Formula	IP	B3LYP	PBE0	ωB97X	B3LYP	PBE0	ωB97X
acetone	C_3H_6O	9.70	-6.94	-7.26	-9.73	0.10	0.09	0.00
ammonia	NH_3	10.07	-7.46	-7.78	-10.36	0.10	0.08	0.01
benzene	C_6H_6	9.24	-6.99	-7.30	-9.47	0.08	0.07	0.01
butadiene	C_4H_6	9.07	-6.52	-6.81	-9.00	0.09	0.08	0.00
carbondioxide	CO_2	13.78	-10.36	-10.72	-13.14	0.13	0.11	0.02
carbondisulfide	CS_2	10.07	-7.55	-7.88	-9.99	0.09	0.08	0.00
cyclobutadiene	C_4H_4	8.16	-5.26	-5.54	-7.85	0.11	0.10	0.01
cyclohexane	C_6H_{12}	9.88	-8.02	-8.29	-10.68	0.07	0.06	0.03
cyclopentadiene	C_5H_6	8.57	-6.03	-6.33	-8.58	0.09	0.08	0.00
cyclopentane	C_5H_{10}	10.33	-8.47	-8.73	-11.20	0.07	0.06	0.03
diazomethane	CH_2N_2	9.00	-6.26	-6.52	-8.73	0.10	0.09	0.01
dimethylbutane	C_6H_{14}	10.04	-8.35	-8.61	-11.03	0.06	0.05	0.04
dimethyldisulfide	$C_2H_6S_2$	7.40	-6.44	-6.74	-9.08	0.04	0.02	0.06
dimethylether	C_2H_6O	10.03	-7.17	-7.49	-9.95	0.10	0.09	0.00
dimethylperoxide	$C_2H_6O_2$	9.10	-7.06	-7.39	-9.91	0.07	0.06	0.03
dimethylsulfide	C_2H_6S	8.69	-6.00	-6.30	-8.68	0.10	0.09	0.00
diphospine	P_2H_4	8.80	-6.63	-6.91	-9.13	0.08	0.07	0.01
dithiethane	$C_4H_{10}S_2$	8.50	-6.31	-6.61	-8.98	0.08	0.07	0.02
ethane	C_2H_6	11.52	-9.32	-9.59	-12.10	0.08	0.07	0.02
ethene	C_2H_4	10.51	-7.56	-7.89	-10.30	0.11	0.10	0.01
formaldehyde	CH_2O	10.88	-7.58	-7.88	-10.35	0.12	0.11	0.02
hexane	C_6H_{14}	10.13	-8.38	-8.67	-11.08	0.06	0.05	0.03
methylamine	CH_3NH_2	8.90	-6.65	-6.96	-9.46	0.08	0.07	0.02
methylazide	CH_3N_3	9.81	-7.17	-7.44	-9.69	0.10	0.09	0.00
naphthalene	$C_{10}H_8$	8.14	-6.02	-6.31	-8.29	0.08	0.07	0.01
neohexane	C_6H_{14}	10.07	-8.39	-8.66	-11.08	0.06	0.05	0.04
neopentane	C_5H_{12}	10.30	-8.70	-8.98	-11.42	0.08	0.07	0.02
nonbornadiene	C_7H_8	8.38	-6.12	-6.42	-8.75	0.08	0.07	0.01
phosphine	PH_3	9.87	-7.56	-7.85	-10.22	0.08	0.07	0.01
phosphorine	C_5H_5P	9.00	-6.85	-7.16	-9.20	0.08	0.07	0.01
phosphorus	P_4	10.49	-7.25	-7.62	-9.71	0.12	0.11	0.03
propane	C_3H_8	10.94	-8.90	-9.16	-11.65	0.07	0.07	0.03
propene	C_3H_6	9.73	-7.05	-7.36	-9.73	0.10	0.09	0.00
thioacetone	C_3H_6S	8.60	-6.00	-6.31	-8.70	0.10	0.08	0.00
thiophene	C_4H_4S	8.86	-6.55	-6.86	-9.09	0.09	0.07	0.01
toluene	C_7H_8	8.83	-6.65	-6.95	-9.10	0.08	0.07	0.01
triazine	$C_3H_3N_3$	9.80	-7.83	-8.15	-10.62	0.07	0.06	0.03
triazole	$C_2H_3N_3$	9.80	-7.59	-7.89	-10.21	0.08	0.07	0.02

3.2.2 Evaluation of mean absolute deviation in a grid of C_{OS} and C_{SS} values

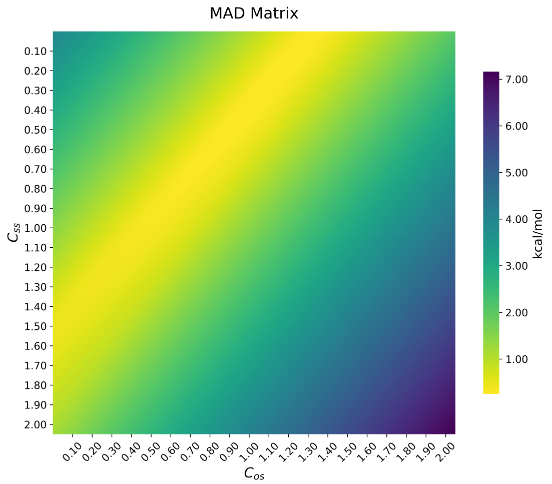

(a)

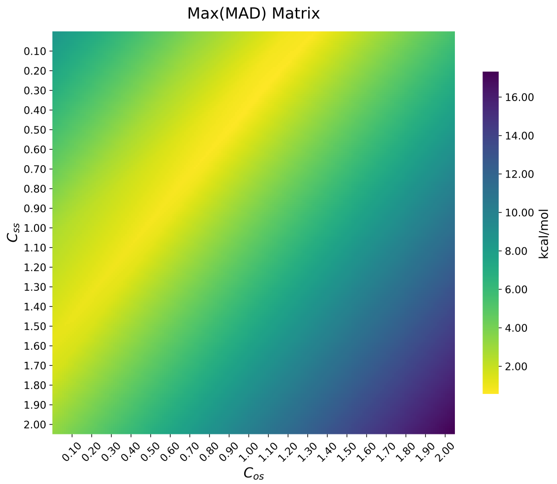

(b)

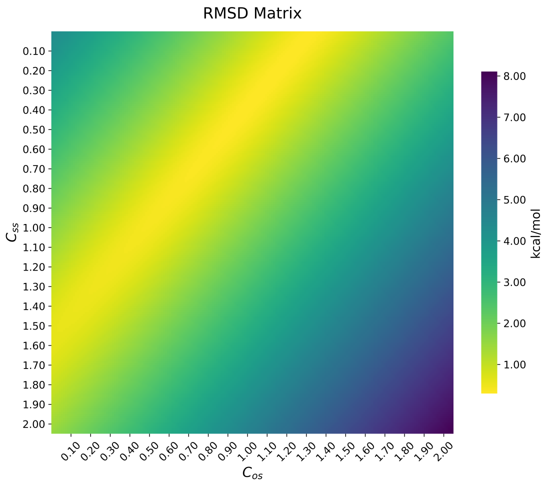

(c)

Figure S5: Evaluation of MADs (S5a), Max(MADs) (S5b) and RMSDs (S5c) in grid of values C_{OS} and C_{SS}. The optimal coefficients are C_{OS}=0.27 and C_{SS}=1.38. The theory level used is RIJK-MP2/aug-cc-pVDZ.

3.2.3 Interaction energies

MP2 with C_{OS} y C_{SS} values

Table S24: Interaction energies for CCSD(T)/CBS, MP2, SCS-MP2, SCS(MI)-MP2, SCSN-MP2, SCS-MP2-vdW, RI-MP2, RI-SCS-MP2, RIJK-MP2, RIJK-SCS-MP2, RIJCOSX-MP2, RIJCOSX-SCS-MP2, SCS-MP2^{BWI-TZ}, RI-SCS-MP2^{BWI-TZ}, RIJK-SCS-MP2^{BWI-TZ}, RIJCOSX-SCS-MP2^{BWI-DZ} and SCS-MP2-hal^{G-XZ}. Data: 50 interaction energies. MP2: $C_{OS} = C_{SS}$ =1.00; SCS-MP2: C_{OS} =1.20, C_{SS} =0.33; SCS(MI)-MP2: C_{OS} =0.40, C_{SS} =1.29; SCSN-MP2: C_{OS} =0.00, C_{SS} =1.76; SCS-MP2-vdW: C_{OS} =1.28, C_{SS} =0.50; SCS-MP2^{BWI-TZ} RI-SCS-MP2^{BWI-TZ} , RIJK-SCS-MP2^{BWI-TZ} : C_{OS} =0.27, C_{SS} =1.38; RIJCOSX-SCS-MP2^{BWI-TZ} : C_{OS} =0.17, C_{SS} =1.59. All values in kcal/mol.

Dimer	Formula	CCSD(T)/CBS	MP2	SCS-MP2	SCS(MI)-MP2	SCSN-MP2	SCS-MP2-vdW	RI-MP2	RI-SCS-MP2	RIJK-MP2	RIJK-SCS-MP2	RIJCOSX-MP2	RIJCOSX-SCS-MP2	SCS-MP2^{BWI-TZ}	RI-SCS-MP2^{BWI-TZ}	RIJK-SCS-MP2^{BWI-TZ}	RIJCOSX-SCS-MP2^{BWI-TZ}	SCS-MP2-hal^{G-XZ}
1 − 2 − 4 − triazole − thiophene	$C_2H_3N_3$	-2.98	-4.60	-3.60	-3.65	-3.65	-4.20	-4.60	-3.60	-4.60	-3.60	-4.58	-3.58	-3.51	-3.51	-3.51	-3.50	-3.30
1 − 3 − 5 − triazine − phosphine	$C_3H_3N_3$	-1.94	-2.83	-2.02	-2.26	-2.37	-2.46	-2.83	-2.02	-2.83	-2.01	-2.83	-2.01	-2.18	-2.18	-2.18	-2.20	-1.79
1 − 3 − 5 − triazine − phosphorine	$C_3H_3N_3$	-4.04	-7.05	-5.06	-5.45	-5.61	-6.19	-7.05	-5.06	-7.06	-5.07	-7.03	-5.03	-5.23	-5.23	-5.24	-5.24	-4.48
1 − 3 − 5 − triazine − thiophene	$C_3H_3N_3$	-3.41	-5.09	-4.03	-4.18	-4.23	-4.64	-5.09	-4.03	-5.09	-4.03	-5.04	-3.99	-4.05	-4.05	-4.05	-4.03	-3.72
2 − 3 − dimethylbutane − diphospine	C_6H_{14}	-2.74	-4.05	-3.02	-2.44	-2.13	-3.80	-4.05	-3.02	-4.05	-3.03	-4.00	-2.98	-2.17	-2.17	-2.17	-2.14	-2.67
acetone − phosphorine	C_3H_6O	-4.51	-6.56	-5.04	-5.32	-5.42	-5.91	-6.56	-5.04	-6.56	-5.05	-6.51	-5.00	-5.14	-5.14	-5.14	-5.13	-4.61
ammonia − phosphorine	NH_3	-2.23	-3.12	-2.39	-2.52	-2.57	-2.81	-3.12	-2.39	-3.12	-2.39	-3.08	-2.35	-2.44	-2.44	-2.44	-2.42	-2.18
ammonia − thiophene	NH_3	-2.28	-2.94	-2.47	-2.37	-2.31	-2.79	-2.94	-2.47	-2.94	-2.47	-2.92	-2.45	-2.28	-2.28	-2.28	-2.26	-2.32
benzene − carbondisulfide	C_6H_6	-3.03	-5.27	-3.70	-4.08	-4.23	-4.58	-5.27	-3.70	-5.26	-3.69	-5.25	-3.68	-3.91	-3.91	-3.91	-3.92	-3.26
benzene − phosphorine	C_6H_6	-3.48	-5.75	-4.36	-4.62	-4.72	-5.15	-5.75	-4.36	-5.77	-4.37	-5.71	-4.31	-4.46	-4.46	-4.47	-4.44	-3.96
butadinene − thiophene	C_4H_6	-2.58	-4.55	-3.27	-3.24	-3.19	-4.07	-4.55	-3.27	-4.55	-3.27	-4.52	-3.24	-3.03	-3.03	-3.04	-3.04	-2.88
carbondioxide − phosphorine	CO_2	-2.52	-3.27	-2.44	-3.24	-3.63	-2.75	-3.27	-2.44	-3.26	-2.44	-3.26	-2.44	-3.28	-3.28	-3.27	-3.29	-2.25
carbondioxide − thiophene	CO_2	-2.62	-2.96	-2.49	-3.03	-3.30	-2.64	-2.96	-2.49	-2.95	-2.48	-2.96	-2.49	-3.07	-3.07	-3.06	-3.08	-2.39
cyclobutadiene − dithiethane	C_4H_4	-4.22	-7.35	-5.07	-5.48	-5.63	-6.38	-7.35	-5.07	-7.34	-5.06	-7.31	-5.03	-5.21	-5.21	-5.20	-5.20	-4.41
cyclobutadiene − phosphorine	C_4H_4	-2.85	-4.85	-3.46	-3.89	-4.07	-4.21	-4.85	-3.46	-4.85	-3.46	-4.82	-3.43	-3.76	-3.76	-3.76	-3.75	-3.08
cyclohexane − diphosphine	C_6H_{12}	-2.35	-3.22	-2.53	-2.07	-1.82	-3.07	-3.22	-2.53	-3.23	-2.53	-3.20	-2.51	-1.87	-1.87	-1.87	-1.86	-2.28
cyclopentadiene − thiophene	C_5H_6	-2.38	-3.66	-3.01	-2.87	-2.79	-3.45	-3.66	-3.01	-3.67	-3.02	-3.62	-2.97	-2.74	-2.74	-2.75	-2.71	-2.80
cyclopentane − diphosphine	C_5H_{10}	-2.34	-3.09	-2.42	-2.00	-1.77	-2.94	-3.09	-2.42	-3.09	-2.43	-3.07	-2.40	-1.81	-1.81	-1.81	-1.80	-2.19
cyclopentane − phosphine	C_5H_{10}	-1.80	-2.42	-1.78	-1.43	-1.25	-2.26	-2.42	-1.78	-2.42	-1.78	-2.42	-1.78	-1.27	-1.27	-1.27	-1.28	-1.56
cyclopentane − phosphorus	C_5H_{10}	-3.25	-5.64	-4.12	-3.88	-3.72	-5.13	-5.64	-4.12	-5.66	-4.14	-5.60	-4.09	-3.59	-3.59	-3.61	-3.58	-3.65
diazomethane − carbondisulfide	CH_4	-2.18	-2.86	-1.96	-2.72	-3.08	-2.32	-2.86	-1.96	-2.84	-1.94	-2.86	-1.96	-2.73	-2.73	-2.71	-2.76	-1.74
diazomethane − diphosphine	CH_4	-2.96	-3.63	-2.70	-3.17	-3.39	-3.16	-3.63	-2.70	-3.61	-2.69	-3.62	-2.70	-3.12	-3.12	-3.11	-3.14	-2.46
diazomethane − phosphine	CH_4	-1.82	-1.96	-1.47	-1.98	-2.23	-1.64	-1.96	-1.47	-1.95	-1.46	-1.95	-1.46	-2.01	-2.01	-2.00	-2.01	-1.36
diazomethane − phosphorine	CH_4	-3.46	-4.93	-3.70	-4.32	-4.60	-4.30	-4.93	-3.70	-4.93	-3.71	-4.92	-3.69	-4.25	-4.25	-4.26	-4.27	-3.38
dimethylether − dimethyldisulfide	C_3H_6O	-3.75	-4.48	-3.64	-3.42	-3.28	-4.22	-4.48	-3.64	-4.48	-3.65	-4.46	-3.62	-3.24	-3.24	-3.25	-3.24	-3.37
dimethylether − thiophene	C_3H_6O	-2.82	-3.25	-2.90	-2.74	-2.66	-3.15	-3.25	-2.90	-3.25	-2.91	-3.19	-2.85	-2.66	-2.66	-2.66	-2.61	-2.78
dimethylperoxide − dimethylsulfide	$C_3H_6O_2$	-3.41	-3.98	-3.25	-3.10	-3.00	-3.74	-3.98	-3.25	-3.98	-3.25	-3.95	-3.22	-2.95	-2.95	-2.96	-2.94	-3.01
dimethylperoxide − thiophene	$C_3H_6O_2$	-2.35	-3.41	-2.91	-2.69	-2.56	-3.28	-3.41	-2.91	-3.41	-2.92	-3.38	-2.88	-2.56	-2.56	-2.57	-2.53	-2.75
ethane − dimethyldisulfide	C_2H_6	-2.16	-2.89	-2.18	-1.90	-1.75	-2.69	-2.89	-2.18	-2.89	-2.18	-2.86	-2.15	-1.74	-1.74	-1.73	-1.72	-1.94
ethene − thiophene	C_2H_4	-2.18	-3.25	-2.59	-2.51	-2.45	-3.02	-3.25	-2.59	-3.25	-2.59	-3.20	-2.55	-2.39	-2.39	-2.39	-2.36	-2.38
formaldehyde − phosphorine	CH_2O	-3.18	-4.52	-3.22	-3.80	-4.06	-3.88	-4.52	-3.22	-4.51	-3.22	-4.50	-3.21	-3.72	-3.72	-3.71	-3.73	-2.88
formaldehyde − thiophene	CH_2O	-3.04	-3.73	-3.09	-3.37	-3.50	-3.42	-3.73	-3.09	-3.73	-3.09	-3.70	-3.07	-3.33	-3.33	-3.33	-3.32	-2.92
hexane − carbondisulfide	C_6H_{14}	-3.26	-5.00	-3.63	-3.51	-3.42	-4.51	-5.00	-3.63	-5.00	-3.63	-4.97	-3.60	-3.28	-3.28	-3.28	-3.28	-3.20
methylamine − thiophene	CH_3NH_2	-3.15	-4.43	-3.62	-3.47	-3.38	-4.16	-4.43	-3.62	-4.44	-3.63	-4.39	-3.58	-3.32	-3.32	-3.33	-3.30	-3.37
methylazide − thiophene	CH_3N_3	-4.20	-5.32	-4.39	-5.29	-5.73	-4.73	-5.32	-4.39	-5.32	-4.39	-5.28	-4.36	-5.33	-5.33	-5.34	-5.33	-4.17
naphthalene − carbondisulfide	$C_{10}H_8$	-3.25	-5.73	-4.30	-4.77	-4.97	-5.06	-5.73	-4.30	-5.74	-4.30	-5.71	-4.27	-4.64	-4.64	-4.64	-4.64	-3.90
naphthalene − dimethyldisulfide	$C_{10}H_8$	-5.03	-8.65	-6.39	-6.38	-6.32	-7.79	-8.65	-6.39	-8.67	-6.41	-8.56	-6.31	-6.03	-6.03	-6.05	-5.99	-5.71
naphthalene − diphospine	$C_{10}H_8$	-5.13	-8.37	-6.26	-6.38	-6.39	-7.53	-8.37	-6.26	-8.38	-6.26	-8.32	-6.21	-6.08	-6.08	-6.08	-6.08	-5.63
naphthalene − thioacetone	$C_{10}H_8$	-6.35	-10.22	-7.81	-8.00	-8.04	-9.25	-10.22	-7.81	-10.24	-7.83	-10.14	-7.74	-7.67	-7.67	-7.68	-7.65	-7.09
neohexane − diphospine	C_6H_{14}	-2.70	-3.66	-2.86	-2.33	-2.05	-3.49	-3.66	-2.86	-3.66	-2.86	-3.63	-2.83	-2.11	-2.11	-2.10	-2.08	-2.57
neopentane − phospine	C_5H_{12}	-1.28	-1.79	-1.37	-0.98	-0.78	-1.73	-1.79	-1.37	-1.79	-1.37	-1.77	-1.35	-0.84	-0.84	-0.84	-0.83	-1.22
nonbornadiene − dimethyldisulfide	C_7H_8	-3.20	-4.29	-3.46	-3.31	-3.21	-4.02	-4.29	-3.46	-4.30	-3.47	-4.20	-3.36	-3.15	-3.15	-3.15	-3.07	-3.20
propane − carbondisulfide	C_3H_8	-2.26	-3.39	-2.45	-2.45	-2.43	-3.03	-3.39	-2.45	-3.38	-2.45	-3.37	-2.44	-2.31	-2.31	-2.30	-2.31	-2.17
propane − diphospine	C_3H_8	-2.35	-3.27	-2.39	-2.07	-1.89	-3.02	-3.27	-2.39	-3.27	-2.39	-3.25	-2.38	-1.87	-1.87	-1.87	-1.87	-2.10
propane − phosphorine	C_3H_8	-2.92	-4.83	-3.51	-3.43	-3.35	-4.35	-4.83	-3.51	-4.84	-3.53	-4.76	-3.45	-3.21	-3.21	-3.22	-3.17	-3.11
propane − phospine	C_3H_8	-1.37	-1.87	-1.35	-1.11	-0.98	-1.73	-1.87	-1.35	-1.87	-1.35	-1.86	-1.34	-0.99	-0.99	-0.99	-0.98	-1.18
propene − thiophene	C_3H_8	-2.61	-3.94	-3.14	-3.04	-2.97	-3.66	-3.94	-3.14	-3.95	-3.15	-3.90	-3.10	-2.90	-2.90	-2.91	-2.87	-2.89
toluene − dimethylsulfide	$C_6H_5CH_3$	-4.29	-6.29	-4.83	-4.86	-4.84	-5.73	-6.29	-4.83	-6.30	-4.84	-6.23	-4.77	-4.64	-4.64	-4.65	-4.61	-4.39
toluene − diphospine	$C_6H_5CH_3$	-4.95	-7.58	-5.64	-5.67	-5.63	-6.84	-7.58	-5.64	-7.59	-5.65	-7.52	-5.58	-5.37	-5.37	-5.38	-5.35	-5.06
toluene − phosphorine	$C_6H_5CH_3$	-4.04	-8.20	-5.69	-5.77	-5.75	-7.22	-8.20	-5.69	-8.21	-5.70	-8.15	-5.65	-5.40	-5.40	-5.41	-5.40	-4.94

DFT

Table S25: Interaction energies for CCSD(T)/CBS B97M-V, ωB97X-V, ωB97M-V, ωB97X-D3, B2PLYP-D3BJ, DSD-BLYP-D3BJ, ωB97X-D4, B2PLYP-D4. Data: 50 interaction energies. All values in kcal/mol.

Dimer	Formula	CCSD(T)/CBS	B97M-V	ωB97X-V	ωB97M-V	ωB97X-D3	B2PLYP-D3BJ	DSD-BLYP-D3BJ	ωB97X-D4	B2PLYP-D4
$1-2-4-triazole-thiophene$	$C_2H_3N_3-C_4H_4S$	-2.98	-3.28	-3.48	-3.73	-3.64	-3.47	-3.63	-3.91	-3.65
$1-3-5-triazine-phosphine$	$C_3H_3N_3-PH_3$	-1.94	-1.88	-1.96	-2.03	-2.06	-2.13	-2.22	-2.56	-2.11
$1-3-5-triazine-phosphorine$	$C_3H_3N_3-PC_5H_5$	-4.04	-4.24	-4.20	-4.54	-4.65	-4.71	-4.96	-4.62	-4.86
$1-3-5-triazine-thiophene$	$C_3H_3N_3-C_4H_4S$	-3.41	-3.58	-3.71	-3.97	-4.00	-3.90	-4.07	-4.18	-3.92
$2-3-dimethylbutane-diphospine$	$C_6H_{14}-P_2H_4$	-2.74	-2.63	-2.71	-2.72	-2.88	-3.18	-3.12	-3.43	-3.01
$acetone-phosphorine$	$C_3H_6O-PC_5H_5$	-4.51	-4.72	-4.86	-5.07	-5.27	-4.93	-5.11	-5.41	-4.86
$ammonia-phosphorine$	$NH_3-PC_5H_5$	-2.23	-2.21	-2.42	-2.52	-2.77	-2.40	-2.50	-2.98	-2.31
$ammonia-thiophene$	$NH_3-C_4H_4S$	-2.28	-2.38	-2.59	-2.68	-2.94	-2.57	-2.60	-3.15	-2.49
$benzene-carbondisulfide$	$C_6H_6-CS_2$	-3.03	-2.76	-2.85	-3.14	-3.42	-3.38	-3.62	-3.56	-3.25
$benzene-phosphorine$	$C_6H_6-PC_5H_5$	-3.48	-3.57	-3.52	-3.84	-3.95	-4.02	-4.26	-4.26	-3.97
$butadinene-thiophene$	$C_4H_6-C_4H_4S$	-2.58	-2.94	-2.89	-3.27	-3.48	-3.19	-3.30	-3.56	-3.21
$carbondioxide-phosphorine$	$CO_2-PC_5H_5$	-2.52	-2.88	-2.91	-3.13	-3.07	-2.70	-2.82	-3.27	-2.64
$carbondioxide-thiophene$	$CO_2-C_4H_4S$	-2.62	-3.04	-3.13	-3.31	-3.09	-2.85	-2.92	-3.49	-2.79
$cyclobutadiene-dithiethane$	$C_4H_4-C_2H_4S_2$	-4.22	-5.70	-4.14	-4.84	-4.26	-5.36	-5.51	-4.68	-5.31
$cyclobutadiene-phosphorine$	$C_4H_4-PC_5H_5$	-2.85	-3.72	-3.10	-3.44	-3.25	-3.53	-3.72	-4.02	-3.52
$cyclohexane-diphosphine$	$C_6H_{12}-P_2H_4$	-2.35	-1.96	-2.21	-2.17	-2.16	-2.59	-2.58	-2.76	-2.48
$cyclopentadiene-thiophene$	$C_5H_6-C_4H_4S$	-2.38	-2.63	-2.78	-2.96	-3.07	-2.86	-2.97	-3.33	-2.82
$cyclopentane-diphosphine$	$C_5H_{10}-P_2H_4$	-2.34	-1.93	-2.19	-2.11	-2.12	-2.53	-2.52	-2.78	-2.42
$cyclopentane-phosphine$	$C_5H_{10}-PH_3$	-1.80	-1.68	-1.86	-1.77	-1.89	-2.05	-1.99	-2.52	-1.96
$cyclopentane-phosphorus$	$C_5H_{10}-P_4$	-3.25	-3.21	-3.53	-3.40	-3.44	-4.08	-4.18	-4.27	-3.88
$diazomethane-carbondisulfide$	CH_4-CS_2	-2.18	-2.20	-2.31	-2.38	-2.50	-2.21	-2.30	-2.97	-2.14
$diazomethane-diphosphine$	$CH_4-P_2H_4$	-2.96	-2.89	-2.91	-2.95	-2.83	-3.06	-3.11	-3.70	-2.92
$diazomethane-phosphine$	CH_4-PH_3	-1.82	-1.96	-2.01	-1.94	-1.98	-1.90	-1.89	-2.58	-1.82
$diazomethane-phosphorine$	$CH_4-PC_5H_5$	-3.46	-3.59	-3.67	-3.88	-4.07	-3.76	-3.91	-4.28	-3.62
$dimethylether-dimethyldisulfide$	$C_3H_6O-CH_3S_2CH_3$	-3.75	-3.48	-3.68	-3.57	-3.88	-4.00	-3.92	-4.24	-4.01
$dimethylether-thiophene$	$C_3H_6O-C_4H_4S$	-2.82	-2.65	-3.02	-2.98	-3.05	-2.94	-2.99	-3.37	-2.99
$dimethylperoxide-dimethylsulfide$	$C_3H_6O_2-CH_3SCH_3$	-3.41	-3.19	-3.50	-3.34	-3.60	-3.53	-3.50	-4.04	-3.50
$dimethylperoxide-thiophene$	$C_3H_6O_2-C_4H_4S$	-2.35	-2.73	-2.79	-2.88	-2.99	-3.01	-3.01	-3.30	-2.94
$ethane-dimethyldisulfide$	$C_2H_6-CH_3S_2CH_3$	-2.16	-2.04	-2.08	-2.04	-2.27	-2.36	-2.32	-2.60	-2.28
$ethene-thiophene$	$C_2H_4-C_4H_4S$	-2.18	-2.43	-2.49	-2.65	-2.81	-2.57	-2.64	-3.10	-2.52
$formaldehyde-phosphorine$	$CH_2O-PC_5H_5$	-3.18	-3.85	-3.39	-3.69	-3.59	-3.57	-3.68	-3.98	-3.49
$formaldehyde-thiophene$	$CH_2O-C_4H_4S$	-3.04	-3.41	-3.40	-3.60	-3.61	-3.41	-3.47	-3.99	-3.24
$hexane-carbondisulfide$	$C_6H_{14}-CS_2$	-3.26	-2.92	-3.21	-3.22	-3.58	-3.51	-3.64	-3.90	-3.16
$methylamine-thiophene$	$CH_3NH_2-C_4H_4S$	-3.15	-3.28	-3.51	-3.68	-3.85	-3.61	-3.70	-4.01	-3.52
$methylazide-thiophene$	$CH_3N_3-C_4H_4S$	-4.20	-4.60	-5.09	-5.26	-5.52	-4.53	-4.68	-5.81	-4.41
$naphthalene-carbondisulfide$	$C_{10}H_8-CS_2$	-3.25	-3.21	-3.23	-3.52	-3.52	-3.85	-4.20	-3.81	-3.69
$naphthalene-dimethyldisulfide$	$C_{10}H_8-CH_3S_3CH_3$	-5.03	-5.14	-5.04	-5.59	-5.69	-6.05	-6.27	-5.50	-5.97
$naphthalene-diphospine$	$C_{10}H_8-PSH_4$	-5.13	-4.93	-5.06	-5.54	-5.64	-5.97	-6.21	-5.71	-5.76
$naphthalene-thioacetone$	$C_{10}H_8-C_3H_6S$	-6.35	-6.77	-6.80	-7.28	-7.41	-7.31	-7.65	-7.24	-7.11
$neohexane-diphospine$	$C_6H_{14}-PSH_4$	-2.70	-2.38	-2.61	-2.54	-2.60	-2.92	-2.90	-3.26	-2.76
$neopentane-phospine$	$C_5H_{12}-PH_3$	-1.28	-1.32	-1.40	-1.27	-1.45	-1.46	-1.41	-1.94	-1.39
$nonbornadiene-dimethyldisulfide$	$C_7H_8-CH_3S_2CH_3$	-3.20	-3.28	-3.34	-3.44	-3.75	-3.71	-3.65	-3.83	-3.63
$propane-carbondisulfide$	$C_3H_8-CS_2$	-2.26	-2.03	-2.29	-2.27	-2.56	-2.36	-2.47	-2.89	-2.12
$propane-diphospine$	$C_3H_8-P_2H_4$	-2.35	-2.08	-2.21	-2.15	-2.21	-2.59	-2.57	-2.98	-2.44
$propane-phosphorine$	$C_3H_8-PC_5H_5$	-2.92	-3.04	-3.17	-3.37	-3.50	-3.31	-3.44	-3.69	-3.23
$propane-phospine$	$C_3H_8-PH_3$	-1.37	-1.29	-1.40	-1.30	-1.42	-1.51	-1.49	-1.98	-1.43
$propene-thiophene$	$C_3H_8-C_4H_4S$	-2.61	-2.82	-2.94	-3.10	-3.28	-3.11	-3.20	-3.50	-3.03
$toluene-dimethylsulfide$	$C_6H_5CH_3-CH_3SCH_3$	-4.29	-4.28	-4.26	-4.54	-4.63	-4.74	-4.89	-4.83	-4.64
$toluene-diphospine$	$C_6H_5CH_3-P_2H_4$	-4.95	-4.59	-4.61	-4.99	-4.82	-5.55	-5.75	-5.41	-5.38
$toluene-phosphorine$	$C_6H_5CH_3-PC_5H_5$	-4.04	-4.30	-4.11	-4.70	-4.79	-5.07	-5.37	-4.66	-5.12

SAPT-DFT

Table S26: Interaction energies for CCSD(T)/CBS and SAPT-DFT. For SAPT-DFT method we used the B3LYP, PBE0 and ωB97X DFA's in conjunction with the aug-cc-pVDZ basis set. Data: 50 interaction energies. All values in kcal/mol.

Dimer	Formula	CCSD(T)/CBS	SAPT-B3LYP	SAPT-PBE0	SAPT-ωB97X
$1-2-4-triazole-thiophene$	$C_2H_3N_3-C_4H_4S$	-2.98	-2.08	-2.00	-1.52
$1-3-5-triazine-phosphine$	$C_3H_3N_3-PH_3$	-1.94	-1.32	-1.28	-1.04
$1-3-5-triazine-phosphorine$	$C_3H_3N_3-PC_5H_5$	-4.04	-2.90	-2.85	-2.30
$1-3-5-triazine-thiophene$	$C_3H_3N_3-C_4H_4S$	-3.41	-2.42	-2.47	-2.13
$2-3-dimethylbutane-diphospine$	$C_6H_{14}-P_2H_4$	-2.74	-1.80	-1.72	-1.29
$acetone-phosphorine$	$C_3H_6O-PC_5H_5$	-4.51	-3.59	-3.66	-2.95
$ammonia-phosphorine$	$NH_3-PC_5H_5$	-2.23	-1.70	-1.75	-1.43
$ammonia-thiophene$	$NH_3-C_4H_4S$	-2.28	-1.76	-1.79	-1.46
$benzene-carbondisulfide$	$C_6H_6-CS_2$	-3.03	-2.19	-2.13	-1.55
$benzene-phosphorine$	$C_6H_6-PC_5H_5$	-3.48	-2.60	-2.68	-2.19
$butadinene-thiophene$	$C_4H_6-C_4H_4S$	-2.58	-1.87	-1.75	-1.07
$carbondioxide-phosphorine$	$CO_2-PC_5H_5$	-2.52	-1.85	-1.84	-1.45
$carbondioxide-thiophene$	$CO_2-C_4H_4S$	-2.62	-1.91	-1.91	-1.56
$cyclobutadiene-dithiethane$	$C_4H_4-C_2H_4S_2$	-4.22	-2.71	-2.78	-1.85
$cyclobutadiene-phosphorine$	$C_4H_4-PC_5H_5$	-2.85	-2.15	-2.17	-1.52
$cyclohexane-diphosphine$	$C_6H_{12}-P_2H_4$	-2.35	-1.65	-1.58	-1.23
$cyclopentadiene-thiophene$	$C_5H_6-C_4H_4S$	-2.38	-1.79	-1.80	-1.32
$cyclopentane-diphosphine$	$C_5H_{10}-P_2H_4$	-2.34	-1.66	-1.61	-1.29
$cyclopentane-phosphine$	$C_5H_{10}-PH_3$	-1.80	-1.26	-1.23	-0.88
$cyclopentane-phosphorus$	$C_5H_{10}-P_4$	-3.25	-2.49	-2.44	-1.80
$diazomethane-carbondisulfide$	CH_4-CS_2	-2.18	-1.53	-1.50	-1.08
$diazomethane-diphosphine$	$CH_4-P_2H_4$	-2.96	-2.23	-2.21	-1.76
$diazomethane-phosphine$	CH_4-PH_3	-1.82	-1.43	-1.43	-1.17
$diazomethane-phosphorine$	$CH_4-PC_5H_5$	-3.46	-2.65	-2.74	-2.21
$dimethylether-dimethyldisulfide$	$C_3H_6O-CH_3S_2CH_3$	-3.75	-2.62	-2.66	-2.45
$dimethylether-thiophene$	$C_3H_6O-C_4H_4S$	-2.82	-2.19	-2.23	-1.85
$dimethylperoxide-dimethylsulfide$	$C_3H_6O_2-CH_3SCH_3$	-3.41	-2.64	-2.65	-2.19
$dimethylperoxide-thiophene$	$C_3H_6O_2-C_4H_4S$	-2.35	-1.77	-1.78	-1.50
$ethane-dimethyldisulfide$	$C_2H_6-CH_3S_2CH_3$	-2.16	-1.41	-1.38	-1.28
$ethene-thiophene$	$C_2H_4-C_4H_4S$	-2.18	-1.70	-1.71	-1.25
$formaldehyde-phosphorine$	$CH_2O-PC_5H_5$	-3.18	-2.52	-2.56	-1.93
$formaldehyde-thiophene$	$CH_2O-C_4H_4S$	-3.04	-2.37	-2.44	-2.06
$hexane-carbondisulfide$	$C_6H_{14}-CS_2$	-3.26	-2.35	-2.28	-1.64
$methylamine-thiophene$	$CH_3NH_2-C_4H_4S$	-3.15	-2.37	-2.44	-2.02
$methylazide-thiophene$	$CH_3N_3-C_4H_4S$	-4.20	-3.21	-3.27	-2.64
$naphthalene-carbondisulfide$	$C_{10}H_8-CS_2$	-3.25	-2.45	-2.49	-1.99
$naphthalene-dimethyldisulfide$	$C_{10}H_8-CH_3S_3CH_3$	-5.03	-3.23	-3.19	-2.99
$naphthalene-diphospine$	$C_{10}H_8-PSH_4$	-5.13	-3.76	-3.81	-3.06
$naphthalene-thioacetone$	$C_{10}H_8-C_3H_6S$	-6.35	-4.99	-5.12	-4.11
$neohexane-diphospine$	$C_6H_{14}-PSH_4$	-2.70	-1.87	-1.80	-1.44
$neopentane-phospine$	$C_5H_{12}-PH_3$	-1.28	-0.93	-0.88	-0.57
$nonbornadiene-dimethyldisulfide$	$C_7H_8-CH_3S_2CH_3$	-3.20	-2.20	-2.29	-2.22
$propane-carbondisulfide$	$C_3H_8-CS_2$	-2.26	-1.61	-1.55	-1.12
$propane-diphospine$	$C_3H_8-P_2H_4$	-2.35	-1.63	-1.61	-1.18
$propane-phosphorine$	$C_3H_8-PC_5H_5$	-2.92	-2.16	-2.14	-1.62
$propane-phospine$	$C_3H_8-PH_3$	-1.37	-0.98	-0.94	-0.66
$propene-thiophene$	$C_3H_8-C_4H_4S$	-2.61	-1.92	-1.94	-1.56
$toluene-dimethylsulfide$	$C_6H_5CH_3-CH_3SCH_3$	-4.29	-3.34	-3.42	-2.65
$toluene-diphospine$	$C_6H_5CH_3-P_2H_4$	-4.95	-3.72	-3.71	-2.97
$toluene-phosphorine$	$C_6H_5CH_3-PC_5H_5$	-4.04	-2.95	-2.82	-1.89

3.2.4 Absolute deviations of molecular systems.

MP2 with C_{OS} y C_{SS} values

Table S27: Absolute deviations for MP2, SCS-MP2, SCS(MI)-MP2, SCSN-MP2, SCS-MP2-vdW, RI-MP2, RI-SCS-MP2, RIJK-MP2, RIJK-SCS-MP2, RIJCOSX-MP2, RIJCOSX-SCS-MP2, SCS-MP2^{BWI-TZ}, RI-SCS-MP2^{BWI-TZ}, RIJK-SCS-MP2^{BWI-TZ}, RIJCOSX-SCS-MP2^{BWI-DZ} and SCS-MP2-hal^{G-XZ}. Data: 50 interaction energies. MP2: $C_{OS} = C_{SS}$ =1.00; SCS-MP2: C_{OS} =1.20, C_{SS} =0.33; SCS(MI)-MP2: C_{OS} =0.40, C_{SS} =1.29; SCSN-MP2: C_{OS} =0.00, C_{SS} =1.76; SCS-MP2-vdW: C_{OS} =1.28, C_{SS} =0.50; SCS-MP2^{BWI-TZ} RI-SCS-MP2^{BWI-TZ} , RIJK-SCS-MP2^{BWI-TZ} : C_{OS} =0.27, C_{SS} =1.38; RIJCOSX-SCS-MP2^{BWI-TZ} : C_{OS} =0.17, C_{SS} =1.59. All values in kcal/mol.

Dimer	Formula	MP2	SCS-MP2	SCS(MI)-MP2	SCSN-MP2	SCS-MP2-vdW	RI-MP2	RI-SCS-MP2	RIJK-MP2	RIJK-SCS-MP2	RIJCOSX-MP2	RIJCOSX-SCS-MP2	SCS-MP2^{BWI-TZ}	RI-SCS-MP2^{BWI-TZ}	RIJK-SCS-MP2^{BWI-TZ}	RIJCOSX-SCS-MP2^{BWI-TZ}	SCS-MP2-hal^{G-XZ}
1 − 2 − 4 − triazole − thiophene	$C_2H_3N_3$	1.62	0.62	0.67	0.67	1.22	1.62	0.62	1.62	0.62	1.60	0.60	0.53	0.53	0.53	0.52	0.32
1 − 3 − 5 − triazine − phosphine	$C_3H_3N_3$	0.89	0.08	0.32	0.43	0.52	0.89	0.08	0.89	0.07	0.89	0.07	0.24	0.24	0.24	0.26	0.15
1 − 3 − 5 − triazine − phosphorine	$C_3H_3N_3$	3.01	1.02	1.41	1.57	2.15	3.01	1.02	3.02	1.03	2.99	0.99	1.19	1.19	1.20	1.20	0.44
1 − 3 − 5 − triazine − thiophene	$C_3H_3N_3$	1.68	0.62	0.77	0.82	1.23	1.68	0.62	1.68	0.62	1.63	0.58	0.64	0.64	0.64	0.62	0.31
2 − 3 − dimethylbutane − diphospine	C_6H_{14}	1.31	0.28	0.30	0.61	1.06	1.31	0.28	1.31	0.29	1.26	0.24	0.57	0.57	0.57	0.60	0.07
acetone − phosphorine	C_3H_6O	2.05	0.53	0.81	0.91	1.40	2.05	0.53	2.05	0.54	2.00	0.49	0.63	0.63	0.63	0.62	0.10
ammonia − phosphorine	NH_3	0.89	0.16	0.29	0.34	0.58	0.89	0.16	0.89	0.16	0.85	0.12	0.21	0.21	0.21	0.19	0.05
ammonia − thiophene	NH_3	0.66	0.19	0.09	0.03	0.51	0.66	0.19	0.66	0.19	0.64	0.17	0.00	0.00	0.00	0.02	0.04
benzene − carbondisulfide	C_6H_6	2.24	0.67	1.05	1.20	1.55	2.24	0.67	2.23	0.66	2.22	0.65	0.88	0.88	0.88	0.89	0.23
benzene − phosphorine	C_6H_6	2.27	0.88	1.14	1.24	1.67	2.27	0.88	2.29	0.89	2.23	0.83	0.98	0.98	0.99	0.96	0.48
butadinene − thiophene	C_4H_6	1.97	0.69	0.66	0.61	1.49	1.97	0.69	1.97	0.69	1.94	0.66	0.45	0.45	0.46	0.46	0.30
carbondioxide − phosphorine	CO_2	0.75	0.08	0.72	1.11	0.23	0.75	0.08	0.74	0.08	0.74	0.08	0.76	0.76	0.75	0.77	0.27
carbondioxide − thiophene	CO_2	0.34	0.13	0.41	0.68	0.02	0.34	0.13	0.33	0.14	0.34	0.13	0.45	0.45	0.44	0.46	0.23
cyclobutadiene − dithiethane	C_4H_4	3.13	0.85	1.26	1.41	2.16	3.13	0.85	3.12	0.84	3.09	0.81	0.99	0.99	0.98	0.98	0.19
cyclobutadiene − phosphorine	C_4H_4	2.00	0.61	1.04	1.22	1.36	2.00	0.61	2.00	0.61	1.97	0.58	0.91	0.91	0.91	0.90	0.23
cyclohexane − diphosphine	C_6H_{12}	0.87	0.18	0.28	0.53	0.72	0.87	0.18	0.88	0.18	0.85	0.16	0.48	0.48	0.48	0.49	0.07
cyclopentadiene − thiophene	C_5H_6	1.28	0.63	0.49	0.41	1.07	1.28	0.63	1.29	0.64	1.24	0.59	0.36	0.36	0.37	0.33	0.42
cyclopentane − diphosphine	C_5H_{10}	0.75	0.08	0.34	0.57	0.60	0.75	0.08	0.75	0.09	0.73	0.06	0.53	0.53	0.53	0.54	0.15
cyclopentane − phosphine	C_5H_{10}	0.62	0.02	0.37	0.55	0.46	0.62	0.02	0.62	0.02	0.62	0.02	0.53	0.53	0.53	0.52	0.24
cyclopentane − phosphorus	C_5H_{10}	2.39	0.87	0.63	0.47	1.88	2.39	0.87	2.41	0.89	2.35	0.84	0.34	0.34	0.36	0.33	0.40
diazomethane − carbondisulfide	CH_4	0.68	0.22	0.54	0.90	0.14	0.68	0.22	0.66	0.24	0.68	0.22	0.55	0.55	0.53	0.58	0.44
diazomethane − diphosphine	CH_4	0.67	0.26	0.21	0.43	0.20	0.67	0.26	0.65	0.27	0.66	0.26	0.16	0.16	0.15	0.18	0.50
diazomethane − phosphine	CH_4	0.14	0.35	0.16	0.41	0.18	0.14	0.35	0.13	0.36	0.13	0.36	0.19	0.19	0.18	0.19	0.46
diazomethane − phosphorine	CH_4	1.47	0.24	0.86	1.14	0.84	1.47	0.24	1.47	0.25	1.46	0.23	0.79	0.79	0.80	0.81	0.08
dimethylether − dimethyldisulfide	C_3H_6O	0.73	0.11	0.33	0.47	0.47	0.73	0.11	0.73	0.10	0.71	0.13	0.51	0.51	0.50	0.51	0.38
dimethylether − thiophene	C_3H_6O	0.43	0.08	0.08	0.16	0.33	0.43	0.08	0.43	0.09	0.37	0.03	0.16	0.16	0.16	0.21	0.04
dimethylperoxide − dimethylsulfide	$C_3H_6O_2$	0.57	0.16	0.31	0.41	0.33	0.57	0.16	0.57	0.16	0.54	0.19	0.46	0.46	0.45	0.47	0.40
dimethylperoxide − thiophene	$C_3H_6O_2$	1.06	0.56	0.34	0.21	0.93	1.06	0.56	1.06	0.57	1.03	0.53	0.21	0.21	0.22	0.18	0.40
ethane − dimethyldisulfide	C_2H_6	0.73	0.02	0.26	0.41	0.53	0.73	0.02	0.73	0.02	0.70	0.01	0.42	0.42	0.43	0.44	0.22
ethene − thiophene	C_2H_4	1.07	0.41	0.33	0.27	0.84	1.07	0.41	1.07	0.41	1.02	0.37	0.21	0.21	0.21	0.18	0.20
formaldehyde − phosphorine	CH_2O	1.34	0.04	0.62	0.88	0.70	1.34	0.04	1.33	0.04	1.32	0.03	0.54	0.54	0.53	0.55	0.30
formaldehyde − thiophene	CH_2O	0.69	0.05	0.33	0.46	0.38	0.69	0.05	0.69	0.05	0.66	0.03	0.29	0.29	0.29	0.28	0.12
hexane − carbondisulfide	C_6H_{14}	1.74	0.37	0.25	0.16	1.25	1.74	0.37	1.74	0.37	1.71	0.34	0.02	0.02	0.02	0.02	0.06
methylamine − thiophene	CH_3NH_2	1.28	0.47	0.32	0.23	1.01	1.28	0.47	1.29	0.48	1.24	0.43	0.17	0.17	0.18	0.15	0.22
methylazide − thiophene	CH_3N_3	1.12	0.19	1.09	1.53	0.53	1.12	0.19	1.12	0.19	1.08	0.16	1.13	1.13	1.14	1.13	0.03
naphthalene − carbondisulfide	$C_{10}H_8$	2.48	1.05	1.52	1.72	1.81	2.48	1.05	2.49	1.05	2.46	1.02	1.39	1.39	1.39	1.39	0.65
naphthalene − dimethyldisulfide	$C_{10}H_8$	3.62	1.36	1.35	1.29	2.76	3.62	1.36	3.64	1.38	3.53	1.28	1.00	1.00	1.02	0.96	0.68
naphthalene − diphospine	$C_{10}H_8$	3.24	1.13	1.25	1.26	2.40	3.24	1.13	3.25	1.13	3.19	1.08	0.95	0.95	0.95	0.95	0.50
naphthalene − thioacetone	$C_{10}H_8$	3.87	1.46	1.65	1.69	2.90	3.87	1.46	3.89	1.48	3.79	1.39	1.32	1.32	1.33	1.30	0.74
neohexane − diphospine	C_6H_{14}	0.96	0.16	0.37	0.65	0.79	0.96	0.16	0.96	0.16	0.93	0.13	0.59	0.59	0.60	0.62	0.13
neopentane − phospine	C_5H_{12}	0.51	0.09	0.30	0.50	0.45	0.51	0.09	0.51	0.09	0.49	0.07	0.44	0.44	0.44	0.45	0.06
nonbornadiene − dimethyldisulfide	C_7H_8	1.09	0.26	0.11	0.01	0.82	1.09	0.26	1.10	0.27	1.00	0.16	0.05	0.05	0.05	0.13	0.00
propane − carbondisulfide	C_3H_8	1.13	0.19	0.19	0.17	0.77	1.13	0.19	1.12	0.19	1.11	0.18	0.05	0.05	0.04	0.05	0.09
propane − diphospine	C_3H_8	0.92	0.04	0.28	0.46	0.67	0.92	0.04	0.92	0.04	0.90	0.03	0.48	0.48	0.48	0.48	0.25
propane − phosphorine	C_3H_8	1.91	0.59	0.51	0.43	1.43	1.91	0.59	1.92	0.61	1.84	0.53	0.29	0.29	0.30	0.25	0.19
propane − phospine	C_3H_8	0.50	0.02	0.26	0.39	0.36	0.50	0.02	0.50	0.02	0.49	0.03	0.38	0.38	0.38	0.39	0.19
propene − thiophene	C_3H_8	1.33	0.53	0.43	0.36	1.05	1.33	0.53	1.34	0.54	1.29	0.49	0.29	0.29	0.30	0.26	0.28
toluene − dimethylsulfide	$C_6H_5CH_3$	2.00	0.54	0.57	0.55	1.44	2.00	0.54	2.01	0.55	1.94	0.48	0.35	0.35	0.36	0.32	0.10
toluene − diphospine	$C_6H_5CH_3$	2.63	0.69	0.72	0.68	1.89	2.63	0.69	2.64	0.70	2.57	0.63	0.42	0.42	0.43	0.40	0.11
toluene − phosphorine	$C_6H_5CH_3$	4.16	1.65	1.73	1.71	3.18	4.16	1.65	4.17	1.66	4.11	1.61	1.36	1.36	1.37	1.36	0.90

DFT

Table S28: Absolute deviations for B97M-V, ωB97X-V, ωB97M-V, ωB97X-D3, B2PLYP-D3BJ and DSD-BLYP-D3BJ, ωB97X-D4 and B2PLYP-D4. Data: 50 interaction energies. All values in kcal/mol.

Dimer	Formula	B97M-V	ωB97X-V	ωB97M-V	ωB97X-D3	B2PLYP-D3BJ	DSD-BLYP-D3BJ	ωB97X-D4	B2PLYP-D4
1 − 2 − 4 − *triazole − thiophene*	$C_2H_3N_3-C_4H_4S$	0.30	0.50	0.75	0.66	0.49	0.65	0.93	0.67
1 − 3 − 5 − *triazine − phosphine*	$C_3H_3N_3-PH_3$	0.06	0.02	0.09	0.12	0.19	0.28	0.62	0.17
1 − 3 − 5 − *triazine − phosphorine*	$C_3H_3N_3-PC_5H_5$	0.20	0.16	0.50	0.61	0.67	0.92	0.58	0.82
1 − 3 − 5 − *triazine − thiophene*	$C_3H_3N_3-C_4H_4S$	0.17	0.30	0.56	0.59	0.49	0.66	0.77	0.51
2 − 3 − *dimethylbutane − diphospine*	$C_6H_{14}-P_2H_4$	0.11	0.03	0.02	0.14	0.44	0.38	0.69	0.27
acetone − phosphorine	$C_3H_6O-PC_5H_5$	0.21	0.35	0.56	0.76	0.42	0.60	0.90	0.35
ammonia − phosphorine	$NH_3-PC_5H_5$	0.02	0.19	0.29	0.54	0.17	0.27	0.75	0.08
ammonia − thiophene	$NH_3-C_4H_4S$	0.10	0.31	0.40	0.66	0.29	0.32	0.87	0.21
benzene − carbondisulfide	$C_6H_6-CS_2$	0.27	0.18	0.11	0.39	0.35	0.59	0.53	0.22
benzene − phosphorine	$C_6H_6-PC_5H_5$	0.09	0.04	0.36	0.47	0.54	0.78	0.78	0.49
butadinene − thiophene	$C_4H_6-C_4H_4S$	0.36	0.31	0.69	0.90	0.61	0.72	0.98	0.63
carbondioxide − phosphorine	$CO_2-PC_5H_5$	0.36	0.39	0.61	0.55	0.18	0.30	0.75	0.12
carbondioxide − thiophene	$CO_2-C_4H_4S$	0.42	0.51	0.69	0.47	0.23	0.30	0.87	0.17
cyclobutadiene − dithiethane	$C_4H_4-C_2H_4S_2$	1.48	0.08	0.62	0.04	1.14	1.29	0.46	1.09
cyclobutadiene − phosphorine	$C_4H_4-PC_5H_5$	0.87	0.25	0.59	0.40	0.68	0.87	1.17	0.67
cyclohexane − diphosphine	$C_6H_{12}-P_2H_4$	0.39	0.14	0.18	0.19	0.24	0.23	0.41	0.13
cyclopentadiene − thiophene	$C_5H_6-C_4H_4S$	0.25	0.40	0.58	0.69	0.48	0.59	0.95	0.44
cyclopentane − diphosphine	$C_5H_{10}-P_2H_4$	0.41	0.15	0.23	0.22	0.19	0.18	0.44	0.08
cyclopentane − phosphine	$C_5H_{10}-PH_3$	0.12	0.06	0.03	0.09	0.25	0.19	0.72	0.16
cyclopentane − phosphorus	$C_5H_{10}-P_4$	0.04	0.28	0.15	0.19	0.83	0.93	1.02	0.63
diazomethane − carbondisulfide	CH_4-CS_2	0.02	0.13	0.20	0.32	0.03	0.12	0.79	0.04
diazomethane − diphosphine	$CH_4-P_2H_4$	0.07	0.05	0.01	0.13	0.10	0.15	0.74	0.04
diazomethane − phosphine	CH_4-PH_3	0.14	0.19	0.12	0.16	0.08	0.07	0.76	0.00
diazomethane − phosphorine	$CH_4-PC_5H_5$	0.13	0.21	0.42	0.61	0.30	0.45	0.82	0.16
dimethylether − dimethyldisulfide	$C_3H_6O-CH_3S_2CH_3$	0.27	0.07	0.18	0.13	0.25	0.17	0.49	0.26
dimethylether − thiophene	$C_3H_6O-C_4H_4S$	0.17	0.20	0.16	0.23	0.12	0.17	0.55	0.17
dimethylperoxide − dimethylsulfide	$C_3H_6O_2-CH_3SCH_3$	0.22	0.09	0.07	0.19	0.12	0.09	0.63	0.09
dimethylperoxide − thiophene	$C_3H_6O_2-C_4H_4S$	0.38	0.44	0.53	0.64	0.66	0.66	0.95	0.59
ethane − dimethyldisulfide	$C_2H_6-CH_3S_2CH_3$	0.12	0.08	0.12	0.11	0.20	0.16	0.44	0.12
ethene − thiophene	$C_2H_4-C_4H_4S$	0.25	0.31	0.47	0.63	0.39	0.46	0.92	0.34
formaldehyde − phosphorine	$CH_2O-PC_5H_5$	0.67	0.21	0.51	0.41	0.39	0.50	0.80	0.31
formaldehyde − thiophene	$CH_2O-C_4H_4S$	0.37	0.36	0.56	0.57	0.37	0.43	0.95	0.20
hexane − carbondisulfide	$C_6H_{14}-CS_2$	0.34	0.05	0.04	0.32	0.25	0.38	0.64	0.10
methylamine − thiophene	$CH_3NH_2-C_4H_4S$	0.13	0.36	0.53	0.70	0.46	0.55	0.86	0.37
methylazide − thiophene	$CH_3N_3-C_4H_4S$	0.40	0.89	1.06	1.32	0.33	0.48	1.61	0.21
naphthalene − carbondisulfide	$C_{10}H_8-CS_2$	0.04	0.02	0.27	0.27	0.60	0.95	0.56	0.44
naphthalene − dimethyldisulfide	$C_{10}H_8-CH_3S_3CH_3$	0.11	0.01	0.56	0.66	1.02	1.24	0.47	0.94
naphthalene − diphospine	$C_{10}H_8-PSH_4$	0.20	0.07	0.41	0.51	0.84	1.08	0.58	0.63
naphthalene − thioacetone	$C_{10}H_8-C_3H_6S$	0.42	0.45	0.93	1.06	0.96	1.30	0.89	0.76
neohexane − diphospine	$C_6H_{14}-PSH_4$	0.32	0.09	0.16	0.10	0.22	0.20	0.56	0.06
neopentane − phospine	$C_5H_{12}-PH_3$	0.04	0.12	0.01	0.17	0.18	0.13	0.66	0.11
nonbornadiene − dimethyldisulfide	$C_7H_8-CH_3S_2CH_3$	0.08	0.14	0.24	0.55	0.51	0.45	0.63	0.43
propane − carbondisulfide	$C_3H_8-CS_2$	0.23	0.03	0.01	0.30	0.10	0.21	0.63	0.14
propane − diphospine	$C_3H_8-P_2H_4$	0.27	0.14	0.20	0.14	0.24	0.22	0.63	0.09
propane − phosphorine	$C_3H_8-PC_5H_5$	0.12	0.25	0.45	0.58	0.39	0.52	0.77	0.31
propane − phospine	$C_3H_8-PH_3$	0.08	0.03	0.07	0.05	0.14	0.12	0.61	0.06
propene − thiophene	$C_3H_8-C_4H_4S$	0.21	0.33	0.49	0.67	0.50	0.59	0.89	0.42
toluene − dimethylsulfide	$C_6H_5CH_3-CH_3SCH_3$	0.01	0.03	0.25	0.34	0.45	0.60	0.54	0.35
toluene − diphospine	$C_6H_5CH_3-P_2H_4$	0.36	0.34	0.04	0.13	0.60	0.80	0.46	0.43
toluene − phosphorine	$C_6H_5CH_3-PC_5H_5$	0.26	0.07	0.66	0.75	1.03	1.33	0.62	1.08

SAPT-DFT

Table S29: Absolute deviations for SAPT-DFT with respect to CCSD(T)/CBS. In SAPT-DFT method we used the B3LYP, PBE0 and ωB97X DFA's in conjunction with the aug-cc-pVTZ basis set. Data: 50 interaction energies. All values in kcal/mol.

Dimer	Formula	SAPT-B3LYP	SAPT-PBE0	SAPT-ωB97X
$1-2-4-triazole-thiophene$	$C_2H_3N_3-C_4H_4S$	0.90	0.98	1.46
$1-3-5-triazine-phosphine$	$C_3H_3N_3-PH_3$	0.62	0.66	0.90
$1-3-5-triazine-phosphorine$	$C_3H_3N_3-PC_5H_5$	1.14	1.19	1.74
$1-3-5-triazine-thiophene$	$C_3H_3N_3-C_4H_4S$	0.99	0.94	1.28
$2-3-dimethylbutane-diphospine$	$C_6H_{14}-P_2H_4$	0.94	1.02	1.45
$acetone-phosphorine$	$C_3H_6O-PC_5H_5$	0.92	0.85	1.56
$ammonia-phosphorine$	$NH_3-PC_5H_5$	0.53	0.48	0.80
$ammonia-thiophene$	$NH_3-C_4H_4S$	0.52	0.49	0.82
$benzene-carbondisulfide$	$C_6H_6-CS_2$	0.84	0.90	1.48
$benzene-phosphorine$	$C_6H_6-PC_5H_5$	0.88	0.80	1.29
$butadinene-thiophene$	$C_4H_6-C_4H_4S$	0.71	0.83	1.51
$carbondioxide-phosphorine$	$CO_2-PC_5H_5$	0.67	0.68	1.07
$carbondioxide-thiophene$	$CO_2-C_4H_4S$	0.71	0.71	1.06
$cyclobutadiene-dithiethane$	$C_4H_4-C_2H_4S_2$	1.51	1.44	2.37
$cyclobutadiene-phosphorine$	$C_4H_4-PC_5H_5$	0.70	0.68	1.33
$cyclohexane-diphosphine$	$C_6H_{12}-P_2H_4$	0.70	0.77	1.12
$cyclopentadiene-thiophene$	$C_5H_6-C_4H_4S$	0.59	0.58	1.06
$cyclopentane-diphosphine$	$C_5H_{10}-P_2H_4$	0.68	0.73	1.05
$cyclopentane-phosphine$	$C_5H_{10}-PH_3$	0.54	0.57	0.92
$cyclopentane-phosphorus$	$C_5H_{10}-P_4$	0.76	0.81	1.45
$diazomethane-carbondisulfide$	CH_4-CS_2	0.65	0.68	1.10
$diazomethane-diphosphine$	$CH_4-P_2H_4$	0.73	0.75	1.20
$diazomethane-phosphine$	CH_4-PH_3	0.39	0.39	0.65
$diazomethane-phosphorine$	$CH_4-PC_5H_5$	0.81	0.72	1.25
$dimethylether-dimethyldisulfide$	$C_3H_6O-CH_3S_2CH_3$	1.13	1.09	1.30
$dimethylether-thiophene$	$C_3H_6O-C_4H_4S$	0.63	0.59	0.97
$dimethylperoxide-dimethylsulfide$	$C_3H_6O_2-CH_3SCH_3$	0.77	0.76	1.22
$dimethylperoxide-thiophene$	$C_3H_6O_2-C_4H_4S$	0.58	0.57	0.85
$ethane-dimethyldisulfide$	$C_2H_6-CH_3S_2CH_3$	0.75	0.78	0.88
$ethene-thiophene$	$C_2H_4-C_4H_4S$	0.48	0.47	0.93
$formaldehyde-phosphorine$	$CH_2O-PC_5H_5$	0.66	0.62	1.25
$formaldehyde-thiophene$	$CH_2O-C_4H_4S$	0.67	0.60	0.98
$hexane-carbondisulfide$	$C_6H_{14}-CS_2$	0.91	0.98	1.62
$methylamine-thiophene$	$CH_3NH_2-C_4H_4S$	0.78	0.71	1.13
$methylazide-thiophene$	$CH_3N_3-C_4H_4S$	0.99	0.93	1.56
$naphthalene-carbondisulfide$	$C_{10}H_8-CS_2$	0.80	0.76	1.26
$naphthalene-dimethyldisulfide$	$C_{10}H_8-CH_3S_3CH_3$	1.80	1.84	2.04
$naphthalene-diphospine$	$C_{10}H_8-PSH_4$	1.37	1.32	2.07
$naphthalene-thioacetone$	$C_{10}H_8-C_3H_6S$	1.36	1.23	2.24
$neohexane-diphospine$	$C_6H_{14}-PSH_4$	0.83	0.90	1.26
$neopentane-phospine$	$C_5H_{12}-PH_3$	0.35	0.40	0.71
$nonbornadiene-dimethyldisulfide$	$C_7H_8-CH_3S_2CH_3$	1.00	0.91	0.98
$propane-carbondisulfide$	$C_3H_8-CS_2$	0.65	0.71	1.14
$propane-diphospine$	$C_3H_8-P_2H_4$	0.72	0.74	1.17
$propane-phosphorine$	$C_3H_8-PC_5H_5$	0.76	0.78	1.30
$propane-phospine$	$C_3H_8-PH_3$	0.39	0.43	0.71
$propene-thiophene$	$C_3H_8-C_4H_4S$	0.69	0.67	1.05
$toluene-dimethylsulfide$	$C_6H_5CH_3-CH_3SCH_3$	0.95	0.87	1.64
$toluene-diphospine$	$C_6H_5CH_3-P_2H_4$	1.23	1.24	1.98
$toluene-phosphorine$	$C_6H_5CH_3-PC_5H_5$	1.09	1.22	2.15

3.3 Mean calculation times

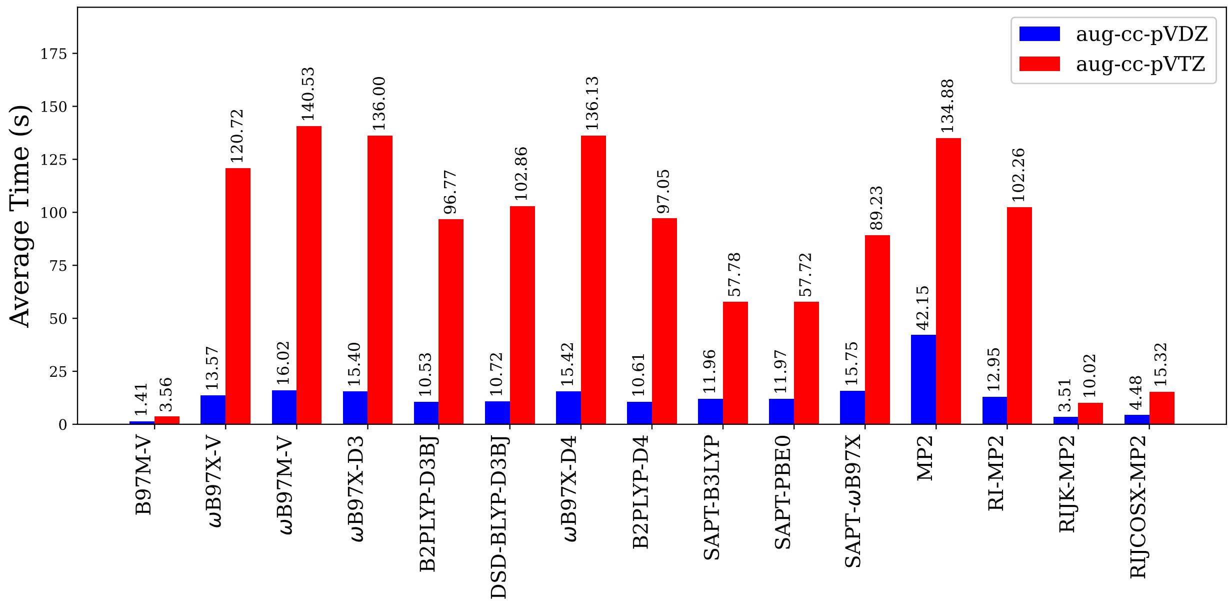

Figure S6: Comparison of computational times for different methods and basis sets in the dataset HB300SPX(S,P). All values in seconds.

4 HB300SPX(Cl,F)

4.1 aug-cc-pVDZ basis set data.

4.1.1 Ionization Potentials and HOMO values for SAPT-DFT calculations

Table S30: Experimental ionization potentials (IP) of molecules comprising dimers in the HB300SPX(Cl,F) database. IP obtained from NIST (https://webbook.nist.gov/chemistry). Kohn-Sham energies of HOMO orbitals were calculated at the DFA/aug-cc-pVDZ level of theory (DFA=B3LYP, PBE0, and ωB97X). Ionization energies in ev and HOMO energies in atomic units.

			HOMO energies (eV)			$\Delta_{XC} = \epsilon_{HOMO} - (-IP)$		
Molecule	Formula	IP	B3LYP	PBE0	ωB97X	B3LYP	PBE0	ωB97X
$1-3-5-triazine$	$C_3H_3N_3$	9.80	-7.81	-8.14	-10.63	0.07	0.06	0.03
borazine	$B_3N_3H_6$	9.88	-7.77	-8.11	-10.48	0.08	0.06	0.02
butadyine	C_4H_6	9.07	-7.33	-7.65	-9.90	0.06	0.05	0.03
carbondioxide	CO_2	13.78	-10.35	-10.70	-13.16	0.13	0.11	0.02
chlorine	Cl_2	11.48	-8.41	-8.78	-11.26	0.11	0.10	0.01
chloromethane	CH_3Cl	11.26	-8.14	-8.49	-10.99	0.11	0.10	0.01
cyclohexane	C_6H_{12}	9.88	-8.00	-8.27	-10.67	0.07	0.06	0.03
dichloromethane	CH_2Cl_2	11.33	-8.44	-8.78	-11.27	0.11	0.09	0.00
difluoromethane	CH_2F_2	12.71	-9.66	-9.97	-12.51	0.11	0.10	0.01
ethyne	C_2H_2	11.40	-8.05	-8.39	-10.86	0.12	0.11	0.02
fluorine	F_2	15.70	-11.38	-11.79	-14.39	0.16	0.14	0.05
fluoromethane	CH_3F	12.50	-9.54	-9.88	-12.42	0.11	0.10	0.00
formaldehyde	CH_2O	10.88	-7.54	-7.85	-10.33	0.12	0.11	0.02
hexafluorobenzene	C_6F_6	9.90	-7.70	-7.99	-10.21	0.08	0.07	0.01
hydrogen	H_2	15.43	-11.68	-11.97	-14.66	0.14	0.13	0.03
methylamine	CH_3NH_2	8.90	-6.62	-6.93	-9.45	0.08	0.07	0.02
neohexane	C_6H_{14}	10.07	-8.37	-8.64	-11.07	0.06	0.05	0.04
nitrogen	N_2	15.58	-11.83	-12.17	-14.85	0.14	0.13	0.03
norbornadiene	C_7H_8	8.38	-6.11	-6.40	-8.75	0.08	0.07	0.01
phosphine	PH_3	9.87	-7.53	-7.82	-10.21	0.09	0.08	0.01
phosphorine	C_5H_5P	9.00	-6.86	-7.16	-9.23	0.08	0.07	0.01
propane	C_3H_8	10.94	-8.88	-9.14	-11.63	0.08	0.07	0.03
propene	C_3H_6	9.73	-7.04	-7.34	-9.73	0.10	0.09	0.00
tetrachloroethylene	C_2Cl_4	9.33	-7.03	-7.35	-9.62	0.08	0.07	0.01
tetrachloromethane	CCl_4	11.47	-8.75	-9.13	-11.61	0.10	0.09	0.01
tetrafluoroethylene	C_2F_4	10.14	-7.35	-7.63	-10.05	0.10	0.09	0.00
thiophene	C_4H_4S	8.86	-6.53	-6.85	-9.09	0.09	0.07	0.01
toluene	C_7H_8	8.83	-6.64	-6.94	-9.10	0.08	0.07	0.01
trichloromethane	$CHCl_3$	11.37	-8.55	-8.93	-11.40	0.10	0.09	0.00
trifluoromethane	CHF_3	13.86	-11.10	-11.42	-13.98	0.10	0.09	0.00
tetrafluoromethane	CF_4	16.20	-12.23	-12.66	-15.23	0.15	0.13	0.04

4.1.2 Evaluation of mean absolute deviation in a grid of C_{OS} and C_{SS} values

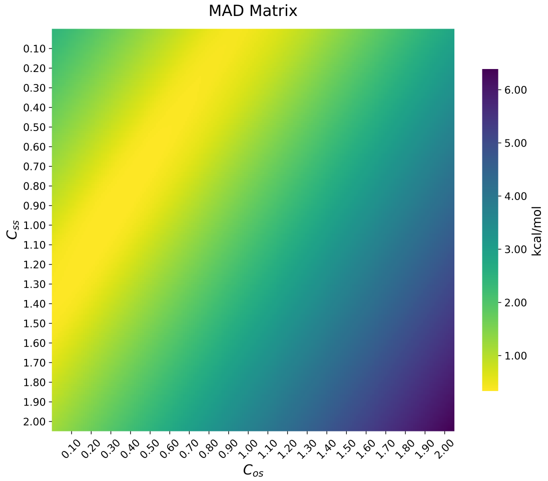

(a)

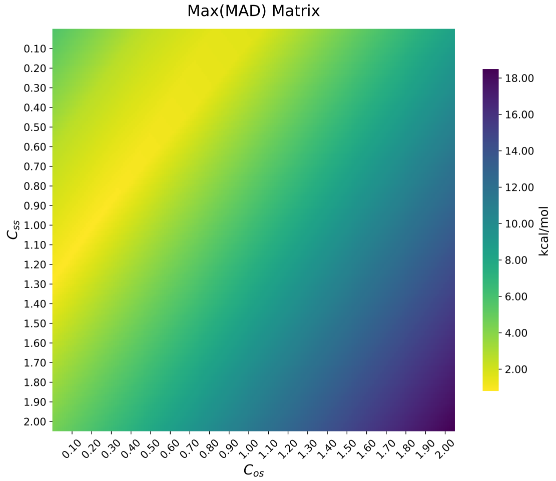

(b)

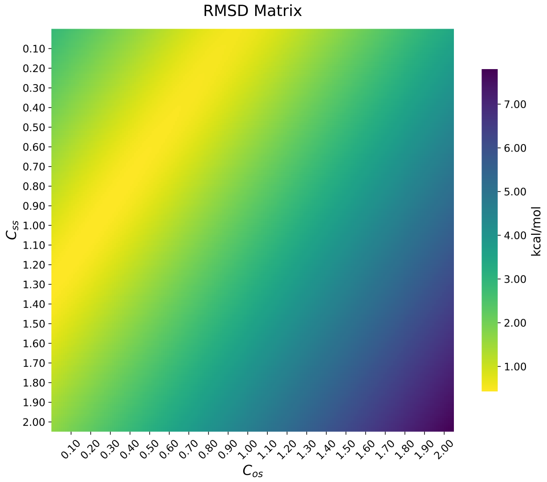

(c)

Figure S7: Evaluation of MADs (S7a), Max(MADs) (S7b) and RMSDs (S7c) in grid of values C_{OS} and C_{SS}. The optimal coefficients are C_{OS}=0.00 and C_{SS}=1.50. The theory level used is RIJK-MP2/aug-cc-pVDZ.

4.1.3 Interaction energies

MP2 with C_{OS} y C_{SS} values

Table S31: Interaction energies for CCSD(T)/CBS, MP2, SCS-MP2, SCS(MI)-MP2, SCSN-MP2, SCS-MP2-vdW, RI-MP2, RI-SCS-MP2, RIJK-MP2, RIJK-SCS-MP2, RIJCOSX-MP2, RIJCOSX-SCS-MP2, SCS-MP2^{BWI-DZ}, RI-SCS-MP2^{BWI-DZ}, RIJK-SCS-MP2^{BWI-DZ}, RIJCOSX-SCS-MP2^{BWI-DZ} and SCS-MP2-hal^{G-XZ}. Data: 30 interaction energies. MP2: $C_{OS} = C_{SS}$ =1.00; SCS-MP2: C_{OS} =1.20, C_{SS} =0.33; SCS(MI)-MP2: C_{OS} =0.40, C_{SS} =1.29; SCSN-MP2: C_{OS} =0.00, C_{SS} =1.76; SCS-MP2-vdW: C_{OS} =1.28, C_{SS} =0.50; SCS-MP2^{BWI-DZ} RI-SCS-MP2^{BWI-DZ} , RIJK-SCS-MP2^{BWI-DZ} : C_{OS} =0.00, C_{SS} =1.50; RIJCOSX-SCS-MP2^{BWI-DZ} : C_{OS} =0.00, C_{SS} =0.17. All values in kcal/mol.

Dimer	Formula	CCSD(T)/CBS	MP2	SCS-MP2	SCS(MI)-MP2	SCSN-MP2	SCS-MP2-vdW	RI-MP2	RI-SCS-MP2	RIJK-MP2	RIJK-SCS-MP2	RIJCOSX-MP2	RIJCOSX-SCS-MP2	SCS-MP2^{BWI-DZ}	RI-SCS-MP2^{BWI_DZ}	RIJK-SCS-MP2^{BWI-DZ}	RIJCOSX-SCS-MP2^{BWI-DZ}	SCS-MP2-hal^{G-XZ}
$1-3-5-triazine-tetrachloroethylene$	$C_3H_3N_3$	-4.31	-8.90	-7.10	-6.95	-6.83	-8.26	-8.92	-7.11	-8.91	-7.09	-8.91	-7.10	-5.72	-5.75	-5.73	-5.78	-5.92
$1-3-5-triazine-tetrafluoroethylene$	$C_3H_3N_3$	-2.66	-5.49	-4.47	-4.30	-4.19	-5.14	-5.50	-4.47	-5.51	-4.47	-5.49	-4.45	-3.54	-3.55	-3.56	-3.56	-3.72
$borazine-difluoromethane$	$N_3B_3H_6$	-2.37	-3.95	-3.34	-2.99	-2.80	-3.80	-3.95	-3.34	-3.95	-3.34	-3.94	-3.33	-2.38	-2.38	-2.38	-2.38	-2.67
$borazine-hexafluorobenzene$	$N_3B_3H_6$	-5.42	-11.57	-9.64	-8.88	-8.45	-11.03	-11.59	-9.65	-11.60	-9.67	-11.57	-9.63	-7.17	-7.18	-7.19	-7.22	-7.84
$borazine-tetrachloromethane$	$N_3B_3H_6$	-2.98	-5.84	-4.79	-4.08	-3.70	-5.62	-5.84	-4.79	-5.85	-4.79	-5.85	-4.80	-2.96	-2.98	-2.98	-3.02	-3.55
$butadyine-chlorine$	C_4H_2	-1.43	-0.22	0.16	-0.68	-1.09	0.14	-0.23	0.16	-0.20	0.19	-0.23	0.16	-0.99	-0.99	-0.96	-1.00	-0.37
$carbondioxide-tetrachloroethylene$	CO_2	-2.50	-3.06	-2.59	-3.00	-3.20	-2.78	-3.07	-2.59	-3.04	-2.56	-3.06	-2.57	-2.98	-2.98	-2.96	-2.98	-2.68
$carbondioxide-tetrafluoroethylene$	CO_2	-1.73	-1.69	-1.46	-1.85	-2.03	-1.51	-1.69	-1.47	-1.68	-1.45	-1.68	-1.46	-1.96	-1.96	-1.95	-1.96	-1.68
$cyclohexane-chloromethane$	C_6H_{12}	-2.15	-4.00	-3.43	-2.78	-2.45	-3.95	-4.00	-3.43	-4.00	-3.42	-4.00	-3.42	-2.00	-2.01	-2.00	-2.03	-2.52
$ethyne-tetrachloroethylene$	C_2H_2	-2.64	-3.71	-2.98	-3.15	-3.22	-3.38	-3.71	-2.98	-3.69	-2.96	-3.74	-3.01	-2.81	-2.82	-2.79	-2.86	-2.71
$fluorine-trifluoromethane$	F_2	-0.63	-1.46	-1.31	-0.88	-0.66	-1.51	-1.46	-1.31	-1.45	-1.30	-1.45	-1.30	-0.50	-0.50	-0.49	-0.50	-0.83
$formaldehyde-tetrachloromethane$	CH_2O	-1.78	-2.76	-2.36	-2.07	-1.92	-2.68	-2.76	-2.36	-2.76	-2.36	-2.76	-2.36	-1.63	-1.64	-1.64	-1.66	-1.87
$formaldehyde-trifluoromethane$	CH_2O	-1.29	-1.99	-1.84	-1.63	-1.53	-1.98	-1.99	-1.84	-1.99	-1.83	-2.00	-1.84	-1.41	-1.41	-1.40	-1.42	-1.57
$hydrogen-hexafluorobenzene$	H_2	-0.71	-1.33	-1.16	-0.95	-0.84	-1.32	-1.33	-1.16	-1.33	-1.16	-1.31	-1.13	-0.70	-0.70	-0.70	-0.68	-0.87
$hydrogen-tetrachloromethane$	H_2	-0.48	-1.23	-1.20	-0.65	-0.37	-1.36	-1.23	-1.20	-1.23	-1.20	-1.22	-1.19	-0.27	-0.26	-0.26	-0.26	-0.69
$methylamine-dichloromethane$	CH_3NH_2	-2.06	-3.29	-2.69	-2.29	-2.08	-3.16	-3.29	-2.69	-3.28	-2.68	-3.29	-2.70	-1.66	-1.67	-1.66	-1.68	-1.99
$methylamine-trifluoromethane$	CH_3NH_2	-1.34	-2.40	-2.12	-1.64	-1.39	-2.42	-2.40	-2.12	-2.40	-2.12	-2.39	-2.12	-1.15	-1.15	-1.14	-1.16	-1.53
$neohexane-chlorine$	C_6H_{14}	-1.69	-3.93	-3.47	-2.73	-2.34	-3.95	-3.94	-3.47	-3.93	-3.46	-3.96	-3.49	-1.95	-1.96	-1.96	-2.00	-2.54
$nitrogen-chlorine$	N_2	-0.73	0.95	0.90	-0.10	-0.60	1.18	0.95	0.90	0.96	0.92	0.95	0.91	-0.78	-0.78	-0.77	-0.77	-0.02
$nonbornadiene-chlorine$	C_7H_8	-2.14	-3.64	-2.83	-2.62	-2.50	-3.39	-3.65	-2.83	-3.64	-2.82	-3.67	-2.85	-1.98	-1.99	-1.98	-2.03	-2.17
$phosphine-hexafluorobenzene$	PH_3	-2.65	-5.01	-4.06	-3.74	-3.56	-4.73	-5.01	-4.07	-5.01	-4.06	-4.97	-4.03	-2.94	-2.94	-2.94	-2.95	-3.22
$phosphorine-chlorine$	PC_5H_5	-2.25	-3.71	-2.64	-2.72	-2.73	-3.29	-3.73	-2.65	-3.72	-2.64	-3.72	-2.64	-2.09	-2.11	-2.10	-2.13	-2.09
$phosphorine-tetrafluoromethane$	PC_5H_5	-1.75	-2.73	-2.06	-2.15	-2.17	-2.46	-2.74	-2.07	-2.73	-2.07	-2.73	-2.07	-1.78	-1.79	-1.78	-1.80	-1.75
$propane-difluoromethane$	C_3H_8	-1.68	-3.25	-2.83	-2.25	-1.95	-3.23	-3.25	-2.83	-3.24	-2.82	-3.23	-2.81	-1.61	-1.61	-1.60	-1.60	-2.06
$propene-tetrachloroethylene$	C_3H_6	-3.67	-7.40	-6.10	-5.65	-5.38	-7.02	-7.41	-6.11	-7.39	-6.09	-7.42	-6.12	-4.53	-4.55	-4.53	-4.59	-4.94
$tetrafluoromethane-difluoromethane$	CF_4	-1.33	-2.33	-2.09	-1.73	-1.53	-2.33	-2.33	-2.09	-2.33	-2.09	-2.37	-2.13	-1.33	-1.33	-1.33	-1.37	-1.62
$thiophene-fluoromethane$	C_4H_4S	-2.52	-4.36	-3.87	-3.48	-3.28	-4.27	-4.36	-3.87	-4.36	-3.87	-4.35	-3.86	-2.92	-2.92	-2.92	-2.93	-3.24
$thiophene-hexafluorobenzene$	C_4H_4S	-5.83	-11.95	-10.03	-9.36	-8.98	-11.39	-11.96	-10.04	-11.97	-10.05	-12.00	-10.07	-7.72	-7.73	-7.73	-7.80	-8.32
$thiophene-trichloromethane$	C_4H_4S	-2.93	-5.16	-4.07	-3.69	-3.48	-4.84	-5.17	-4.07	-5.16	-4.07	-5.16	-4.07	-2.76	-2.77	-2.76	-2.79	-3.10
$toluene-trifluoromethane$	$C_6H_5CH_3$	-1.73	-3.65	-2.95	-2.54	-2.32	-3.49	-3.65	-2.96	-3.65	-2.96	-3.68	-2.99	-1.84	-1.84	-1.84	-1.89	-2.18

DFT

Table S32: Interaction energies for CCSD(T)/CBS, B97M-V, ωB97X-V, ωB97M-V, ωB97X-D3, B2PLYP-D3BJ, DSD-BLYP-D3BJ,ωB97X-D4 and B2PLYP-D4. Data: 30 interaction energies. All values in kcal/mol.

Dimer	Formula	CCSD(T)/CBS	B97M-V	ωB97X-V	ωB97M-V	ωB97X-D3	B2PLYP-D3BJ	DSD-BLYP-D3BJ	ωB97X-D4	B2PLYP-D4
$1-3-5-triazine-tetrachloroethylene$	$C_3H_3N_3-C_2Cl_4$	-4.31	-5.09	-5.53	-5.87	-5.78	-5.95	-6.40	-5.43	-6.10
$1-3-5-triazine-tetrafluoroethylene$	$C_3H_3N_3-C_2F_4$	-2.66	-3.45	-3.64	-3.73	-3.13	-3.66	-4.00	-3.17	-3.84
$borazine-difluoromethane$	$N_3B_3H_6-CH_2F_2$	-2.37	-3.22	-3.18	-3.17	-3.17	-3.33	-3.39	-3.35	-3.26
$borazine-hexafluorobenzene$	$N_3B_3H_6-C_6F_6$	-5.42	-7.49	-7.27	-7.67	-6.99	-7.89	-8.55	-6.49	-8.11
$borazine-tetrachloromethane$	$N_3B_3H_6-Cl_2$	-2.98	-4.07	-3.76	-4.04	-4.11	-4.31	-4.43	-3.98	-4.37
$butadyine-chlorine$	$C_4H_2-Cl_2$	-1.43	-1.36	-1.47	-1.70	-1.77	-0.66	-0.57	-1.97	-0.63
$carbondioxide-tetrachloroethylene$	$CO_2-C_2Cl_4$	-2.50	-2.77	-2.90	-3.07	-2.61	-2.70	-2.83	-2.93	-2.71
$carbondioxide-tetrafluoroethylene$	$CO_2-C_2F_4$	-1.73	-1.95	-1.98	-1.94	-1.43	-1.61	-1.70	-1.80	-1.68
$cyclohexane-chloromethane$	$C_6H_{12}-CH_3Cl$	-2.15	-2.46	-2.81	-2.82	-2.97	-3.09	-3.16	-3.18	-2.97
$ethyne-tetrachloroethylene$	$C_2H_2-C_2Cl_4$	-2.64	-2.88	-3.18	-3.53	-3.53	-2.88	-2.99	-3.61	-2.86
$fluorine-trifluoromethane$	F_2-CHF_3	-0.63	-1.03	-1.06	-0.99	-0.42	-1.09	-1.12	-0.73	-1.15
$formaldehyde-tetrachloromethane$	CH_2O-CHF_3	-1.78	-2.42	-2.17	-2.28	-2.41	-2.50	-2.44	-2.53	-2.36
$formaldehyde-trifluoromethane$	CH_2O-CF_4	-1.29	-1.78	-1.92	-1.77	-1.66	-1.78	-1.81	-1.80	-1.80
$hydrogen-hexafluorobenzene$	$H_2-C_6F_6$	-0.71	-1.09	-1.09	-1.11	-1.03	-1.09	-1.12	-1.16	-1.05
$hydrogen-tetrachloromethane$	H_2-CCl_4	-0.48	-0.83	-0.78	-0.83	-0.94	-1.08	-1.01	-0.98	-1.04
$methylamine-dichloromethane$	$CH_3NH_2-CH_2Cl_2$	-2.06	-2.33	-2.48	-2.55	-2.78	-2.68	-2.70	-2.90	-2.59
$methylamine-trifluoromethane$	$CH_3NH_2-CHF_3$	-1.34	-1.71	-2.04	-1.83	-1.67	-1.99	-2.01	-1.82	-2.03
$neohexane-chlorine$	$C_6H_{14}-Cl_2$	-1.69	-2.41	-2.52	-2.41	-2.75	-2.82	-2.96	-2.67	-2.59
$nitrogen-chlorine$	N_2-Cl_2	-0.73	-0.91	-0.85	-1.00	-0.93	-0.08	0.07	-1.17	-0.04
$nonbornadiene-chlorine$	$C_7H_8-Cl_2$	-2.14	-2.73	-2.84	-3.08	-3.18	-2.81	-2.85	-3.07	-2.69
$phosphine-hexafluorobenzene$	$PH_3-C_6F_6$	-2.65	-3.16	-3.37	-3.62	-3.30	-3.64	-3.84	-3.49	-3.56
$phosphorine-chlorine$	$PC_5H_5-Cl_2$	-2.25	-2.55	-2.50	-2.87	-2.79	-2.63	-2.73	-2.83	-2.54
$phosphorine-tetrafluoromethane$	$PC_5H_5-CF_4$	-1.75	-2.08	-2.30	-2.33	-1.79	-1.89	-2.03	-1.90	-2.04
$propane-difluoromethane$	$C_3H_8-CH_2F_2$	-1.68	-2.29	-2.61	-2.43	-2.23	-2.64	-2.67	-2.55	-2.58
$propene-tetrachloroethylene$	$C_3H_6-C_2Cl_4$	-3.67	-4.60	-4.93	-5.28	-5.42	-5.22	-5.53	-5.11	-5.09
$tetrafluoromethane-difluoromethane$	$CF_4-CH_2F_2$	-1.33	-1.76	-1.96	-1.77	-1.13	-1.85	-1.93	-1.40	-1.99
$thiophene-fluoromethane$	$C_4H_4S-CF_4$	-2.52	-3.25	-3.53	-3.63	-3.54	-3.53	-3.69	-3.74	-3.48
$thiophene-hexafluorobenzene$	$C_4H_4S-C_6F_6$	-5.83	-7.19	-7.52	-8.07	-7.15	-8.22	-8.84	-6.72	-8.38
$thiophene-trichloromethane$	$C_4H_4S-CHCl_3$	-2.93	-3.67	-3.75	-4.17	-4.05	-3.88	-3.96	-3.96	-3.88
$toluene-trifluoromethane$	$C_6H_5CH_3-CHF_3$	-1.73	-2.54	-2.79	-2.76	-2.16	-2.48	-2.64	-2.23	-2.64

SAPT-DFT

Table S33: Interaction energies for CCSD(T)/CBS SAPT-DFT. For SAPT-DFT method we used the B3LYP, PBE0 and ωB97X DFA's in conjunction with the aug-cc-pVDZ basis set. Data: 30 interaction energies. All values in kcal/mol.

Dimer	Formula	CCSD(T)/CBS	SAPT-B3LYP	SAPT-PBE0	SAPT-ωB97X
$1-3-5-triazine-tetrachloroethylene$	$C_3H_3N_3{-}C_2Cl_4$	-4.31	-2.25	-2.29	-1.75
$1-3-5-triazine-tetrafluoroethylene$	$C_3H_3N_3{-}C_2F_4$	-2.66	-1.39	-1.45	-1.12
$borazine-difluoromethane$	$N_3B_3H_6{-}CH_2F_2$	-2.37	-1.44	-1.52	-1.20
$borazine-hexafluorobenzene$	$N_3B_3H_6{-}C_6F_6$	-5.42	-3.15	-3.14	-2.53
$borazine-tetrachloromethane$	$N_3B_3H_6{-}Cl_2$	-2.98	-1.48	-1.42	-0.83
$butadyine-chlorine$	$C_4H_2{-}Cl_2$	-1.43	-0.56	-0.54	-0.32
$carbondioxide-tetrachloroethylene$	$CO_2{-}C_2Cl_4$	-2.50	-1.30	-1.27	-0.97
$carbondioxide-tetrafluoroethylene$	$CO_2{-}C_2F_4$	-1.73	-0.98	-0.94	-0.74
$cyclohexane-chloromethane$	$C_6H_{12}{-}CH_3Cl$	-2.15	-1.30	-1.26	-0.86
$ethyne-tetrachloroethylene$	$C_2H_2{-}C_2Cl_4$	-2.64	-1.55	-1.48	-1.05
$fluorine-trifluoromethane$	$F_2{-}CHF_3$	-0.63	-0.23	-0.25	-0.16
$formaldehyde-tetrachloromethane$	$CH_2O{-}CHF_3$	-1.78	-1.00	-1.01	-0.64
$formaldehyde-trifluoromethane$	$CH_2O{-}CF_4$	-1.29	-0.87	-0.87	-0.74
$methylamine-dichloromethane$	$CH_3NH_2{-}CH_2Cl_2$	-2.06	-1.03	-0.99	-0.61
$methylamine-trifluoromethane$	$CH_3NH_2{-}CHF_3$	-1.34	-0.65	-0.64	-0.51
$neohexane-chlorine$	$C_6H_{14}{-}Cl_2$	-1.69	-0.87	-0.82	-0.52
$nitrogen-chlorine$	$N_2{-}Cl_2$	-0.73	-0.29	-0.30	-0.09
$nonbornadiene-chlorine$	$C_7H_8{-}Cl_2$	-2.14	-1.01	-0.96	-0.41
$phosphine-hexafluorobenzene$	$PH_3{-}C_6F_6$	-2.65	-1.58	-1.56	-1.22
$phosphorine-chlorine$	$PC_5H_5{-}Cl_2$	-2.25	-1.10	-1.08	-0.55
$phosphorine-tetrafluoromethane$	$PC_5H_5{-}CF_4$	-1.75	-0.95	-0.96	-0.66
$propane-difluoromethane$	$C_3H_8{-}CH_2F_2$	-1.68	-0.90	-0.91	-0.66
$propene-tetrachloroethylene$	$C_3H_6{-}C_2Cl_4$	-3.67	-2.14	-2.09	-1.38
$tetrafluoromethane-difluoromethane$	$CF_4{-}CH_2F_2$	-1.33	-0.73	-0.71	-0.52
$thiophene-fluoromethane$	$C_4H_4S{-}CF_4$	-2.52	-1.66	-1.72	-1.34
$thiophene-hexafluorobenzene$	$C_4H_4S{-}C_6F_6$	-5.83	-3.85	-3.84	-3.16
$thiophene-trichloromethane$	$C_4H_4S{-}CHCl_3$	-2.93	-1.44	-1.41	-0.68
$toluene-trifluoromethane$	$C_6H_5CH_3{-}CHF_3$	-1.73	-0.92	-0.88	-0.52

4.1.4 Absolute deviations of molecular systems.

MP2 with C_{OS} y C_{SS} values

Table S34: Absolute deviations for MP2, SCS-MP2, SCS(MI)-MP2, SCSN-MP2, SCS-MP2-vdW, RI-MP2, RI-SCS-MP2, RIJK-MP2, RIJK-SCS-MP2, RIJCOSX-MP2, RIJCOSX-SCS-MP2, SCS-MP2^{BWI-DZ}, RI-SCS-MP2^{BWI-DZ}, RIJK-SCS-MP2^{BWI-DZ}, RIJCOSX-SCS-MP2^{BWI-DZ} and SCS-MP2-hal^{G-XZ}. Data: 30 interaction energies. MP2: C_{OS} = C_{SS} =1.00; SCS-MP2: C_{OS} =1.20, C_{SS} =0.33; SCS(MI)-MP2: C_{OS} =0.40, C_{SS} =1.29; SCSN-MP2: C_{OS} =0.00, C_{SS} =1.76; SCS-MP2-vdW: C_{OS} =1.28, C_{SS} =0.50; SCS-MP2^{BWI-DZ} RI-SCS-MP2^{BWI-DZ} , RIJK-SCS-MP2^{BWI-DZ} : C_{OS} =0.00, C_{SS} =1.50; RIJCOSX-SCS-MP2^{BWI-DZ} : C_{OS} =0.00, C_{SS} =0.17. All values in kcal/mol.

Dimer	Formula	MP2	SCS-MP2	SCS(MI)-MP2	SCSN-MP2	SCS-MP2-vdW	RI-MP2	RI-SCS-MP2	RIJK-MP2	RIJK-SCS-MP2	RIJCOSX-MP2	RIJCOSX-SCS-MP2	SCS-MP2^{BWI-DZ}	RI-SCS-MP2^{BWI-DZ}	RIJK-SCS-MP2^{BWI-DZ}	RIJCOSX-SCS-MP2^{BWI-DZ}	SCS-MP2-hal^{G-XZ}
1 − 3 − 5 − *triazine* − *tetrachloroethylene*	$C_3H_3N_3$	4.59	2.79	2.64	2.52	3.95	4.61	2.80	4.60	2.78	4.60	2.79	1.41	1.44	1.42	1.47	1.61
1 − 3 − 5 − *triazine* − *tetrafluoroethylene*	$C_3H_3N_3$	2.83	1.81	1.64	1.53	2.48	2.84	1.81	2.85	1.81	2.83	1.79	0.88	0.89	0.90	0.90	1.06
borazine − *difluoromethane*	$N_3B_3H_6$	1.58	0.97	0.62	0.43	1.43	1.58	0.97	1.58	0.97	1.57	0.96	0.01	0.01	0.01	0.01	0.30
borazine − *hexafluorobenzene*	$N_3B_3H_6$	6.15	4.22	3.46	3.03	5.61	6.17	4.23	6.18	4.25	6.15	4.21	1.75	1.76	1.77	1.80	2.42
borazine − *tetrachloromethane*	$N_3B_3H_6$	2.86	1.81	1.10	0.72	2.64	2.86	1.81	2.87	1.81	2.87	1.82	0.02	0.00	0.00	0.04	0.57
butadyine − *chlorine*	C_4H_2	1.21	1.59	0.75	0.34	1.57	1.20	1.59	1.23	1.62	1.20	1.59	0.44	0.44	0.47	0.43	1.06
carbondioxide − *tetrachloroethylene*	CO_2	0.56	0.09	0.50	0.70	0.28	0.57	0.09	0.54	0.06	0.56	0.07	0.48	0.48	0.46	0.48	0.18
carbondioxide − *tetrafluoroethylene*	CO_2	0.04	0.27	0.12	0.30	0.22	0.04	0.26	0.05	0.28	0.05	0.27	0.23	0.23	0.22	0.23	0.05
cyclohexane − *chloromethane*	C_6H_{12}	1.85	1.28	0.63	0.30	1.80	1.85	1.28	1.85	1.27	1.85	1.27	0.15	0.14	0.15	0.12	0.37
ethyne − *tetrachloroethylene*	C_2H_2	1.07	0.34	0.51	0.58	0.74	1.07	0.34	1.05	0.32	1.10	0.37	0.17	0.18	0.15	0.22	0.07
fluorine − *trifluoromethane*	F_2	0.83	0.68	0.25	0.03	0.88	0.83	0.68	0.82	0.67	0.82	0.67	0.13	0.13	0.14	0.13	0.20
formaldehyde − *tetrachloromethane*	CH_2O	0.98	0.58	0.29	0.14	0.90	0.98	0.58	0.98	0.58	0.98	0.58	0.15	0.14	0.14	0.12	0.09
formaldehyde − *trifluoromethane*	CH_2O	0.70	0.55	0.34	0.24	0.69	0.70	0.55	0.70	0.54	0.71	0.55	0.12	0.12	0.11	0.13	0.28
hydrogen − *hexafluorobenzene*	H_2	0.62	0.45	0.24	0.13	0.61	0.62	0.45	0.62	0.45	0.60	0.42	0.01	0.01	0.01	0.03	0.16
hydrogen − *tetrachloromethane*	H_2	0.75	0.72	0.17	0.11	0.88	0.75	0.72	0.75	0.72	0.74	0.71	0.21	0.22	0.22	0.22	0.21
methylamine − *dichloromethane*	CH_3NH_2	1.23	0.63	0.23	0.02	1.10	1.23	0.63	1.22	0.62	1.23	0.64	0.40	0.39	0.40	0.38	0.07
methylamine − *trifluoromethane*	CH_3NH_2	1.06	0.78	0.30	0.05	1.08	1.06	0.78	1.06	0.78	1.05	0.78	0.19	0.19	0.20	0.18	0.19
neohexane − *chlorine*	C_6H_{14}	2.24	1.78	1.04	0.65	2.26	2.25	1.78	2.24	1.77	2.27	1.80	0.26	0.27	0.27	0.31	0.85
nitrogen − *chlorine*	N_2	1.68	1.63	0.63	0.13	1.91	1.68	1.63	1.69	1.65	1.68	1.64	0.05	0.05	0.04	0.04	0.71
nonbornadiene − *chlorine*	C_7H_8	1.50	0.69	0.48	0.36	1.25	1.51	0.69	1.50	0.68	1.53	0.71	0.16	0.15	0.16	0.11	0.03
phosphine − *hexafluorobenzene*	PH_3	2.36	1.41	1.09	0.91	2.08	2.36	1.42	2.36	1.41	2.32	1.38	0.29	0.29	0.29	0.30	0.57
phosphorine − *chlorine*	PC_5H_5	1.46	0.39	0.47	0.48	1.04	1.48	0.40	1.47	0.39	1.47	0.39	0.16	0.14	0.15	0.12	0.16
phosphorine − *tetrafluoromethane*	PC_5H_5	0.98	0.31	0.40	0.42	0.71	0.99	0.32	0.98	0.32	0.98	0.32	0.03	0.04	0.03	0.05	0.00
propane − *difluoromethane*	C_3H_8	1.57	1.15	0.57	0.27	1.55	1.57	1.15	1.56	1.14	1.55	1.13	0.07	0.07	0.08	0.08	0.38
propene − *tetrachloroethylene*	C_3H_6	3.73	2.43	1.98	1.71	3.35	3.74	2.44	3.72	2.42	3.75	2.45	0.86	0.88	0.86	0.92	1.27
tetrafluoromethane − *difluoromethane*	CF_4	1.00	0.76	0.40	0.20	1.00	1.00	0.76	1.00	0.76	1.04	0.80	0.00	0.00	0.00	0.04	0.29
thiophene − *fluoromethane*	C_4H_4S	1.84	1.35	0.96	0.76	1.75	1.84	1.35	1.84	1.35	1.83	1.34	0.40	0.40	0.40	0.41	0.72
thiophene − *hexafluorobenzene*	C_4H_4S	6.12	4.20	3.53	3.15	5.56	6.13	4.21	6.14	4.22	6.17	4.24	1.89	1.90	1.90	1.97	2.49
thiophene − *trichloromethane*	C_4H_4S	2.23	1.14	0.76	0.55	1.91	2.24	1.14	2.23	1.14	2.23	1.14	0.17	0.16	0.17	0.14	0.17
toluene − *trifluoromethane*	$C_6H_5CH_3$	1.92	1.22	0.81	0.59	1.76	1.92	1.23	1.92	1.23	1.95	1.26	0.11	0.11	0.11	0.16	0.45

DFT

Table S35: Absolute deviations for B97M-V, ωB97X-V, ωB97M-V, ωB97X-D3, B2PLYP-D3BJ, DSD-BLYP-D3BJ, ωB97X-D4 and B2PLYP-D4. Data: 30 interaction energies. All values in kcal/mol.

Dimer	Formula	B97M-V	ωB97X-V	ωB97M-V	ωB97X-D3	B2PLYP-D3BJ	DSD-BLYP-D3BJ	ωB97X-D4	B2PLYP-D4
1 − 3 − 5 − *triazine* − *tetrachloroethylene*	$C_3H_3N_3$−C_2Cl_4	0.78	1.22	1.56	1.47	1.64	2.09	1.12	1.79
1 − 3 − 5 − *triazine* − *tetrafluoroethylene*	$C_3H_3N_3$−C_2F_4	0.79	0.98	1.07	0.47	1.00	1.34	0.51	1.18
borazine − *difluoromethane*	$N_3B_3H_6$−CH_2F_2	0.85	0.81	0.80	0.80	0.96	1.02	0.98	0.89
borazine − *hexafluorobenzene*	$N_3B_3H_6$−C_6F_6	2.07	1.85	2.25	1.57	2.47	3.13	1.07	2.69
borazine − *tetrachloromethane*	$N_3B_3H_6$−Cl_2	1.09	0.78	1.06	1.13	1.33	1.45	1.00	1.39
butadyine − *chlorine*	C_4H_2−Cl_2	0.07	0.04	0.27	0.34	0.77	0.86	0.54	0.80
carbondioxide − *tetrachloroethylene*	CO_2−C_2Cl_4	0.27	0.40	0.57	0.11	0.20	0.33	0.43	0.21
carbondioxide − *tetrafluoroethylene*	CO_2−C_2F_4	0.22	0.25	0.21	0.30	0.12	0.03	0.07	0.05
cyclohexane − *chloromethane*	C_6H_{12}−CH_3Cl	0.31	0.66	0.67	0.82	0.94	1.01	1.03	0.82
ethyne − *tetrachloroethylene*	C_2H_2−C_2Cl_4	0.24	0.54	0.89	0.89	0.24	0.35	0.97	0.22
fluorine − *trifluoromethane*	F_2−CHF_3	0.40	0.43	0.36	0.21	0.46	0.49	0.10	0.52
formaldehyde − *tetrachloromethane*	CH_2O−CHF_3	0.64	0.39	0.50	0.63	0.72	0.66	0.75	0.58
formaldehyde − *trifluoromethane*	CH_2O−CF_4	0.49	0.63	0.48	0.37	0.49	0.52	0.51	0.51
hydrogen − *hexafluorobenzene*	H_2−C_6F_6	0.38	0.38	0.40	0.32	0.38	0.41	0.45	0.34
hydrogen − *tetrachloromethane*	H_2−CCl_4	0.35	0.30	0.35	0.46	0.60	0.53	0.50	0.56
methylamine − *dichloromethane*	CH_3NH_2−CH_2Cl_2	0.27	0.42	0.49	0.72	0.62	0.64	0.84	0.53
methylamine − *trifluoromethane*	CH_3NH_2−CHF_3	0.37	0.70	0.49	0.33	0.65	0.67	0.48	0.69
neohexane − *chlorine*	C_6H_{14}−Cl_2	0.72	0.83	0.72	1.06	1.13	1.27	0.98	0.90
nitrogen − *chlorine*	N_2−Cl_2	0.18	0.12	0.27	0.20	0.65	0.80	0.44	0.69
nonbornadiene − *chlorine*	C_7H_8−Cl_2	0.59	0.70	0.94	1.04	0.67	0.71	0.93	0.55
phosphine − *hexafluorobenzene*	PH_3−C_6F_6	0.51	0.72	0.97	0.65	0.99	1.19	0.84	0.91
phosphorine − *chlorine*	PC_5H_5−Cl_2	0.30	0.25	0.62	0.54	0.38	0.48	0.58	0.29
phosphorine − *tetrafluoromethane*	PC_5H_5−CF_4	0.33	0.55	0.58	0.04	0.14	0.28	0.15	0.29
propane − *difluoromethane*	C_3H_8−CH_2F_2	0.61	0.93	0.75	0.55	0.96	0.99	0.87	0.90
propene − *tetrachloroethylene*	C_3H_6−C_2Cl_4	0.93	1.26	1.61	1.75	1.55	1.86	1.44	1.42
tetrafluoromethane − *difluoromethane*	CF_4−CH_2F_2	0.43	0.63	0.44	0.20	0.52	0.60	0.07	0.66
thiophene − *fluoromethane*	C_4H_4S−CF_4	0.73	1.01	1.11	1.02	1.01	1.17	1.22	0.96
thiophene − *hexafluorobenzene*	C_4H_4S−C_6F_6	1.36	1.69	2.24	1.32	2.39	3.01	0.89	2.55
thiophene − *trichloromethane*	C_4H_4S−$CHCl_3$	0.74	0.82	1.24	1.12	0.95	1.03	1.03	0.95
toluene − *trifluoromethane*	$C_6H_5CH_3$−CHF_3	0.81	1.06	1.03	0.43	0.75	0.91	0.50	0.91

SAPT-DFT

Table S36: Absolute deviations for SAPT-DFT with respect to CCSD(T)/CBS. In SAPT-DFT method we used the B3LYP, PBE0 and ωB97X DFA's in conjunction with the aug-cc-pVDZ basis set. Data: 30 interaction energies. All values in kcal/mol.

Dimer	Formula	SAPT-B3LYP	SAPT-PBE0	SAPT-ωB97X
$1-3-5-triazine-tetrachloroethylene$	$C_3H_3N_3$−C_2Cl_4	2.06	2.02	2.56
$1-3-5-triazine-tetrafluoroethylene$	$C_3H_3N_3$−C_2F_4	1.27	1.21	1.54
$borazine-difluoromethane$	$N_3B_3H_6$−CH_2F_2	0.93	0.85	1.17
$borazine-hexafluorobenzene$	$N_3B_3H_6$−C_6F_6	2.27	2.28	2.89
$borazine-tetrachloromethane$	$N_3B_3H_6$−Cl_2	1.50	1.56	2.15
$butadyine-chlorine$	C_4H_2−Cl_2	0.87	0.89	1.11
$carbondioxide-tetrachloroethylene$	CO_2−C_2Cl_4	1.20	1.23	1.53
$carbondioxide-tetrafluoroethylene$	CO_2−C_2F_4	0.75	0.79	0.99
$cyclohexane-chloromethane$	C_6H_{12}−CH_3Cl	0.85	0.89	1.29
$ethyne-tetrachloroethylene$	C_2H_2−C_2Cl_4	1.09	1.16	1.59
$fluorine-trifluoromethane$	F_2−CHF_3	0.40	0.38	0.47
$formaldehyde-tetrachloromethane$	CH_2O−CHF_3	0.78	0.77	1.14
$formaldehyde-trifluoromethane$	CH_2O−CF_4	0.42	0.42	0.55
$methylamine-dichloromethane$	CH_3NH_2−CH_2Cl_2	1.03	1.07	1.45
$methylamine-trifluoromethane$	CH_3NH_2−CHF_3	0.69	0.70	0.83
$neohexane-chlorine$	C_6H_{14}−Cl_2	0.82	0.87	1.17
$nitrogen-chlorine$	N_2−Cl_2	0.44	0.43	0.64
$nonbornadiene-chlorine$	C_7H_8−Cl_2	1.13	1.18	1.73
$phosphine-hexafluorobenzene$	PH_3−C_6F_6	1.07	1.09	1.43
$phosphorine-chlorine$	PC_5H_5−Cl_2	1.15	1.17	1.70
$phosphorine-tetrafluoromethane$	PC_5H_5−CF_4	0.80	0.79	1.09
$propane-difluoromethane$	C_3H_8−CH_2F_2	0.78	0.77	1.02
$propene-tetrachloroethylene$	C_3H_6−C_2Cl_4	1.53	1.58	2.29
$tetrafluoromethane-difluoromethane$	CF_4−CH_2F_2	0.60	0.62	0.81
$thiophene-fluoromethane$	C_4H_4S−CF_4	0.86	0.80	1.18
$thiophene-hexafluorobenzene$	C_4H_4S−C_6F_6	1.98	1.99	2.67
$thiophene-trichloromethane$	C_4H_4S−$CHCl_3$	1.49	1.52	2.25
$toluene-trifluoromethane$	$C_6H_5CH_3$−CHF_3	0.81	0.85	1.21

4.2 aug-cc-pVTZ basis set data.

4.2.1 Ionization Potentials and HOMO values for SAPT-DFT calculations

Table S37: Experimental ionization potentials (IP) of molecules comprising dimers in the HB300SPX(Cl,F) database. IP obtained from NIST (https://webbook.nist.gov/chemistry). Kohn-Sham energies of HOMO orbitals were calculated at the DFA/aug-cc-pVTZ level of theory (DFA=B3LYP, PBE0, and ωB97X). Ionization energies in ev and HOMO energies in atomic units.

			HOMO energies (eV)			$\Delta_{XC} = \epsilon_{HOMO} - (-IP)$		
Molecule	Formula	IP	B3LYP	PBE0	ωB97X	B3LYP	PBE0	ωB97X
$1-3-5-triazine$	$C_3H_3N_3$	9.80	-7.83	-8.15	-10.62	0.07	0.06	0.03
borazine	$B_3N_3H_6$	9.88	-7.80	-8.14	-10.51	0.08	0.06	0.02
butadyine	C_4H_6	9.07	-7.34	-7.66	-9.88	0.06	0.05	0.03
carbondioxide	CO_2	13.78	-10.36	-10.72	-13.14	0.13	0.11	0.02
chlorine	Cl_2	11.48	-8.40	-8.75	-11.21	0.11	0.10	0.01
chloromethane	CH_3Cl	11.26	-8.15	-8.49	-10.97	0.11	0.10	0.01
cyclohexane	C_6H_{12}	9.88	-8.02	-8.29	-10.68	0.07	0.06	0.03
dichloromethane	CH_2Cl_2	11.33	-8.44	-8.77	-11.25	0.11	0.09	0.00
difluoromethane	CH_2F_2	12.71	-9.68	-9.98	-12.51	0.11	0.10	0.01
ethyne	C_2H_2	11.40	-8.07	-8.40	-10.85	0.12	0.11	0.02
fluorine	F_2	15.70	-11.37	-11.77	-14.33	0.16	0.14	0.05
fluoromethane	CH_3F	12.50	-9.57	-9.90	-12.42	0.11	0.10	0.00
formaldehyde	CH_2O	10.88	-7.58	-7.88	-10.35	0.12	0.11	0.02
hexafluorobenzene	C_6F_6	9.90	-7.70	-7.98	-10.17	0.08	0.07	0.01
hydrogen	H_2	15.43	-11.75	-12.02	-14.74	0.13	0.13	0.03
methylamine	CH_3NH_2	8.90	-6.65	-6.96	-9.46	0.08	0.07	0.02
neohexane	C_6H_{14}	10.07	-8.39	-8.66	-11.08	0.06	0.05	0.04
nitrogen	N_2	15.58	-11.86	-12.19	-14.84	0.14	0.12	0.03
norbornadiene	C_7H_8	8.38	-6.12	-6.42	-8.75	0.08	0.07	0.01
phosphine	PH_3	9.87	-7.56	-7.85	-10.22	0.08	0.07	0.01
phosphorine	C_5H_5P	9.00	-6.85	-7.16	-9.20	0.08	0.07	0.01
propane	C_3H_8	10.94	-8.90	-9.16	-11.65	0.07	0.07	0.03
propene	C_3H_6	9.73	-7.05	-7.36	-9.73	0.10	0.09	0.00
tetrachloroethylene	C_2Cl_4	9.33	-7.03	-7.33	-9.58	0.08	0.07	0.01
tetrachloromethane	CCl_4	11.47	-8.75	-9.13	-11.59	0.10	0.09	0.00
tetrafluoroethylene	C_2F_4	10.14	-7.36	-7.63	-10.02	0.10	0.09	0.00
thiophene	C_4H_4S	8.86	-6.55	-6.86	-9.09	0.09	0.07	0.01
toluene	C_7H_8	8.83	-6.65	-6.95	-9.10	0.08	0.07	0.01
trichloromethane	$CHCl_3$	11.37	-8.55	-8.92	-11.37	0.10	0.09	0.00
trifluoromethane	CHF_3	13.86	-11.11	-11.43	-13.96	0.10	0.09	0.00
tetrafluoromethane	CF_4	16.20	-12.26	-12.67	-15.20	0.14	0.13	0.04

4.2.2 Evaluation of mean absolute deviation in a grid of C_{OS} and C_{SS} values

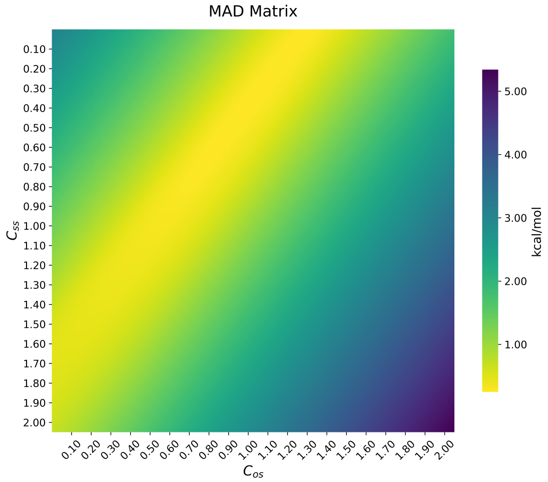

(a)

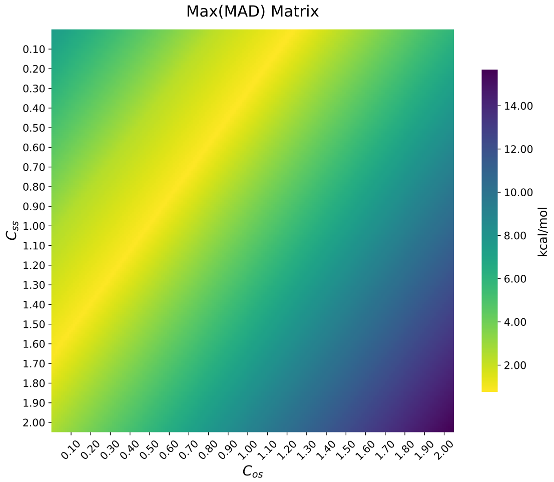

(b)

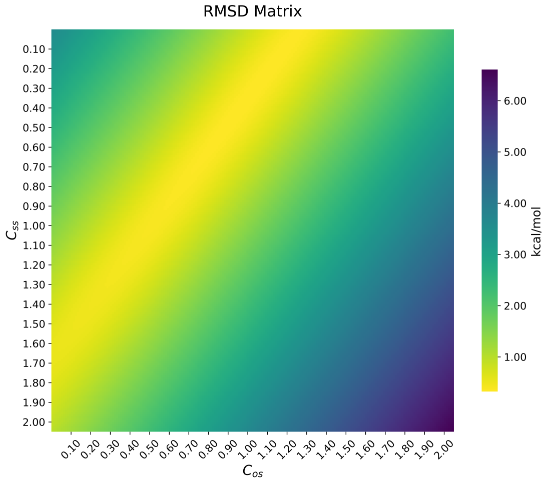

(c)

Figure S8: Evaluation of MADs (S8a), Max(MADs) (S8b) and RMSDs (S8c) in grid of values C_{OS} and C_{SS}. The optimal coefficients are C_{OS}=0.27 and C_{SS}=1.38. The theory level used is RIJK-MP2/aug-cc-pVDZ.

4.2.3 Interaction energies

MP2 with C_{OS} y C_{SS} values

Table S38: Interaction energies for CCSD(T)/CBS, MP2, SCS-MP2, SCS(MI)-MP2, SCSN-MP2, SCS-MP2-vdW, RI-MP2, RI-SCS-MP2, RIJK-MP2, RIJK-SCS-MP2, RIJCOSX-MP2, RIJCOSX-SCS-MP2, SCS-MP2^{BWI-TZ}, RI-SCS-MP2^{BWI-TZ}, RIJK-SCS-MP2^{BWI-TZ}, RIJCOSX-SCS-MP2^{BWI-DZ} and SCS-MP2-hal^{G-XZ}. Data: 30 interaction energies. MP2: $C_{OS} = C_{SS}$ =1.00; SCS-MP2: C_{OS} =1.20, C_{SS} =0.33; SCS(MI)-MP2: C_{OS} =0.40, C_{SS} =1.29; SCSN-MP2: C_{OS} =0.00, C_{SS} =1.76; SCS-MP2-vdW: C_{OS} =1.28, C_{SS} =0.50; SCS-MP2^{BWI-TZ} RI-SCS-MP2^{BWI-TZ} , RIJK-SCS-MP2^{BWI-TZ} : C_{OS} =0.27, C_{SS} =1.38; RIJCOSX-SCS-MP2^{BWI-TZ} : C_{OS} =0.17, C_{SS} =1.59. All values in kcal/mol.

Dimer	Formula	CCSD(T)/CBS	MP2	SCS-MP2	SCS(MI)-MP2	SCSN-MP2	SCS-MP2-vdW	RI-MP2	RI-SCS-MP2	RIJK-MP2	RIJK-SCS-MP2	RIJCOSX-MP2	RIJCOSX-SCS-MP2	SCS-MP2^{BWI-TZ}	RI-SCS-MP2^{BWI-TZ}	RIJK-SCS-MP2^{BWI-TZ}	RIJCOSX-SCS-MP2^{BWI-TZ}	SCS-MP2-hal^{G-XZ}
1 − 3 − 5 − *triazine − tetrachloroethylene*	$C_3H_3N_3$	-4.31	-7.30	-5.47	-5.53	-5.51	-6.58	-7.30	-5.47	-7.29	-5.46	-7.28	-5.45	-5.26	-5.26	-5.25	-5.27	-4.92
1 − 3 − 5 − *triazine − tetrafluoroethylene*	$C_3H_3N_3$	-2.66	-4.44	-3.39	-3.43	-3.43	-4.03	-4.44	-3.39	-4.43	-3.39	-4.43	-3.38	-3.28	-3.28	-3.28	-3.30	-3.08
borazine − difluoromethane	$N_3B_3H_6$	-2.37	-2.99	-2.37	-2.10	-1.96	-2.82	-2.99	-2.37	-2.99	-2.37	-2.96	-2.34	-1.96	-1.96	-1.96	-1.94	-2.16
borazine − hexafluorobenzene	$N_3B_3H_6$	-5.42	-8.99	-7.05	-6.61	-6.33	-8.37	-8.99	-7.05	-9.01	-7.08	-8.95	-7.01	-6.22	-6.22	-6.24	-6.21	-6.44
borazine − tetrachloromethane	$N_3B_3H_6$	-2.98	-4.15	-3.04	-2.69	-2.48	-3.82	-4.15	-3.04	-4.14	-3.04	-4.11	-3.01	-2.45	-2.45	-2.44	-2.43	-2.68
butadyine − chlorine	C_4H_2	-1.43	-1.60	-1.15	-1.83	-2.17	-1.25	-1.60	-1.15	-1.57	-1.12	-1.59	-1.14	-1.90	-1.90	-1.87	-1.91	-1.06
carbondioxide − tetrachloroethylene	CO_2	-2.50	-2.99	-2.47	-2.91	-3.12	-2.68	-2.99	-2.47	-2.97	-2.45	-2.99	-2.47	-2.91	-2.91	-2.89	-2.93	-2.35
carbondioxide − tetrafluoroethylene	CO_2	-1.73	-1.75	-1.49	-1.89	-2.09	-1.55	-1.75	-1.49	-1.73	-1.48	-1.73	-1.47	-1.94	-1.94	-1.92	-1.92	-1.44
cyclohexane − chloromethane	C_6H_{12}	-2.15	-2.85	-2.28	-1.86	-1.64	-2.74	-2.85	-2.28	-2.85	-2.27	-2.82	-2.25	-1.69	-1.69	-1.69	-1.67	-2.07
ethyne − tetrachloroethylene	C_2H_2	-2.64	-3.71	-2.92	-3.15	-3.24	-3.35	-3.71	-2.92	-3.70	-2.91	-3.73	-2.94	-3.07	-3.07	-3.06	-3.11	-2.70
fluorine − trifluoromethane	F_2	-0.63	-0.83	-0.68	-0.32	-0.14	-0.87	-0.83	-0.68	-0.83	-0.68	-0.83	-0.68	-0.23	-0.23	-0.22	-0.22	-0.61
formaldehyde − tetrachloromethane	CH_2O	-1.78	-2.04	-1.61	-1.46	-1.37	-1.92	-2.04	-1.61	-2.03	-1.60	-2.03	-1.60	-1.36	-1.36	-1.35	-1.36	-1.47
formaldehyde − trifluoromethane	CH_2O	-1.29	-1.34	-1.17	-1.07	-1.01	-1.30	-1.34	-1.17	-1.33	-1.16	-1.32	-1.15	-1.02	-1.02	-1.01	-1.00	-1.11
hydrogen − hexafluorobenzene	H_2	-0.71	-1.06	-0.86	-0.71	-0.62	-1.03	-1.06	-0.86	-1.07	-0.87	-1.03	-0.83	-0.65	-0.65	-0.65	-0.61	-0.79
hydrogen − tetrachloromethane	H_2	-0.48	-0.54	-0.50	-0.07	0.15	-0.63	-0.54	-0.50	-0.53	-0.50	-0.52	-0.48	0.02	0.02	0.03	0.04	-0.46
methylamine − dichloromethane	CH_3NH_2	-2.06	-2.60	-1.97	-1.73	-1.59	-2.42	-2.60	-1.97	-2.59	-1.96	-2.59	-1.96	-1.58	-1.58	-1.58	-1.58	-1.76
methylamine − trifluoromethane	CH_3NH_2	-1.34	-1.62	-1.35	-0.97	-0.78	-1.61	-1.62	-1.35	-1.62	-1.34	-1.61	-1.33	-0.86	-0.86	-0.85	-0.84	-1.23
neohexane − chlorine	C_6H_{14}	-1.69	-2.63	-2.19	-1.57	-1.25	-2.62	-2.63	-2.19	-2.63	-2.18	-2.63	-2.18	-1.38	-1.38	-1.38	-1.38	-2.00
nitrogen − chlorine	N_2	-0.73	-0.20	-0.19	-1.11	-1.58	0.04	-0.20	-0.19	-0.18	-0.17	-0.18	-0.17	-1.30	-1.30	-1.28	-1.29	-0.26
nonbornadiene − chlorine	C_7H_8	-2.14	-3.34	-2.49	-2.31	-2.21	-3.06	-3.34	-2.49	-3.34	-2.48	-3.36	-2.51	-2.15	-2.15	-2.14	-2.18	-2.22
phosphine − hexafluorobenzene	PH_3	-2.65	-4.30	-3.32	-3.04	-2.88	-4.00	-4.30	-3.32	-4.30	-3.32	-4.26	-3.28	-2.84	-2.84	-2.84	-2.81	-3.00
phosphorine − chlorine	PC_5H_5	-2.25	-3.84	-2.71	-2.73	-2.72	-3.40	-3.84	-2.71	-3.84	-2.70	-3.83	-2.70	-2.56	-2.56	-2.56	-2.57	-2.36
phosphorine − tetrafluoromethane	PC_5H_5	-1.75	-2.67	-1.99	-2.08	-2.12	-2.38	-2.67	-1.99	-2.66	-1.98	-2.67	-1.98	-2.00	-2.00	-1.99	-2.01	-1.79
propane − difluoromethane	C_3H_8	-1.68	-2.14	-1.72	-1.30	-1.08	-2.09	-2.14	-1.72	-2.14	-1.71	-2.13	-1.70	-1.15	-1.15	-1.15	-1.14	-1.56
propene − tetrachloroethylene	C_3H_6	-3.67	-5.81	-4.51	-4.21	-4.03	-5.39	-5.81	-4.51	-5.81	-4.50	-5.81	-4.50	-3.95	-3.95	-3.95	-3.97	-4.09
tetrafluoromethane − difluoromethane	CF_4	-1.33	-1.62	-1.38	-1.10	-0.96	-1.60	-1.62	-1.38	-1.62	-1.37	-1.62	-1.37	-1.01	-1.01	-1.01	-1.01	-1.28
thiophene − fluoromethane	C_4H_4S	-2.52	-3.36	-2.86	-2.54	-2.37	-3.25	-3.36	-2.86	-3.36	-2.86	-3.33	-2.83	-2.40	-2.40	-2.40	-2.37	-2.69
thiophene − hexafluorobenzene	C_4H_4S	-5.83	-9.88	-7.97	-7.43	-7.12	-9.29	-9.88	-7.97	-9.90	-7.99	-9.78	-7.87	-7.03	-7.03	-7.05	-6.97	-7.35
thiophene − trichloromethane	C_4H_4S	-2.93	-4.52	-3.37	-3.04	-2.84	-4.17	-4.52	-3.37	-4.51	-3.37	-4.50	-3.36	-2.80	-2.80	-2.79	-2.80	-3.00
toluene − trifluoromethane	$C_6H_5CH_3$	-1.73	-2.71	-2.02	-1.71	-1.54	-2.53	-2.71	-2.02	-2.71	-2.02	-2.69	-2.00	-1.54	-1.54	-1.54	-1.53	-1.78

DFT

Table S39: Interaction energies for CCSD(T)/CBS, B97M-V, ωB97X-V, ωB97M-V, ωB97X-D3, B2PLYP-D3BJ, DSD-BLYP-D3BJ, ωB97X-D4 and B2PLYP-D4. Data: 30 interaction energies. All values in kcal/mol.

Dimer	Formula	CCSD(T)/CBS	B97M-V	ωB97X-V	ωB97M-V	ωB97X-D3	B2PLYP-D3BJ	DSD-BLYP-D3BJ	ωB97X-D4	B2PLYP-D4
1 − 3 − 5 − *triazine* − *tetrachloroethylene*	$C_3H_3N_3{-}C_2Cl_4$	-4.31	-4.19	-4.62	-4.89	-4.79	-5.15	-5.35	-4.69	-5.28
1 − 3 − 5 − *triazine* − *tetrafluoroethylene*	$C_3H_3N_3{-}C_2F_4$	-2.66	-2.91	-3.33	-3.40	-2.95	-3.12	-3.30	-3.04	-3.30
borazine − *difluoromethane*	$N_3B_3H_6{-}CH_2F_2$	-2.37	-2.33	-2.57	-2.65	-2.66	-2.51	-2.53	-2.76	-2.44
borazine − *hexafluorobenzene*	$N_3B_3H_6{-}C_6F_6$	-5.42	-6.01	-6.35	-6.84	-6.33	-6.47	-6.77	-5.83	-6.68
borazine − *tetrachloromethane*	$N_3B_3H_6{-}Cl_2$	-2.98	-3.10	-2.94	-3.27	-3.32	-3.31	-3.23	-3.21	-3.35
butadyine − *chlorine*	$C_4H_2{-}Cl_2$	-1.43	-1.93	-2.09	-2.28	-2.34	-1.70	-1.66	-2.65	-1.66
carbondioxide − *tetrachloroethylene*	$CO_2{-}C_2Cl_4$	-2.50	-2.73	-2.92	-3.11	-2.71	-2.76	-2.83	-3.11	-2.78
carbondioxide − *tetrafluoroethylene*	$CO_2{-}C_2F_4$	-1.73	-2.01	-2.15	-2.16	-1.71	-1.71	-1.77	-2.13	-1.77
cyclohexane − *chloromethane*	$C_6H_{12}{-}CH_3Cl$	-2.15	-1.90	-2.18	-2.12	-2.31	-2.30	-2.29	-2.56	-2.17
ethyne − *tetrachloroethylene*	$C_2H_2{-}C_2Cl_4$	-2.64	-2.78	-3.16	-3.39	-3.42	-3.09	-3.12	-3.64	-3.05
fluorine − *trifluoromethane*	$F_2{-}CHF_3$	-0.63	-0.58	-0.76	-0.68	-0.12	-0.65	-0.64	-0.49	-0.72
formaldehyde − *tetrachloromethane*	$CH_2O{-}CHF_3$	-1.78	-1.92	-1.73	-1.82	-1.97	-2.02	-1.89	-2.13	-1.88
formaldehyde − *trifluoromethane*	$CH_2O{-}CF_4$	-1.29	-1.30	-1.63	-1.50	-1.46	-1.32	-1.29	-1.63	-1.31
hydrogen − *hexafluorobenzene*	$H_2{-}C_6F_6$	-0.71	-0.69	-0.86	-0.87	-0.91	-0.81	-0.84	-1.08	-0.77
hydrogen − *tetrachloromethane*	$H_2{-}CCl_4$	-0.48	-0.38	-0.38	-0.39	-0.57	-0.57	-0.46	-0.64	-0.53
methylamine − *dichloromethane*	$CH_3NH_2{-}CH_2Cl_2$	-2.06	-1.93	-2.04	-2.10	-2.33	-2.20	-2.16	-2.52	-2.11
methylamine − *trifluoromethane*	$CH_3NH_2{-}CHF_3$	-1.34	-1.26	-1.74	-1.55	-1.47	-1.44	-1.40	-1.64	-1.46
neohexane − *chlorine*	$C_6H_{14}{-}Cl_2$	-1.69	-1.56	-1.78	-1.71	-2.03	-2.01	-2.02	-2.01	-1.77
nitrogen − *chlorine*	$N_2{-}Cl_2$	-0.73	-1.57	-1.56	-1.70	-1.64	-1.01	-0.89	-1.92	-0.96
nonbornadiene − *chlorine*	$C_7H_8{-}Cl_2$	-2.14	-2.23	-2.42	-2.60	-2.71	-2.63	-2.62	-2.73	-2.52
phosphine − *hexafluorobenzene*	$PH_3{-}C_6F_6$	-2.65	-2.58	-2.85	-3.01	-2.71	-3.18	-3.28	-3.05	-3.10
phosphorine − *chlorine*	$PC_5H_5{-}Cl_2$	-2.25	-2.22	-2.17	-2.46	-2.42	-2.65	-2.75	-2.60	-2.55
phosphorine − *tetrafluoromethane*	$PC_5H_5{-}CF_4$	-1.75	-2.05	-2.33	-2.39	-1.97	-1.84	-1.96	-2.14	-1.98
propane − *difluoromethane*	$C_3H_8{-}CH_2F_2$	-1.68	-1.52	-1.97	-1.79	-1.68	-1.73	-1.73	-1.99	-1.66
propene − *tetrachloroethylene*	$C_3H_6{-}C_2Cl_4$	-3.67	-3.59	-3.98	-4.22	-4.39	-4.33	-4.43	-4.27	-4.18
tetrafluoromethane − *difluoromethane*	$CF_4{-}CH_2F_2$	-1.33	-1.31	-1.68	-1.55	-0.98	-1.31	-1.35	-1.33	-1.43
thiophene − *fluoromethane*	$C_4H_4S{-}CF_4$	-2.52	-2.61	-2.90	-3.01	-2.95	-2.84	-2.89	-3.25	-2.77
thiophene − *hexafluorobenzene*	$C_4H_4S{-}C_6F_6$	-5.83	-5.95	-6.48	-6.96	-6.22	-7.11	-7.43	-6.02	-7.21
thiophene − *trichloromethane*	$C_4H_4S{-}CHCl_3$	-2.93	-2.97	-3.02	-3.32	-3.25	-3.46	-3.44	-3.35	-3.45
toluene − *trifluoromethane*	$C_6H_5CH_3{-}CHF_3$	-1.73	-1.98	-2.36	-2.31	-1.89	-1.82	-1.90	-2.02	-1.95

SAPT-DFT

Table S40: Interaction energies for CCSD(T)/CBS and SAPT-DFT. For SAPT-DFT method we used the B3LYP, PBE0 and ωB97X DFA's in conjunction with the aug-cc-pVDZ basis set. Data: 30 interaction energies. All values in kcal/mol.

Dimer	Formula	CCSD(T)/CBS	SAPT-B3LYP	SAPT-PBE0	SAPT-ωB97X
$1-3-5-triazine-tetrachloroethylene$	$C_3H_3N_3{-}C_2Cl_4$	-4.31	-2.83	-2.81	-2.23
$1-3-5-triazine-tetrafluoroethylene$	$C_3H_3N_3{-}C_2F_4$	-2.66	-1.74	-1.76	-1.40
$borazine-difluoromethane$	$N_3B_3H_6{-}CH_2F_2$	-2.37	-1.69	-1.74	-1.39
$borazine-hexafluorobenzene$	$N_3B_3H_6{-}C_6F_6$	-5.42	-3.73	-3.68	-3.01
$borazine-tetrachloromethane$	$N_3B_3H_6{-}Cl_2$	-2.98	-2.03	-1.93	-1.29
$butadyine-chlorine$	$C_4H_2{-}Cl_2$	-1.43	-0.97	-0.92	-0.68
$carbondioxide-tetrachloroethylene$	$CO_2{-}C_2Cl_4$	-2.50	-1.74	-1.68	-1.34
$carbondioxide-tetrafluoroethylene$	$CO_2{-}C_2F_4$	-1.73	-1.26	-1.19	-0.96
$cyclohexane-chloromethane$	$C_6H_{12}{-}CH_3Cl$	-2.15	-1.62	-1.57	-1.14
$ethyne-tetrachloroethylene$	$C_2H_2{-}C_2Cl_4$	-2.64	-2.02	-1.92	-1.46
$fluorine-trifluoromethane$	$F_2{-}CHF_3$	-0.63	-0.36	-0.36	-0.25
$formaldehyde-tetrachloromethane$	$CH_2O{-}CHF_3$	-1.78	-1.28	-1.26	-0.87
$formaldehyde-trifluoromethane$	$CH_2O{-}CF_4$	-1.29	-1.03	-1.01	-0.86
$methylamine-dichloromethane$	$CH_3NH_2{-}CH_2Cl_2$	-2.06	-1.44	-1.38	-0.96
$methylamine-trifluoromethane$	$CH_3NH_2{-}CHF_3$	-1.34	-0.89	-0.87	-0.70
$neohexane-chlorine$	$C_6H_{14}{-}Cl_2$	-1.69	-1.19	-1.13	-0.80
$nitrogen-chlorine$	$N_2{-}Cl_2$	-0.73	-0.48	-0.47	-0.26
$nonbornadiene-chlorine$	$C_7H_8{-}Cl_2$	-2.14	-1.53	-1.45	-0.87
$phosphine-hexafluorobenzene$	$PH_3{-}C_6F_6$	-2.65	-1.94	-1.88	-1.52
$phosphorine-chlorine$	$PC_5H_5{-}Cl_2$	-2.25	-1.62	-1.55	-1.00
$phosphorine-tetrafluoromethane$	$PC_5H_5{-}CF_4$	-1.75	-1.27	-1.24	-0.91
$propane-difluoromethane$	$C_3H_8{-}CH_2F_2$	-1.68	-1.18	-1.18	-0.89
$propene-tetrachloroethylene$	$C_3H_6{-}C_2Cl_4$	-3.67	-2.68	-2.59	-1.84
$tetrafluoromethane-difluoromethane$	$CF_4{-}CH_2F_2$	-1.33	-0.97	-0.93	-0.70
$thiophene-fluoromethane$	$C_4H_4S{-}CF_4$	-2.52	-1.96	-1.98	-1.57
$thiophene-hexafluorobenzene$	$C_4H_4S{-}C_6F_6$	-5.83	-4.39	-4.30	-3.59
$thiophene-trichloromethane$	$C_4H_4S{-}CHCl_3$	-2.93	-2.04	-1.94	-1.18
$toluene-trifluoromethane$	$C_6H_5CH_3{-}CHF_3$	-1.73	-1.25	-1.19	-0.80

4.2.4 Absolute deviations of molecular systems.

MP2 with C_{OS} y C_{SS} values

Table S41: Absolute deviations for MP2, SCS-MP2, SCS(MI)-MP2, SCSN-MP2, SCS-MP2-vdW, RI-MP2, RI-SCS-MP2, RIJK-MP2, RIJK-SCS-MP2, RIJCOSX-MP2, RIJCOSX-SCS-MP2, SCS-MP2^{BWI-TZ}, RI-SCS-MP2^{BWI-TZ}, RIJK-SCS-MP2^{BWI-TZ}, RIJCOSX-SCS-MP2^{BWI-DZ} and SCS-MP2-hal^{G-XZ}. Data: 30 interaction energies. MP2: C_{OS} = C_{SS} =1.00; SCS-MP2: C_{OS} =1.20, C_{SS} =0.33; SCS(MI)-MP2: C_{OS} =0.40, C_{SS} =1.29; SCSN-MP2: C_{OS} =0.00, C_{SS} =1.76; SCS-MP2-vdW: C_{OS} =1.28, C_{SS} =0.50; SCS-MP2^{BWI-TZ} RI-SCS-MP2^{BWI-TZ} , RIJK-SCS-MP2^{BWI-TZ} : C_{OS} =0.27, C_{SS} =1.38; RIJCOSX-SCS-MP2^{BWI-TZ} : C_{OS} =0.17, C_{SS} =1.59. All values in kcal/mol.

Dimer	Formula	MP2	SCS-MP2	SCS(MI)-MP2	SCSN-MP2	SCS-MP2-vdW	RI-MP2	RI-SCS-MP2	RIJK-MP2	RIJK-SCS-MP2	RIJCOSX-MP2	RIJCOSX-SCS-MP2	SCS-MP2^{BWI-TZ}	RI-SCS-MP2^{BWI-TZ}	RIJK-SCS-MP2^{BWI-TZ}	RIJCOSX-SCS-MP2^{BWI-TZ}	SCS-MP2-hal^{G-XZ}
$1-3-5-triazine-tetrachloroethylene$	$C_3H_3N_3$	2.99	1.16	1.22	1.20	2.27	2.99	1.16	2.98	1.15	2.97	1.14	0.95	0.95	0.94	0.96	0.61
$1-3-5-triazine-tetrafluoroethylene$	$C_3H_3N_3$	1.78	0.73	0.77	0.77	1.37	1.78	0.73	1.77	0.73	1.77	0.72	0.62	0.62	0.62	0.64	0.42
$borazine-difluoromethane$	$N_3B_3H_6$	0.62	0.00	0.27	0.41	0.45	0.62	0.00	0.62	0.00	0.59	0.03	0.41	0.41	0.41	0.43	0.21
$borazine-hexafluorobenzene$	$N_3B_3H_6$	3.57	1.63	1.19	0.91	2.95	3.57	1.63	3.59	1.66	3.53	1.59	0.80	0.80	0.82	0.79	1.02
$borazine-tetrachloromethane$	$N_3B_3H_6$	1.17	0.06	0.29	0.50	0.84	1.17	0.06	1.16	0.06	1.13	0.03	0.53	0.53	0.54	0.55	0.30
$butadyine-chlorine$	C_4H_2	0.17	0.28	0.40	0.74	0.18	0.17	0.28	0.14	0.31	0.16	0.29	0.47	0.47	0.44	0.48	0.37
$carbondioxide-tetrachloroethylene$	CO_2	0.49	0.03	0.41	0.62	0.18	0.49	0.03	0.47	0.05	0.49	0.03	0.41	0.41	0.39	0.43	0.15
$carbondioxide-tetrafluoroethylene$	CO_2	0.02	0.24	0.16	0.36	0.18	0.02	0.24	0.00	0.25	0.00	0.26	0.21	0.21	0.19	0.19	0.29
$cyclohexane-chloromethane$	C_6H_{12}	0.70	0.13	0.29	0.51	0.59	0.70	0.13	0.70	0.12	0.67	0.10	0.46	0.46	0.46	0.48	0.08
$ethyne-tetrachloroethylene$	C_2H_2	1.07	0.28	0.51	0.60	0.71	1.07	0.28	1.06	0.27	1.09	0.30	0.43	0.43	0.42	0.47	0.06
$fluorine-trifluoromethane$	F_2	0.20	0.05	0.31	0.49	0.24	0.20	0.05	0.20	0.05	0.20	0.05	0.40	0.40	0.41	0.41	0.02
$formaldehyde-tetrachloromethane$	CH_2O	0.26	0.17	0.32	0.41	0.14	0.26	0.17	0.25	0.18	0.25	0.18	0.42	0.42	0.43	0.42	0.31
$formaldehyde-trifluoromethane$	CH_2O	0.05	0.12	0.22	0.28	0.01	0.05	0.12	0.04	0.13	0.03	0.14	0.27	0.27	0.28	0.29	0.18
$hydrogen-hexafluorobenzene$	H_2	0.35	0.15	0.00	0.09	0.32	0.35	0.15	0.36	0.16	0.32	0.12	0.06	0.06	0.06	0.10	0.08
$hydrogen-tetrachloromethane$	H_2	0.06	0.02	0.41	0.63	0.15	0.06	0.02	0.05	0.02	0.04	0.00	0.50	0.50	0.51	0.52	0.02
$methylamine-dichloromethane$	CH_3NH_2	0.54	0.09	0.33	0.47	0.36	0.54	0.09	0.53	0.10	0.53	0.10	0.48	0.48	0.48	0.48	0.30
$methylamine-trifluoromethane$	CH_3NH_2	0.28	0.01	0.37	0.56	0.27	0.28	0.01	0.28	0.00	0.27	0.01	0.48	0.48	0.49	0.50	0.11
$neohexane-chlorine$	C_6H_{14}	0.94	0.50	0.12	0.44	0.93	0.94	0.50	0.94	0.49	0.94	0.49	0.31	0.31	0.31	0.31	0.31
$nitrogen-chlorine$	N_2	0.53	0.54	0.38	0.85	0.77	0.53	0.54	0.55	0.56	0.55	0.56	0.57	0.57	0.55	0.56	0.47
$nonbornadiene-chlorine$	C_7H_8	1.20	0.35	0.17	0.07	0.92	1.20	0.35	1.20	0.34	1.22	0.37	0.01	0.01	0.00	0.04	0.08
$phosphine-hexafluorobenzene$	PH_3	1.65	0.67	0.39	0.23	1.35	1.65	0.67	1.65	0.67	1.61	0.63	0.19	0.19	0.19	0.16	0.35
$phosphorine-chlorine$	PC_5H_5	1.59	0.46	0.48	0.47	1.15	1.59	0.46	1.59	0.45	1.58	0.45	0.31	0.31	0.31	0.32	0.11
$phosphorine-tetrafluoromethane$	PC_5H_5	0.92	0.24	0.33	0.37	0.63	0.92	0.24	0.91	0.23	0.92	0.23	0.25	0.25	0.24	0.26	0.04
$propane-difluoromethane$	C_3H_8	0.46	0.04	0.38	0.60	0.41	0.46	0.04	0.46	0.03	0.45	0.02	0.53	0.53	0.53	0.54	0.12
$propene-tetrachloroethylene$	C_3H_6	2.14	0.84	0.54	0.36	1.72	2.14	0.84	2.14	0.83	2.14	0.83	0.28	0.28	0.28	0.30	0.42
$tetrafluoromethane-difluoromethane$	CF_4	0.29	0.05	0.23	0.37	0.27	0.29	0.05	0.29	0.04	0.29	0.04	0.32	0.32	0.32	0.32	0.05
$thiophene-fluoromethane$	C_4H_4S	0.84	0.34	0.02	0.15	0.73	0.84	0.34	0.84	0.34	0.81	0.31	0.12	0.12	0.12	0.15	0.17
$thiophene-hexafluorobenzene$	C_4H_4S	4.05	2.14	1.60	1.29	3.46	4.05	2.14	4.07	2.16	3.95	2.04	1.20	1.20	1.22	1.14	1.52
$thiophene-trichloromethane$	C_4H_4S	1.59	0.44	0.11	0.09	1.24	1.59	0.44	1.58	0.44	1.57	0.43	0.13	0.13	0.14	0.13	0.07
$toluene-trifluoromethane$	$C_6H_5CH_3$	0.98	0.29	0.02	0.19	0.80	0.98	0.29	0.98	0.29	0.96	0.27	0.19	0.19	0.19	0.20	0.05

DFT

Table S42: Absolute deviations for B97M-V, ωB97X-V, ωB97M-V, ωB97X-D3, B2PLYP-D3BJ, DSD-BLYP-D3BJ, ωB97X-D4 and B2PLYP-D4. Data: 30 interaction energies. All values in kcal/mol.

Dimer	Formula	B97M-V	ωB97X-V	ωB97M-V	ωB97X-D3	B2PLYP-D3BJ	DSD-BLYP-D3BJ	ωB97X-D4	B2PLYP-D4
1 − 3 − 5 − *triazine − tetrachloroethylene*	$C_3H_3N_3{-}C_2Cl_4$	0.12	0.31	0.58	0.48	0.84	1.04	0.38	0.97
1 − 3 − 5 − *triazine − tetrafluoroethylene*	$C_3H_3N_3{-}C_2F_4$	0.25	0.67	0.74	0.29	0.46	0.64	0.38	0.64
borazine − difluoromethane	$N_3B_3H_6{-}CH_2F_2$	0.04	0.20	0.28	0.29	0.14	0.16	0.39	0.07
borazine − hexafluorobenzene	$N_3B_3H_6{-}C_6F_6$	0.59	0.93	1.42	0.91	1.05	1.35	0.41	1.26
borazine − tetrachloromethane	$N_3B_3H_6{-}Cl_2$	0.12	0.04	0.29	0.34	0.33	0.25	0.23	0.37
butadyine − chlorine	$C_4H_2{-}Cl_2$	0.50	0.66	0.85	0.91	0.27	0.23	1.22	0.23
carbondioxide − tetrachloroethylene	$CO_2{-}C_2Cl_4$	0.23	0.42	0.61	0.21	0.26	0.33	0.61	0.28
carbondioxide − tetrafluoroethylene	$CO_2{-}C_2F_4$	0.28	0.42	0.43	0.02	0.02	0.04	0.40	0.04
cyclohexane − chloromethane	$C_6H_{12}{-}CH_3Cl$	0.25	0.03	0.03	0.16	0.15	0.14	0.41	0.02
ethyne − tetrachloroethylene	$C_2H_2{-}C_2Cl_4$	0.14	0.52	0.75	0.78	0.45	0.48	1.00	0.41
fluorine − trifluoromethane	$F_2{-}CHF_3$	0.05	0.13	0.05	0.51	0.02	0.01	0.14	0.09
formaldehyde − tetrachloromethane	$CH_2O{-}CHF_3$	0.14	0.05	0.04	0.19	0.24	0.11	0.35	0.10
formaldehyde − trifluoromethane	$CH_2O{-}CF_4$	0.01	0.34	0.21	0.17	0.03	0.00	0.34	0.02
hydrogen − hexafluorobenzene	$H_2{-}C_6F_6$	0.02	0.15	0.16	0.20	0.10	0.13	0.37	0.06
hydrogen − tetrachloromethane	$H_2{-}CCl_4$	0.10	0.10	0.09	0.09	0.09	0.02	0.16	0.05
methylamine − dichloromethane	$CH_3NH_2{-}CH_2Cl_2$	0.13	0.02	0.04	0.27	0.14	0.10	0.46	0.05
methylamine − trifluoromethane	$CH_3NH_2{-}CHF_3$	0.08	0.40	0.21	0.13	0.10	0.06	0.30	0.12
neohexane − chlorine	$C_6H_{14}{-}Cl_2$	0.13	0.09	0.02	0.34	0.32	0.33	0.32	0.08
nitrogen − chlorine	$N_2{-}Cl_2$	0.84	0.83	0.97	0.91	0.28	0.16	1.19	0.23
nonbornadiene − chlorine	$C_7H_8{-}Cl_2$	0.09	0.28	0.46	0.57	0.49	0.48	0.59	0.38
phosphine − hexafluorobenzene	$PH_3{-}C_6F_6$	0.07	0.20	0.36	0.06	0.53	0.63	0.40	0.45
phosphorine − chlorine	$PC_5H_5{-}Cl_2$	0.03	0.08	0.21	0.17	0.40	0.50	0.35	0.30
phosphorine − tetrafluoromethane	$PC_5H_5{-}CF_4$	0.30	0.58	0.64	0.22	0.09	0.21	0.39	0.23
propane − difluoromethane	$C_3H_8{-}CH_2F_2$	0.16	0.29	0.11	0.00	0.05	0.05	0.31	0.02
propene − tetrachloroethylene	$C_3H_6{-}C_2Cl_4$	0.08	0.31	0.55	0.72	0.66	0.76	0.60	0.51
tetrafluoromethane − difluoromethane	$CF_4{-}CH_2F_2$	0.02	0.35	0.22	0.35	0.02	0.02	0.00	0.10
thiophene − fluoromethane	$C_4H_4S{-}CF_4$	0.09	0.38	0.49	0.43	0.32	0.37	0.73	0.25
thiophene − hexafluorobenzene	$C_4H_4S{-}C_6F_6$	0.12	0.65	1.13	0.39	1.28	1.60	0.19	1.38
thiophene − trichloromethane	$C_4H_4S{-}CHCl_3$	0.04	0.09	0.39	0.32	0.53	0.51	0.42	0.52
toluene − trifluoromethane	$C_6H_5CH_3{-}CHF_3$	0.25	0.63	0.58	0.16	0.09	0.17	0.29	0.22

SAPT-DFT

Table S43: Absolute deviations for SAPT-DFT with respect to CCSD(T)/CBS. In SAPT-DFT method we used the B3LYP, PBE0 and ωB97X DFA's in conjunction with the aug-cc-pVTZ basis set. Data: 30 interaction energies. All values in kcal/mol.

Dimer	Formula	SAPT-B3LYP	SAPT-PBE0	SAPT-ωB97X
$1-3-5-triazine-tetrachloroethylene$	$C_3H_3N_3$−C_2Cl_4	1.48	1.50	2.08
$1-3-5-triazine-tetrafluoroethylene$	$C_3H_3N_3$−C_2F_4	0.92	0.90	1.26
$borazine-difluoromethane$	$N_3B_3H_6$−CH_2F_2	0.68	0.63	0.98
$borazine-hexafluorobenzene$	$N_3B_3H_6$−C_6F_6	1.69	1.74	2.41
$borazine-tetrachloromethane$	$N_3B_3H_6$−Cl_2	0.95	1.05	1.69
$butadyine-chlorine$	C_4H_2−Cl_2	0.46	0.51	0.75
$carbondioxide-tetrachloroethylene$	CO_2−C_2Cl_4	0.76	0.82	1.16
$carbondioxide-tetrafluoroethylene$	CO_2−C_2F_4	0.47	0.54	0.77
$cyclohexane-chloromethane$	C_6H_{12}−CH_3Cl	0.53	0.58	1.01
$ethyne-tetrachloroethylene$	C_2H_2−C_2Cl_4	0.62	0.72	1.18
$fluorine-trifluoromethane$	F_2−CHF_3	0.27	0.27	0.38
$formaldehyde-tetrachloromethane$	CH_2O−CHF_3	0.50	0.52	0.91
$formaldehyde-trifluoromethane$	CH_2O−CF_4	0.26	0.28	0.43
$methylamine-dichloromethane$	CH_3NH_2−CH_2Cl_2	0.62	0.68	1.10
$methylamine-trifluoromethane$	CH_3NH_2−CHF_3	0.45	0.47	0.64
$neohexane-chlorine$	C_6H_{14}−Cl_2	0.50	0.56	0.89
$nitrogen-chlorine$	N_2−Cl_2	0.25	0.26	0.47
$nonbornadiene-chlorine$	C_7H_8−Cl_2	0.61	0.69	1.27
$phosphine-hexafluorobenzene$	PH_3−C_6F_6	0.71	0.77	1.13
$phosphorine-chlorine$	PC_5H_5−Cl_2	0.63	0.70	1.25
$phosphorine-tetrafluoromethane$	PC_5H_5−CF_4	0.48	0.51	0.84
$propane-difluoromethane$	C_3H_8−CH_2F_2	0.50	0.50	0.79
$propene-tetrachloroethylene$	C_3H_6−C_2Cl_4	0.99	1.08	1.83
$tetrafluoromethane-difluoromethane$	CF_4−CH_2F_2	0.36	0.40	0.63
$thiophene-fluoromethane$	C_4H_4S−CF_4	0.56	0.54	0.95
$thiophene-hexafluorobenzene$	C_4H_4S−C_6F_6	1.44	1.53	2.24
$thiophene-trichloromethane$	C_4H_4S−$CHCl_3$	0.89	0.99	1.75
$toluene-trifluoromethane$	$C_6H_5CH_3$−CHF_3	0.48	0.54	0.93

4.3 Mean calculation times

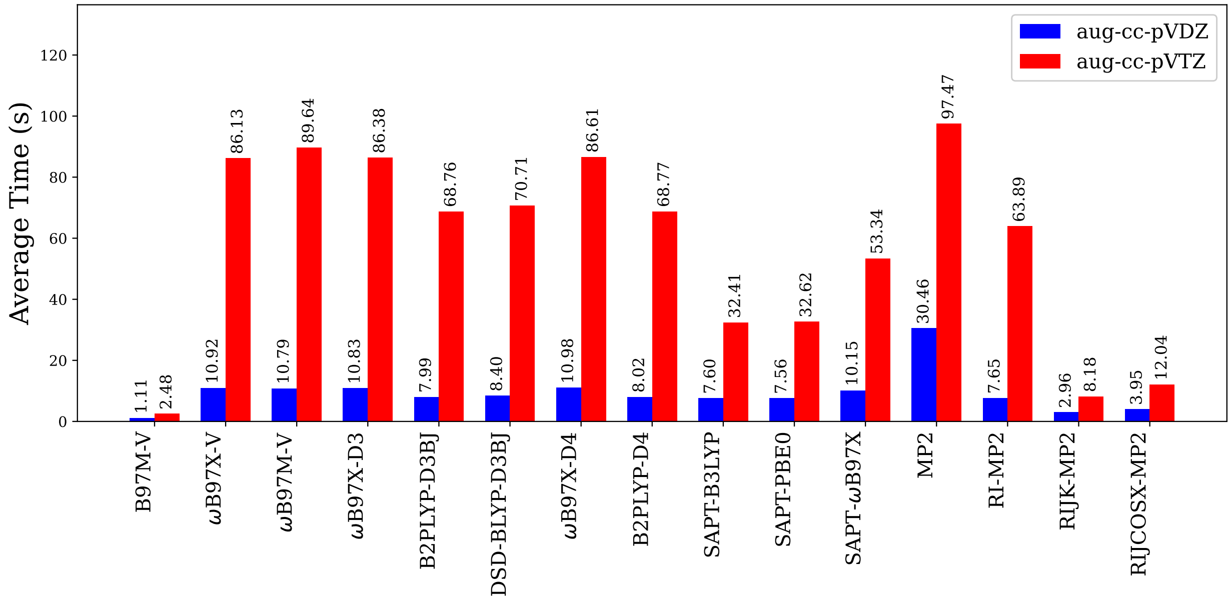

Figure S9: Comparison of computational times for different methods and basis sets in the dataset HB300SPX(Cl,F). All values in seconds.

5 JSCH-2005* dataset

5.1 aug-cc-pVDZ basis set data.

5.1.1 Ionization Potentials and HOMO values for SAPT-DFT calculations

Table S44: Experimental ionization potentials (IP) of molecules comprising dimers in the JSCH-2005* database. IP obtained from NIST (https://webbook.nist.gov/chemistry). Theoretical IP obtained in this work with DLPNO-CCSD(T)/aug-cc-pVDZ (marked with *). Kohn-Sham energies of HOMO orbitals were calculated at the DFA/aug-cc-pVDZ level of theory (DFA=B3LYP, PBE0, and ωB97X). Ionization energies in ev and HOMO energies in atomic units.

			HOMO energies (eV)			$\Delta_{XC} = \epsilon_{HOMO} - (-IP)$		
Molecule	Formula	IP	B3LYP	PBE0	ωB97X	B3LYP	PBE0	ωB97X
$AA(9 - methyl - adenine)$	$C_6H_6N_5CH_3$	7.90	-6.15	-6.42	-8.53	0.06	0.05	0.02
$Aas(Adenine)$	$C_5H_5N_5$	8.30	-6.19	-6.45	-8.56	0.08	0.07	0.01
$ACS(1 - methylcytosine)$	$C_5H_6N_3O$	9.50	-6.35	-6.63	-8.83	0.12	0.11	0.02
$Acst(Cytosine)$	$C_4H_5N_3O$	8.45	-6.52	-6.81	-9.04	0.07	0.06	0.02
$AG(Thymine)$	$C_5H_6N_2O_2$	9.00	-6.85	-7.13	-9.32	0.08	0.07	0.01
$Ags(Guanine)$	$C_5H_5N_5O$	7.85	-5.85	-6.11	-8.19	0.07	0.06	0.01
$Atis(Thymine)$	$C_5H_6N_2O_2$	9.00	-6.85	-7.13	-9.32	0.08	0.07	0.01
$ATS2 * (1 - methylThymine)$	$C_6H_8N_2O_2$	8.81	-6.61	-6.89	-9.05	0.08	0.07	0.01
$Aust(Uracil)$	$C_4H_4N_2O_2$	9.20	-7.20	-7.49	-9.71	0.07	0.06	0.02
$CWC(Cytosine)$	$C_4H_5N_3O$	8.45	-6.53	-6.82	-9.04	0.07	0.06	0.02
$F30 * (metil/fenilalanina)$	$C_9H_{11}NO_2$	9.19	-6.39	-6.68	-9.06	0.10	0.09	0.00
$GWC * (guanine)$	$C_5H_5N_5O$	7.91	-5.79	-6.05	-8.13	0.08	0.07	0.01
$ICWC * (adenine)$	$C_5H_5N_5$	8.68	-6.46	-6.72	-8.81	0.08	0.07	0.00
$K46 * (methyl/lysine)$	$C_6H_{14}N_2O_2$	9.36	-6.40	-6.70	-9.18	0.11	0.10	0.01
$L33 * (methyl/isoleucine)$	$C_6H_{13}NO_2$	9.51	-6.57	-6.87	-9.35	0.11	0.10	0.01
$mGm * (2 - methylguanine)$	$C_6H_7N_5O$	8.03	-5.91	-6.17	-8.26	0.08	0.07	0.01
$V5C6(acetamide)$	C_2H_5NO	9.70	-6.89	-7.24	-9.75	0.10	0.09	0.00
$Y13 * (methyl/Tyrosine)$	$C_9H_{11}NO_3$	8.50	-6.17	-6.45	-8.63	0.09	0.08	0.00

5.1.2 Evaluation of mean absolute deviation in a grid of C_{OS} and C_{SS} values

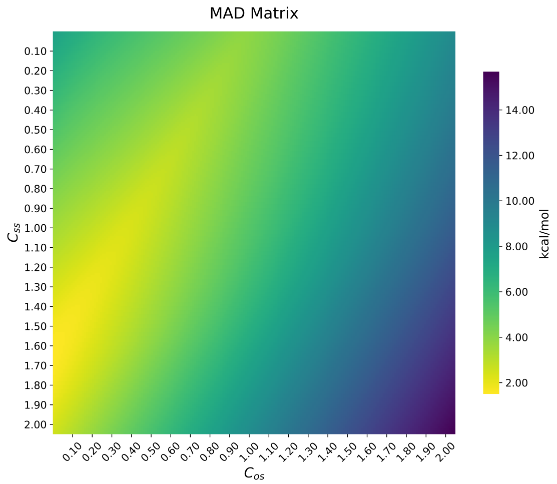

(a)

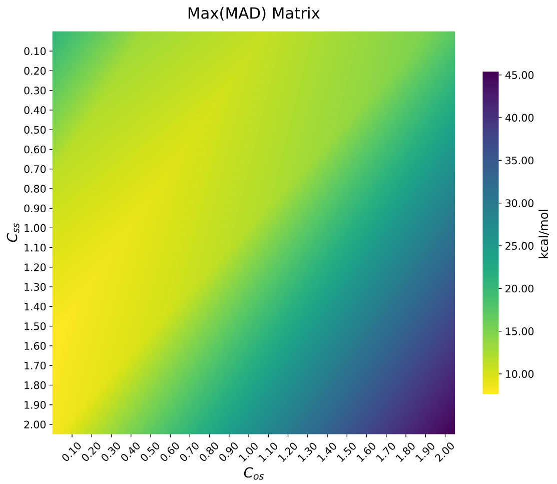

(b)

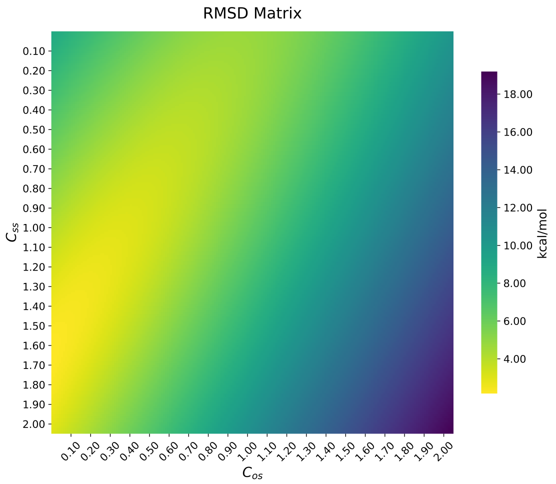

(c)

Figure S10: Evaluation of MADs (S10a), Max(MADs) (S10b) and RMSDs (S10c) in grid of values C_{OS} and C_{SS}. The optimal coefficients are C_{OS}=0.00 and C_{SS}=1.50. The theory level used is RIJK-MP2/aug-cc-pVDZ.

5.1.3 Interaction energies

MP2 with C_{OS} y C_{SS} values

Table S45: Interaction energies for CCSD(T)/CBS, MP2, SCS-MP2, SCS(MI)-MP2, SCSN-MP2, SCS-MP2-vdW, RI-MP2, RI-SCS-MP2, RIJK-MP2, RIJK-SCS-MP2, RIJCOSX-MP2, RIJCOSX-SCS-MP2, SCS-MP2^{BWI-DZ}, RI-SCS-MP2^{BWI-DZ}, RIJK-SCS-MP2^{BWI-DZ}, RIJCOSX-SCS-MP2^{BWI-DZ} and SCS-MP2-hal^{G-XZ}. Data: 82 interaction energies. MP2: $C_{OS} = C_{SS}$ =1.00; SCS-MP2: C_{OS} =1.20, C_{SS} =0.33; SCS(MI)-MP2: C_{OS} =0.40, C_{SS} =1.29; SCSN-MP2: C_{OS} =0.00, C_{SS} =1.76; SCS-MP2-vdW: C_{OS} =1.28, C_{SS} =0.50; SCS-MP2^{BWI-DZ} RI-SCS-MP2^{BWI-DZ} , RIJK-SCS-MP2^{BWI-DZ} : C_{OS} =0.00, C_{SS} =1.50; RIJCOSX-SCS-MP2^{BWI-DZ} : C_{OS} =0.00, C_{SS} =0.17. All values in kcal/mol.

Dimer	Formula	CCSD(T)/CBS	MP2	SCS-MP2	SCS(MI)-MP2	SCSN-MP2	SCS-MP2-vdW	RI-MP2	RI-SCS-MP2	RIJK-MP2	RIJK-SCS-MP2	RIJCOSX-MP2	RIJCOSX-SCS-MP2	SCS-MP2^{BWI-DZ}	RI-SCS-MP2^{BWI-DZ}	RIJK-SCS-MP2^{BWI-DZ}	RIJCOSX-SCS-MP2^{BWI-DZ}	SCS-MP2-hal^{G-XZ}
8oxoG	oxo	-33.30	-31.12	-28.40	-30.46	-31.44	-29.56	-31.10	-28.40	-31.10	-28.39	-31.04	-28.34	-30.11	-30.09	-30.08	-30.08	-28.67
AA0324AAs	A	-6.25	-4.40	-1.72	-4.87	-6.40	-2.57	-4.42	-1.74	-4.44	-1.75	-4.37	-1.69	-5.26	-5.29	-5.30	-5.29	-2.99
AA0324ATis	T	-1.71	6.09	5.96	2.21	0.32	6.99	6.08	5.95	6.08	5.95	6.08	5.96	-0.33	-0.34	-0.34	-0.30	2.53
AA0324TAis	A	-1.30	6.57	6.41	2.69	0.80	7.46	6.56	6.41	6.56	6.41	6.59	6.44	0.14	0.13	0.13	0.17	2.98
AA0324TTs	T	-3.86	-2.56	-0.56	-2.75	-3.82	-1.23	-2.58	-0.56	-2.59	-0.58	-2.57	-0.57	-2.94	-2.96	-2.98	-3.00	-1.36
AA20305AAs2005	A	-6.06	-4.68	-1.81	-4.89	-6.39	-2.80	-4.70	-1.82	-4.73	-1.84	-4.67	-1.79	-5.12	-5.15	-5.17	-5.17	-2.90
AA20305ATis2005	T	-2.34	5.52	5.55	1.83	-0.05	6.48	5.51	5.55	5.51	5.55	5.55	5.58	-0.60	-0.61	-0.61	-0.57	2.24
AA20305TAis2005	A	-2.16	5.20	5.33	1.56	-0.35	6.21	5.19	5.32	5.19	5.32	5.18	5.32	-0.84	-0.85	-0.85	-0.85	2.02
AA20305TTs2005	T	-4.18	-2.89	-1.03	-3.16	-4.20	-1.64	-2.90	-1.04	-2.92	-1.06	-2.91	-1.05	-3.40	-3.43	-3.44	-3.48	-1.86
AA	mA	-0.70	-0.15	0.29	1.07	1.48	-0.18	-0.15	0.29	-0.15	0.29	-0.13	0.30	1.86	1.86	1.86	1.86	1.24
AAS	A	-8.58	-18.75	-15.70	-12.53	-10.86	-18.39	-18.76	-15.70	-18.78	-15.72	-18.72	-15.66	-8.54	-8.55	-8.57	-8.61	-11.09
Acst	A	-10.22	-19.02	-16.28	-13.57	-12.13	-18.66	-19.03	-16.28	-19.04	-16.29	-18.99	-16.23	-10.06	-10.07	-10.08	-10.12	-12.25
AG08319ACis	A	-0.18	7.50	7.66	4.13	2.35	8.46	7.49	7.65	7.49	7.65	7.51	7.67	1.91	1.90	1.90	1.93	4.59
AG08319AGs	A	-7.58	-4.39	-2.20	-5.65	-7.35	-2.68	-4.41	-2.22	-4.43	-2.23	-4.40	-2.21	-6.55	-6.57	-6.59	-6.59	-4.02
AG08319TCs	T	-6.07	-3.39	-1.83	-4.20	-5.36	-2.19	-3.41	-1.84	-3.42	-1.85	-3.41	-1.84	-4.78	-4.80	-4.81	-4.82	-3.04
AG08319TGis	G	-0.47	7.39	7.63	3.88	1.99	8.44	7.38	7.62	7.38	7.63	7.38	7.63	1.57	1.55	1.55	1.57	4.41
AT10326AAis	A	-0.92	7.62	7.77	3.55	1.42	8.75	7.60	7.76	7.60	7.75	7.63	7.79	0.87	0.86	0.86	0.90	4.08
AT10326ATs	A	-6.64	-4.91	-2.78	-5.49	-6.81	-3.41	-4.93	-2.79	-4.95	-2.81	-4.95	-2.81	-5.93	-5.95	-5.97	-6.00	-3.97
AT10326TTis	T	0.88	7.86	7.59	4.46	2.87	8.56	7.86	7.59	7.86	7.59	7.84	7.57	2.23	2.22	2.22	2.23	4.63
ATS1	A	-12.30	-21.33	-17.31	-16.11	-15.41	-20.10	-21.35	-17.32	-21.37	-17.33	-21.32	-17.28	-12.79	-12.82	-12.83	-12.90	-13.89
ATS2	mA	-8.10	-13.41	-10.61	-8.93	-8.02	-12.77	-13.42	-10.61	-13.44	-10.63	-13.40	-10.59	-6.07	-6.09	-6.11	-6.15	-7.48
ATWC	A	-16.86	-17.77	-15.82	-16.21	-16.36	-16.93	-17.77	-15.82	-17.76	-15.82	-17.70	-15.75	-15.24	-15.23	-15.22	-15.21	-15.04
Aust	A	-9.79	-18.46	-15.68	-12.95	-11.51	-18.10	-18.47	-15.69	-18.49	-15.70	-18.43	-15.65	-9.41	-9.43	-9.44	-9.48	-11.62
CC10	C	-8.27	-15.21	-12.90	-10.28	-8.90	-15.00	-15.22	-12.90	-15.23	-12.92	-15.23	-12.92	-7.10	-7.11	-7.12	-7.20	-9.21
CC11	C	-9.43	-16.27	-14.04	-11.59	-10.30	-16.05	-16.28	-14.04	-16.29	-14.05	-16.27	-14.04	-8.58	-8.59	-8.60	-8.66	-10.55
CC12	C	-7.43	-11.90	-10.73	-8.57	-7.46	-12.00	-11.90	-10.73	-11.91	-10.74	-11.91	-10.74	-6.42	-6.42	-6.43	-6.47	-8.12
CC13	C	-8.80	-15.01	-13.09	-10.55	-9.22	-14.93	-15.02	-13.09	-15.03	-13.10	-15.02	-13.09	-7.68	-7.68	-7.69	-7.75	-9.70
CC14	C	-9.11	-16.10	-13.65	-11.28	-10.02	-15.77	-16.11	-13.65	-16.12	-13.66	-16.12	-13.67	-8.18	-8.19	-8.20	-8.28	-10.10
CC1	C	2.45	-5.04	-2.76	0.92	2.83	-5.11	-5.05	-2.77	-5.05	-2.77	-5.07	-2.79	4.76	4.74	4.74	4.64	1.85
CC2	C	-3.85	-11.58	-9.11	-6.07	-4.48	-11.41	-11.59	-9.11	-11.59	-9.12	-11.60	-9.13	-2.52	-2.54	-2.54	-2.63	-4.95
CC3	C	-8.88	-16.22	-13.76	-11.11	-9.71	-15.96	-16.23	-13.76	-16.23	-13.76	-16.23	-13.76	-7.82	-7.83	-7.84	-7.91	-9.95
CC4	C	-9.92	-17.29	-14.86	-12.35	-11.03	-17.00	-17.29	-14.86	-17.30	-14.86	-17.29	-14.86	-9.18	-9.19	-9.19	-9.26	-11.20
CC5	C	0.32	-7.36	-4.98	-1.50	0.31	-7.34	-7.37	-4.98	-7.38	-4.99	-7.38	-4.99	2.28	2.26	2.26	2.17	-0.48
CC6	C	0.64	-7.26	-4.83	-1.30	0.54	-7.23	-7.27	-4.83	-7.28	-4.84	-7.28	-4.84	2.54	2.53	2.52	2.43	-0.26
CC7	C	-0.98	-6.60	-5.11	-2.20	-0.69	-6.77	-6.60	-5.11	-6.61	-5.12	-6.61	-5.12	0.65	0.64	0.64	0.58	-1.64
CC8	C	-9.10	-15.53	-13.56	-11.02	-9.69	-15.43	-15.54	-13.56	-15.55	-13.58	-15.55	-13.58	-8.11	-8.12	-8.13	-8.19	-10.14
CC9	C	-9.11	-16.50	-14.09	-11.63	-10.34	-16.20	-16.51	-14.09	-16.52	-14.10	-16.50	-14.09	-8.51	-8.52	-8.53	-8.59	-10.49
CG0319CCis	G	1.24	2.53	3.96	-0.13	-2.17	4.12	2.51	3.95	2.50	3.93	2.54	3.98	-1.93	-1.95	-1.97	-1.94	1.12
CG0319GCs	C	-7.88	-3.30	-1.61	-4.93	-6.56	-1.81	-3.32	-1.62	-3.33	-1.63	-3.32	-1.62	-6.04	-6.06	-6.07	-6.08	-3.59
CG0319GGis	C	-3.91	7.96	8.13	5.38	3.99	8.73	7.95	8.12	7.95	8.12	7.95	8.12	3.68	3.67	3.67	3.68	5.77
Cust	C	-10.42	-17.11	-14.80	-12.61	-11.45	-16.79	-17.12	-14.80	-17.13	-14.81	-17.10	-14.79	-9.73	-9.74	-9.75	-9.79	-11.50
F30K46	mPhe	-3.10	3.93	4.02	4.11	4.16	3.94	3.92	4.02	3.92	4.02	3.96	4.05	4.23	4.23	4.23	4.26	4.16
F30L33	mPhe	-5.00	-0.12	1.42	1.31	1.28	0.50	-0.13	1.42	-0.12	1.42	-0.09	1.45	2.20	2.19	2.19	2.18	2.21
GA10315ACis	A	-0.31	7.37	7.55	4.02	2.24	8.34	7.36	7.55	7.36	7.55	7.35	7.54	1.82	1.81	1.81	1.82	4.50
GA10315AGs	A	-9.14	-7.94	-4.80	-7.98	-9.52	-5.93	-7.97	-4.81	-7.99	-4.83	-7.95	-4.79	-8.10	-8.13	-8.15	-8.18	-5.82
GA10315TCs	T	-4.69	-2.38	-0.65	-2.87	-3.96	-1.15	-2.40	-0.65	-2.41	-0.67	-2.40	-0.66	-3.25	-3.27	-3.28	-3.31	-1.63
GA10315TGis	T	0.58	9.00	8.86	5.09	3.17	9.91	8.99	8.86	8.99	8.86	9.00	8.87	2.52	2.51	2.51	2.53	5.40
Gast	G	-11.38	-21.97	-18.76	-15.58	-13.91	-21.55	-21.98	-18.76	-22.00	-18.78	-21.94	-18.73	-11.48	-11.49	-11.51	-11.57	-14.05
GC0325CCis	C	3.09	9.91	10.00	7.36	6.03	10.62	9.90	10.00	9.90	10.00	9.92	10.01	5.69	5.68	5.68	5.70	7.69
GC0325GCs	G	-10.80	-9.06	-6.29	-9.17	-10.56	-7.27	-9.08	-6.30	-9.10	-6.32	-9.06	-6.29	-9.32	-9.35	-9.36	-9.39	-7.26
GC0325GGis	G	1.93	10.54	10.94	6.86	4.80	11.73	10.52	10.93	10.52	10.92	10.55	10.96	4.42	4.41	4.40	4.43	7.51
GCS	G	-19.02	-26.47	-22.64	-21.67	-21.10	-25.25	-26.49	-22.65	-26.51	-22.67	-26.48	-22.63	-18.64	-18.66	-18.68	-18.74	-19.55
Gcst	G	-10.60	-18.77	-16.25	-13.34	-11.82	-18.55	-18.78	-16.25	-18.79	-16.26	-18.78	-16.25	-9.85	-9.86	-9.87	-9.95	-12.18
GCWC1	C	-32.06	-31.43	-28.93	-30.23	-30.84	-30.14	-31.42	-28.92	-31.42	-28.93	-31.36	-28.87	-29.52	-29.50	-29.50	-29.50	-28.64
GCWC2	G	-31.40	-24.60	-22.26	-23.93	-24.72	-23.28	-24.58	-22.25	-24.58	-22.25	-24.51	-22.17	-23.55	-23.53	-23.53	-23.51	-22.39
GG0336CCs036	G	-1.62	-0.63	1.88	-1.42	-3.02	1.16	-0.66	1.87	-0.68	1.84	-0.65	1.88	-2.00	-2.03	-2.05	-2.08	0.39
GG0336CGis036	G	-3.68	4.50	4.71	0.84	-1.12	5.57	4.49	4.71	4.49	4.70	4.50	4.72	-1.58	-1.59	-1.59	-1.57	1.37
GG0336GCis036	C	-4.82	3.79	3.79	-0.23	-2.27	4.82	3.78	3.79	3.78	3.79	3.78	3.79	-2.88	-2.89	-2.89	-2.87	0.19
GG0336GGs036	C	-3.54	1.07	2.84	0.88	-0.07	2.24	1.05	2.83	1.04	2.82	1.04	2.82	0.71	0.69	0.68	0.65	2.11
Ggst	G	-12.67	-23.23	-19.96	-16.61	-14.84	-22.84	-23.24	-19.96	-23.26	-19.98	-23.23	-19.95	-12.35	-12.36	-12.37	-12.45	-15.05
GT10315ACs	A	-5.44	-3.57	-1.17	-3.77	-5.04	-1.99	-3.59	-1.18	-3.60	-1.20	-3.59	-1.19	-3.98	-4.01	-4.02	-4.06	-2.11
GT10315AGis	A	-4.06	4.42	4.82	0.54	-1.61	5.66	4.41	4.81	4.40	4.81	4.42	4.83	-2.02	-2.03	-2.03	-2.01	1.22
GT10315TCis	T	0.30	7.36	7.24	4.21	2.68	8.08	7.36	7.23	7.36	7.23	7.34	7.22	2.14	2.13	2.13	2.14	4.45
GT10315TGs	T	-4.96	-3.48	-0.97	-3.55	-4.79	-1.87	-3.51	-0.98	-3.52	-1.00	-3.51	-0.98	-3.66	-3.70	-3.71	-3.75	-1.82
Gust	G	-12.09	-20.64	-17.82	-15.13	-13.71	-20.25	-20.65	-17.83	-20.66	-17.84	-20.62	-17.80	-11.59	-11.60	-11.61	-11.67	-13.77
Guwobble	G	-19.10	-18.99	-16.88	-17.78	-18.19	-17.95	-18.98	-16.88	-18.98	-16.88	-18.92	-16.82	-17.05	-17.04	-17.04	-17.04	-16.46
ICWC	C	-24.90	-24.58	-22.31	-23.74	-24.41	-23.35	-24.57	-22.30	-24.57	-22.30	-24.53	-22.26	-23.25	-23.24	-23.23	-23.24	-22.27
mAmTH	mA	-18.16	-19.70	-17.47	-18.04	-18.27	-18.71	-19.69	-17.47	-19.69	-17.46	-19.65	-17.43	-17.01	-17.00	-16.99	-17.00	-16.69
mAmTS	mA	-14.57	-26.48	-20.93	-19.46	-18.59	-24.73	-26.50	-20.94	-26.52	-20.96	-26.48	-20.92	-15.02	-15.05	-15.06	-15.16	-16.39
mAmTWCAT	mA	-16.40	-16.00	-14.21	-14.25	-14.23	-15.31	-15.99	-14.21	-15.98	-14.20	-15.96	-14.19	-13.16	-13.14	-13.14	-13.15	-13.21
mGmCS	mG	-20.35	-28.98	-23.80	-22.70	-22.03	-27.28	-29.00	-23.81	-29.02	-23.83	-28.98	-23.80	-18.73	-18.76	-18.78	-18.88	-19.81
TA08316AAis	A	-1.55	4.94	5.96	2.17	0.28	6.29	4.92	5.95	4.92	5.94	4.93	5.97	0.32	0.31	0.30	0.31	3.16
TA08316ATs	A	-6.07	-5.33	-1.95	-4.24	-5.32	-3.46	-5.35	-1.97	-5.37	-1.98	-5.34	-1.96	-3.63	-3.67	-3.68	-3.73	-2.04
TA08316TTis	T	0.70	7.83	7.49	4.34	2.74	8.51	7.83	7.49	7.83	7.50	7.83	7.48	2.06	2.05	2.05	2.07	4.47
TG0319ACs	A	-4.96	-1.69	0.31	-2.52	-3.90	-0.21	-1.71	0.29	-1.72	0.29	-1.70	0.30	-3.12	-3.14	-3.15	-3.17	-1.06
TG0319AGis	A	-4.22	2.66	3.71	-0.43	-2.50	4.11	2.65	3.70	2.64	3.69	2.67	3.72	-2.49	-2.51	-2.51	-2.49	0.61
TG0319TCis	T	-1.15	5.93	5.81	2.70	1.12	6.68	5.93	5.81	5.93	5.81	5.93	5.81	0.57	0.56	0.56	0.58	2.95
TG0319TGs	T	-5.67	-1.24	0.24	-3.05	-4.67	0.16	-1.25	0.23	-1.26	0.22	-1.25	0.23	-4.28	-4.30	-4.31	-4.31	-1.85
TT	mT	1.00	3.94	4.16	4.44	4.58	3.95	3.94	4.16	3.94	4.17	3.92	4.15	4.76	4.75	4.76	4.74	4.54
Uucalcutta	U	-10.30	-11.60	-10.18	-10.87	-11.19	-10.88	-11.60	-10.18	-11.60	-10.18	-11.59	-10.16	-10.43	-10.43	-10.43	-10.43	-9.97
Uupl	U	-13.70	-14.57	-12.82	-13.73	-14.15	-13.67	-14.57	-12.82	-14.57	-12.82	-14.56	-12.82	-13.23	-13.22	-13.22	-13.23	-12.62
Uust	U	-7.46	-14.11	-11.96	-9.69	-8.50	-13.87	-14.12	-11.96	-14.13	-11.97	-14.11	-11.95	-6.85	-6.86	-6.87	-6.90	-8.68

DFT

Table S46: Interaction energies for CCSD(T)/CBS, B97M-V, ωB97X-V, ωB97M-V, ωB97X-D3, B2PLYP-D3BJ, DSD-BLYP-D3BJ, ωB97X-D4 and B2PLYP-D4. Data: 82 interaction energies. All values in kcal/mol.

Dimer	Formula	CCSD(T)/CBS	B97M-V	ωB97X-V	ωB97M-V	ωB97X-D3	B2PLYP-D3BJ	DSD-BLYP-D3BJ	ωB97X-D4	B2PLYP-D4
8oxoG	oxo−G−C	-33.30	-28.76	-30.47	-30.54	-31.41	-31.36	-31.05	-31.83	-28.76
AA0324AAs	A−A	-6.25	-3.20	-2.60	-3.83	-3.66	-1.58	-2.35	-3.24	-3.20
AA0324ATis	T−A	-1.71	1.76	2.22	1.71	1.12	4.12	4.08	1.29	1.76
AA0324TAis	A−T	-1.30	2.08	2.63	2.12	1.51	4.52	4.48	1.68	2.08
AA0324TTs	T−T	-3.86	-1.31	-1.06	-1.89	-1.95	-0.36	-1.03	-1.43	-1.31
AA20305AAs2005	A−A	-6.06	-2.96	-2.40	-3.65	-3.45	-1.46	-2.29	-3.05	-2.96
AA20305ATis2005	T−A	-2.34	1.09	1.69	1.14	0.57	3.52	3.49	0.53	1.09
AA20305TAis2005	A−T	-2.16	1.19	1.61	1.08	0.58	3.40	3.32	0.40	1.19
AA20305TTs2005	T−T	-4.18	-1.68	-1.59	-2.32	-2.47	-0.77	-1.40	-1.86	-1.68
AA	mA−mA	-0.70	1.49	0.24	0.38	0.10	0.36	0.39	0.05	1.49
AAS	A−A	-8.58	-8.44	-9.63	-9.79	-8.91	-11.28	-12.34	-8.49	-8.44
Acst	A−C	-10.22	-9.91	-11.38	-11.53	-10.86	-12.75	-13.60	-10.39	-9.91
AG08319ACis	A−C	-0.18	3.40	4.30	3.76	3.09	5.71	5.71	3.23	3.40
AG08319AGs	A−G	-7.58	-4.35	-3.68	-4.60	-4.59	-2.44	-3.08	-4.29	-4.35
AG08319TCs	T−C	-6.07	-3.22	-2.84	-3.37	-3.66	-2.08	-2.52	-3.34	-3.22
AG08319TGis	G−T	-0.47	3.26	4.00	3.42	2.65	5.73	5.62	2.85	3.26
AT10326AAis	A−A	-0.92	2.52	3.26	2.62	1.86	5.25	5.32	1.91	2.52
AT10326ATs	A−T	-6.64	-3.90	-3.73	-4.56	-4.69	-2.69	-3.33	-4.24	-3.90
AT10326TTis	T−T	0.88	4.38	4.75	4.31	3.86	6.42	6.26	3.98	4.38
ATS1	A−T	-12.30	-13.00	-12.48	-13.42	-12.21	-14.00	-15.19	-11.51	-13.00
ATS2	mA−mT	-8.10	-5.95	-7.46	-7.68	-7.31	-7.95	-8.72	-6.68	-5.95
ATWC	A−T	-16.86	-15.11	-15.93	-15.97	-16.48	-17.01	-16.92	-16.76	-15.11
Aust	A−U	-9.79	-9.78	-10.97	-11.11	-10.42	-12.41	-13.20	-9.90	-9.78
CC10	C−C	-8.27	-7.87	-9.30	-9.32	-9.09	-10.44	-10.94	-8.54	-7.87
CC11	C−C	-9.43	-8.95	-10.56	-10.55	-10.13	-11.62	-12.15	-9.73	-8.95
CC12	C−C	-7.43	-7.02	-8.44	-8.02	-7.52	-8.95	-9.22	-7.67	-7.02
CC13	C−C	-8.80	-8.37	-9.78	-9.69	-9.64	-10.64	-11.12	-9.16	-8.37
CC14	C−C	-9.11	-8.76	-10.27	-10.30	-9.91	-11.32	-11.85	-9.52	-8.76
CC1	C−C	2.45	2.50	1.63	1.59	1.96	0.03	-0.44	2.46	2.50
CC2	C−C	-3.85	-3.87	-4.90	-5.06	-4.53	-6.33	-6.91	-4.01	-3.87
CC3	C−C	-8.88	-8.73	-10.02	-10.08	-9.61	-11.18	-11.75	-9.25	-8.73
CC4	C−C	-9.92	-9.56	-11.10	-11.11	-10.68	-12.17	-12.77	-10.29	-9.56
CC5	C−C	0.32	0.36	-0.52	-0.62	-0.09	-2.00	-2.58	0.32	0.36
CC6	C−C	0.64	0.62	-0.24	-0.36	0.16	-1.75	-2.36	0.61	0.62
CC7	C−C	-0.98	-0.88	-1.80	-1.66	-1.40	-2.90	-3.22	-1.14	-0.88
CC8	C−C	-9.10	-8.81	-10.29	-10.06	-9.60	-11.03	-11.55	-9.54	-8.81
CC9	C−C	-9.11	-8.72	-10.35	-10.47	-10.12	-11.52	-12.10	-9.53	-8.72
CG0319CCis	G−G	1.24	-0.15	0.70	-0.19	-1.16	2.34	1.98	-0.42	-0.15
CG0319GCs	C−G	-7.88	-4.28	-3.38	-4.13	-4.05	-2.37	-2.85	-4.18	-4.28
CG0319GGis	C−C	-3.91	4.93	6.07	5.62	5.07	6.82	6.74	5.28	4.93
Cust	C−U	-10.42	-10.44	-11.65	-11.64	-11.13	-12.66	-13.15	-10.78	-10.44
F30K46	mPhe−mLys	-3.10	4.23	3.38	3.36	2.96	4.07	4.06	3.08	4.23
F30L33	mPhe−mLeu	-5.00	0.85	0.62	0.09	-0.25	1.13	1.02	-0.26	0.85
GA10315ACis	A−C	-0.31	3.41	4.23	3.74	3.26	5.72	5.69	3.26	3.41
GA10315AGs	A−G	-9.14	-6.18	-5.35	-6.58	-6.44	-4.41	-5.32	-5.80	-6.18
GA10315TCs	T−C	-4.69	-1.76	-1.33	-2.02	-2.46	-0.66	-1.19	-1.81	-1.76
GA10315TGis	T−G	0.58	4.49	5.30	4.76	4.27	7.13	7.00	4.32	4.49
Gast	G−A	-11.38	-11.20	-12.56	-12.71	-11.67	-14.27	-15.37	-11.19	-11.20
GC0325CCis	C−C	3.09	6.71	7.99	7.49	7.15	8.75	8.67	7.28	6.71
GC0325GCs	G−C	-10.80	-7.68	-6.89	-7.93	-7.95	-6.22	-6.99	-7.37	-7.68
GC0325GGis	G−G	1.93	6.22	7.37	6.73	5.94	8.95	8.81	6.21	6.22
GCS	G−C	-19.02	-19.54	-18.39	-19.39	-17.99	-19.72	-20.91	-17.71	-19.54
Gcst	G−C	-10.60	-10.51	-12.02	-11.99	-11.55	-13.33	-13.96	-10.88	-10.51
GCWC1	C−G	-32.06	-29.28	-29.83	-30.00	-30.68	-30.99	-30.85	-31.10	-29.28
GCWC2	G−C	-31.40	-23.38	-24.48	-24.77	-25.47	-25.02	-24.79	-25.85	-23.38
GG0336CCs036	G−G	-1.62	0.01	0.98	0.00	-0.09	1.94	1.16	0.43	0.01
GG0336CGis036	G−C	-3.68	0.14	1.09	0.62	-0.13	2.64	2.61	0.06	0.14
GG0336GCis036	C−G	-4.82	-0.92	0.04	-0.45	-1.12	1.68	1.66	-0.99	-0.92
GG0336GGs036	C−C	-3.54	1.59	2.56	1.92	1.47	2.75	2.30	1.93	1.59
Ggst	G−G	-12.67	-12.59	-14.03	-14.11	-13.02	-15.79	-16.78	-12.41	-12.59
GT10315ACs	A−C	-5.44	-2.29	-1.64	-2.62	-2.74	-1.01	-1.69	-2.20	-2.29
GT10315AGis	A−G	-4.06	-0.48	0.48	-0.18	-0.89	2.42	2.40	-0.87	-0.48
GT10315TCis	T−C	0.30	3.89	4.63	4.17	3.76	5.95	5.84	3.85	3.89
GT10315TGs	T−G	-4.96	-2.06	-1.44	-2.39	-2.44	-0.60	-1.41	-1.90	-2.06
Gust	G−U	-12.09	-12.21	-13.45	-13.50	-12.64	-14.87	-15.58	-12.08	-12.21
Guwobble	G−U	-19.10	-16.44	-17.38	-17.56	-17.67	-18.15	-18.12	-18.06	-16.44
ICWC	C−oA	-24.90	-21.89	-23.19	-23.20	-23.89	-24.04	-23.95	-24.14	-21.89
mAmTH	mA−mT	-18.16	-16.22	-17.42	-17.49	-17.94	-18.70	-18.65	-18.32	-16.22
mAmTS	mA−mT	-14.57	-15.36	-14.91	-16.35	-14.35	-17.10	-18.63	-13.67	-15.36
mAmTWCAT	mA−mT	-16.40	-13.41	-14.88	-14.80	-15.31	-15.55	-15.38	-15.63	-13.41
mGmCS	mG−mC	-20.35	-19.23	-18.58	-20.02	-18.03	-20.25	-21.77	-17.41	-19.23
TA08316AAis	A−A	-1.55	2.01	2.79	1.90	1.50	4.25	4.05	1.43	2.01
TA08316ATs	A−T	-6.07	-2.05	-1.29	-2.76	-2.03	-0.91	-1.99	-1.44	-2.05
TA08316TTis	T−T	0.70	4.26	4.62	4.17	3.77	6.36	6.19	3.82	4.26
TG0319ACs	A−C	-4.96	-1.81	-1.00	-1.91	-1.96	-0.15	-0.66	-1.81	-1.81
TG0319AGis	A−G	-4.22	-0.66	0.06	-0.75	-1.51	1.79	1.60	-1.12	-0.66
TG0319TCis	T−C	-1.15	2.45	3.12	2.68	2.19	4.45	4.35	2.33	2.45
TG0319TGs	T−G	-5.67	-2.26	-1.64	-2.39	-2.38	-0.34	-0.87	-2.29	-2.26
TT	mT−mT	1.00	4.50	3.89	3.96	3.91	4.31	4.27	3.96	4.50
Uucalcutta	U−U	-10.30	-10.13	-10.30	-10.42	-10.52	-11.01	-11.00	-11.04	-10.13
Uupl	U−U	-13.70	-12.85	-13.35	-13.58	-13.62	-14.01	-13.97	-14.09	-12.85
Uust	U−U	-7.46	-7.65	-8.68	-8.73	-8.33	-9.89	-10.32	-7.75	-7.65

SAPT-DFT

Table S47: Interaction energies for CCSD(T)/CBS and SAPT-DFT. For SAPT-DFT method we used the B3LYP, PBE0 and ωB97X DFA's in conjunction with the aug-cc-pVDZ basis set. Data: 82 interaction energies. All values in kcal/mol.

Dimer	Formula	CCSD(T)/CBS	SAPT-B3LYP	SAPT-PBE0	SAPT-ωB97X
8oxoG	oxo−G−C	-33.30	-25.04	-26.56	-26.21
AA0324AAs	A−A	-6.25	-3.22	-3.10	-2.04
AA0324ATis	T−A	-1.71	-1.56	-1.53	-1.40
AA0324TAis	A−T	-1.30	-1.20	-1.19	-1.04
AA0324TTs	T−T	-3.86	-1.12	-1.11	-0.17
AA20305AAs2005	A−A	-6.06	-2.82	-2.73	-1.59
AA20305ATis2005	T−A	-2.34	-1.71	-1.74	-1.48
AA20305TAis2005	A−T	-2.16	-1.62	-1.65	-1.43
AA20305TTs2005	T−T	-4.18	-1.75	-1.75	-0.82
AA	mA−mA	-0.70	0.10	0.11	0.36
AAS	A−A	-8.58	-5.56	-5.52	-4.52
Acst	A−C	-10.22	-7.24	-7.26	-6.60
AG08319ACis	A−C	-0.18	0.22	0.20	0.39
AG08319AGs	A−G	-7.58	-5.37	-5.08	-4.27
AG08319TCs	T−C	-6.07	-4.38	-4.17	-3.60
AG08319TGis	G−T	-0.47	0.41	0.33	0.70
AT10326AAis	A−A	-0.92	-0.36	-0.37	-0.10
AT10326ATs	A−T	-6.64	-4.16	-4.12	-3.19
AT10326TTis	T−T	0.88	1.00	0.98	1.11
ATS1	A−T	-12.30	-7.70	-7.83	-6.53
ATS2	mA−mT	-8.10	-5.08	-5.03	-4.11
ATWC	A−T	-16.86	-11.65	-12.57	-11.86
Aust	A−U	-9.79	-6.93	-6.97	-6.11
CC10	C−C	-8.27	-5.75	-5.61	-4.96
CC11	C−C	-9.43	-6.87	-6.75	-6.33
CC12	C−C	-7.43	-6.30	-5.83	-5.74
CC13	C−C	-8.80	-6.37	-6.27	-5.81
CC14	C−C	-9.11	-6.29	-6.15	-5.63
CC1	C−C	2.45	4.87	4.91	5.92
CC2	C−C	-3.85	-1.07	-1.03	-0.26
CC3	C−C	-8.88	-6.02	-5.91	-5.33
CC4	C−C	-9.92	-7.16	-7.00	-6.54
CC5	C−C	0.32	3.02	3.09	3.97
CC6	C−C	0.64	3.47	3.46	4.36
CC7	C−C	-0.98	0.59	0.71	1.31
CC8	C−C	-9.10	-6.90	-6.53	-6.16
CC9	C−C	-9.11	-6.30	-6.30	-5.83
CG0319CCis	G−G	1.24	-1.68	-2.02	-1.38
CG0319GCs	C−G	-7.88	-6.08	-5.57	-5.23
CG0319GGis	C−C	-3.91	1.41	1.38	1.56
Cust	C−U	-10.42	-7.87	-7.79	-7.29
F30L33	mPhe−mLeu	-5.00	-3.39	-3.32	-2.08
GA10315ACis	A−C	-0.31	0.07	0.14	0.30
GA10315AGs	A−G	-9.14	-5.68	-5.65	-4.38
GA10315TCs	T−C	-4.69	-2.55	-2.55	-1.88
GA10315TGis	T−G	0.58	0.91	0.92	1.07
Gast	G−A	-11.38	-8.17	-8.11	-7.26
GC0325CCis	C−C	3.09	3.12	3.15	3.33
GC0325GCs	G−C	-10.80	-7.67	-7.59	-6.79
GC0325GGis	G−G	1.93	2.81	2.78	3.16
GCS	G−C	-19.02	-14.29	-14.48	-13.62
Gcst	G−C	-10.60	-8.00	-8.07	-7.32
GCWC1	C−G	-32.06	-24.21	-25.50	-25.23
GCWC2	G−C	-31.40	-24.39	-25.95	-25.48
GG0336CCs036	G−G	-1.62	-0.74	-0.55	0.43
GG0336CGis036	G−C	-3.68	-3.42	-3.39	-3.22
GG0336GCis036	C−G	-4.82	-4.78	-4.76	-4.73
GG0336GGs036	C−C	-3.54	0.21	0.35	0.99
Ggst	G−G	-12.67	-9.31	-9.27	-8.40
GT10315ACs	A−C	-5.44	-2.68	-2.59	-1.81
GT10315AGis	A−G	-4.06	-3.33	-3.32	-2.93
GT10315TCis	T−C	0.30	0.33	0.32	0.40
GT10315TGs	T−G	-4.96	-1.69	-1.70	-0.65
Gust	G−U	-12.09	-9.16	-9.18	-8.41
Guwobble	G−U	-19.10	-13.26	-14.31	-13.97
ICWC	C−oA	-24.90	-18.84	-20.08	-19.71
mAmTH	mA−mT	-18.16	-12.83	-13.80	-13.10
mAmTS	mA−mT	-14.57	-7.71	-7.99	-6.39
mAmTWCAT	mA−mT	-16.40	-11.70	-12.67	-12.05
mGmCS	mG−mC	-20.35	-13.64	-13.84	-12.09
TA08316AAis	A−A	-1.55	-0.04	0.01	0.56
TA08316ATs	A−T	-6.07	-0.33	-0.43	1.05
TA08316TTis	T−T	0.70	0.80	0.81	0.88
TG0319ACs	A−C	-4.96	-2.65	-2.33	-1.70
TG0319AGis	A−G	-4.22	-2.79	-2.88	-2.28
TG0319TCis	T−C	-1.15	-1.13	-1.12	-1.06
TG0319TGs	T−G	-5.67	-3.77	-3.48	-2.89
TT	mT−mT	1.00	1.09	1.07	1.23
Uucalcutta	U−U	-10.30	-7.30	-7.72	-7.50
Uupl	U−U	-13.70	-9.18	-9.89	-9.72
Uust	U−U	-7.46	-5.40	-5.41	-4.83

5.1.4 Absolute deviations of molecular systems.

MP2 with C_{OS} y C_{SS} values

Table S48: Absolute deviations for MP2, SCS-MP2, SCS(MI)-MP2, SCSN-MP2, SCS-MP2-vdW, RI-MP2, RI-SCS-MP2, RIJK-MP2, RIJK-SCS-MP2, RIJCOSX-MP2, RIJCOSX-SCS-MP2, SCS-MP2^{BWI-DZ}, RI-SCS-MP2^{BWI-DZ}, RIJK-SCS-MP2^{BWI-DZ}, RIJCOSX-SCS-MP2^{BWI-DZ} and SCS-MP2-hal^{G-XZ}. Data: 82 interaction energies. MP2: C_{OS} = C_{SS} =1.00; SCS-MP2: C_{OS} =1.20, C_{SS} =0.33; SCS(MI)-MP2: C_{OS} =0.40, C_{SS} =1.29; SCSN-MP2: C_{OS} =0.00, C_{SS} =1.76; SCS-MP2-vdW: C_{OS} =1.28, C_{SS} =0.50; SCS-MP2^{BWI-DZ} RI-SCS-MP2^{BWI-DZ} , RIJK-SCS-MP2^{BWI-DZ} : C_{OS} =0.00, C_{SS} =1.50; RIJCOSX-SCS-MP2^{BWI-DZ} : C_{OS} =0.00, C_{SS} =0.17. All values in kcal/mol.

Dimer	Formula	MP2	SCS-MP2	SCS(MI)-MP2	SCSN-MP2	SCS-MP2-vdW	RI-MP2	RI-SCS-MP2	RIJK-MP2	RIJK-SCS-MP2	RIJCOSX-MP2	RIJCOSX-SCS-MP2	SCS-MP2^{BWI-DZ}	RI-SCS-MP2^{BWI-DZ}	RIJK-SCS-MP2^{BWI-DZ}	RIJCOSX-SCS-MP2^{BWI-DZ}	SCS-MP2-hal^{G-XZ}
8oxoG	oxo	2.18	4.90	2.84	1.86	3.74	2.20	4.90	2.20	4.91	2.26	4.96	3.19	3.21	3.22	3.22	4.63
AA0324AAs	A	1.85	4.53	1.38	0.15	3.68	1.83	4.51	1.81	4.50	1.88	4.56	0.99	0.96	0.95	0.96	3.26
AA0324ATis	T	7.80	7.67	3.92	2.03	8.70	7.79	7.66	7.79	7.66	7.79	7.67	1.38	1.37	1.37	1.41	4.24
AA0324TAis	A	7.87	7.71	3.99	2.10	8.76	7.86	7.71	7.86	7.71	7.89	7.74	1.44	1.43	1.43	1.47	4.28
AA0324TTs	T	1.30	3.30	1.11	0.04	2.63	1.28	3.30	1.27	3.28	1.29	3.29	0.92	0.90	0.88	0.86	2.50
AA20305AAs2005	A	1.38	4.25	1.17	0.33	3.26	1.36	4.24	1.33	4.22	1.39	4.27	0.94	0.91	0.89	0.89	3.16
AA20305ATis2005	T	7.86	7.89	4.17	2.29	8.82	7.85	7.89	7.85	7.89	7.89	7.92	1.74	1.73	1.73	1.77	4.58
AA20305TAis2005	A	7.36	7.49	3.72	1.81	8.37	7.35	7.48	7.35	7.48	7.34	7.48	1.32	1.31	1.31	1.31	4.18
AA20305TTs2005	T	1.29	3.15	1.02	0.02	2.54	1.28	3.14	1.26	3.12	1.27	3.13	0.78	0.75	0.74	0.70	2.32
AA	mA	0.55	0.99	1.77	2.18	0.52	0.55	0.99	0.55	0.99	0.57	1.00	2.56	2.56	2.56	2.56	1.94
AAS	A	10.17	7.12	3.95	2.28	9.81	10.18	7.12	10.20	7.14	10.14	7.08	0.04	0.03	0.01	0.03	2.51
Acst	A	8.80	6.06	3.35	1.91	8.44	8.81	6.06	8.82	6.07	8.77	6.01	0.16	0.15	0.14	0.10	2.03
AG08319ACis	A	7.68	7.84	4.31	2.53	8.64	7.67	7.83	7.67	7.83	7.69	7.85	2.09	2.08	2.08	2.11	4.77
AG08319AGs	A	3.19	5.38	1.93	0.23	4.90	3.17	5.36	3.15	5.35	3.18	5.37	1.03	1.01	0.99	0.99	3.56
AG08319TCs	T	2.68	4.24	1.87	0.71	3.88	2.66	4.23	2.65	4.22	2.66	4.23	1.29	1.27	1.26	1.25	3.03
AG08319TGis	G	7.86	8.10	4.35	2.46	8.91	7.85	8.09	7.85	8.10	7.85	8.10	2.04	2.02	2.02	2.04	4.88
AT10326AAis	A	8.54	8.69	4.47	2.34	9.67	8.52	8.68	8.52	8.67	8.55	8.71	1.79	1.78	1.78	1.82	5.00
AT10326ATs	A	1.73	3.86	1.15	0.17	3.23	1.71	3.85	1.69	3.83	1.69	3.83	0.71	0.69	0.67	0.64	2.67
AT10326TTis	T	6.98	6.71	3.58	1.99	7.68	6.98	6.71	6.98	6.71	6.96	6.69	1.35	1.34	1.34	1.35	3.75
ATS1	A	9.03	5.01	3.81	3.11	7.80	9.05	5.02	9.07	5.03	9.02	4.98	0.49	0.52	0.53	0.60	1.59
ATS2	mA	5.31	2.51	0.83	0.08	4.67	5.32	2.51	5.34	2.53	5.30	2.49	2.03	2.01	1.99	1.95	0.62
ATWC	A	0.91	1.04	0.65	0.50	0.07	0.91	1.04	0.90	1.04	0.84	1.11	1.62	1.63	1.64	1.65	1.82
Aust	A	8.67	5.89	3.16	1.72	8.31	8.68	5.90	8.70	5.91	8.64	5.86	0.38	0.36	0.35	0.31	1.83
CC10	C	6.94	4.63	2.01	0.63	6.73	6.95	4.63	6.96	4.65	6.96	4.65	1.17	1.16	1.15	1.07	0.94
CC11	C	6.84	4.61	2.16	0.87	6.62	6.85	4.61	6.86	4.62	6.84	4.61	0.85	0.84	0.83	0.77	1.12
CC12	C	4.47	3.30	1.14	0.03	4.57	4.47	3.30	4.48	3.31	4.48	3.31	1.01	1.01	1.00	0.96	0.69
CC13	C	6.21	4.29	1.75	0.42	6.13	6.22	4.29	6.23	4.30	6.22	4.29	1.12	1.12	1.11	1.05	0.90
CC14	C	6.99	4.54	2.17	0.91	6.66	7.00	4.54	7.01	4.55	7.01	4.56	0.93	0.92	0.91	0.83	0.99
CC1	C	7.49	5.21	1.53	0.38	7.56	7.50	5.22	7.50	5.22	7.52	5.24	2.31	2.29	2.29	2.19	0.60
CC2	C	7.73	5.26	2.22	0.63	7.56	7.74	5.26	7.74	5.27	7.75	5.28	1.33	1.31	1.31	1.22	1.10
CC3	C	7.34	4.88	2.23	0.83	7.08	7.35	4.88	7.35	4.88	7.35	4.88	1.06	1.05	1.04	0.97	1.07
CC4	C	7.37	4.94	2.43	1.11	7.08	7.37	4.94	7.38	4.94	7.37	4.94	0.74	0.73	0.73	0.66	1.28
CC5	C	7.68	5.30	1.82	0.01	7.66	7.69	5.30	7.70	5.31	7.70	5.31	1.96	1.94	1.94	1.85	0.80
CC6	C	7.90	5.47	1.94	0.10	7.87	7.91	5.47	7.92	5.48	7.92	5.48	1.90	1.89	1.88	1.79	0.90
CC7	C	5.62	4.13	1.22	0.29	5.79	5.62	4.13	5.63	4.14	5.63	4.14	1.63	1.62	1.62	1.56	0.66
CC8	C	6.43	4.46	1.92	0.59	6.33	6.44	4.46	6.45	4.48	6.45	4.48	0.99	0.98	0.97	0.91	1.04
CC9	C	7.39	4.98	2.52	1.23	7.09	7.40	4.98	7.41	4.99	7.39	4.98	0.60	0.59	0.58	0.52	1.38
CG0319CCis	G	1.29	2.72	1.37	3.41	2.88	1.27	2.71	1.26	2.69	1.30	2.74	3.17	3.19	3.21	3.18	0.12
CG0319GCs	C	4.58	6.27	2.95	1.32	6.07	4.56	6.26	4.55	6.25	4.56	6.26	1.84	1.82	1.81	1.80	4.29
CG0319GGis	C	11.87	12.04	9.29	7.90	12.64	11.86	12.03	11.86	12.03	11.86	12.03	7.59	7.58	7.58	7.59	9.68
Cust	C	6.69	4.38	2.19	1.03	6.37	6.70	4.38	6.71	4.39	6.68	4.37	0.69	0.68	0.67	0.63	1.08
F30K46	mPhe	7.03	7.12	7.21	7.26	7.04	7.02	7.12	7.02	7.12	7.06	7.15	7.33	7.33	7.33	7.36	7.26
F30L33	mPhe	4.88	6.42	6.31	6.28	5.50	4.87	6.42	4.88	6.42	4.91	6.45	7.20	7.19	7.19	7.18	7.21
GA10315ACis	A	7.68	7.86	4.33	2.55	8.65	7.67	7.86	7.67	7.86	7.66	7.85	2.13	2.12	2.12	2.13	4.81
GA10315AGs	A	1.20	4.34	1.16	0.38	3.21	1.17	4.33	1.15	4.31	1.19	4.35	1.04	1.01	0.99	0.96	3.32
GA10315TCs	T	2.31	4.04	1.82	0.73	3.54	2.29	4.04	2.28	4.02	2.29	4.03	1.44	1.42	1.41	1.38	3.06
GA10315TGis	T	8.42	8.28	4.51	2.59	9.33	8.41	8.28	8.41	8.28	8.42	8.29	1.94	1.93	1.93	1.95	4.82
Gast	G	10.59	7.38	4.20	2.53	10.17	10.60	7.38	10.62	7.40	10.56	7.35	0.10	0.11	0.13	0.19	2.67
GC0325CCis	C	6.82	6.91	4.27	2.94	7.53	6.81	6.91	6.81	6.91	6.83	6.92	2.60	2.59	2.59	2.61	4.60
GC0325GCs	G	1.74	4.51	1.63	0.24	3.53	1.72	4.50	1.70	4.48	1.74	4.51	1.48	1.45	1.44	1.41	3.54
GC0325GGis	G	8.61	9.01	4.93	2.87	9.80	8.59	9.00	8.59	8.99	8.62	9.03	2.49	2.48	2.47	2.50	5.58
GCS	G	7.45	3.62	2.65	2.08	6.23	7.47	3.63	7.49	3.65	7.46	3.61	0.38	0.36	0.34	0.28	0.53
Gcst	G	8.17	5.65	2.74	1.22	7.95	8.18	5.65	8.19	5.66	8.18	5.65	0.75	0.74	0.73	0.65	1.58
GCWC1	C	0.63	3.13	1.83	1.22	1.92	0.64	3.14	0.64	3.13	0.70	3.19	2.54	2.56	2.56	2.56	3.42
GCWC2	G	6.80	9.14	7.47	6.68	8.12	6.82	9.15	6.82	9.15	6.89	9.23	7.85	7.87	7.87	7.89	9.01
GG0336CCs036	G	0.99	3.50	0.20	1.40	2.78	0.96	3.49	0.94	3.46	0.97	3.50	0.38	0.41	0.43	0.46	2.01
GG0336CGis036	G	8.18	8.39	4.52	2.56	9.25	8.17	8.39	8.17	8.38	8.18	8.40	2.10	2.09	2.09	2.11	5.05
GG0336GCis036	C	8.61	8.61	4.59	2.55	9.64	8.60	8.61	8.60	8.61	8.60	8.61	1.94	1.93	1.93	1.95	5.01
GG0336GGs036	C	4.61	6.38	4.42	3.47	5.78	4.59	6.37	4.58	6.36	4.58	6.36	4.25	4.23	4.22	4.19	5.65
Ggst	G	10.56	7.29	3.94	2.17	10.17	10.57	7.29	10.59	7.31	10.56	7.28	0.32	0.31	0.30	0.22	2.38
GT10315ACs	A	1.87	4.27	1.67	0.40	3.45	1.85	4.26	1.84	4.24	1.85	4.25	1.46	1.43	1.42	1.38	3.33
GT10315AGis	A	8.48	8.88	4.60	2.45	9.72	8.47	8.87	8.46	8.87	8.48	8.89	2.04	2.03	2.03	2.05	5.28
GT10315TCis	T	7.06	6.94	3.91	2.38	7.78	7.06	6.93	7.06	6.93	7.04	6.92	1.84	1.83	1.83	1.84	4.15
GT10315TGs	T	1.48	3.99	1.41	0.17	3.09	1.45	3.98	1.44	3.96	1.45	3.98	1.30	1.26	1.25	1.21	3.14
Gust	G	8.55	5.73	3.04	1.62	8.16	8.56	5.74	8.57	5.75	8.53	5.71	0.50	0.49	0.48	0.42	1.68
Guwobble	G	0.11	2.22	1.32	0.91	1.15	0.12	2.22	0.12	2.22	0.18	2.28	2.05	2.06	2.06	2.06	2.64
ICWC	C	0.32	2.59	1.16	0.49	1.55	0.33	2.60	0.33	2.60	0.37	2.64	1.65	1.66	1.67	1.66	2.63
mAmTH	mA	1.54	0.69	0.12	0.11	0.55	1.53	0.69	1.53	0.70	1.49	0.73	1.15	1.16	1.17	1.16	1.47
mAmTS	mA	11.91	6.36	4.89	4.02	10.16	11.93	6.37	11.95	6.39	11.91	6.35	0.45	0.48	0.49	0.59	1.82
mAmTWCAT	mA	0.40	2.19	2.15	2.17	1.09	0.41	2.19	0.42	2.20	0.44	2.21	3.24	3.26	3.26	3.25	3.19
mGmCS	mG	8.63	3.45	2.35	1.68	6.93	8.65	3.46	8.67	3.48	8.63	3.45	1.62	1.59	1.57	1.47	0.54
TA08316AAis	A	6.49	7.51	3.72	1.83	7.84	6.47	7.50	6.47	7.49	6.48	7.52	1.87	1.86	1.85	1.86	4.71
TA08316ATs	A	0.74	4.12	1.83	0.75	2.61	0.72	4.10	0.70	4.09	0.73	4.11	2.44	2.40	2.39	2.34	4.03
TA08316TTis	T	7.13	6.79	3.64	2.04	7.81	7.13	6.79	7.13	6.80	7.13	6.78	1.36	1.35	1.35	1.37	3.77
TG0319ACs	A	3.27	5.27	2.44	1.06	4.75	3.25	5.25	3.24	5.25	3.26	5.26	1.84	1.82	1.81	1.79	3.90
TG0319AGis	A	6.88	7.93	3.79	1.72	8.33	6.87	7.92	6.86	7.91	6.89	7.94	1.73	1.71	1.71	1.73	4.83
TG0319TCis	T	7.08	6.96	3.85	2.27	7.83	7.08	6.96	7.08	6.96	7.08	6.96	1.72	1.71	1.71	1.73	4.10
TG0319TGs	T	4.43	5.91	2.62	1.00	5.83	4.42	5.90	4.41	5.89	4.42	5.90	1.39	1.37	1.36	1.36	3.82
TT	mT	2.94	3.16	3.44	3.58	2.95	2.94	3.16	2.94	3.17	2.92	3.15	3.76	3.75	3.76	3.74	3.54
Uucalcutta	U	1.30	0.12	0.57	0.89	0.58	1.30	0.12	1.30	0.12	1.29	0.14	0.13	0.13	0.13	0.13	0.33
Uupl	U	0.87	0.88	0.03	0.45	0.03	0.87	0.88	0.87	0.88	0.86	0.88	0.47	0.48	0.48	0.47	1.08
Uust	U	6.65	4.50	2.23	1.04	6.41	6.66	4.50	6.67	4.51	6.65	4.49	0.61	0.60	0.59	0.56	1.22

DFT

Table S49: Absolute deviations for B97M-V, ωB97X-V, ωB97M-V, ωB97X-D3, B2PLYP-D3BJ, DSD-BLYP-D3BJ, ωB97X-D4 and B2PLYP-D4. Data: 82 interaction energies. All values in kcal/mol.

Dimer	Formula	B97M-V	ωB97X-V	ωB97M-V	ωB97X-D3	B2PLYP-D3BJ	DSD-BLYP-D3BJ	ωB97X-D4	B2PLYP-D4
8oxoG	oxo−G−C	4.54	2.83	2.76	1.89	1.94	2.25	1.47	4.54
AA0324AAs	A−A	3.05	3.65	2.42	2.59	4.67	3.90	3.01	3.05
AA0324ATis	T−A	3.47	3.93	3.42	2.83	5.83	5.79	3.00	3.47
AA0324TAis	A−T	3.38	3.93	3.42	2.81	5.82	5.78	2.98	3.38
AA0324TTs	T−T	2.55	2.80	1.97	1.91	3.50	2.83	2.43	2.55
AA20305AAs2005	A−A	3.10	3.66	2.41	2.61	4.60	3.77	3.01	3.10
AA20305ATis2005	T−A	3.43	4.03	3.48	2.91	5.86	5.83	2.87	3.43
AA20305TAis2005	A−T	3.35	3.77	3.24	2.74	5.56	5.48	2.56	3.35
AA20305TTs2005	T−T	2.50	2.59	1.86	1.71	3.41	2.78	2.32	2.50
AA	mA−mA	2.19	0.94	1.08	0.80	1.06	1.09	0.75	2.19
AAS	A−A	0.14	1.05	1.21	0.33	2.70	3.76	0.09	0.14
Acst	A−C	0.31	1.16	1.31	0.64	2.53	3.38	0.17	0.31
AG08319ACis	A−C	3.58	4.48	3.94	3.27	5.89	5.89	3.41	3.58
AG08319AGs	A−G	3.23	3.90	2.98	2.99	5.14	4.50	3.29	3.23
AG08319TCs	T−C	2.85	3.23	2.70	2.41	3.99	3.55	2.73	2.85
AG08319TGis	G−T	3.73	4.47	3.89	3.12	6.20	6.09	3.32	3.73
AT10326AAis	A−A	3.44	4.18	3.54	2.78	6.17	6.24	2.83	3.44
AT10326ATs	A−T	2.74	2.91	2.08	1.95	3.95	3.31	2.40	2.74
AT10326TTis	T−T	3.50	3.87	3.43	2.98	5.54	5.38	3.10	3.50
ATS1	A−T	0.70	0.18	1.12	0.09	1.70	2.89	0.79	0.70
ATS2	mA−mT	2.15	0.64	0.42	0.79	0.15	0.62	1.42	2.15
ATWC	A−T	1.75	0.93	0.89	0.38	0.15	0.06	0.10	1.75
Aust	A−U	0.01	1.18	1.32	0.63	2.62	3.41	0.11	0.01
CC10	C−C	0.40	1.03	1.05	0.82	2.17	2.67	0.27	0.40
CC11	C−C	0.48	1.13	1.12	0.70	2.19	2.72	0.30	0.48
CC12	C−C	0.41	1.01	0.59	0.09	1.52	1.79	0.24	0.41
CC13	C−C	0.43	0.98	0.89	0.84	1.84	2.32	0.36	0.43
CC14	C−C	0.35	1.16	1.19	0.80	2.21	2.74	0.41	0.35
CC1	C−C	0.05	0.82	0.86	0.49	2.42	2.89	0.01	0.05
CC2	C−C	0.02	1.05	1.21	0.68	2.48	3.06	0.16	0.02
CC3	C−C	0.15	1.14	1.20	0.73	2.30	2.87	0.37	0.15
CC4	C−C	0.36	1.18	1.19	0.76	2.25	2.85	0.37	0.36
CC5	C−C	0.04	0.84	0.94	0.41	2.32	2.90	0.00	0.04
CC6	C−C	0.02	0.88	1.00	0.48	2.39	3.00	0.03	0.02
CC7	C−C	0.10	0.82	0.68	0.42	1.92	2.24	0.16	0.10
CC8	C−C	0.29	1.19	0.96	0.50	1.93	2.45	0.44	0.29
CC9	C−C	0.39	1.24	1.36	1.01	2.41	2.99	0.42	0.39
CG0319CCis	G−G	1.39	0.54	1.43	2.40	1.10	0.74	1.66	1.39
CG0319GCs	C−G	3.60	4.50	3.75	3.83	5.51	5.03	3.70	3.60
CG0319GGis	C−C	8.84	9.98	9.53	8.98	10.73	10.65	9.19	8.84
Cust	C−U	0.02	1.23	1.22	0.71	2.24	2.73	0.36	0.02
F30K46	mPhe−mLys	7.33	6.48	6.46	6.06	7.17	7.16	6.18	7.33
F30L33	mPhe−mLeu	5.85	5.62	5.09	4.75	6.13	6.02	4.74	5.85
GA10315ACis	A−C	3.72	4.54	4.05	3.57	6.03	6.00	3.57	3.72
GA10315AGs	A−G	2.96	3.79	2.56	2.70	4.73	3.82	3.34	2.96
GA10315TCs	T−C	2.93	3.36	2.67	2.23	4.03	3.50	2.88	2.93
GA10315TGis	T−G	3.91	4.72	4.18	3.69	6.55	6.42	3.74	3.91
Gast	G−A	0.18	1.18	1.33	0.29	2.89	3.99	0.19	0.18
GC0325CCis	C−C	3.62	4.90	4.40	4.06	5.66	5.58	4.19	3.62
GC0325GCs	G−C	3.12	3.91	2.87	2.85	4.58	3.81	3.43	3.12
GC0325GGis	G−G	4.29	5.44	4.80	4.01	7.02	6.88	4.28	4.29
GCS	G−C	0.52	0.63	0.37	1.03	0.70	1.89	1.31	0.52
Gcst	G−C	0.09	1.42	1.39	0.95	2.73	3.36	0.28	0.09
GCWC1	C−G	2.78	2.23	2.06	1.38	1.07	1.21	0.96	2.78
GCWC2	G−C	8.02	6.92	6.63	5.93	6.38	6.61	5.55	8.02
GG0336CCs036	G−G	1.63	2.60	1.62	1.53	3.56	2.78	2.05	1.63
GG0336CGis036	G−C	3.82	4.77	4.30	3.55	6.32	6.29	3.74	3.82
GG0336GCis036	C−G	3.90	4.86	4.37	3.70	6.50	6.48	3.83	3.90
GG0336GGs036	C−C	5.13	6.10	5.46	5.01	6.29	5.84	5.47	5.13
Ggst	G−G	0.08	1.36	1.44	0.35	3.12	4.11	0.26	0.08
GT10315ACs	A−C	3.15	3.80	2.82	2.70	4.43	3.75	3.24	3.15
GT10315AGis	A−G	3.58	4.54	3.88	3.17	6.48	6.46	3.19	3.58
GT10315TCis	T−C	3.59	4.33	3.87	3.46	5.65	5.54	3.55	3.59
GT10315TGs	T−G	2.90	3.52	2.57	2.52	4.36	3.55	3.06	2.90
Gust	G−U	0.12	1.36	1.41	0.55	2.78	3.49	0.01	0.12
Guwobble	G−U	2.66	1.72	1.54	1.43	0.95	0.98	1.04	2.66
ICWC	C−oA	3.01	1.71	1.70	1.01	0.86	0.95	0.76	3.01
mAmTH	mA−mT	1.94	0.74	0.67	0.22	0.54	0.49	0.16	1.94
mAmTS	mA−mT	0.79	0.34	1.78	0.22	2.53	4.06	0.90	0.79
mAmTWCAT	mA−mT	2.99	1.52	1.60	1.09	0.85	1.02	0.77	2.99
mGmCS	mG−mC	1.12	1.77	0.33	2.32	0.10	1.42	2.94	1.12
TA08316AAis	A−A	3.56	4.34	3.45	3.05	5.80	5.60	2.98	3.56
TA08316ATs	A−T	4.02	4.78	3.31	4.04	5.16	4.08	4.63	4.02
TA08316TTis	T−T	3.56	3.92	3.47	3.07	5.66	5.49	3.12	3.56
TG0319ACs	A−C	3.15	3.96	3.05	3.00	4.81	4.30	3.15	3.15
TG0319AGis	A−G	3.56	4.28	3.47	2.71	6.01	5.82	3.10	3.56
TG0319TCis	T−C	3.60	4.27	3.83	3.34	5.60	5.50	3.48	3.60
TG0319TGs	T−G	3.41	4.03	3.28	3.29	5.33	4.80	3.38	3.41
TT	mT−mT	3.50	2.89	2.96	2.91	3.31	3.27	2.96	3.50
Uucalcutta	U−U	0.17	0.00	0.12	0.22	0.71	0.70	0.74	0.17
Uupl	U−U	0.85	0.35	0.12	0.08	0.31	0.27	0.39	0.85
Uust	U−U	0.19	1.22	1.27	0.87	2.43	2.86	0.29	0.19

SAPT-DFT

Table S50: Absolute deviations for SAPT-DFT. For SAPT-DFT method we used the B3LYP, PBE0 and ωB97X DFA's in conjunction with the aug-cc-pVDZ basis set. Data: 82 interaction energies. All values in kcal/mol.

Dimer	Formula	SAPT-B3LYP	SAPT-PBE0	SAPT-ωB97X
8oxoG	oxo−G−C	8.26	6.74	7.09
AA0324AAs	A−A	3.03	3.15	4.21
AA0324ATis	T−A	0.15	0.18	0.31
AA0324TAis	A−T	0.10	0.11	0.26
AA0324TTs	T−T	2.74	2.75	3.69
AA20305AAs2005	A−A	3.24	3.33	4.47
AA20305ATis2005	T−A	0.63	0.60	0.86
AA20305TAis2005	A−T	0.54	0.51	0.73
AA20305TTs2005	T−T	2.43	2.43	3.36
AA	mA−mA	0.80	0.81	1.06
AAS	A−A	3.02	3.06	4.06
Acst	A−C	2.98	2.96	3.62
AG08319ACis	A−C	0.40	0.38	0.57
AG08319AGs	A−G	2.21	2.50	3.31
AG08319TCs	T−C	1.69	1.90	2.47
AG08319TGis	G−T	0.88	0.80	1.17
AT10326AAis	A−A	0.56	0.55	0.82
AT10326ATs	A−T	2.48	2.52	3.45
AT10326TTis	T−T	0.12	0.10	0.23
ATS1	A−T	4.60	4.47	5.77
ATS2	mA−mT	3.02	3.07	3.99
ATWC	A−T	5.21	4.29	5.00
Aust	A−U	2.86	2.82	3.68
CC10	C−C	2.52	2.66	3.31
CC11	C−C	2.56	2.68	3.10
CC12	C−C	1.13	1.60	1.69
CC13	C−C	2.43	2.53	2.99
CC14	C−C	2.82	2.96	3.48
CC1	C−C	2.42	2.46	3.47
CC2	C−C	2.78	2.82	3.59
CC3	C−C	2.86	2.97	3.55
CC4	C−C	2.76	2.92	3.38
CC5	C−C	2.70	2.77	3.65
CC6	C−C	2.83	2.82	3.72
CC7	C−C	1.57	1.69	2.29
CC8	C−C	2.20	2.57	2.94
CC9	C−C	2.81	2.81	3.28
CG0319CCis	G−G	2.92	3.26	2.62
CG0319GCs	C−G	1.80	2.31	2.65
CG0319GGis	C−C	5.32	5.29	5.47
Cust	C−U	2.55	2.63	3.13
F30L33	mPhe−mLeu	1.61	1.68	2.92
GA10315ACis	A−C	0.38	0.45	0.61
GA10315AGs	A−G	3.46	3.49	4.76
GA10315TCs	T−C	2.14	2.14	2.81
GA10315TGis	T−G	0.33	0.34	0.49
Gast	G−A	3.21	3.27	4.12
GC0325CCis	C−C	0.03	0.06	0.24
GC0325GCs	G−C	3.13	3.21	4.01
GC0325GGis	G−G	0.88	0.85	1.23
GCS	G−C	4.73	4.54	5.40
Gcst	G−C	2.60	2.53	3.28
GCWC1	C−G	7.85	6.56	6.83
GCWC2	G−C	7.01	5.45	5.92
GG0336CCs036	G−G	0.88	1.07	2.05
GG0336CGis036	G−C	0.26	0.29	0.46
GG0336GCis036	C−G	0.04	0.06	0.09
GG0336GGs036	C−C	3.75	3.89	4.53
Ggst	G−G	3.36	3.40	4.27
GT10315ACs	A−C	2.76	2.85	3.63
GT10315AGis	A−G	0.73	0.74	1.13
GT10315TCis	T−C	0.03	0.02	0.10
GT10315TGs	T−G	3.27	3.26	4.31
Gust	G−U	2.93	2.91	3.68
Guwobble	G−U	5.84	4.79	5.13
ICWC	C−oA	6.06	4.82	5.19
mAmTH	mA−mT	5.33	4.36	5.06
mAmTS	mA−mT	6.86	6.58	8.18
mAmTWCAT	mA−mT	4.70	3.73	4.35
mGmCS	mG−mC	6.71	6.51	8.26
TA08316AAis	A−A	1.51	1.56	2.11
TA08316ATs	A−T	5.74	5.64	7.12
TA08316TTis	T−T	0.10	0.11	0.18
TG0319ACs	A−C	2.31	2.63	3.26
TG0319AGis	A−G	1.43	1.34	1.94
TG0319TCis	T−C	0.02	0.03	0.09
TG0319TGs	T−G	1.90	2.19	2.78
TT	mT−mT	0.09	0.07	0.23
Uucalcutta	U−U	3.00	2.58	2.80
Uupl	U−U	4.52	3.81	3.98
Uust	U−U	2.06	2.05	2.63

5.2 aug-cc-pVTZ basis set data.

5.2.1 Ionization Potentials and HOMO values for SAPT-DFT calculations

Table S51: Experimental ionization potentials (IP) of molecules comprising dimers in the JSCH-2005* database. IP obtained from NIST (https://webbook.nist.gov/chemistry). Theoretical IP obtained in this work with DLPNO-CCSD(T)/aug-cc-pVDZ (marked with *). Kohn-Sham energies of HOMO orbitals were calculated at the DFA/aug-cc-pVTZ level of theory (DFA=B3LYP, PBE0, and ωB97X). Ionization energies in ev and HOMO energies in atomic units.

			HOMO energies (eV)			$\Delta_{XC} = \epsilon_{HOMO} - (-IP)$		
Molecule	Formula	IP	B3LYP	PBE0	ωB97X	B3LYP	PBE0	ωB97X
$AA(9-methyl-adenine)$	$C_6H_6N_5CH_3$	7.90	-6.17	-6.44	-8.53	0.06	0.05	0.02
$Aas(Adenine)$	$C_5H_5N_5$	8.30	-6.20	-6.47	-8.56	0.08	0.07	0.01
$ACS(1-methylcytosine)$	$C_5H_6N_3O$	9.50	-6.37	-6.66	-8.84	0.12	0.10	0.02
$Acst(Cytosine)$	$C_4H_5N_3O$	8.45	-6.54	-6.84	-9.04	0.07	0.06	0.02
$AG(Thymine)$	$C_5H_6N_2O_2$	9.00	-6.87	-7.15	-9.32	0.08	0.07	0.01
$Ags(Guanine)$	$C_5H_5N_5O$	7.85	-5.86	-6.12	-8.19	0.07	0.06	0.01
$Atis(Thymine)$	$C_5H_6N_2O_2$	9.00	-6.87	-7.15	-9.32	0.08	0.07	0.01
$ATS2*(1-methylThymine)$	$C_6H_8N_2O_2$	8.81	-6.64	-6.91	-9.06	0.08	0.07	0.01
$Aust(Uracil)$	$C_4H_4N_2O_2$	9.20	-7.23	-7.52	-9.71	0.07	0.06	0.02
$CWC(Cytosine)$	$C_4H_5N_3O$	8.45	-6.55	-6.84	-9.04	0.07	0.06	0.02
$F30*(metil/fenilalanina)$	$C_9H_{11}NO_2$	9.19	-6.41	-6.71	-9.07	0.10	0.09	0.00
$GWC*(guanine)$	$C_5H_5N_5O$	7.91	-5.81	-6.06	-8.12	0.08	0.07	0.01
$ICWC*(adenine)$	$C_5H_5N_5$	8.68	-6.47	-6.73	-8.80	0.08	0.07	0.00
$K46*(methyl/lysine)$	$C_6H_{14}N_2O_2$	9.36	-6.43	-6.73	-9.20	0.11	0.10	0.01
$L33*(methyl/isoleucine)$	$C_6H_{13}NO_2$	9.51	-6.60	-6.90	-9.36	0.11	0.10	0.01
$mGm*(2-methylguanine)$	$C_6H_7N_5O$	8.03	-5.92	-6.18	-8.25	0.08	0.07	0.01
$V5C6(acetamide)$	C_2H_5NO	9.70	-6.92	-7.26	-9.75	0.10	0.09	0.00
$Y13*(methyl/Tyrosine)$	$C_9H_{11}NO_3$	8.50	-6.18	-6.47	-8.63	0.09	0.07	0.00

5.2.2 Evaluation of mean absolute deviation in a grid of C_{OS} and C_{SS} values

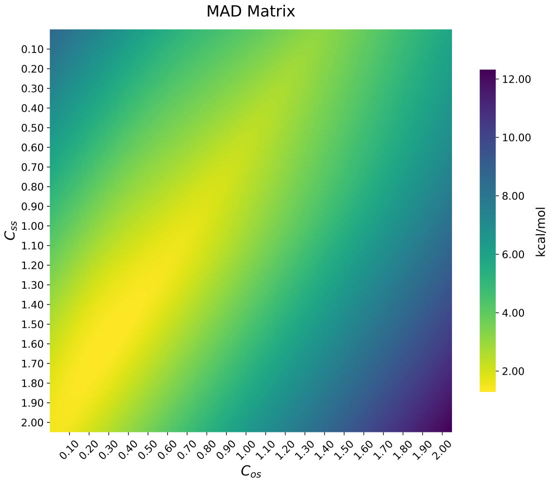

(a)

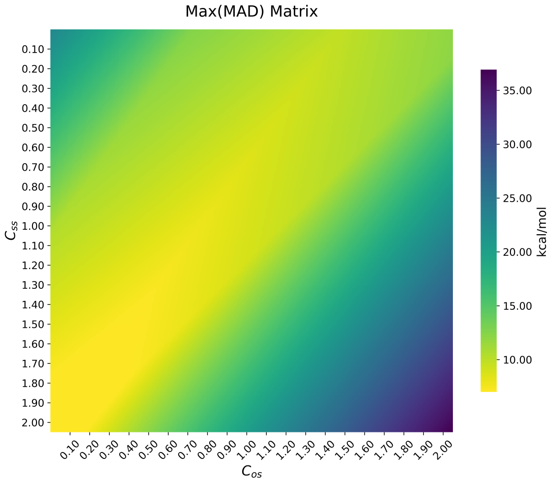

(b)

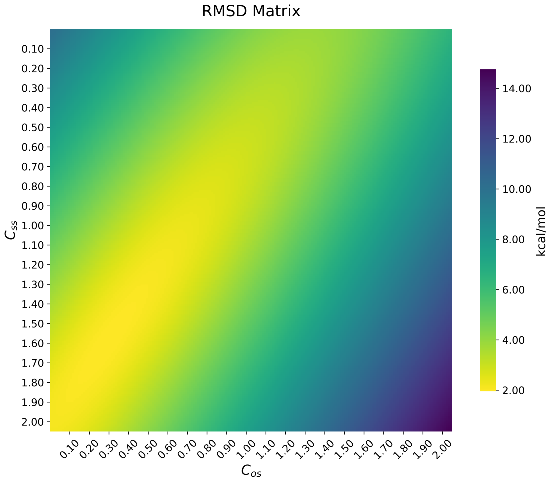

(c)

Figure S11: Evaluation of MADs (S11a), Max(MADs) (S11b) and RMSDs (S11c) in grid of values C_{OS} and C_{SS}. The optimal coefficients are C_{OS}=0.27 and C_{SS}=1.38. The theory level used is RIJK-MP2/aug-cc-pVDZ.

5.2.3 Interaction energies

MP2 with C_{OS} y C_{SS} values

Table S52: Interaction energies for CCSD(T)/CBS, MP2, SCS-MP2, SCS(MI)-MP2, SCSN-MP2, SCS-MP2-vdW, RI-MP2, RI-SCS-MP2, RIJK-MP2, RIJK-SCS-MP2, RIJCOSX-MP2, RIJCOSX-SCS-MP2, SCS-MP2^{BWI-TZ}, RI-SCS-MP2^{BWI-TZ}, RIJK-SCS-MP2^{BWI-TZ}, RIJCOSX-SCS-MP2^{BWI-DZ} and SCS-MP2-hal^{G-XZ}. Data: 82 interaction energies. MP2: $C_{OS} = C_{SS}$ =1.00; SCS-MP2: C_{OS} =1.20, C_{SS} =0.33; SCS(MI)-MP2: C_{OS} =0.40, C_{SS} =1.29; SCSN-MP2: C_{OS} =0.00, C_{SS} =1.76; SCS-MP2-vdW: C_{OS} =1.28, C_{SS} =0.50; SCS-MP2^{BWI-TZ} RI-SCS-MP2^{BWI-TZ} , RIJK-SCS-MP2^{BWI-TZ} : C_{OS} =0.27, C_{SS} =1.38; RIJCOSX-SCS-MP2^{BWI-TZ} : C_{OS} =0.17, C_{SS} =1.59. All values in kcal/mol.

Dimer	Formula	CCSD(T)/CBS	MP2	SCS-MP2	SCS(MI)-MP2	SCSN-MP2	SCS-MP2-vdW	RI-MP2	RI-SCS-MP2	RIJK-MP2	RIJK-SCS-MP2	RIJCOSX-MP2	RIJCOSX-SCS-MP2	SCS-MP2^{BWI-TZ}	RI-SCS-MP2^{BWI-TZ}	RIJK-SCS-MP2^{BWI-TZ}	RIJCOSX-SCS-MP2^{BWI-TZ}	SCS-MP2-hal^{G-XZ}
8oxoG	oxo	-33.30	-30.50	-27.81	-29.82	-30.77	-28.96	-30.50	-27.81	-30.49	-27.80	-30.07	-27.38	-29.80	-29.80	-29.80	-29.43	-27.15
AA0324AAs	A	-6.25	-6.10	-3.37	-6.69	-8.31	-4.21	-6.10	-3.37	-6.11	-3.39	-6.03	-3.30	-6.94	-6.94	-6.96	-6.95	-2.79
AA0324ATis	T	-1.71	2.25	2.18	-1.10	-2.76	3.06	2.25	2.18	2.26	2.18	2.28	2.21	-1.77	-1.77	-1.76	-1.76	1.91
AA0324TAis	A	-1.30	2.68	2.58	-0.66	-2.31	3.46	2.68	2.58	2.68	2.58	2.77	2.67	-1.33	-1.33	-1.33	-1.26	2.30
AA0324TTs	T	-3.86	-3.50	-1.44	-3.78	-4.91	-2.12	-3.50	-1.44	-3.51	-1.45	-3.45	-1.39	-3.93	-3.93	-3.94	-3.94	-0.99
AA20305AAs2005	A	-6.06	-6.19	-3.26	-6.57	-8.18	-4.23	-6.19	-3.26	-6.20	-3.28	-6.16	-3.24	-6.79	-6.79	-6.80	-6.84	-2.63
AA20305ATis2005	T	-2.34	1.67	1.77	-1.49	-3.13	2.54	1.67	1.77	1.68	1.77	1.74	1.83	-2.12	-2.12	-2.12	-2.09	1.55
AA20305TAis2005	A	-2.16	1.51	1.69	-1.63	-3.31	2.42	1.51	1.69	1.51	1.69	1.55	1.73	-2.27	-2.27	-2.27	-2.25	1.49
AA20305TTs2005	T	-4.18	-3.78	-1.87	-4.14	-5.25	-2.47	-3.78	-1.87	-3.79	-1.88	-3.73	-1.82	-4.31	-4.31	-4.32	-4.31	-1.47
AA	mA	-0.70	1.17	1.63	2.01	2.22	1.25	1.17	1.63	1.18	1.63	1.26	1.72	2.16	2.16	2.17	2.25	1.80
AAS	A	-8.58	-13.53	-10.52	-8.50	-7.41	-12.89	-13.53	-10.52	-13.55	-10.54	-13.45	-10.44	-7.64	-7.64	-7.66	-7.60	-9.46
Acst	A	-10.22	-13.94	-11.21	-9.55	-8.65	-13.32	-13.94	-11.21	-13.95	-11.23	-13.88	-11.15	-8.80	-8.80	-8.81	-8.78	-10.26
AG08319ACis	A	-0.18	3.80	4.01	0.97	-0.56	4.66	3.80	4.01	3.80	4.02	3.81	4.02	0.39	0.39	0.40	0.38	3.85
AG08319AGs	A	-7.58	-6.40	-4.17	-7.70	-9.44	-4.65	-6.40	-4.17	-6.41	-4.18	-6.38	-4.15	-8.07	-8.07	-8.08	-8.12	-3.76
AG08319TCs	T	-6.07	-4.66	-3.05	-5.48	-6.67	-3.43	-4.66	-3.05	-4.67	-3.06	-4.66	-3.05	-5.72	-5.72	-5.73	-5.76	-2.75
AG08319TGis	G	-0.47	3.60	3.91	0.62	-1.03	4.56	3.60	3.91	3.61	3.92	3.64	3.96	0.01	0.01	0.02	0.02	3.76
AT10326AAis	A	-0.92	3.31	3.52	-0.16	-2.02	4.33	3.31	3.52	3.31	3.52	3.37	3.58	-0.87	-0.87	-0.87	-0.85	3.31
AT10326ATs	A	-6.64	-6.14	-3.96	-6.83	-8.23	-4.58	-6.14	-3.96	-6.15	-3.97	-6.13	-3.96	-7.07	-7.07	-7.08	-7.13	-3.52
AT10326TTis	T	0.88	4.44	4.23	1.51	0.12	5.05	4.44	4.23	4.45	4.24	4.45	4.25	0.93	0.93	0.94	0.93	3.97
ATS1	A	-12.30	-17.33	-13.31	-13.00	-12.75	-15.87	-17.33	-13.31	-17.35	-13.33	-17.29	-13.27	-12.32	-12.32	-12.34	-12.36	-12.07
ATS2	mA	-8.10	-9.17	-6.35	-5.72	-5.34	-8.25	-9.17	-6.35	-9.18	-6.37	-9.10	-6.28	-5.16	-5.16	-5.18	-5.15	-5.45
ATWC	A	-16.86	-16.89	-14.97	-15.37	-15.53	-16.06	-16.89	-14.97	-16.88	-14.97	-16.52	-14.60	-15.16	-15.16	-15.15	-14.83	-14.42
Aust	A	-9.79	-13.51	-10.76	-9.04	-8.10	-12.90	-13.51	-10.76	-13.53	-10.78	-13.46	-10.71	-8.27	-8.27	-8.29	-8.26	-9.80
CC10	C	-8.27	-10.69	-8.39	-6.67	-5.75	-10.25	-10.69	-8.39	-10.71	-8.40	-10.67	-8.37	-5.97	-5.97	-5.99	-5.98	-7.56
CC11	C	-9.43	-11.65	-9.44	-7.90	-7.07	-11.20	-11.65	-9.44	-11.66	-9.45	-11.62	-9.40	-7.25	-7.25	-7.26	-7.24	-8.65
CC12	C	-7.43	-8.18	-7.03	-5.65	-4.93	-8.09	-8.18	-7.03	-8.19	-7.04	-8.16	-7.00	-5.20	-5.20	-5.21	-5.18	-6.58
CC13	C	-8.80	-10.72	-8.81	-7.10	-6.19	-10.42	-10.72	-8.81	-10.73	-8.82	-10.69	-8.78	-6.46	-6.46	-6.47	-6.46	-8.10
CC14	C	-9.11	-11.42	-8.98	-7.56	-6.78	-10.85	-11.42	-8.98	-11.43	-8.99	-11.40	-8.97	-6.90	-6.90	-6.91	-6.91	-8.14
CC1	C	2.45	-0.66	1.62	4.40	5.86	-0.49	-0.66	1.62	-0.67	1.61	-0.62	1.66	5.31	5.31	5.30	5.33	2.52
CC2	C	-3.85	-6.87	-4.40	-2.31	-1.20	-6.46	-6.87	-4.40	-6.87	-4.40	-6.82	-4.35	-1.52	-1.52	-1.52	-1.49	-3.49
CC3	C	-8.88	-11.44	-8.98	-7.32	-6.42	-10.92	-11.44	-8.98	-11.45	-8.99	-11.41	-8.95	-6.61	-6.61	-6.62	-6.61	-8.11
CC4	C	-9.92	-12.49	-10.07	-8.52	-7.69	-11.96	-12.49	-10.07	-12.50	-10.08	-12.45	-10.03	-7.84	-7.84	-7.85	-7.83	-9.22
CC5	C	0.32	-2.85	-0.46	2.09	3.43	-2.59	-2.85	-0.46	-2.86	-0.47	-2.82	-0.43	2.97	2.97	2.96	2.98	0.46
CC6	C	0.64	-2.60	-0.15	2.42	3.78	-2.32	-2.60	-0.15	-2.61	-0.17	-2.57	-0.13	3.32	3.32	3.30	3.32	0.78
CC7	C	-0.98	-2.75	-1.26	0.88	1.99	-2.72	-2.75	-1.26	-2.75	-1.27	-2.69	-1.20	1.53	1.53	1.53	1.58	-0.65
CC8	C	-9.10	-10.99	-9.04	-7.46	-6.62	-10.65	-10.99	-9.04	-11.01	-9.05	-10.98	-9.03	-6.85	-6.85	-6.86	-6.85	-8.33
CC9	C	-9.11	-11.74	-9.34	-7.80	-6.96	-11.22	-11.74	-9.34	-11.75	-9.36	-11.69	-9.29	-7.12	-7.12	-7.13	-7.09	-8.50
CG0319CCis	G	1.24	-0.77	0.71	-3.03	-4.89	0.74	-0.77	0.71	-0.77	0.71	-0.69	0.79	-3.55	-3.55	-3.55	-3.52	0.87
CG0319GCs	C	-7.88	-5.43	-3.68	-7.00	-8.63	-3.92	-5.43	-3.68	-5.43	-3.69	-5.36	-3.62	-7.40	-7.40	-7.40	-7.38	-3.41
CG0319GGis	C	-3.91	4.88	5.10	2.77	1.60	5.55	4.88	5.10	4.88	5.10	4.92	5.14	2.34	2.34	2.34	2.37	4.99
Cust	C	-10.42	-12.55	-10.26	-8.97	-8.27	-12.01	-12.55	-10.26	-12.57	-10.28	-12.54	-10.25	-8.36	-8.36	-8.37	-8.38	-9.47
F30K46	mPhe	-3.10	4.07	4.22	4.06	3.98	4.17	4.07	4.22	4.08	4.23	4.59	4.74	4.05	4.05	4.05	4.56	4.25
F30L33	mPhe	-5.00	0.43	2.03	1.57	1.37	1.16	0.43	2.03	0.44	2.04	0.87	2.47	1.72	1.72	1.73	2.13	2.48
GA10315ACis	A	-0.31	3.73	3.96	0.89	-0.65	4.60	3.73	3.96	3.73	3.97	3.75	3.99	0.31	0.31	0.32	0.31	3.80
GA10315AGs	A	-9.14	-9.14	-5.96	-9.41	-11.09	-7.05	-9.14	-5.96	-9.16	-5.97	-9.10	-5.91	-9.62	-9.62	-9.63	-9.66	-5.26
GA10315TCs	T	-4.69	-3.61	-1.82	-4.09	-5.20	-2.34	-3.61	-1.82	-3.62	-1.83	-3.56	-1.77	-4.28	-4.28	-4.29	-4.28	-1.45
GA10315TGis	T	0.58	4.93	4.87	1.61	-0.04	5.73	4.93	4.87	4.94	4.88	5.00	4.94	0.95	0.95	0.96	0.99	4.60
Gast	G	-11.38	-16.33	-13.18	-11.21	-10.15	-15.63	-16.33	-13.18	-16.36	-13.20	-16.28	-13.13	-10.34	-10.34	-10.36	-10.33	-12.07
GC0325CCis	C	3.09	6.79	6.93	4.71	3.59	7.41	6.79	6.93	6.79	6.94	6.81	6.96	4.28	4.28	4.29	4.29	6.81
GC0325GCs	G	-10.80	-10.06	-7.24	-10.34	-11.84	-8.19	-10.06	-7.24	-10.07	-7.25	-10.01	-7.20	-10.53	-10.53	-10.54	-10.56	-6.62
GC0325GGis	G	1.93	6.28	6.76	3.21	1.43	7.37	6.28	6.76	6.29	6.76	6.35	6.83	2.57	2.57	2.58	2.61	6.63
GCS	G	-19.02	-22.70	-18.88	-18.56	-18.31	-21.32	-22.70	-18.88	-22.72	-18.90	-22.65	-18.83	-17.91	-17.91	-17.93	-17.92	-17.69
Gcst	G	-10.60	-13.76	-11.26	-9.32	-8.28	-13.30	-13.76	-11.26	-13.77	-11.28	-13.70	-11.21	-8.55	-8.55	-8.56	-8.53	-10.36
GCWC1	C	-32.06	-30.66	-28.20	-29.36	-29.89	-29.43	-30.66	-28.20	-30.66	-28.20	-30.43	-27.97	-29.22	-29.22	-29.22	-29.04	-27.55
GCWC2	G	-31.40	-24.49	-22.16	-23.63	-24.32	-23.23	-24.49	-22.16	-24.48	-22.15	-24.25	-21.91	-23.57	-23.57	-23.56	-23.38	-21.56
GG0336CCs036	G	-1.62	-2.50	0.06	-3.30	-4.94	-0.67	-2.50	0.06	-2.51	0.04	-2.44	0.12	-3.58	-3.58	-3.60	-3.60	0.58
GG0336CGis036	G	-3.68	0.77	1.04	-2.35	-4.06	1.74	0.77	1.04	0.78	1.04	0.79	1.06	-2.99	-2.99	-2.99	-3.00	0.86
GG0336GCis036	C	-4.82	-0.08	-0.02	-3.53	-5.29	0.83	-0.08	-0.02	-0.08	-0.02	0.00	0.06	-4.22	-4.22	-4.22	-4.17	-0.27
GG0336GGs036	C	-3.54	-0.26	1.56	-0.42	-1.38	0.94	-0.26	1.56	-0.27	1.56	-0.21	1.61	-0.54	-0.54	-0.54	-0.53	1.96
Ggst	G	-12.67	-17.30	-14.10	-11.96	-10.81	-16.62	-17.30	-14.10	-17.32	-14.12	-17.25	-14.05	-11.04	-11.04	-11.06	-11.03	-12.97
GT10315ACs	A	-5.44	-4.78	-2.33	-5.09	-6.42	-3.14	-4.78	-2.33	-4.79	-2.35	-4.76	-2.32	-5.26	-5.26	-5.28	-5.32	-1.80
GT10315AGis	A	-4.06	0.26	0.72	-3.08	-4.99	1.40	0.26	0.72	0.26	0.72	0.34	0.81	-3.77	-3.77	-3.76	-3.72	0.57
GT10315TCis	T	0.30	4.06	3.99	1.39	0.07	4.70	4.06	3.99	4.06	3.99	4.07	4.00	0.86	0.86	0.86	0.85	3.77
GT10315TGs	T	-4.96	-4.55	-1.98	-4.76	-6.10	-2.86	-4.55	-1.98	-4.56	-1.99	-4.51	-1.94	-4.92	-4.92	-4.93	-4.96	-1.41
Gust	G	-12.09	-15.42	-12.65	-10.99	-10.09	-14.79	-15.42	-12.65	-15.44	-12.66	-15.37	-12.60	-10.23	-10.23	-10.25	-10.22	-11.69
Guwobble	G	-19.10	-18.22	-16.12	-16.97	-17.35	-17.20	-18.22	-16.12	-18.22	-16.11	-17.97	-15.87	-16.82	-16.82	-16.81	-16.61	-15.54
ICWC	C	-24.90	-23.88	-21.64	-23.00	-23.64	-22.68	-23.88	-21.64	-23.87	-21.63	-23.72	-21.48	-22.93	-22.93	-22.92	-22.82	-21.06
mAmTH	mA	-18.16	-18.60	-16.39	-17.06	-17.35	-17.58	-18.60	-16.39	-18.59	-16.38	-18.02	-15.81	-16.86	-16.86	-16.85	-16.32	-15.77
mAmTS	mA	-14.57	-21.25	-15.69	-15.44	-15.19	-19.19	-21.25	-15.69	-21.27	-15.71	-21.10	-15.54	-14.54	-14.54	-14.56	-14.51	-13.99
mAmTWCAT	mA	-16.40	-14.63	-12.86	-13.05	-13.10	-13.91	-14.63	-12.86	-14.62	-12.85	-14.36	-12.59	-12.81	-12.81	-12.80	-12.57	-12.34
mGmCS	mG	-20.35	-24.08	-18.89	-18.85	-18.71	-22.11	-24.08	-18.89	-24.09	-18.91	-23.98	-18.80	-18.05	-18.05	-18.06	-18.05	-17.32
TA08316AAis	A	-1.55	1.37	2.45	-1.03	-2.77	2.67	1.37	2.45	1.37	2.46	1.44	2.52	-1.56	-1.56	-1.56	-1.55	2.52
TA08316ATs	A	-6.07	-5.80	-2.35	-5.04	-6.32	-3.80	-5.80	-2.35	-5.81	-2.36	-5.72	-2.28	-5.05	-5.05	-5.06	-5.07	-1.51
TA08316TTis	T	0.70	4.35	4.07	1.35	-0.03	4.94	4.35	4.07	4.36	4.08	4.49	4.21	0.76	0.76	0.77	0.89	3.78
TG0319ACs	A	-4.96	-3.56	-1.51	-4.38	-5.78	-2.05	-3.56	-1.51	-3.56	-1.51	-3.53	-1.49	-4.64	-4.64	-4.64	-4.67	-1.11
TG0319AGis	A	-4.22	-0.89	0.22	-3.56	-5.45	0.50	-0.89	0.22	-0.89	0.22	-0.76	0.34	-4.15	-4.15	-4.15	-4.08	0.27
TG0319TCis	T	-1.15	2.65	2.58	-0.11	-1.47	3.31	2.65	2.58	2.65	2.58	2.68	2.62	-0.66	-0.66	-0.66	-0.64	2.36
TG0319TGs	T	-5.67	-3.47	-1.93	-5.20	-6.81	-2.05	-3.47	-1.93	-3.47	-1.93	-3.40	-1.87	-5.62	-5.62	-5.62	-5.61	-1.72
TT	mT	1.00	4.57	4.86	4.96	5.02	4.66	4.57	4.86	4.58	4.87	4.66	4.95	5.03	5.03	5.03	5.11	4.95
Uucalcutta	U	-10.30	-10.78	-9.37	-10.10	-10.43	-10.06	-10.78	-9.37	-10.78	-9.36	-10.55	-9.13	-10.03	-10.03	-10.02	-9.82	-8.99
Uupl	U	-13.70	-13.99	-12.25	-13.11	-13.51	-13.11	-13.99	-12.25	-13.99	-12.24	-13.86	-12.11	-13.02	-13.02	-13.01	-12.92	-11.79
Uust	U	-7.46	-9.95	-7.81	-6.32	-5.52	-9.52	-9.95	-7.81	-9.97	-7.83	-9.97	-7.83	-5.70	-5.70	-5.71	-5.74	-7.06

DFT

Table S53: Interaction energies for CCSD(T)/CBS B97M-V, ωB97X-V, ωB97M-V, ωB97X-D3, B2PLYP-D3BJ, DSD-BLYP-D3BJ, ωB97X-D4 and B2PLYP-D4. Data: 82 interaction energies. All values in kcal/mol.

Dimer	Formula	CCSD(T)/CBS	B97M-V	ωB97X-V	ωB97M-V	ωB97X-D3	B2PLYP-D3BJ	DSD-BLYP-D3BJ	ωB97X-D4	B2PLYP-D4
8oxoG	oxo−G−C	-33.30	-28.40	-29.45	-29.31	-30.21	-30.32	-30.14	-30.77	-30.35
AA0324AAs	A−A	-6.25	-5.44	-4.31	-5.50	-5.42	-3.58	-4.22	-5.21	-4.32
AA0324ATis	T−A	-1.71	-0.68	0.00	-0.62	-1.24	1.34	1.06	-1.03	1.19
AA0324TAis	A−T	-1.30	-0.35	0.40	-0.22	-0.86	1.72	1.44	-0.66	1.60
AA0324TTs	T−T	-3.86	-2.68	-2.12	-3.02	-3.14	-1.51	-2.09	-2.69	-1.78
AA20305AAs2005	A−A	-6.06	-5.16	-4.05	-5.25	-5.14	-3.36	-4.05	-4.97	-4.18
AA20305ATis2005	T−A	-2.34	-1.34	-0.47	-1.13	-1.72	0.77	0.49	-1.76	0.60
AA20305TAis2005	A−T	-2.16	-1.17	-0.53	-1.18	-1.69	0.71	0.41	-1.86	0.54
AA20305TTs2005	T−T	-4.18	-3.03	-2.60	-3.38	-3.62	-1.88	-2.41	-3.09	-2.17
AA	mA−mA	-0.70	1.79	0.78	1.02	0.66	1.18	1.32	0.57	1.08
AAS	A−A	-8.58	-6.63	-7.65	-7.63	-6.81	-8.49	-8.94	-6.64	-9.11
Acst	A−C	-10.22	-7.91	-9.23	-9.15	-8.57	-9.84	-10.14	-8.34	-10.24
AG08319ACis	A−C	-0.18	1.13	2.23	1.58	0.91	3.08	2.84	1.06	2.97
AG08319AGs	A−G	-7.58	-6.67	-5.55	-6.51	-6.55	-4.58	-5.14	-6.39	-5.36
AG08319TCs	T−C	-6.07	-4.73	-4.01	-4.63	-4.95	-3.39	-3.79	-4.68	-3.69
AG08319TGis	G−T	-0.47	0.92	1.89	1.18	0.41	3.01	2.67	0.62	2.87
AT10326AAis	A−A	-0.92	-0.23	0.79	0.06	-0.73	2.15	1.95	-0.69	1.94
AT10326ATs	A−T	-6.64	-5.66	-5.09	-5.93	-6.13	-4.21	-4.73	-5.81	-4.73
AT10326TTis	T−T	0.88	2.26	2.80	2.21	1.76	3.95	3.57	1.93	3.86
ATS1	A−T	-12.30	-11.69	-10.99	-11.80	-10.65	-11.95	-12.63	-10.23	-12.39
ATS2	mA−mT	-8.10	-4.74	-5.87	-5.90	-5.63	-5.59	-5.89	-5.15	-6.06
ATWC	A−T	-16.86	-14.57	-15.10	-14.98	-15.52	-16.06	-16.02	-15.89	-16.17
Aust	A−U	-9.79	-7.84	-8.93	-8.89	-8.27	-9.63	-9.87	-7.98	-10.02
CC10	C−C	-8.27	-5.95	-7.21	-7.02	-6.86	-7.72	-7.76	-6.52	-8.02
CC11	C−C	-9.43	-6.98	-8.44	-8.22	-7.88	-8.81	-8.89	-7.67	-9.09
CC12	C−C	-7.43	-5.45	-6.83	-6.31	-5.83	-6.62	-6.57	-6.04	-6.95
CC13	C−C	-8.80	-6.47	-7.79	-7.51	-7.50	-8.04	-8.09	-7.19	-8.26
CC14	C−C	-9.11	-6.81	-8.10	-7.92	-7.60	-8.49	-8.55	-7.41	-8.80
CC1	C−C	2.45	4.51	3.74	3.87	4.14	2.74	2.69	4.50	2.45
CC2	C−C	-3.85	-1.85	-2.77	-2.73	-2.30	-3.51	-3.61	-1.96	-3.77
CC3	C−C	-8.88	-6.73	-7.87	-7.73	-7.34	-8.33	-8.42	-7.17	-8.63
CC4	C−C	-9.92	-7.58	-8.96	-8.77	-8.41	-9.31	-9.43	-8.20	-9.61
CC5	C−C	0.32	2.37	1.58	1.64	2.08	0.73	0.60	2.33	0.46
CC6	C−C	0.64	2.69	1.91	1.95	2.38	1.07	0.92	2.66	0.79
CC7	C−C	-0.98	0.90	-0.02	0.25	0.46	-0.49	-0.46	0.64	-0.68
CC8	C−C	-9.10	-7.03	-8.38	-8.00	-7.57	-8.34	-8.41	-7.65	-8.73
CC9	C−C	-9.11	-6.64	-8.12	-7.99	-7.75	-8.62	-8.73	-7.35	-8.79
CG0319CCis	G−G	1.24	-2.56	-1.35	-2.35	-3.35	-0.27	-0.75	-2.69	-0.56
CG0319GCs	C−G	-7.88	-6.40	-5.12	-5.95	-5.88	-4.35	-4.84	-6.08	-4.89
CG0319GGis	C−C	-3.91	3.11	4.39	3.81	3.27	4.65	4.37	3.54	4.66
Cust	C−U	-10.42	-8.51	-9.64	-9.48	-9.04	-9.96	-9.98	-8.87	-10.24
F30K46	mPhe−mLys	-3.10	4.05	3.18	3.25	2.87	4.06	4.07	3.06	4.21
F30L33	mPhe−mLeu	-5.00	0.75	0.77	0.48	0.01	1.44	1.38	0.05	1.64
GA10315ACis	A−C	-0.31	1.13	2.15	1.53	1.04	3.10	2.84	1.07	2.92
GA10315AGs	A−G	-9.14	-8.32	-6.92	-8.15	-8.09	-6.19	-6.91	-7.68	-7.04
GA10315TCs	T−C	-4.69	-3.20	-2.43	-3.18	-3.66	-1.98	-2.44	-3.09	-2.27
GA10315TGis	T−G	0.58	2.04	3.03	2.34	1.83	4.24	3.85	1.94	4.10
Gast	G−A	-11.38	-9.24	-10.48	-10.45	-9.46	-11.27	-11.71	-9.25	-11.90
GC0325CCis	C−C	3.09	4.86	6.28	5.64	5.30	6.53	6.25	5.50	6.51
GC0325GCs	G−C	-10.80	-9.38	-8.14	-9.19	-9.28	-7.66	-8.27	-8.88	-8.21
GC0325GGis	G−G	1.93	3.63	4.95	4.14	3.31	5.92	5.51	3.60	5.74
GCS	G−C	-19.02	-17.79	-16.75	-17.64	-16.24	-17.68	-18.39	-16.19	-18.03
Gcst	G−C	-10.60	-8.44	-9.82	-9.64	-9.28	-10.37	-10.48	-8.81	-10.67
GCWC1	C−G	-32.06	-28.52	-28.76	-28.72	-29.43	-29.93	-29.88	-29.96	-30.06
GCWC2	G−C	-31.40	-22.95	-23.65	-23.71	-24.44	-24.33	-24.26	-24.95	-24.43
GG0336CCs036	G−G	-1.62	-2.18	-0.80	-1.89	-2.03	-0.12	-0.79	-1.65	-0.92
GG0336CGis036	G−C	-3.68	-2.15	-1.00	-1.61	-2.37	-0.01	-0.28	-2.15	-0.16
GG0336GCis036	C−G	-4.82	-3.25	-2.10	-2.70	-3.40	-1.05	-1.33	-3.22	-1.14
GG0336GGs036	C−C	-3.54	0.16	1.44	0.71	0.27	1.39	0.99	0.66	1.11
Ggst	G−G	-12.67	-10.52	-11.83	-11.77	-10.73	-12.59	-12.90	-10.39	-13.20
GT10315ACs	A−C	-5.44	-4.01	-2.94	-3.92	-4.09	-2.55	-3.09	-3.72	-3.10
GT10315AGis	A−G	-4.06	-3.22	-1.97	-2.72	-3.46	-0.64	-0.91	-3.44	-0.90
GT10315TCis	T−C	0.30	1.88	2.80	2.20	1.80	3.59	3.27	1.94	3.52
GT10315TGs	T−G	-4.96	-3.79	-2.77	-3.79	-3.89	-2.06	-2.72	-3.49	-2.62
Gust	G−U	-12.09	-10.17	-11.34	-11.27	-10.46	-11.92	-12.05	-10.11	-12.30
Guwobble	G−U	-19.10	-15.70	-16.43	-16.48	-16.57	-17.18	-17.21	-17.06	-17.32
ICWC	C−oA	-24.90	-21.35	-22.31	-22.12	-22.82	-23.10	-23.09	-23.20	-23.27
mAmTH	mA−mT	-18.16	-15.72	-16.45	-16.33	-16.84	-17.55	-17.57	-17.32	-17.62
mAmTS	mA−mT	-14.57	-13.76	-12.84	-14.06	-12.21	-14.13	-15.08	-11.86	-14.48
mAmTWCAT	mA−mT	-16.40	-12.68	-13.74	-13.45	-14.03	-14.23	-14.10	-14.43	-14.34
mGmCS	mG−mC	-20.35	-17.48	-16.56	-17.80	-15.91	-17.53	-18.47	-15.59	-17.93
TA08316AAis	A−A	-1.55	-0.59	0.53	-0.42	-0.86	1.49	1.11	-1.00	1.14
TA08316ATs	A−T	-6.07	-3.64	-2.37	-3.80	-3.19	-2.03	-2.91	-2.83	-2.65
TA08316TTis	T−T	0.70	2.14	2.68	2.09	1.68	3.86	3.46	1.78	3.80
TG0319ACs	A−C	-4.96	-3.80	-2.57	-3.52	-3.59	-2.00	-2.47	-3.56	-2.52
TG0319AGis	A−G	-4.22	-3.19	-2.12	-2.99	-3.78	-0.94	-1.29	-3.46	-1.24
TG0319TCis	T−C	-1.15	0.45	1.29	0.73	0.24	2.11	1.80	0.43	2.07
TG0319TGs	T−G	-5.67	-4.40	-3.47	-4.33	-4.33	-2.41	-2.95	-4.31	-2.92
TT	mT−mT	1.00	5.00	4.38	4.50	4.46	4.89	4.86	4.54	4.82
Uucalcutta	U−U	-10.30	-9.55	-9.62	-9.66	-9.76	-10.25	-10.23	-10.35	-10.32
Uupl	U−U	-13.70	-12.27	-12.61	-12.73	-12.77	-13.28	-13.28	-13.34	-13.46
Uust	U−U	-7.46	-5.73	-6.77	-6.65	-6.37	-7.37	-7.39	-5.98	-7.53

SAPT-DFT

Table S54: Interaction energies for CCSD(T)/CBS and SAPT-DFT. For SAPT-DFT method we used the B3LYP, PBE0 and ωB97X DFA's in conjunction with the aug-cc-pVDZ basis set. Data: 82 interaction energies. All values in kcal/mol.

Dimer	Formula	CCSD(T)/CBS	SAPT-B3LYP	SAPT-PBE0	SAPT-ωB97X
8oxoG	oxo−G−C	-33.30	-26.63	-28.08	-27.49
AA0324AAs	A−A	-6.25	-3.82	-3.67	-2.59
AA0324ATis	T−A	-1.71	-1.63	-1.59	-1.45
AA0324TAis	A−T	-1.30	-1.28	-1.26	-1.10
AA0324TTs	T−T	-3.86	-1.81	-1.77	-0.80
AA20305AAs2005	A−A	-6.06	-3.46	-3.34	-2.18
AA20305ATis2005	T−A	-2.34	-1.89	-1.91	-1.64
AA20305TAis2005	A−T	-2.16	-1.79	-1.80	-1.57
AA20305TTs2005	T−T	-4.18	-2.32	-2.29	-1.34
AA	mA−mA	-0.70	-0.11	-0.08	0.18
AAS	A−A	-8.58	-6.13	-6.05	-5.03
Acst	A−C	-10.22	-7.80	-7.77	-7.08
AG08319ACis	A−C	-0.18	0.12	0.11	0.32
AG08319AGs	A−G	-7.58	-5.87	-5.57	-4.74
AG08319TCs	T−C	-6.07	-4.85	-4.63	-4.04
AG08319TGis	G−T	-0.47	0.20	0.14	0.52
AT10326AAis	A−A	-0.92	-0.51	-0.50	-0.21
AT10326ATs	A−T	-6.64	-4.70	-4.64	-3.67
AT10326TTis	T−T	0.88	0.95	0.94	1.06
ATS1	A−T	-12.30	-8.61	-8.69	-7.33
ATS2	mA−mT	-8.10	-5.68	-5.61	-4.65
ATWC	A−T	-16.86	-12.63	-13.51	-12.65
Aust	A−U	-9.79	-7.52	-7.51	-6.62
CC10	C−C	-8.27	-6.35	-6.17	-5.50
CC11	C−C	-9.43	-7.42	-7.27	-6.81
CC12	C−C	-7.43	-6.67	-6.23	-6.10
CC13	C−C	-8.80	-6.86	-6.73	-6.23
CC14	C−C	-9.11	-6.89	-6.72	-6.17
CC1	C−C	2.45	4.28	4.34	5.36
CC2	C−C	-3.85	-1.68	-1.61	-0.82
CC3	C−C	-8.88	-6.65	-6.51	-5.89
CC4	C−C	-9.92	-7.74	-7.55	-7.05
CC5	C−C	0.32	2.43	2.52	3.41
CC6	C−C	0.64	2.86	2.89	3.79
CC7	C−C	-0.98	0.19	0.32	0.93
CC8	C−C	-9.10	-7.45	-7.08	-6.67
CC9	C−C	-9.11	-6.86	-6.81	-6.31
CG0319CCis	G−G	1.24	-2.10	-2.39	-1.72
CG0319GCs	C−G	-7.88	-6.59	-6.09	-5.72
CG0319GGis	C−C	-3.91	1.36	1.34	1.53
Cust	C−U	-10.42	-8.45	-8.34	-7.79
F30L33	mPhe−mLeu	-5.00	-3.89	-3.81	-2.52
GA10315ACis	A−C	-0.31	-0.04	0.04	0.21
GA10315AGs	A−G	-9.14	-6.41	-6.35	-5.04
GA10315TCs	T−C	-4.69	-3.02	-3.00	-2.30
GA10315TGis	T−G	0.58	0.83	0.84	1.00
Gast	G−A	-11.38	-8.78	-8.69	-7.79
GC0325CCis	C−C	3.09	3.10	3.12	3.30
GC0325GCs	G−C	-10.80	-8.40	-8.30	-7.46
GC0325GGis	G−G	1.93	2.68	2.68	3.06
GCS	G−C	-19.02	-15.43	-15.55	-14.60
Gcst	G−C	-10.60	-8.57	-8.59	-7.81
GCWC1	C−G	-32.06	-25.63	-26.86	-26.37
GCWC2	G−C	-31.40	-26.08	-27.59	-26.86
GG0336CCs036	G−G	-1.62	-1.27	-1.07	-0.07
GG0336CGis036	G−C	-3.68	-3.56	-3.51	-3.33
GG0336GCis036	C−G	-4.82	-4.84	-4.81	-4.77
GG0336GGs036	C−C	-3.54	-0.22	-0.06	0.60
Ggst	G−G	-12.67	-9.99	-9.91	-8.98
GT10315ACs	A−C	-5.44	-3.23	-3.12	-2.31
GT10315AGis	A−G	-4.06	-3.57	-3.54	-3.14
GT10315TCis	T−C	0.30	0.30	0.29	0.37
GT10315TGs	T−G	-4.96	-2.40	-2.38	-1.29
Gust	G−U	-12.09	-9.80	-9.78	-8.95
Guwobble	G−U	-19.10	-14.48	-15.46	-14.96
ICWC	C−oA	-24.90	-20.04	-21.23	-20.66
mAmTH	mA−mT	-18.16	-13.82	-14.76	-13.91
mAmTS	mA−mT	-14.57	-9.06	-9.27	-7.61
mAmTWCAT	mA−mT	-16.40	-12.68	-13.62	-12.85
mGmCS	mG−mC	-20.35	-15.11	-15.25	-13.40
TA08316AAis	A−A	-1.55	-0.39	-0.32	0.25
TA08316ATs	A−T	-6.07	-1.24	-1.31	0.19
TA08316TTis	T−T	0.70	0.77	0.78	0.85
TG0319ACs	A−C	-4.96	-3.21	-2.88	-2.23
TG0319AGis	A−G	-4.22	-3.12	-3.18	-2.55
TG0319TCis	T−C	-1.15	-1.16	-1.16	-1.09
TG0319TGs	T−G	-5.67	-4.25	-3.96	-3.34
TT	mT−mT	1.00	1.03	1.02	-3.08
Uucalcutta	U−U	-10.30	-7.93	-8.31	-8.00
Uupl	U−U	-13.70	-10.13	-10.80	-10.51
Uust	U−U	-7.46	-5.92	-5.90	-5.28

5.2.4 Absolute deviations of molecular systems.

MP2 with C_{OS} y C_{SS} values

Table S55: Absolute deviations for MP2, SCS-MP2, SCS(MI)-MP2, SCSN-MP2, SCS-MP2-vdW, RI-MP2, RI-SCS-MP2, RIJK-MP2, RIJK-SCS-MP2, RIJCOSX-MP2, RIJCOSX-SCS-MP2, SCS-MP2^{BWI-TZ}, RI-SCS-MP2^{BWI-TZ}, RIJK-SCS-MP2^{BWI-TZ}, RIJCOSX-SCS-MP2^{BWI-DZ} and SCS-MP2-hal^{G-XZ}. Data: 82 interaction energies. MP2: C_{OS} = C_{SS} =1.00; SCS-MP2: C_{OS} =1.20, C_{SS} =0.33; SCS(MI)-MP2: C_{OS} =0.40, C_{SS} =1.29; SCSN-MP2: C_{OS} =0.00, C_{SS} =1.76; SCS-MP2-vdW: C_{OS} =1.28, C_{SS} =0.50; SCS-MP2^{BWI-TZ} RI-SCS-MP2^{BWI-TZ} , RIJK-SCS-MP2^{BWI-TZ} : C_{OS} =0.27, C_{SS} =1.38; RIJCOSX-SCS-MP2^{BWI-TZ} : C_{OS} =0.17, C_{SS} =1.59. All values in kcal/mol.

Dimer	Formula	MP2	SCS-MP2	SCS(MI)-MP2	SCSN-MP2	SCS-MP2-vdW	RI-MP2	RI-SCS-MP2	RIJK-MP2	RIJK-SCS-MP2	RIJCOSX-MP2	RIJCOSX-SCS-MP2	SCS-MP2^{BWI-TZ}	RI-SCS-MP2^{BWI-TZ}	RIJK-SCS-MP2^{BWI-TZ}	RIJCOSX-SCS-MP2^{BWI-TZ}	SCS-MP2-hal^{G-XZ}
8oxoG	oxo	2.80	5.49	3.48	2.53	4.34	2.80	5.49	2.81	5.50	3.23	5.92	3.50	3.50	3.50	3.87	6.15
AA0324AAs	A	0.15	2.88	0.44	2.06	2.04	0.15	2.88	0.14	2.86	0.22	2.95	0.69	0.69	0.71	0.70	3.46
AA0324ATis	T	3.96	3.89	0.61	1.05	4.77	3.96	3.89	3.97	3.89	3.99	3.92	0.06	0.06	0.05	0.05	3.62
AA0324TAis	A	3.98	3.88	0.64	1.01	4.76	3.98	3.88	3.98	3.88	4.07	3.97	0.03	0.03	0.03	0.04	3.60
AA0324TTs	T	0.36	2.42	0.08	1.05	1.74	0.36	2.42	0.35	2.41	0.41	2.47	0.07	0.07	0.08	0.08	2.87
AA20305AAs2005	A	0.13	2.80	0.51	2.12	1.83	0.13	2.80	0.14	2.78	0.10	2.82	0.73	0.73	0.74	0.78	3.43
AA20305ATis2005	T	4.01	4.11	0.85	0.79	4.88	4.01	4.11	4.02	4.11	4.08	4.17	0.22	0.22	0.22	0.25	3.89
AA20305TAis2005	A	3.67	3.85	0.53	1.15	4.58	3.67	3.85	3.67	3.85	3.71	3.89	0.11	0.11	0.11	0.09	3.65
AA20305TTs2005	T	0.40	2.31	0.04	1.07	1.71	0.40	2.31	0.39	2.30	0.45	2.36	0.13	0.13	0.14	0.13	2.71
AA	mA	1.87	2.33	2.71	2.92	1.95	1.87	2.33	1.88	2.33	1.96	2.42	2.86	2.86	2.87	2.95	2.50
AAS	A	4.95	1.94	0.08	1.17	4.31	4.95	1.94	4.97	1.96	4.87	1.86	0.94	0.94	0.92	0.98	0.88
Acst	A	3.72	0.99	0.67	1.57	3.10	3.72	0.99	3.73	1.01	3.66	0.93	1.42	1.42	1.41	1.44	0.04
AG08319ACis	A	3.98	4.19	1.15	0.38	4.84	3.98	4.19	3.98	4.20	3.99	4.20	0.57	0.57	0.58	0.56	4.03
AG08319AGs	A	1.18	3.41	0.12	1.86	2.93	1.18	3.41	1.17	3.40	1.20	3.43	0.49	0.49	0.50	0.54	3.82
AG08319TCs	T	1.41	3.02	0.59	0.60	2.64	1.41	3.02	1.40	3.01	1.41	3.02	0.35	0.35	0.34	0.31	3.32
AG08319TGis	G	4.07	4.38	1.09	0.56	5.03	4.07	4.38	4.08	4.39	4.11	4.43	0.48	0.48	0.49	0.49	4.23
AT10326AAis	A	4.23	4.44	0.76	1.10	5.25	4.23	4.44	4.23	4.44	4.29	4.50	0.05	0.05	0.05	0.07	4.23
AT10326ATs	A	0.50	2.68	0.19	1.59	2.06	0.50	2.68	0.49	2.67	0.51	2.68	0.43	0.43	0.44	0.49	3.12
AT10326TTis	T	3.56	3.35	0.63	0.76	4.17	3.56	3.35	3.57	3.36	3.57	3.37	0.05	0.05	0.06	0.05	3.09
ATS1	A	5.03	1.01	0.70	0.45	3.57	5.03	1.01	5.05	1.03	4.99	0.97	0.02	0.02	0.04	0.06	0.23
ATS2	mA	1.07	1.75	2.38	2.76	0.15	1.07	1.75	1.08	1.73	1.00	1.82	2.94	2.94	2.92	2.95	2.65
ATWC	A	0.03	1.89	1.49	1.33	0.80	0.03	1.89	0.02	1.89	0.34	2.26	1.70	1.70	1.71	2.03	2.44
Aust	A	3.72	0.97	0.75	1.69	3.11	3.72	0.97	3.74	0.99	3.67	0.92	1.52	1.52	1.50	1.53	0.01
CC10	C	2.42	0.12	1.60	2.52	1.98	2.42	0.12	2.44	0.13	2.40	0.10	2.30	2.30	2.28	2.29	0.71
CC11	C	2.22	0.01	1.53	2.36	1.77	2.22	0.01	2.23	0.02	2.19	0.03	2.18	2.18	2.17	2.19	0.78
CC12	C	0.75	0.40	1.78	2.50	0.66	0.75	0.40	0.76	0.39	0.73	0.43	2.23	2.23	2.22	2.25	0.85
CC13	C	1.92	0.01	1.70	2.61	1.62	1.92	0.01	1.93	0.02	1.89	0.02	2.34	2.34	2.33	2.34	0.70
CC14	C	2.31	0.13	1.55	2.33	1.74	2.31	0.13	2.32	0.12	2.29	0.14	2.21	2.21	2.20	2.20	0.97
CC1	C	3.11	0.83	1.95	3.41	2.94	3.11	0.83	3.12	0.84	3.07	0.79	2.86	2.86	2.85	2.88	0.07
CC2	C	3.02	0.55	1.54	2.65	2.61	3.02	0.55	3.02	0.55	2.97	0.50	2.33	2.33	2.33	2.36	0.36
CC3	C	2.56	0.10	1.56	2.46	2.04	2.56	0.10	2.57	0.11	2.53	0.07	2.27	2.27	2.26	2.27	0.77
CC4	C	2.57	0.15	1.40	2.23	2.04	2.57	0.15	2.58	0.16	2.53	0.11	2.08	2.08	2.07	2.09	0.70
CC5	C	3.17	0.78	1.77	3.11	2.91	3.17	0.78	3.18	0.79	3.14	0.75	2.65	2.65	2.64	2.66	0.14
CC6	C	3.24	0.79	1.78	3.14	2.96	3.24	0.79	3.25	0.81	3.21	0.77	2.68	2.68	2.66	2.68	0.14
CC7	C	1.77	0.28	1.86	2.97	1.74	1.77	0.28	1.77	0.29	1.71	0.22	2.51	2.51	2.51	2.56	0.33
CC8	C	1.89	0.06	1.64	2.48	1.55	1.89	0.06	1.91	0.05	1.88	0.07	2.25	2.25	2.24	2.25	0.77
CC9	C	2.63	0.23	1.31	2.15	2.11	2.63	0.23	2.64	0.25	2.58	0.18	1.99	1.99	1.98	2.02	0.61
CG0319CCis	G	2.01	0.53	4.27	6.13	0.50	2.01	0.53	2.01	0.53	1.93	0.45	4.79	4.79	4.79	4.76	0.37
CG0319GCs	C	2.45	4.20	0.88	0.75	3.96	2.45	4.20	2.45	4.19	2.52	4.26	0.48	0.48	0.48	0.50	4.47
CG0319GGis	C	8.79	9.01	6.68	5.51	9.46	8.79	9.01	8.79	9.01	8.83	9.05	6.25	6.25	6.25	6.28	8.90
Cust	C	2.13	0.16	1.45	2.15	1.59	2.13	0.16	2.15	0.14	2.12	0.17	2.06	2.06	2.05	2.04	0.95
F30K46	mPhe	7.17	7.32	7.16	7.08	7.27	7.17	7.32	7.18	7.33	7.69	7.84	7.15	7.15	7.15	7.66	7.35
F30L33	mPhe	5.43	7.03	6.57	6.37	6.16	5.43	7.03	5.44	7.04	5.87	7.47	6.72	6.72	6.73	7.13	7.48
GA10315ACis	A	4.04	4.27	1.20	0.34	4.91	4.04	4.27	4.04	4.28	4.06	4.30	0.62	0.62	0.63	0.62	4.11
GA10315AGs	A	0.00	3.18	0.27	1.95	2.09	0.00	3.18	0.02	3.17	0.04	3.23	0.48	0.48	0.49	0.52	3.88
GA10315TCs	T	1.08	2.87	0.60	0.51	2.35	1.08	2.87	1.07	2.86	1.13	2.92	0.41	0.41	0.40	0.41	3.24
GA10315TGis	T	4.35	4.29	1.03	0.62	5.15	4.35	4.29	4.36	4.30	4.42	4.36	0.37	0.37	0.38	0.41	4.02
Gast	G	4.95	1.80	0.17	1.23	4.25	4.95	1.80	4.98	1.82	4.90	1.75	1.04	1.04	1.02	1.05	0.69
GC0325CCis	C	3.70	3.84	1.62	0.50	4.32	3.70	3.84	3.70	3.85	3.72	3.87	1.19	1.19	1.20	1.20	3.72
GC0325GCs	G	0.74	3.56	0.46	1.04	2.61	0.74	3.56	0.73	3.55	0.79	3.60	0.27	0.27	0.26	0.24	4.18
GC0325GGis	G	4.35	4.83	1.28	0.50	5.44	4.35	4.83	4.36	4.83	4.42	4.90	0.64	0.64	0.65	0.68	4.70
GCS	G	3.68	0.14	0.46	0.71	2.30	3.68	0.14	3.70	0.12	3.63	0.19	1.11	1.11	1.09	1.10	1.33
Gcst	G	3.16	0.66	1.28	2.32	2.70	3.16	0.66	3.17	0.68	3.10	0.61	2.05	2.05	2.04	2.07	0.24
GCWC1	C	1.40	3.86	2.70	2.17	2.63	1.40	3.86	1.40	3.86	1.63	4.09	2.84	2.84	2.84	3.02	4.51
GCWC2	G	6.91	9.24	7.77	7.08	8.17	6.91	9.24	6.92	9.25	7.15	9.49	7.83	7.83	7.84	8.02	9.84
GG0336CCs036	G	0.88	1.68	1.68	3.32	0.95	0.88	1.68	0.89	1.66	0.82	1.74	1.96	1.96	1.98	1.98	2.20
GG0336CGis036	G	4.45	4.72	1.33	0.38	5.42	4.45	4.72	4.46	4.72	4.47	4.74	0.69	0.69	0.69	0.68	4.54
GG0336GCis036	C	4.74	4.80	1.29	0.47	5.65	4.74	4.80	4.74	4.80	4.82	4.88	0.60	0.60	0.60	0.65	4.55
GG0336GGs036	C	3.28	5.10	3.12	2.16	4.48	3.28	5.10	3.27	5.10	3.33	5.15	3.00	3.00	3.00	3.01	5.50
Ggst	G	4.63	1.43	0.71	1.86	3.95	4.63	1.43	4.65	1.45	4.58	1.38	1.63	1.63	1.61	1.64	0.30
GT10315ACs	A	0.66	3.11	0.35	0.98	2.30	0.66	3.11	0.65	3.09	0.68	3.12	0.18	0.18	0.16	0.12	3.64
GT10315AGis	A	4.32	4.78	0.98	0.93	5.46	4.32	4.78	4.32	4.78	4.40	4.87	0.29	0.29	0.30	0.34	4.63
GT10315TCis	T	3.76	3.69	1.09	0.23	4.40	3.76	3.69	3.76	3.69	3.77	3.70	0.56	0.56	0.56	0.55	3.47
GT10315TGs	T	0.41	2.98	0.20	1.14	2.10	0.41	2.98	0.40	2.97	0.45	3.02	0.04	0.04	0.03	0.00	3.55
Gust	G	3.33	0.56	1.10	2.00	2.70	3.33	0.56	3.35	0.57	3.28	0.51	1.86	1.86	1.84	1.87	0.40
Guwobble	G	0.88	2.98	2.13	1.75	1.90	0.88	2.98	0.88	2.99	1.13	3.23	2.28	2.28	2.29	2.49	3.56
ICWC	C	1.02	3.26	1.90	1.26	2.22	1.02	3.26	1.03	3.27	1.18	3.42	1.97	1.97	1.98	2.08	3.84
mAmTH	mA	0.44	1.77	1.10	0.81	0.58	0.44	1.77	0.43	1.78	0.14	2.35	1.30	1.30	1.31	1.84	2.39
mAmTS	mA	6.68	1.12	0.87	0.62	4.62	6.68	1.12	6.70	1.14	6.53	0.97	0.03	0.03	0.01	0.06	0.58
mAmTWCAT	mA	1.77	3.54	3.35	3.30	2.49	1.77	3.54	1.78	3.55	2.04	3.81	3.59	3.59	3.60	3.83	4.06
mGmCS	mG	3.73	1.46	1.50	1.64	1.76	3.73	1.46	3.74	1.44	3.63	1.55	2.30	2.30	2.29	2.30	3.03
TA08316AAis	A	2.92	4.00	0.52	1.22	4.22	2.92	4.00	2.92	4.01	2.99	4.07	0.01	0.01	0.01	0.00	4.07
TA08316ATs	A	0.27	3.72	1.03	0.25	2.27	0.27	3.72	0.26	3.71	0.35	3.79	1.02	1.02	1.01	1.00	4.56
TA08316TTis	T	3.65	3.37	0.65	0.73	4.24	3.65	3.37	3.66	3.38	3.79	3.51	0.06	0.06	0.07	0.19	3.08
TG0319ACs	A	1.40	3.45	0.58	0.82	2.91	1.40	3.45	1.40	3.45	1.43	3.47	0.32	0.32	0.32	0.29	3.85
TG0319AGis	A	3.33	4.44	0.66	1.23	4.72	3.33	4.44	3.33	4.44	3.46	4.56	0.07	0.07	0.07	0.14	4.49
TG0319TCis	T	3.80	3.73	1.04	0.32	4.46	3.80	3.73	3.80	3.73	3.83	3.77	0.49	0.49	0.49	0.51	3.51
TG0319TGs	T	2.20	3.74	0.47	1.14	3.62	2.20	3.74	2.20	3.74	2.27	3.80	0.05	0.05	0.05	0.06	3.95
TT	mT	3.57	3.86	3.96	4.02	3.66	3.57	3.86	3.58	3.87	3.66	3.95	4.03	4.03	4.03	4.11	3.95
Ucalcutta	U	0.48	0.93	0.20	0.13	0.24	0.48	0.93	0.48	0.94	0.25	1.17	0.27	0.27	0.28	0.48	1.31
Uupl	U	0.29	1.45	0.59	0.19	0.59	0.29	1.45	0.29	1.46	0.16	1.59	0.68	0.68	0.69	0.78	1.91
Uust	U	2.49	0.35	1.14	1.94	2.06	2.49	0.35	2.51	0.37	2.51	0.37	1.76	1.76	1.75	1.72	0.40

DFT

Table S56: Absolute deviations for B97M-V, ωB97X-V, ωB97M-V, ωB97X-D3, B2PLYP-D3BJ, DSD-BLYP-D3BJ, ωB97X-D4 and B2PLYP-D4. Data: 82 interaction energies. All values in kcal/mol.

Dimer	Formula	B97M-V	ωB97X-V	ωB97M-V	ωB97X-D3	B2PLYP-D3BJ	DSD-BLYP-D3BJ	ωB97X-D4	B2PLYP-D4
8oxoG	oxo−G−C	4.90	3.85	3.99	3.09	2.98	3.16	2.53	2.95
AA0324AAs	A−A	0.81	1.94	0.75	0.83	2.67	2.03	1.04	1.93
AA0324ATis	T−A	1.03	1.71	1.09	0.47	3.05	2.77	0.68	2.90
AA0324TAis	A−T	0.95	1.70	1.08	0.44	3.02	2.74	0.64	2.90
AA0324TTs	T−T	1.18	1.74	0.84	0.72	2.35	1.77	1.17	2.08
AA20305AAs2005	A−A	0.90	2.01	0.81	0.92	2.70	2.01	1.09	1.88
AA20305ATis2005	T−A	1.00	1.87	1.21	0.62	3.11	2.83	0.58	2.94
AA20305TAis2005	A−T	0.99	1.63	0.98	0.47	2.87	2.57	0.30	2.70
AA20305TTs2005	T−T	1.15	1.58	0.80	0.56	2.30	1.77	1.09	2.01
AA	mA−mA	2.49	1.48	1.72	1.36	1.88	2.02	1.27	1.78
AAS	A−A	1.95	0.93	0.95	1.77	0.09	0.36	1.94	0.53
Acst	A−C	2.31	0.99	1.07	1.65	0.38	0.08	1.88	0.02
AG08319ACis	A−C	1.31	2.41	1.76	1.09	3.26	3.02	1.24	3.15
AG08319AGs	A−G	0.91	2.03	1.07	1.03	3.00	2.44	1.19	2.22
AG08319TCs	T−C	1.34	2.06	1.44	1.12	2.68	2.28	1.39	2.38
AG08319TGis	G−T	1.39	2.36	1.65	0.88	3.48	3.14	1.09	3.34
AT10326AAis	A−A	0.69	1.71	0.98	0.19	3.07	2.87	0.23	2.86
AT10326ATs	A−T	0.98	1.55	0.71	0.51	2.43	1.91	0.83	1.91
AT10326TTis	T−T	1.38	1.92	1.33	0.88	3.07	2.69	1.05	2.98
ATS1	A−T	0.61	1.31	0.50	1.65	0.35	0.33	2.07	0.09
ATS2	mA−mT	3.36	2.23	2.20	2.47	2.51	2.21	2.95	2.04
ATWC	A−T	2.29	1.76	1.88	1.34	0.80	0.84	0.97	0.69
Aust	A−U	1.95	0.86	0.90	1.52	0.16	0.08	1.81	0.23
CC10	C−C	2.32	1.06	1.25	1.41	0.55	0.51	1.75	0.25
CC11	C−C	2.45	0.99	1.21	1.55	0.62	0.54	1.76	0.34
CC12	C−C	1.98	0.60	1.12	1.60	0.81	0.86	1.39	0.48
CC13	C−C	2.33	1.01	1.29	1.30	0.76	0.71	1.61	0.54
CC14	C−C	2.30	1.01	1.19	1.51	0.62	0.56	1.70	0.31
CC1	C−C	2.06	1.29	1.42	1.69	0.29	0.24	2.05	0.00
CC2	C−C	2.00	1.08	1.12	1.55	0.34	0.24	1.89	0.08
CC3	C−C	2.15	1.01	1.15	1.54	0.55	0.46	1.71	0.25
CC4	C−C	2.34	0.96	1.15	1.51	0.61	0.49	1.72	0.31
CC5	C−C	2.05	1.26	1.32	1.76	0.41	0.28	2.01	0.14
CC6	C−C	2.05	1.27	1.31	1.74	0.43	0.28	2.02	0.15
CC7	C−C	1.88	0.96	1.23	1.44	0.49	0.52	1.62	0.30
CC8	C−C	2.07	0.72	1.10	1.53	0.76	0.69	1.45	0.37
CC9	C−C	2.47	0.99	1.12	1.36	0.49	0.38	1.76	0.32
CG0319CCis	G−G	3.80	2.59	3.59	4.59	1.51	1.99	3.93	1.80
CG0319GCs	C−G	1.48	2.76	1.93	2.00	3.53	3.04	1.80	2.99
CG0319GGis	C−C	7.02	8.30	7.72	7.18	8.56	8.28	7.45	8.57
Cust	C−U	1.91	0.78	0.94	1.38	0.46	0.44	1.55	0.18
F30K46	mPhe−mLys	7.15	6.28	6.35	5.97	7.16	7.17	6.16	7.31
F30L33	mPhe−mLeu	5.75	5.77	5.48	5.01	6.44	6.38	5.05	6.64
GA10315ACis	A−C	1.44	2.46	1.84	1.35	3.41	3.15	1.38	3.23
GA10315AGs	A−G	0.82	2.22	0.99	1.05	2.95	2.23	1.46	2.10
GA10315TCs	T−C	1.49	2.26	1.51	1.03	2.71	2.25	1.60	2.42
GA10315TGis	T−G	1.46	2.45	1.76	1.25	3.66	3.27	1.36	3.52
Gast	G−A	2.14	0.90	0.93	1.92	0.11	0.33	2.13	0.52
GC0325CCis	C−C	1.77	3.19	2.55	2.21	3.44	3.16	2.41	3.42
GC0325GCs	G−C	1.42	2.66	1.61	1.52	3.14	2.53	1.92	2.59
GC0325GGis	G−G	1.70	3.02	2.21	1.38	3.99	3.58	1.67	3.81
GCS	G−C	1.23	2.27	1.38	2.78	1.34	0.63	2.83	0.99
Gcst	G−C	2.16	0.78	0.96	1.32	0.23	0.12	1.79	0.07
GCWC1	C−G	3.54	3.30	3.34	2.63	2.13	2.18	2.10	2.00
GCWC2	G−C	8.45	7.75	7.69	6.96	7.07	7.14	6.45	6.97
GG0336CCs036	G−G	0.56	0.82	0.27	0.41	1.50	0.83	0.03	0.70
GG0336CGis036	G−C	1.53	2.68	2.07	1.31	3.67	3.40	1.53	3.52
GG0336GCis036	C−G	1.57	2.72	2.12	1.42	3.77	3.49	1.60	3.68
GG0336GGs036	C−C	3.70	4.98	4.25	3.81	4.93	4.53	4.20	4.65
Ggst	G−G	2.15	0.84	0.90	1.94	0.08	0.23	2.28	0.53
GT10315ACs	A−C	1.43	2.50	1.52	1.35	2.89	2.35	1.72	2.34
GT10315AGis	A−G	0.84	2.09	1.34	0.60	3.42	3.15	0.62	3.16
GT10315TCis	T−C	1.58	2.50	1.90	1.50	3.29	2.97	1.64	3.22
GT10315TGs	T−G	1.17	2.19	1.17	1.07	2.90	2.24	1.47	2.34
Gust	G−U	1.92	0.75	0.82	1.63	0.17	0.04	1.98	0.21
Guwobble	G−U	3.40	2.67	2.62	2.53	1.92	1.89	2.04	1.78
ICWC	C−oA	3.55	2.59	2.78	2.08	1.80	1.81	1.70	1.63
mAmTH	mA−mT	2.44	1.71	1.83	1.32	0.61	0.59	0.84	0.54
mAmTS	mA−mT	0.81	1.73	0.51	2.36	0.44	0.51	2.71	0.09
mAmTWCAT	mA−mT	3.72	2.66	2.95	2.37	2.17	2.30	1.97	2.06
mGmCS	mG−mC	2.87	3.79	2.55	4.44	2.82	1.88	4.76	2.42
TA08316AAis	A−A	0.96	2.08	1.13	0.69	3.04	2.66	0.55	2.69
TA08316ATs	A−T	2.43	3.70	2.27	2.88	4.04	3.16	3.24	3.42
TA08316TTis	T−T	1.44	1.98	1.39	0.98	3.16	2.76	1.08	3.10
TG0319ACs	A−C	1.16	2.39	1.44	1.37	2.96	2.49	1.40	2.44
TG0319AGis	A−G	1.03	2.10	1.23	0.44	3.28	2.93	0.76	2.98
TG0319TCis	T−C	1.60	2.44	1.88	1.39	3.26	2.95	1.58	3.22
TG0319TGs	T−G	1.27	2.20	1.34	1.34	3.26	2.72	1.36	2.75
TT	mT−mT	4.00	3.38	3.50	3.46	3.89	3.86	3.54	3.82
Uucalcutta	U−U	0.75	0.68	0.64	0.54	0.05	0.07	0.05	0.02
Uupl	U−U	1.43	1.09	0.97	0.93	0.42	0.42	0.36	0.24
Uust	U−U	1.73	0.69	0.81	1.09	0.09	0.07	1.48	0.07

SAPT-DFT

Table S57: Absolute deviations for SAPT-DFT. For SAPT-DFT method we used the B3LYP, PBE0 and ωB97X DFA's in conjunction with the aug-cc-pVTZ basis set. Data: 82 interaction energies. All values in kcal/mol.

Dimer	Formula	SAPT-B3LYP	SAPT-PBE0	SAPT-ωB97X
8oxoG	oxo−G−C	6.67	5.22	5.81
AA0324AAs	A−A	2.43	2.58	3.66
AA0324ATis	T−A	0.08	0.12	0.26
AA0324TAis	A−T	0.02	0.04	0.20
AA0324TTs	T−T	2.05	2.09	3.06
AA20305AAs2005	A−A	2.60	2.72	3.88
AA20305ATis2005	T−A	0.45	0.43	0.70
AA20305TAis2005	A−T	0.37	0.36	0.59
AA20305TTs2005	T−T	1.86	1.89	2.84
AA	mA−mA	0.59	0.62	0.88
AAS	A−A	2.45	2.53	3.55
Acst	A−C	2.42	2.45	3.14
AG08319ACis	A−C	0.30	0.29	0.50
AG08319AGs	A−G	1.71	2.01	2.84
AG08319TCs	T−C	1.22	1.44	2.03
AG08319TGis	G−T	0.67	0.61	0.99
AT10326AAis	A−A	0.41	0.42	0.71
AT10326ATs	A−T	1.94	2.00	2.97
AT10326TTis	T−T	0.07	0.06	0.18
ATS1	A−T	3.69	3.61	4.97
ATS2	mA−mT	2.42	2.49	3.45
ATWC	A−T	4.23	3.35	4.21
Aust	A−U	2.27	2.28	3.17
CC10	C−C	1.92	2.10	2.77
CC11	C−C	2.01	2.16	2.62
CC12	C−C	0.76	1.20	1.33
CC13	C−C	1.94	2.07	2.57
CC14	C−C	2.22	2.39	2.94
CC1	C−C	1.83	1.89	2.91
CC2	C−C	2.17	2.24	3.03
CC3	C−C	2.23	2.37	2.99
CC4	C−C	2.18	2.37	2.87
CC5	C−C	2.11	2.20	3.09
CC6	C−C	2.22	2.25	3.15
CC7	C−C	1.17	1.30	1.91
CC8	C−C	1.65	2.02	2.43
CC9	C−C	2.25	2.30	2.80
CG0319CCis	G−G	3.34	3.63	2.96
CG0319GCs	C−G	1.29	1.79	2.16
CG0319GGis	C−C	5.27	5.25	5.44
Cust	C−U	1.97	2.08	2.63
F30L33	mPhe−mLeu	1.11	1.19	2.48
GA10315ACis	A−C	0.27	0.35	0.52
GA10315AGs	A−G	2.73	2.79	4.10
GA10315TCs	T−C	1.67	1.69	2.39
GA10315TGis	T−G	0.25	0.26	0.42
Gast	G−A	2.60	2.69	3.59
GC0325CCis	C−C	0.01	0.03	0.21
GC0325GCs	G−C	2.40	2.50	3.34
GC0325GGis	G−G	0.75	0.75	1.13
GCS	G−C	3.59	3.47	4.42
Gcst	G−C	2.03	2.01	2.79
GCWC1	C−G	6.43	5.20	5.69
GCWC2	G−C	5.32	3.81	4.54
GG0336CCs036	G−G	0.35	0.55	1.55
GG0336CGis036	G−C	0.12	0.17	0.35
GG0336GCis036	C−G	0.02	0.01	0.05
GG0336GGs036	C−C	3.32	3.48	4.14
Ggst	G−G	2.68	2.76	3.69
GT10315ACs	A−C	2.21	2.32	3.13
GT10315AGis	A−G	0.49	0.52	0.92
GT10315TCis	T−C	0.00	0.01	0.07
GT10315TGs	T−G	2.56	2.58	3.67
Gust	G−U	2.29	2.31	3.14
Guwobble	G−U	4.62	3.64	4.14
ICWC	C−oA	4.86	3.67	4.24
mAmTH	mA−mT	4.34	3.40	4.25
mAmTS	mA−mT	5.51	5.30	6.96
mAmTWCAT	mA−mT	3.72	2.78	3.55
mGmCS	mG−mC	5.24	5.10	6.95
TA08316AAis	A−A	1.16	1.23	1.80
TA08316ATs	A−T	4.83	4.76	6.26
TA08316TTis	T−T	0.07	0.08	0.15
TG0319ACs	A−C	1.75	2.08	2.73
TG0319AGis	A−G	1.10	1.04	1.67
TG0319TCis	T−C	0.01	0.01	0.06
TG0319TGs	T−G	1.42	1.71	2.33
TT	mT−mT	0.03	0.02	4.08
Uucalcutta	U−U	2.37	1.99	2.30
Uupl	U−U	3.57	2.90	3.19
Uust	U−U	1.54	1.56	2.18

5.3 Mean calculation times

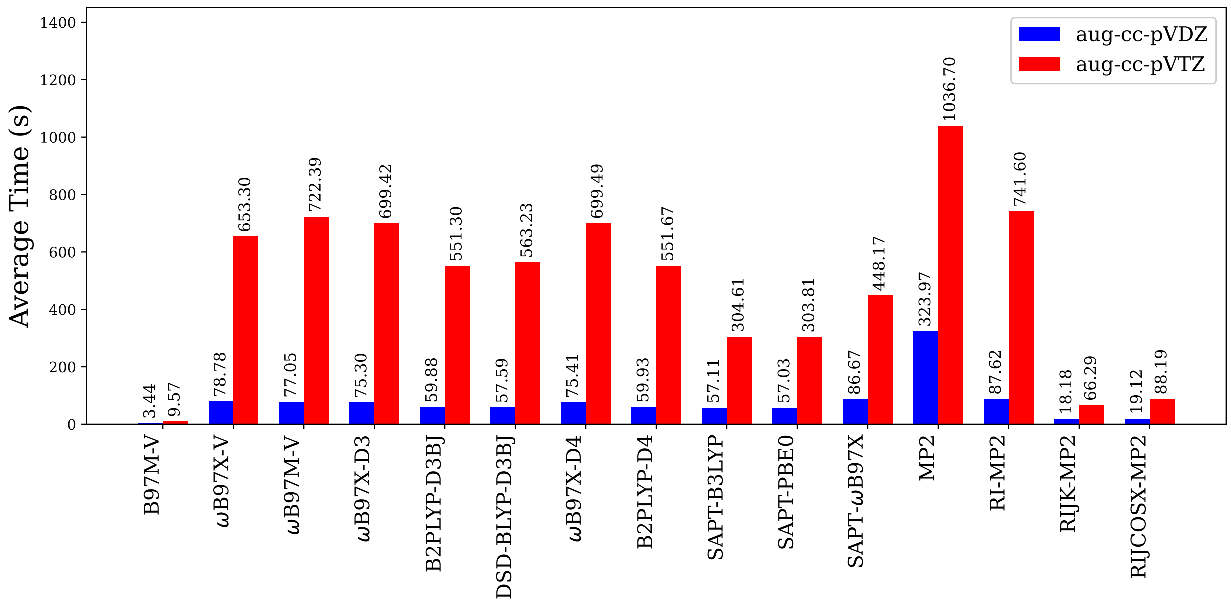

Figure S12: Comparison of computational times for different methods and basis sets in the dataset JSCH-2005*. All values in seconds.

6 S66 dataset

6.1 aug-cc-pVDZ basis set data.

6.1.1 Ionization Potentials and HOMO values for SAPT-DFT calculations

Table S58: Experimental ionization potentials (IP) of molecules comprising dimers in S66 the database. IP obtained from NIST (https://webbook.nist.gov/chemistry). Kohn-Sham energies of HOMO orbitals were calculated at the DFA/aug-cc-pVDZ level of theory (DFA=B3LYP, PBE0, and ωB97X). Ionization energies in ev and HOMO energies in atomic units.

			HOMO energies (eV)			$\Delta_{XC} = \epsilon_{HOMO} - (-IP)$		
Molecule	Formula	IP	B3LYP	PBE0	ωB97X	B3LYP	PBE0	ωB97X
acetamide	CH_3CONH_2	10.00	-7.02	-7.36	-10.69	0.1094	0.0971	0.0254
aceticacid	CH_3COOH	10.65	-7.84	-8.18	-9.87	0.10	0.09	0.03
benzene	C_6H_6	9.24	-6.97	-7.28	-9.48	0.08	0.07	0.01
cyclopentane	C_5H_{10}	10.33	-8.45	-8.71	-11.19	0.07	0.06	0.03
ethene	C_2H_4	10.51	-7.54	-7.86	-10.29	0.11	0.10	0.01
ethyne	C_2H_2	11.40	-8.05	-8.39	-10.86	0.12	0.11	0.02
methylamide	CH_3NH_2	8.90	-6.62	-6.93	-9.45	0.08	0.07	0.02
methanol	CH_3OH	10.84	-7.63	-7.96	-10.50	0.12	0.11	0.01
neopentane	C_5H_{12}	10.90	-8.69	-8.97	-11.42	0.08	0.07	0.02
pentane	C_5H_{12}	10.28	-8.54	-8.84	-11.27	0.06	0.05	0.04
peptide	C_2H_5NO	9.70	-6.90	-7.24	-9.71	0.10	0.09	0.00
pyridine	C_5H_5N	9.26	-7.14	-7.48	-9.84	0.08	0.07	0.02
uracil	$C_4H_4N_2O_2$	9.20	-7.20	-7.49	-9.71	0.07	0.06	0.02
water	H_2O	12.62	-8.72	-9.07	-11.69	0.14	0.13	0.03

6.1.2 Evaluation of mean absolute deviation in a grid of C_{OS} and C_{SS} values

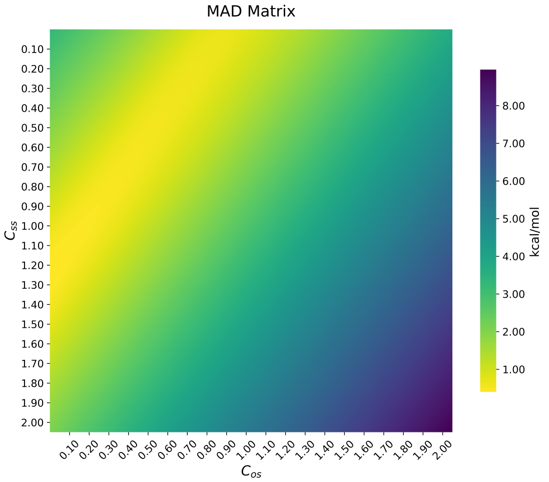

(a)

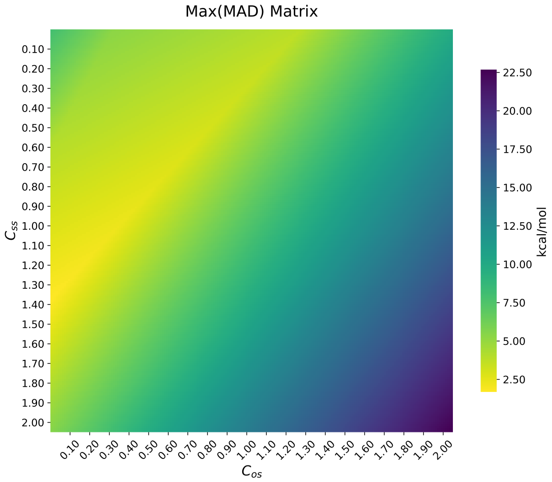

(b)

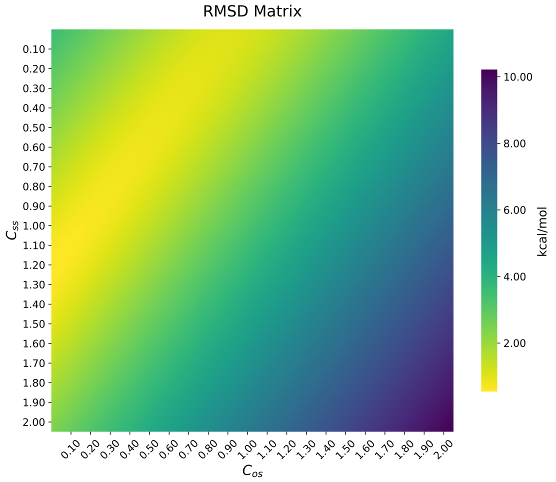

(c)

Figure S13: Evaluation of MADs (S13a), Max(MADs) (S13b) and RMSDs (S13c) in grid of values C_{OS} and C_{SS}. The optimal coefficients are C_{OS}=0.00 and C_{SS}=1.50. The theory level used is RIJK-MP2/aug-cc-pVDZ.

6.1.3 Interaction energies

MP2 with C_{OS} y C_{SS} values

Table S59: Interaction energies for CCSD(T)/CBS, MP2, SCS-MP2, SCS(MI)-MP2, SCSN-MP2, SCS-MP2-vdW, RI-MP2, RI-SCS-MP2, RIJK-MP2, RIJK-SCS-MP2, RIJCOSX-MP2, RIJCOSX-SCS-MP2, SCS-MP2^{BWI-DZ}, RI-SCS-MP2^{BWI-DZ}, RIJK-SCS-MP2^{BWI-DZ}, RIJCOSX-SCS-MP2^{BWI-DZ} and SCS-MP2-hal^{G-XZ}. Data: 66 interaction energies. MP2: C_{OS} = C_{SS} =1.00; SCS-MP2: C_{OS} =1.20, C_{SS} =0.33; SCS(MI)-MP2: C_{OS} =0.40, C_{SS} =1.29; SCSN-MP2: C_{OS} =0.00, C_{SS} =1.76; SCS-MP2-vdW: C_{OS} =1.28, C_{SS} =0.50; SCS-MP2^{BWI-DZ} RI-SCS-MP2^{BWI-DZ} , RIJK-SCS-MP2^{BWI-DZ} : C_{OS} =0.00, C_{SS} =1.50; RIJCOSX-SCS-MP2^{BWI-DZ} : C_{OS} =0.00, C_{SS} =0.17. All values in kcal/mol.

Dimer	Formula	CCSD(T)/CBS	MP2	SCS-MP2	SCS(MI)-MP2	SCSN-MP2	SCS-MP2-vdW	RI-MP2	RI-SCS-MP2	RIJK-MP2	RIJK-SCS-MP2	RIJCOSX-MP2	RIJCOSX-SCS-MP2	SCS-MP2^{BWI-DZ}	RI-SCS-MP2^{BWI-DZ}	RIJK-SCS-MP2^{BWI-DZ}	RIJCOSX-SCS-MP2^{BWI-DZ}	SCS-MP2-hal^{G-XZ}
acetamide − acetamide	CH_3CONH_2	-15.60	-16.10	-14.66	-15.48	-15.85	-15.34	-16.09	-14.66	-16.09	-14.66	-16.08	-14.64	-15.11	-15.10	-15.10	-15.11	-14.56
acetamide − uracil	CH_3CONH_2	-18.44	-19.19	-17.42	-18.76	-19.39	-18.17	-19.19	-17.42	-19.19	-17.41	-19.15	-17.37	-18.52	-18.51	-18.51	-18.51	-17.58
aceticacid − aceticacid	CH_3COOH	-18.39	-17.18	-15.47	-17.11	-17.90	-16.11	-17.18	-15.47	-17.17	-15.47	-17.14	-15.44	-17.12	-17.11	-17.10	-17.10	-15.95
aceticacid − uracil	CH_3COOH	-18.73	-18.63	-16.81	-18.41	-19.18	-17.53	-18.62	-16.81	-18.62	-16.81	-18.56	-16.75	-18.33	-18.32	-18.31	-18.29	-17.19
benzene − acetamide − NH − pi	C_6H_6	-3.84	-6.40	-5.50	-5.37	-5.28	-6.09	-6.40	-5.50	-6.41	-5.51	-6.38	-5.48	-4.71	-4.72	-4.72	-4.72	-4.86
benzene − aceticacid	C_6H_6	-3.10	-7.19	-5.95	-5.53	-5.29	-6.82	-7.20	-5.95	-7.20	-5.96	-7.18	-5.94	-4.48	-4.49	-4.49	-4.51	-4.86
benzene − aceticacid − OH − π	C_6H_6	-4.12	-7.23	-6.22	-5.99	-5.85	-6.91	-7.24	-6.23	-7.25	-6.24	-7.22	-6.21	-5.20	-5.20	-5.21	-5.20	-5.43
benzene − benzene − π − π	C_6H_6	-1.76	-7.57	-5.90	-5.23	-4.85	-7.11	-7.58	-5.91	-7.59	-5.92	-7.58	-5.90	-3.74	-3.75	-3.76	-3.79	-4.33
benzene − benzene − TS	C_6H_6	-2.24	-6.60	-5.52	-4.96	-4.66	-6.33	-6.60	-5.52	-6.60	-5.52	-6.60	-5.52	-3.92	-3.93	-3.93	-3.95	-4.39
benzene − cyclopentane	C_6H_6	-2.67	-7.95	-6.43	-5.71	-5.31	-7.56	-7.95	-6.44	-7.96	-6.44	-7.93	-6.41	-4.28	-4.29	-4.29	-4.30	-4.91
benzene − ethene	C_6H_6	-0.83	-3.54	-2.60	-2.17	-1.94	-3.29	-3.55	-2.60	-3.54	-2.59	-3.54	-2.59	-1.30	-1.31	-1.30	-1.33	-1.67
benzene − ethyne − CH − π	C_6H_6	-2.49	-5.73	-4.99	-4.68	-4.51	-5.53	-5.74	-4.99	-5.75	-5.00	-5.72	-4.97	-4.02	-4.02	-4.03	-4.02	-4.28
benzene − methylamide − NH − π	C_6H_6	-2.65	-5.90	-4.89	-4.48	-4.25	-5.62	-5.90	-4.90	-5.90	-4.90	-5.88	-4.88	-3.58	-3.59	-3.59	-3.59	-3.94
benzene − methanol − OH − π	C_6H_6	-3.60	-6.72	-5.70	-5.37	-5.18	-6.41	-6.72	-5.70	-6.73	-5.71	-6.69	-5.66	-4.52	-4.52	-4.53	-4.51	-4.81
benzene − neopentane	C_6H_6	-2.18	-6.62	-5.46	-4.78	-4.41	-6.35	-6.62	-5.46	-6.64	-5.47	-6.60	-5.44	-3.61	-3.62	-3.63	-3.62	-4.18
benzene − peptide − NH − π	C_6H_6	-4.46	-9.39	-7.97	-7.38	-7.04	-9.00	-9.39	-7.97	-9.41	-7.99	-9.36	-7.94	-6.10	-6.10	-6.11	-6.10	-6.61
benzene − pyridine − π − π	C_6H_6	-2.34	-8.32	-6.55	-5.87	-5.48	-7.82	-8.32	-6.56	-8.34	-6.57	-8.33	-6.56	-4.31	-4.32	-4.33	-4.36	-4.91
benzene − pyridine − TS	C_6H_6	-2.70	-6.99	-5.90	-5.38	-5.10	-6.70	-6.99	-5.90	-7.00	-5.91	-6.98	-5.89	-4.36	-4.37	-4.38	-4.38	-4.80
benzene − uracil − π − π	C_6H_6	-4.28	-11.71	-9.44	-8.64	-8.18	-11.05	-11.72	-9.44	-11.73	-9.45	-11.70	-9.43	-6.69	-6.70	-6.71	-6.74	-7.40
benzene − water − OH − π	C_6H_6	-2.95	-4.79	-4.18	-4.03	-3.94	-4.59	-4.79	-4.18	-4.80	-4.19	-4.77	-4.16	-3.55	-3.55	-3.56	-3.54	-3.69
cyclopentane − cyclopentane	C_5H_{10}	-2.26	-5.68	-4.50	-3.88	-3.54	-5.39	-5.69	-4.50	-5.69	-4.51	-5.65	-4.47	-2.73	-2.74	-2.74	-2.73	-3.26
cyclopentane − neopentane	C_5H_{10}	-1.78	-4.95	-4.00	-3.35	-3.00	-4.75	-4.95	-4.00	-4.96	-4.00	-4.93	-3.98	-2.32	-2.32	-2.32	-2.33	-2.86
ethene − pentane	C_2H_4	-1.52	-3.43	-2.66	-2.28	-2.08	-3.23	-3.43	-2.66	-3.43	-2.65	-3.43	-2.65	-1.55	-1.56	-1.55	-1.57	-1.87
ethyne − aceticacid − OH − π	C_2H_2	-4.52	-5.48	-4.81	-5.01	-5.09	-5.18	-5.48	-4.81	-5.48	-4.81	-5.48	-4.81	-4.72	-4.72	-4.72	-4.73	-4.60
ethyne − ethyne − TS	C_2H_2	-1.37	-2.44	-2.18	-2.02	-1.93	-2.38	-2.44	-2.17	-2.44	-2.17	-2.44	-2.17	-1.75	-1.76	-1.75	-1.76	-1.88
ethyne − pentane	C_2H_2	-1.34	-3.19	-2.53	-2.13	-1.91	-3.04	-3.20	-2.53	-3.19	-2.52	-3.20	-2.53	-1.45	-1.45	-1.45	-1.47	-1.78
ethyne − water − CH − O	C_2H_2	-2.73	-3.63	-3.36	-3.47	-3.53	-3.50	-3.64	-3.36	-3.63	-3.36	-3.63	-3.36	-3.38	-3.38	-3.38	-3.38	-3.30
methylamide − methylamide	CH_3NH_2	-3.75	-5.17	-4.43	-4.38	-4.34	-4.90	-5.17	-4.43	-5.16	-4.42	-5.15	-4.41	-3.88	-3.89	-3.88	-3.89	-3.96
methylamide − methanol	CH_3NH_2	-2.78	-3.79	-3.30	-3.26	-3.23	-3.61	-3.79	-3.30	-3.79	-3.30	-3.77	-3.29	-2.93	-2.93	-2.93	-2.93	-2.98
methylamide − peptide	CH_3NH_2	-4.89	-6.93	-5.99	-5.98	-5.95	-6.58	-6.94	-6.00	-6.93	-5.99	-6.93	-5.99	-5.38	-5.39	-5.38	-5.40	-5.44
methylamide − pyridine	CH_3NH_2	-3.36	-6.01	-4.97	-4.70	-4.54	-5.68	-6.01	-4.97	-6.01	-4.97	-6.00	-4.96	-3.87	-3.87	-3.87	-3.88	-4.12
methylamide − water	CH_3NH_2	-6.92	-8.09	-7.34	-7.50	-7.56	-7.76	-8.09	-7.34	-8.08	-7.34	-8.05	-7.31	-7.13	-7.13	-7.13	-7.11	-7.05
methanol − methylamide	CH_3OH	-7.09	-8.36	-7.48	-7.64	-7.71	-7.98	-8.36	-7.48	-8.36	-7.47	-8.34	-7.46	-7.20	-7.20	-7.20	-7.20	-7.11
methanol − methanol	CH_3OH	-5.45	-6.36	-5.75	-5.91	-5.98	-6.08	-6.36	-5.75	-6.35	-5.73	-6.34	-5.72	-5.64	-5.63	-5.62	-5.63	-5.54
methanol − peptide	CH_3OH	-7.79	-8.89	-8.02	-8.36	-8.51	-8.47	-8.89	-8.02	-8.89	-8.01	-8.89	-8.01	-8.03	-8.03	-8.03	-8.05	-7.81
methanol − pyridine	CH_3OH	-6.93	-8.31	-7.45	-7.51	-7.52	-7.97	-8.31	-7.45	-8.31	-7.46	-8.30	-7.44	-7.01	-7.00	-7.00	-7.01	-7.00
methanol − water	CH_3OH	-4.79	-5.35	-4.89	-5.12	-5.22	-5.12	-5.35	-4.89	-5.35	-4.89	-5.33	-4.88	-4.98	-4.98	-4.97	-4.96	-4.83
neopentane − neopentane	C_5H_{12}	-1.31	-4.03	-3.36	-2.70	-2.35	-3.94	-4.03	-3.36	-4.02	-3.35	-4.02	-3.35	-1.84	-1.85	-1.83	-1.85	-2.38
neopentane − pentane	C_5H_{12}	-1.97	-5.25	-4.24	-3.56	-3.19	-5.04	-5.25	-4.24	-5.25	-4.24	-5.22	-4.21	-2.48	-2.49	-2.48	-2.49	-3.04
pentane − acetamide	C_5H_{12}	-2.75	-5.86	-4.65	-4.19	-3.93	-5.51	-5.86	-4.65	-5.86	-4.65	-5.87	-4.66	-3.12	-3.13	-3.13	-3.16	-3.53
pentane − aceticacid	C_5H_{12}	-2.24	-5.31	-4.26	-3.77	-3.50	-5.03	-5.31	-4.26	-5.31	-4.26	-5.24	-4.19	-2.79	-2.80	-2.80	-2.75	-3.21
pentane − pentane	C_5H_{12}	-2.87	-7.01	-5.52	-4.76	-4.34	-6.63	-7.01	-5.52	-7.01	-5.52	-7.01	-5.52	-3.33	-3.34	-3.33	-3.37	-3.97
peptide − ethene	$C_6H_7NO_2$	-2.51	-4.46	-3.65	-3.49	-3.39	-4.19	-4.46	-3.65	-4.46	-3.65	-4.45	-3.63	-2.87	-2.88	-2.87	-2.88	-3.03
peptide − methylamide	$C_6H_7NO_2$	-6.89	-9.27	-8.32	-8.15	-8.04	-8.95	-9.27	-8.32	-9.26	-8.32	-9.25	-8.31	-7.44	-7.44	-7.44	-7.45	-7.62
peptide − methanol	$C_6H_7NO_2$	-5.78	-7.65	-6.93	-6.84	-6.78	-7.40	-7.65	-6.93	-7.65	-6.93	-7.65	-6.92	-6.32	-6.32	-6.32	-6.33	-6.43
peptide − pentane	$C_6H_7NO_2$	-3.32	-7.51	-6.00	-5.34	-4.97	-7.10	-7.52	-6.00	-7.52	-6.00	-7.48	-5.96	-3.96	-3.96	-3.97	-3.96	-4.53
peptide − peptide	$C_6H_7NO_2$	-8.03	-10.19	-9.06	-9.15	-9.18	-9.73	-10.19	-9.06	-10.19	-9.06	-10.17	-9.04	-8.50	-8.51	-8.51	-8.51	-8.48
peptide − water	$C_6H_7NO_2$	-4.89	-5.88	-5.37	-5.48	-5.53	-5.65	-5.88	-5.37	-5.88	-5.37	-5.85	-5.34	-5.24	-5.24	-5.24	-5.22	-5.18
pyridine − ethene	C_5H_5N	-1.23	-4.09	-3.07	-2.64	-2.39	-3.81	-4.09	-3.08	-4.09	-3.07	-4.08	-3.07	-1.72	-1.72	-1.72	-1.75	-2.10
pyridine − ethyne	C_5H_5N	-3.72	-5.49	-4.97	-4.96	-4.94	-5.29	-5.49	-4.97	-5.48	-4.97	-5.48	-4.97	-4.63	-4.63	-4.63	-4.64	-4.67
pyridine − pyridine − CH − N	C_5H_5N	-3.69	-5.93	-5.18	-4.95	-4.81	-5.71	-5.93	-5.18	-5.93	-5.18	-5.93	-5.18	-4.32	-4.32	-4.32	-4.34	-4.54
pyridine − pyridine − π − π	C_5H_5N	-2.77	-8.82	-7.00	-6.29	-5.88	-8.31	-8.83	-7.00	-8.83	-7.01	-8.82	-6.99	-4.68	-4.69	-4.69	-4.73	-5.30
pyridine − pyridine − TS	C_5H_5N	-2.89	-6.78	-5.66	-5.20	-4.95	-6.47	-6.79	-5.67	-6.79	-5.67	-6.78	-5.65	-4.20	-4.21	-4.21	-4.22	-4.60
pyridine − uracil − π − π	C_5H_5N	-5.41	-12.46	-10.17	-9.48	-9.08	-11.76	-12.47	-10.18	-12.48	-10.18	-12.45	-10.15	-7.59	-7.60	-7.61	-7.63	-8.22
uracil − cyclopentane	$C_4H_4N_2O_2$	-3.08	-8.29	-6.67	-5.85	-5.39	-7.88	-8.30	-6.67	-8.31	-6.68	-8.23	-6.60	-4.29	-4.30	-4.30	-4.26	-4.99
uracil − ethene	$C_4H_4N_2O_2$	-2.68	-5.80	-4.66	-4.33	-4.14	-5.45	-5.80	-4.66	-5.80	-4.66	-5.78	-4.64	-3.40	-3.41	-3.40	-3.42	-3.70
uracil − ethyne	$C_4H_4N_2O_2$	-3.12	-5.68	-4.64	-4.44	-4.32	-5.34	-5.69	-4.64	-5.68	-4.64	-5.69	-4.64	-3.66	-3.67	-3.67	-3.70	-3.86
uracil − neopentane	$C_4H_4N_2O_2$	-2.87	-7.13	-5.83	-5.14	-4.77	-6.81	-7.13	-5.83	-7.14	-5.84	-7.13	-5.83	-3.88	-3.89	-3.89	-3.92	-4.46
uracil − pentane	$C_4H_4N_2O_2$	-3.66	-9.25	-7.39	-6.57	-6.11	-8.75	-9.26	-7.39	-9.26	-7.40	-9.22	-7.36	-4.86	-4.87	-4.88	-4.88	-5.57
uracil − uracil − BP	$C_4H_4N_2O_2$	-16.29	-17.92	-15.97	-17.36	-18.03	-16.82	-17.92	-15.97	-17.92	-15.97	-17.86	-15.90	-17.06	-17.05	-17.05	-17.02	-16.08
uracil − Uracil − π − π	$C_4H_4N_2O_2$	-8.12	-15.18	-12.45	-12.04	-11.77	-14.24	-15.19	-12.45	-15.19	-12.46	-15.16	-12.42	-10.06	-10.08	-10.08	-10.11	-10.50
water − methylamide	H_2O	-6.60	-7.51	-6.87	-7.05	-7.12	-7.22	-7.51	-6.87	-7.51	-6.87	-7.47	-6.84	-6.76	-6.76	-6.76	-6.74	-6.65
water − methanol	H_2O	-5.35	-6.13	-5.61	-5.77	-5.83	-5.89	-6.13	-5.61	-6.14	-5.61	-6.11	-5.58	-5.54	-5.54	-5.54	-5.52	-5.45
water − peptide	H_2O	-7.80	-8.49	-7.72	-8.17	-8.38	-8.09	-8.49	-7.72	-8.49	-7.72	-8.47	-7.70	-7.98	-7.98	-7.98	-7.97	-7.68
water − pyridine	H_2O	-6.51	-7.64	-6.99	-7.02	-7.02	-7.39	-7.64	-6.99	-7.64	-6.99	-7.62	-6.97	-6.63	-6.62	-6.62	-6.62	-6.64
water − water	H_2O	-4.75	-5.25	-4.86	-5.06	-5.16	-5.05	-5.25	-4.86	-5.25	-4.86	-5.22	-4.84	-4.95	-4.95	-4.95	-4.93	-4.82

DFT

Table S60: Interaction energies for CCSD(T)/CBS, B97M-V, ωB97X-V, ωB97M-V, ωB97X-D3, B2PLYP-D3BJ, DSD-BLYP-D3BJ, ωB97X-D4 and B2PLYP-D4. Data: 66 interaction energies. All values in kcal/mol.

Dimer	Formula	CCSD(T)/CBS	B97M-V	ωB97X-V	ωB97M-V	ωB97X-D3	B2PLYP-D3BJ	DSD-BLYP-D3BJ	ωB97X-D4	B2PLYP-D4
$acetamide - acetamide$	$CH_3CONH_2-CH_3CONH_2$	-15.60	-15.40	-15.82	-15.96	-16.29	-16.25	-16.07	-16.93	-16.32
$acetamide - uracil$	$CH_3CONH_2-C_4H_4N_2O_2$	-18.44	-18.06	-18.57	-18.74	-19.07	-19.19	-19.03	-19.54	-19.29
$aceticacid - aceticacid$	$CH_3COOH-CH_3COOH$	-18.39	-16.97	-17.80	-17.83	-18.28	-17.81	-17.53	-18.85	-17.88
$aceticacid - uracil$	$CH_3COOH-C_4H_4N_2O_2$	-18.73	-17.80	-18.43	-18.57	-18.95	-18.87	-18.66	-19.42	-18.95
$benzene - acetamide\_NH - \pi$	$C_6H_6-CH_3CONH_2$	-3.84	-4.50	-4.78	-4.88	-5.00	-5.08	-5.31	-5.15	-5.05
$benzene - aceticacid$	$C_6H_6-CH_3COOH$	-3.10	-4.36	-4.67	-4.86	-4.81	-4.95	-5.32	-4.79	-4.89
$benzene - aceticacid\_OH - \pi$	$C_6H_6-CH_3COOH$	-4.12	-4.82	-5.26	-5.37	-5.27	-5.44	-5.78	-5.22	-5.38
$benzene - benzene\_\pi - \pi$	$C_6H_6-C_6H_6$	-1.76	-3.58	-3.80	-4.24	-4.35	-4.30	-4.74	-4.00	-4.44
$benzene - benzene\_TS$	$C_6H_6-C_6H_6$	-2.24	-3.32	-3.60	-3.80	-3.88	-4.21	-4.61	-3.91	-4.19
$benzene - cyclopentane$	$C_6H_6-C_5H_{10}$	-2.67	-4.39	-4.65	-4.94	-5.11	-5.15	-5.54	-4.98	-5.11
$benzene - ethene$	$C_6H_6-C_2H_4$	-0.83	-1.83	-1.90	-2.23	-2.37	-1.98	-2.16	-2.42	-2.06
$benzene - ethyne\_CH - \pi$	$C_6H_6-C_2H_2$	-2.49	-3.25	-3.53	-3.62	-3.55	-3.97	-4.33	-3.78	-3.86
$benzene - methylamine\_NH - \pi$	$C_6H_6-CH_3NH_2$	-2.65	-3.55	-3.78	-3.95	-4.12	-4.11	-4.39	-4.10	-4.04
$benzene - methanol\_OH - \pi$	$C_6H_6-CH_3OH$	-3.60	-4.43	-4.73	-4.90	-4.92	-4.99	-5.28	-4.91	-4.89
$benzene - neopentane$	$C_6H_6-C_5H_{12}$	-2.18	-3.62	-3.88	-4.03	-4.33	-4.23	-4.60	-4.25	-4.15
$benzene - peptide\_NH - \pi$	$C_6H_6-C_6H_7NO_2$	-4.46	-5.73	-6.23	-6.48	-6.68	-6.73	-7.15	-6.50	-6.64
$benzene - pyridine\_\pi - \pi$	$C_6H_6-C_5H_5N$	-2.34	-4.08	-4.35	-4.82	-4.84	-4.95	-5.41	-4.49	-5.09
$benzene - pyridine\_TS$	$C_6H_6-C_5H_5N$	-2.70	-3.67	-4.04	-4.23	-4.29	-4.67	-5.06	-4.35	-4.61
$benzene - uracil\_\pi - \pi$	$C_6H_6-C_4H_4N_2O_2$	-4.28	-6.38	-6.69	-7.21	-6.92	-7.45	-8.13	-6.48	-7.62
$benzene - water\_OH - \pi$	$C_6H_6-H_2O$	-2.95	-3.46	-3.73	-3.76	-3.90	-3.78	-3.98	-3.89	-3.61
$cyclopentane - cyclopentane$	$C_5H_{10}-C_5H_{10}$	-2.26	-3.72	-3.97	-4.08	-4.24	-4.29	-4.36	-4.54	-4.22
$cyclopentane - neopentane$	$C_5H_{10}-C_5H_{12}$	-1.78	-3.17	-3.41	-3.47	-3.81	-3.71	-3.78	-4.05	-3.66
$ethene - pentane$	$C_2H_4-C_5H_{12}$	-1.52	-2.41	-2.52	-2.55	-2.72	-2.61	-2.65	-3.16	-2.52
$ethyne - aceticacid\_OH - \pi$	$C_2H_2-CH_3COOH$	-4.52	-5.09	-5.21	-5.34	-5.28	-5.20	-5.22	-5.83	-5.16
$ethyne - ethyne\_TS$	$C_2H_2-C_2H_2$	-1.37	-1.78	-1.83	-1.89	-1.85	-2.01	-2.07	-2.17	-1.97
$ethyne - pentane$	$C_2H_2-C_5H_{12}$	-1.34	-2.04	-2.17	-2.29	-2.41	-2.21	-2.31	-2.69	-2.10
$ethyne - water\_CH - O$	$C_2H_2-H_2O$	-2.73	-3.28	-3.35	-3.40	-3.30	-3.46	-3.50	-3.76	-3.45
$methylamine - methylamine$	$CH_3NH_2-CH_3NH_2$	-3.75	-4.31	-4.54	-4.61	-4.65	-4.67	-4.70	-5.22	-4.67
$methylamine - methanol$	$CH_3NH_2-CH_3OH$	-2.78	-3.19	-3.37	-3.36	-3.48	-3.51	-3.52	-3.80	-3.51
$methylamine - peptide$	$CH_3NH_2-C_6H_7NO_2$	-4.89	-5.82	-5.99	-6.15	-6.06	-6.08	-6.20	-6.62	-6.06
$methylamine - pyridine$	$CH_3NH_2-C_5H_5N$	-3.36	-4.14	-4.31	-4.49	-4.54	-4.66	-4.84	-4.87	-4.66
$methylamine - water$	$CH_3NH_2-H_2O$	-6.92	-7.24	-7.79	-7.75	-7.95	-7.99	-7.93	-8.40	-7.91
$methanol - methylamine$	$CH_3OH-CH_3NH_2$	-7.09	-7.45	-7.91	-7.90	-8.25	-8.15	-8.07	-8.51	-8.16
$methanol - methanol$	CH_3OH-CH_3OH	-5.45	-5.68	-5.93	-5.97	-5.97	-6.15	-6.15	-6.41	-6.15
$methanol - peptide$	CH_3OH-CH_3OH	-7.79	-8.31	-8.51	-8.58	-8.61	-8.61	-8.61	-9.13	-8.61
$methanol - pyridine$	$CH_3OH-C_5H_5N$	-6.93	-7.06	-7.52	-7.53	-7.85	-8.03	-7.96	-8.07	-8.06
$methanol - water$	CH_3OH-H_2O	-4.79	-5.11	-5.17	-5.21	-5.19	-5.25	-5.25	-5.70	-5.26
$neopentane - neopentane$	$C_5H_{12}-C_5H_{12}$	-1.31	-2.46	-2.68	-2.60	-3.04	-2.91	-3.01	-3.21	-2.85
$neopentane - pentane$	$C_5H_{12}-C_5H_{12}$	-1.97	-3.56	-3.71	-3.78	-3.98	-3.91	-4.01	-4.30	-3.77
$pentane - acetamide$	$C_5H_{12}-CH_3CONH_2$	-2.75	-4.27	-4.34	-4.40	-4.58	-4.46	-4.59	-4.92	-4.37
$pentane - aceticacid$	$C_5H_{12}-CH_3COOH$	-2.24	-3.67	-3.82	-3.81	-3.93	-3.96	-4.11	-4.12	-3.82
$pentane - pentane$	$C_5H_{12}-C_5H_{12}$	-2.87	-5.10	-5.26	-5.48	-5.57	-5.43	-5.48	-6.00	-5.21
$peptide - ethene$	$C_6H_7NO_2-CH_2H_4$	-2.51	-3.27	-3.44	-3.50	-3.57	-3.59	-3.69	-3.95	-3.54
$peptide - methylamine$	$C_6H_7NO_2-CH_3NH_2$	-6.89	-7.46	-8.05	-8.15	-8.42	-8.39	-8.45	-8.64	-8.39
$peptide - methanol$	$C_6H_7NO_2-CH_3OH$	-5.78	-6.20	-6.65	-6.77	-6.78	-6.89	-7.01	-7.21	-6.89
$peptide - pentane$	$C_6H_7NO_2-C_5H_{12}$	-3.32	-5.27	-5.44	-5.56	-5.57	-5.63	-5.81	-5.98	-5.53
$peptide - peptide$	$C_6H_7NO_2-C_6H_7NO_2$	-8.03	-8.79	-9.04	-9.15	-9.45	-9.42	-9.46	-9.65	-9.38
$peptide - water$	$C_6H_7NO_2-H_2O$	-4.89	-5.27	-5.42	-5.44	-5.45	-5.55	-5.60	-5.93	-5.51
$pyridine - ethene$	$C_5H_5N-C_2H_4$	-1.23	-2.19	-2.25	-2.58	-2.70	-2.46	-2.65	-2.80	-2.52
$pyridine - ethyne$	$C_5H_5N-C_2H_2$	-3.72	-4.47	-4.65	-4.75	-4.85	-5.13	-5.13	-5.14	-5.13
$pyridine - pyridine\_CH - N$	$C_5H_5N-C_5H_5N$	-3.69	-4.01	-4.46	-4.49	-4.49	-5.01	-5.14	-5.20	-5.07
$pyridine - pyridine\_\pi - \pi$	$C_5H_5N-C_5H_5N$	-2.77	-4.39	-4.74	-5.22	-5.24	-5.42	-5.87	-4.89	-5.55
$pyridine - pyridine\_TS$	$C_5H_5N-C_5H_5N$	-2.89	-3.74	-4.10	-4.31	-4.34	-4.70	-5.03	-4.51	-4.66
$pyridine - uracil\_\pi - \pi$	$C_5H_5N-C_4H_4N_2O_2$	-5.41	-7.35	-7.64	-8.09	-7.85	-8.43	-9.05	-7.52	-8.63
$uracil - cyclopentane$	$C_4H_4N_2O_2-C_5H_{10}$	-3.08	-4.93	-5.13	-5.27	-5.39	-5.60	-5.95	-5.27	-5.48
$uracil - ethene$	$C_4H_4N_2O_2-C_2H_4$	-2.68	-3.69	-3.85	-4.10	-4.15	-4.10	-4.34	-4.23	-4.13
$uracil - ethyne$	$C_4H_4N_2O_2-C_2H_2$	-3.12	-3.90	-4.02	-4.33	-4.14	-4.15	-4.39	-4.45	-4.20
$uracil - neopentane$	$C_4H_4N_2O_2-C_5H_{12}$	-2.87	-4.42	-4.64	-4.74	-4.92	-4.95	-5.25	-4.83	-4.77
$uracil - pentane$	$C_4H_4N_2O_2-C_5H_{12}$	-3.66	-5.80	-5.97	-6.13	-6.29	-6.37	-6.74	-6.15	-6.25
$uracil - uracil\_BP$	$C_4H_4N_2O_2-C_4H_4N_2O_2$	-16.29	-16.40	-16.79	-17.00	-17.29	-17.65	-17.52	-17.67	-17.80
$uracil - uracil\_\pi - \pi$	$C_4H_4N_2O_2-C_4H_4N_2O_2$	-8.12	-10.31	-10.35	-10.79	-10.34	-11.11	-11.72	-9.85	-11.40
$water - methylamine$	$H_2O-CH_3NH_2$	-6.60	-6.94	-7.40	-7.36	-7.69	-7.62	-7.51	-7.99	-7.54
$water - methanol$	H_2O-CH_3OH	-5.35	-5.58	-5.88	-5.89	-5.91	-6.06	-6.05	-6.44	-6.00
$water - peptide$	$H_2O-C_6H_7NO_2$	-7.80	-8.28	-8.55	-8.56	-8.57	-8.57	-8.51	-9.25	-8.50
$water - pyridine$	$H_2O-C_5H_5N$	-6.51	-6.71	-7.19	-7.15	-7.47	-7.62	-7.53	-7.79	-7.55
$water - water$	H_2O-H_2O	-4.75	-5.09	-5.19	-5.20	-5.22	-5.25	-5.24	-5.79	-5.23

SAPT-DFT

Table S61: Interaction energies for CCSD(T)/CBS and SAPT-DFT. For SAPT-DFT method we used the B3LYP, PBE0 and ωB97X DFA's in conjunction with the aug-cc-pVDZ basis set. Data: 66 interaction energies. All values in kcal/mol.

Dimer	Formula	CCSD(T)/CBS	SAPT-B3LYP	SAPT-PBE0	SAPT-ωB97X
acetamide − acetamide	$CH_3CONH_2-CH_3CONH_2$	-15.60	-12.33	-13.05	-12.66
acetamide − uracil	$CH_3CONH_2-C_4H_4N_2O_2$	-18.44	-14.80	-15.63	-15.33
aceticacid − aceticacid	$CH_3COOH-CH_3COOH$	-18.39	-13.66	-14.72	-14.77
aceticacid − uracil	$CH_3COOH-C_4H_4N_2O_2$	-18.73	-14.46	-15.37	-15.31
benzene − acetamide_NH − π	$C_6H_6-CH_3CONH_2$	-3.84	-3.07	-3.35	-2.95
benzene − aceticacid	$C_6H_6-CH_3COOH$	-3.10	-2.50	-2.59	-2.14
benzene − aceticacid_OH − π	$C_6H_6-CH_3COOH$	-4.12	-3.08	-3.45	-3.13
benzene − benzene_π − π	$C_6H_6-C_6H_6$	-1.76	-1.72	-1.62	-0.93
benzene − benzene_TS	$C_6H_6-C_6H_6$	-2.24	-1.84	-1.93	-1.51
benzene − cyclopentane	$C_6H_6-C_5H_{10}$	-2.67	-2.21	-2.19	-1.66
benzene − ethene	$C_6H_6-C_2H_4$	-0.83	-0.78	-0.65	-0.09
benzene − ethyne_CH − π	$C_6H_6-C_2H_2$	-2.49	-2.05	-2.26	-1.99
benzene − methylamine_NH − π	$C_6H_6-CH_3NH_2$	-2.65	-2.06	-2.17	-1.78
benzene − methanol_OH − π	$C_6H_6-CH_3OH$	-3.60	-2.86	-3.10	-2.59
benzene − neopentane	$C_6H_6-C_5H_{12}$	-2.18	-1.87	-1.87	-1.37
benzene − peptide_NH − π	$C_6H_6-C_6H_7NO_2$	-4.46	-3.55	-3.80	-3.40
benzene − pyridine_π − π	$C_6H_6-C_5H_5N$	-2.34	-2.19	-2.11	-1.52
benzene − pyridine_TS	$C_6H_6-C_5H_5N$	-2.70	-2.20	-2.33	-2.01
benzene − uracil_π − π	$C_6H_6-C_4H_4N_2O_2$	-4.28	-3.40	-3.40	-2.67
benzene − water_OH − π	$C_6H_6-H_2O$	-2.95	-2.26	-2.50	-2.23
cyclopentane − cyclopentane	$C_5H_{10}-C_5H_{10}$	-2.26	-1.62	-1.59	-1.25
cyclopentane − neopentane	$C_5H_{10}-C_5H_{12}$	-1.78	-1.29	-1.27	-0.89
ethene − pentane	$C_2H_4-C_5H_{12}$	-1.52	-1.15	-1.13	-0.72
ethyne − aceticacid_OH − π	$C_2H_2-CH_3COOH$	-4.52	-3.48	-3.71	-3.51
ethyne − ethyne_TS	$C_2H_2-C_2H_2$	-1.37	-1.28	-1.33	-1.15
ethyne − pentane	$C_2H_2-C_5H_{12}$	-1.34	-1.17	-1.12	-0.74
ethyne − water_CH − O	$C_2H_2-H_2O$	-2.73	-2.42	-2.53	-2.33
methylamine − methylamine	$CH_3NH_2-CH_3NH_2$	-3.75	-2.73	-2.88	-2.63
methylamine − methanol	$CH_3NH_2-CH_3OH$	-2.78	-2.11	-2.20	-1.92
methylamine − peptide	$CH_3NH_2-C_6H_7NO_2$	-4.89	-3.70	-3.85	-3.67
methylamine − pyridine	$CH_3NH_2-C_5H_5N$	-3.36	-2.63	-2.69	-2.38
methylamine − water	$CH_3NH_2-H_2O$	-6.92	-5.42	-5.75	-5.45
methanol − methylamine	$CH_3OH-CH_3NH_2$	-7.09	-5.54	-5.93	-5.47
methanol − methanol	CH_3OH-CH_3OH	-5.45	-4.17	-4.36	-4.09
methanol − peptide	CH_3OH-CH_3OH	-7.79	-5.98	-6.28	-6.06
methanol − pyridine	$CH_3OH-C_5H_5N$	-6.93	-5.59	-5.81	-5.54
methanol − water	CH_3OH-H_2O	-4.79	-3.85	-4.02	-3.76
neopentane − neopentane	$C_5H_{12}-C_5H_{12}$	-1.31	-1.04	-0.99	-0.67
neopentane − pentane	$C_5H_{12}-C_5H_{12}$	-1.97	-1.40	-1.37	-1.06
pentane − acetamide	$C_5H_{12}-CH_3CONH_2$	-2.75	-2.06	-2.07	-1.56
pentane − aceticacid	$C_5H_{12}-CH_3COOH$	-2.24	-1.58	-1.59	-1.25
pentane − pentane	$C_5H_{12}-C_5H_{12}$	-2.87	-1.97	-1.90	-1.61
peptide − ethene	$C_6H_7NO_2-CH_2H_4$	-2.51	-1.97	-2.03	-1.68
peptide − methylamine	$C_6H_7NO_2-CH_3NH_2$	-6.89	-5.27	-5.63	-5.34
peptide − methanol	$C_6H_7NO_2-CH_3OH$	-5.78	-4.54	-4.73	-4.45
peptide − pentane	$C_6H_7NO_2-C_5H_{12}$	-3.32	-2.20	-2.21	-1.92
peptide − peptide	$C_6H_7NO_2-C_6H_7NO_2$	-8.03	-6.44	-6.72	-6.25
peptide − water	$C_6H_7NO_2-H_2O$	-4.89	-4.06	-4.22	-4.01
pyridine − ethene	$C_5H_5N-C_2H_4$	-1.23	-1.13	-1.01	-0.48
pyridine − ethyne	$C_5H_5N-C_2H_2$	-3.72	-3.13	-3.25	-3.17
pyridine − pyridine_CH − N	$C_5H_5N-C_5H_5N$	-3.69	-2.81	-2.93	-2.88
pyridine − pyridine_π − π	$C_5H_5N-C_5H_5N$	-2.77	-2.56	-2.50	-2.02
pyridine − pyridine_TS	$C_5H_5N-C_5H_5N$	-2.89	-2.28	-2.40	-2.15
pyridine − uracil_π − π	$C_5H_5N-C_4H_4N_2O_2$	-5.41	-4.57	-4.52	-4.00
uracil − cyclopentane	$C_4H_4N_2O_2-C_5H_{10}$	-3.08	-2.17	-2.16	-1.73
uracil − ethene	$C_4H_4N_2O_2-C_2H_4$	-2.68	-2.17	-2.11	-1.59
uracil − ethyne	$C_4H_4N_2O_2-C_2H_2$	-3.12	-2.61	-2.54	-2.12
uracil − neopentane	$C_4H_4N_2O_2-C_5H_{12}$	-2.87	-2.22	-2.19	-1.72
uracil − pentane	$C_4H_4N_2O_2-C_5H_{12}$	-3.66	-2.53	-2.55	-2.11
uracil − uracil_BP	$C_4H_4N_2O_2-C_4H_4N_2O_2$	-16.29	-12.69	-13.45	-13.26
uracil − uracil_π − π	$C_4H_4N_2O_2-C_4H_4N_2O_2$	-8.12	-6.81	-6.98	-6.19
water − methylamine	$H_2O-CH_3NH_2$	-6.60	-5.18	-5.51	-5.24
water − methanol	H_2O-CH_3OH	-5.35	-4.10	-4.28	-4.07
water − peptide	$H_2O-C_6H_7NO_2$	-7.80	-6.18	-6.43	-6.06
water − pyridine	$H_2O-C_5H_5N$	-6.51	-5.26	-5.42	-5.29
water − water	H_2O-H_2O	-4.75	-3.83	-3.98	-3.78

6.1.4 Absolute deviations of molecular systems.

MP2 with C_{OS} y C_{SS} values

Table S62: Absolute deviations for MP2, SCS-MP2, SCS(MI)-MP2, SCSN-MP2, SCS-MP2-vdW, RI-MP2, RI-SCS-MP2, RIJK-MP2, RIJK-SCS-MP2, RIJCOSX-MP2, RIJCOSX-SCS-MP2, SCS-MP2^{BWI-DZ}, RI-SCS-MP2^{BWI-DZ}, RIJK-SCS-MP2^{BWI-DZ}, RIJCOSX-SCS-MP2^{BWI-DZ} and SCS-MP2-hal^{G-XZ}. Data: 66 interaction energies. MP2: C_{OS} = C_{SS} =1.00; SCS-MP2: C_{OS} =1.20, C_{SS} =0.33; SCS(MI)-MP2: C_{OS} =0.40, C_{SS} =1.29; SCSN-MP2: C_{OS} =0.00, C_{SS} =1.76; SCS-MP2-vdW: C_{OS} =1.28, C_{SS} =0.50; SCS-MP2^{BWI-DZ} RI-SCS-MP2^{BWI-DZ} , RIJK-SCS-MP2^{BWI-DZ} : C_{OS} =0.00, C_{SS} =1.50; RIJCOSX-SCS-MP2^{BWI-DZ} : C_{OS} =0.00, C_{SS} =0.17. All values in kcal/mol.

Dimer	Formula	MP2	SCS-MP2	SCS(MI)-MP2	SCSN-MP2	SCS-MP2-vdW	RI-MP2	RI-SCS-MP2	RIJK-MP2	RIJK-SCS-MP2	RIJCOSX-MP2	RIJCOSX-SCS-MP2	SCS-MP2^{BWI-DZ}	RI-SCS-MP2^{BWI-DZ}	RIJK-SCS-MP2^{BWI-DZ}	RIJCOSX-SCS-MP2^{BWI-DZ}	SCS-MP2-hal^{G-XZ}
acetamide − acetamide	CH_3CONH_2	0.50	0.94	0.12	0.25	0.26	0.49	0.94	0.49	0.94	0.48	0.96	0.49	0.50	0.50	0.49	1.04
acetamide − uracil	CH_3CONH_2	0.75	1.02	0.32	0.95	0.27	0.75	1.02	0.75	1.03	0.71	1.07	0.08	0.07	0.07	0.07	0.86
aceticacid − aceticacid	CH_3COOH	1.21	2.92	1.28	0.49	2.28	1.21	2.92	1.22	2.92	1.25	2.95	1.27	1.28	1.29	1.29	2.44
aceticacid − Uracil	CH_3COOH	0.10	1.92	0.32	0.45	1.20	0.11	1.92	0.11	1.92	0.17	1.98	0.40	0.41	0.42	0.44	1.54
benzene − acetamide − NH − π	C_6H_6	2.56	1.66	1.53	1.44	2.25	2.56	1.66	2.57	1.67	2.54	1.64	0.87	0.88	0.88	0.88	1.02
benzene − aceticacid	C_6H_6	4.09	2.85	2.43	2.19	3.72	4.10	2.85	4.10	2.86	4.08	2.84	1.38	1.39	1.39	1.41	1.76
benzene − aceticacid − OH − π	C_6H_6	3.11	2.10	1.87	1.73	2.79	3.12	2.11	3.13	2.12	3.10	2.09	1.08	1.08	1.09	1.08	1.31
benzene − benzene − π − π	C_6H_6	5.81	4.14	3.47	3.09	5.35	5.82	4.15	5.83	4.16	5.82	4.14	1.98	1.99	2.00	2.03	2.57
benzene − benzene − TS	C_6H_6	4.36	3.28	2.72	2.42	4.09	4.36	3.28	4.36	3.28	4.36	3.28	1.68	1.69	1.69	1.71	2.15
benzene − cyclopentane	C_6H_6	5.28	3.76	3.04	2.64	4.89	5.28	3.77	5.29	3.77	5.26	3.74	1.61	1.62	1.62	1.63	2.24
benzene − ethene	C_6H_6	2.71	1.77	1.34	1.11	2.46	2.72	1.77	2.71	1.76	2.71	1.76	0.47	0.48	0.47	0.50	0.84
benzene − ethyne − CH − π	C_6H_6	3.24	2.50	2.19	2.02	3.04	3.25	2.50	3.26	2.51	3.23	2.48	1.53	1.53	1.54	1.53	1.79
benzene − methylamine − NH − π	C_6H_6	3.25	2.24	1.83	1.60	2.97	3.25	2.25	3.25	2.25	3.23	2.23	0.93	0.94	0.94	0.94	1.29
benzene − methanol − OH − π	C_6H_6	3.12	2.10	1.77	1.58	2.81	3.12	2.10	3.13	2.11	3.09	2.06	0.92	0.92	0.93	0.91	1.21
benzene − neopentane	C_6H_6	4.44	3.28	2.60	2.23	4.17	4.44	3.28	4.46	3.29	4.42	3.26	1.43	1.44	1.45	1.44	2.00
benzene − peptide − NH − π	C_6H_6	4.93	3.51	2.92	2.58	4.54	4.93	3.51	4.95	3.53	4.90	3.48	1.64	1.64	1.65	1.64	2.15
benzene − pyridine − π − π	C_6H_6	5.98	4.21	3.53	3.14	5.48	5.98	4.22	6.00	4.23	5.99	4.22	1.97	1.98	1.99	2.02	2.57
benzene − pyridine − TS	C_6H_6	4.29	3.20	2.68	2.40	4.00	4.29	3.20	4.30	3.21	4.28	3.19	1.66	1.67	1.68	1.68	2.10
benzene − uracil − π − π	C_6H_6	7.43	5.16	4.36	3.90	6.77	7.44	5.16	7.45	5.17	7.42	5.15	2.41	2.42	2.43	2.46	3.12
benzene − water − OH − π	C_6H_6	1.84	1.23	1.08	0.99	1.64	1.84	1.23	1.85	1.24	1.82	1.21	0.60	0.60	0.61	0.59	0.74
cyclopentane − cyclopentane	C_5H_{10}	3.42	2.24	1.62	1.28	3.13	3.43	2.24	3.43	2.25	3.39	2.21	0.47	0.48	0.48	0.47	1.00
cyclopentane − neopentane	C_5H_{10}	3.17	2.22	1.57	1.22	2.97	3.17	2.22	3.18	2.22	3.15	2.20	0.54	0.54	0.54	0.55	1.08
ethene − pentane	C_2H_4	1.91	1.14	0.76	0.56	1.71	1.91	1.14	1.91	1.13	1.91	1.13	0.03	0.04	0.03	0.05	0.35
ethyne − aceticacid − OH − π	C_2H_2	0.96	0.29	0.49	0.57	0.66	0.96	0.29	0.96	0.29	0.96	0.29	0.20	0.20	0.20	0.21	0.08
ethyne − ethyne − TS	C_2H_2	1.07	0.81	0.65	0.56	1.01	1.07	0.80	1.07	0.80	1.07	0.80	0.38	0.39	0.38	0.39	0.51
ethyne − pentane	C_2H_2	1.85	1.19	0.79	0.57	1.70	1.86	1.19	1.85	1.18	1.86	1.19	0.11	0.11	0.11	0.13	0.44
ethyne − water − CH − O	C_2H_2	0.90	0.63	0.74	0.80	0.77	0.91	0.63	0.90	0.63	0.90	0.63	0.65	0.65	0.65	0.65	0.57
methylamine − methylamine	CH_3NH_2	1.42	0.68	0.63	0.59	1.15	1.42	0.68	1.41	0.67	1.40	0.66	0.13	0.14	0.13	0.14	0.21
methylamine − methanol	CH_3NH_2	1.01	0.52	0.48	0.45	0.83	1.01	0.52	1.01	0.52	0.99	0.51	0.15	0.15	0.15	0.15	0.20
methylamine − peptide	CH_3NH_2	2.04	1.10	1.09	1.06	1.69	2.05	1.11	2.04	1.10	2.04	1.10	0.49	0.50	0.49	0.51	0.55
methylamine − pyridine	CH_3NH_2	2.65	1.61	1.34	1.18	2.32	2.65	1.61	2.65	1.61	2.64	1.60	0.51	0.51	0.51	0.52	0.76
methylamine − water	CH_3NH_2	1.17	0.42	0.58	0.64	0.84	1.17	0.42	1.16	0.42	1.13	0.39	0.21	0.21	0.21	0.19	0.13
methanol − methylamine	CH_3OH	1.27	0.39	0.55	0.62	0.89	1.27	0.39	1.27	0.38	1.25	0.37	0.11	0.11	0.11	0.11	0.02
methanol − methanol	CH_3OH	0.91	0.30	0.46	0.53	0.63	0.91	0.30	0.90	0.28	0.89	0.27	0.19	0.18	0.17	0.18	0.09
methanol − peptide	CH_3OH	1.10	0.23	0.57	0.72	0.68	1.10	0.23	1.10	0.22	1.10	0.22	0.24	0.24	0.24	0.26	0.02
methanol − pyridine	CH_3OH	1.38	0.52	0.58	0.59	1.04	1.38	0.52	1.38	0.53	1.37	0.51	0.08	0.07	0.07	0.08	0.07
methanol − water	CH_3OH	0.56	0.10	0.33	0.43	0.33	0.56	0.10	0.56	0.10	0.54	0.09	0.19	0.19	0.18	0.17	0.04
neopentane − neopentane	C_5H_{12}	2.72	2.05	1.39	1.04	2.63	2.72	2.05	2.71	2.04	2.71	2.04	0.53	0.54	0.52	0.54	1.07
neopentane − pentane	C_5H_{12}	3.28	2.27	1.59	1.22	3.07	3.28	2.27	3.28	2.27	3.25	2.24	0.51	0.52	0.51	0.52	1.07
pentane − acetamide	C_5H_{12}	3.11	1.90	1.44	1.18	2.76	3.11	1.90	3.11	1.90	3.12	1.91	0.37	0.38	0.38	0.41	0.78
pentane − aceticacid	C_5H_{12}	3.07	2.02	1.53	1.26	2.79	3.07	2.02	3.07	2.02	3.00	1.95	0.55	0.56	0.56	0.51	0.97
pentane − pentane	C_5H_{12}	4.14	2.65	1.89	1.47	3.76	4.14	2.65	4.14	2.65	4.14	2.65	0.46	0.47	0.46	0.50	1.10
peptide − ethene	$C_6H_7NO_2$	1.95	1.14	0.98	0.88	1.68	1.95	1.14	1.95	1.14	1.94	1.12	0.36	0.37	0.36	0.37	0.52
peptide − methylamine	$C_6H_7NO_2$	2.38	1.43	1.26	1.15	2.06	2.38	1.43	2.37	1.43	2.36	1.42	0.55	0.55	0.55	0.56	0.73
peptide − methanol	$C_6H_7NO_2$	1.87	1.15	1.06	1.00	1.62	1.87	1.15	1.87	1.15	1.87	1.14	0.54	0.54	0.54	0.55	0.65
peptide − pentane	$C_6H_7NO_2$	4.19	2.68	2.02	1.65	3.78	4.20	2.68	4.20	2.68	4.16	2.64	0.64	0.64	0.65	0.64	1.21
peptide − peptide	$C_6H_7NO_2$	2.16	1.03	1.12	1.15	1.70	2.16	1.03	2.16	1.03	2.14	1.01	0.47	0.48	0.48	0.48	0.45
peptide − water	$C_6H_7NO_2$	0.99	0.48	0.59	0.64	0.76	0.99	0.48	0.99	0.48	0.96	0.45	0.35	0.35	0.35	0.33	0.29
pyridine − ethene	C_5H_5N	2.86	1.84	1.41	1.16	2.58	2.86	1.85	2.86	1.84	2.85	1.84	0.49	0.49	0.49	0.52	0.87
pyridine − ethyne	C_5H_5N	1.77	1.25	1.24	1.22	1.57	1.77	1.25	1.76	1.25	1.76	1.25	0.91	0.91	0.91	0.92	0.95
pyridine − pyridine − CH − N	C_5H_5N	2.24	1.49	1.26	1.12	2.02	2.24	1.49	2.24	1.49	2.24	1.49	0.63	0.63	0.63	0.65	0.85
pyridine − pyridine − π − π	C_5H_5N	6.05	4.23	3.52	3.11	5.54	6.06	4.23	6.06	4.24	6.05	4.22	1.91	1.92	1.92	1.96	2.53
pyridine − pyridine − TS	C_5H_5N	3.89	2.77	2.31	2.06	3.58	3.90	2.78	3.90	2.78	3.89	2.76	1.31	1.32	1.32	1.33	1.71
pyridine − uracil − π − π	C_5H_5N	7.05	4.76	4.07	3.67	6.35	7.06	4.77	7.07	4.77	7.04	4.74	2.18	2.19	2.20	2.22	2.81
uracil − cyclopentane	$C_4H_4N_2O_2$	5.21	3.59	2.77	2.31	4.80	5.22	3.59	5.23	3.60	5.15	3.52	1.21	1.22	1.22	1.18	1.91
uracil − ethene	$C_4H_4N_2O_2$	3.12	1.98	1.65	1.46	2.77	3.12	1.98	3.12	1.98	3.10	1.96	0.72	0.73	0.72	0.74	1.02
uracil − ethyne	$C_4H_4N_2O_2$	2.56	1.52	1.32	1.20	2.22	2.57	1.52	2.56	1.52	2.57	1.52	0.54	0.55	0.55	0.58	0.74
uracil − neopentane	$C_4H_4N_2O_2$	4.26	2.96	2.27	1.90	3.94	4.26	2.96	4.27	2.97	4.26	2.96	1.01	1.02	1.02	1.05	1.59
uracil − pentane	$C_4H_4N_2O_2$	5.59	3.73	2.91	2.45	5.09	5.60	3.73	5.60	3.74	5.56	3.70	1.20	1.21	1.22	1.22	1.91
uracil − uracil − BP	$C_4H_4N_2O_2$	1.63	0.32	1.07	1.74	0.53	1.63	0.32	1.63	0.32	1.57	0.39	0.77	0.76	0.76	0.73	0.21
uracil − uracil − π − π	$C_4H_4N_2O_2$	7.06	4.33	3.92	3.65	6.12	7.07	4.33	7.07	4.34	7.04	4.30	1.94	1.96	1.96	1.99	2.38
water − methylamine	H_2O	0.91	0.27	0.45	0.52	0.62	0.91	0.27	0.91	0.27	0.87	0.24	0.16	0.16	0.16	0.14	0.05
water − methanol	H_2O	0.78	0.26	0.42	0.48	0.54	0.78	0.26	0.79	0.26	0.76	0.23	0.19	0.19	0.19	0.17	0.10
water − Peptide	H_2O	0.69	0.08	0.37	0.58	0.29	0.69	0.08	0.69	0.08	0.67	0.10	0.18	0.18	0.18	0.17	0.12
water − Pyridine	H_2O	1.13	0.48	0.51	0.51	0.88	1.13	0.48	1.13	0.48	1.11	0.46	0.12	0.11	0.11	0.11	0.13
water − Water	H_2O	0.50	0.11	0.31	0.41	0.30	0.50	0.11	0.50	0.11	0.47	0.09	0.20	0.20	0.20	0.18	0.07

DFT

Table S63: Absolute deviations for B97M-V, ωB97X-V, ωB97M-V, ωB97X-D3, B2PLYP-D3BJ, DSD-BLYP-D3BJ, ωB97X-D4 and B2PLYP-D4. Data: 66 interaction energies. All values in kcal/mol.

Dimer	Formula	B97M-V	ωB97X-V	ωB97M-V	ωB97X-D3	B2PLYP-D3BJ	DSD-BLYP-D3BJ	ωB97X-D4	B2PLYP-D4
acetamide − acetamide	$CH_3CONH_2{-}CH_3CONH_2$	0.20	0.23	0.36	0.70	0.65	0.48	1.33	0.72
acetamide − uracil	$CH_3CONH_2{-}C_4H_4N_2O_2$	0.38	0.13	0.30	0.63	0.75	0.59	1.10	0.85
aceticacid − aceticacid	$CH_3COOH{-}CH_3COOH$	1.41	0.59	0.56	0.10	0.57	0.86	0.46	0.51
aceticacid − uracil	$CH_3COOH{-}C_4H_4N_2O_2$	0.93	0.30	0.16	0.22	0.14	0.07	0.69	0.22
benzene − acetamide_NH − π	$C_6H_6{-}CH_3CONH_2$	0.66	0.94	1.03	1.16	1.24	1.47	1.30	1.20
benzene − aceticacid	$C_6H_6{-}CH_3COOH$	1.27	1.57	1.77	1.72	1.85	2.22	1.69	1.80
benzene − aceticacid_OH − π	$C_6H_6{-}CH_3COOH$	0.70	1.14	1.25	1.15	1.32	1.66	1.10	1.26
benzene − benzene_π − π	$C_6H_6{-}C_6H_6$	1.82	2.03	2.48	2.58	2.53	2.98	2.23	2.68
benzene − benzene_TS	$C_6H_6{-}C_6H_6$	1.08	1.36	1.56	1.64	1.97	2.37	1.67	1.95
benzene − cyclopentane	$C_6H_6{-}C_5H_{10}$	1.72	1.98	2.27	2.44	2.48	2.87	2.31	2.44
benzene − ethene	$C_6H_6{-}C_2H_4$	1.01	1.07	1.40	1.55	1.15	1.33	1.60	1.23
benzene − ethyne_CH − π	$C_6H_6{-}C_2H_2$	0.76	1.04	1.13	1.06	1.48	1.83	1.29	1.36
benzene − methylamine_NH − π	$C_6H_6{-}CH_3NH_2$	0.89	1.13	1.30	1.47	1.45	1.74	1.44	1.39
benzene − methanol_OH − π	$C_6H_6{-}CH_3OH$	0.83	1.13	1.29	1.32	1.38	1.68	1.31	1.29
benzene − neopentane	$C_6H_6{-}C_5H_{12}$	1.43	1.70	1.84	2.15	2.04	2.41	2.06	1.96
benzene − peptide_NH − π	$C_6H_6{-}C_6H_7NO_2$	1.27	1.77	2.02	2.22	2.27	2.69	2.04	2.18
benzene − pyridine_π − π	$C_6H_6{-}C_5H_5N$	1.74	2.01	2.48	2.51	2.61	3.07	2.16	2.75
benzene − pyridine_TS	$C_6H_6{-}C_5H_5N$	0.97	1.34	1.52	1.59	1.96	2.36	1.64	1.90
benzene − uracil_π − π	$C_6H_6{-}C_4H_4N_2O_2$	2.10	2.41	2.93	2.64	3.17	3.84	2.20	3.33
benzene − water_OH − π	$C_6H_6{-}H_2O$	0.51	0.78	0.81	0.95	0.83	1.03	0.95	0.66
cyclopentane − cyclopentane	$C_5H_{10}{-}C_5H_{10}$	1.46	1.71	1.83	1.98	2.03	2.10	2.29	1.97
cyclopentane − neopentane	$C_5H_{10}{-}C_5H_{12}$	1.38	1.62	1.68	2.03	1.93	2.00	2.27	1.88
ethene − pentane	$C_2H_4{-}C_5H_{12}$	0.89	0.99	1.03	1.19	1.09	1.13	1.64	1.00
ethyne − aceticacid_OH − π	$C_2H_2{-}CH_3COOH$	0.58	0.69	0.83	0.77	0.68	0.70	1.32	0.65
ethyne − ethyne_TS	$C_2H_2{-}C_2H_2$	0.41	0.46	0.52	0.48	0.63	0.70	0.79	0.60
ethyne − pentane	$C_2H_2{-}C_5H_{12}$	0.70	0.83	0.95	1.07	0.86	0.97	1.35	0.76
ethyne − water_CH − O	$C_2H_2{-}H_2O$	0.55	0.62	0.66	0.57	0.73	0.77	1.03	0.72
methylamine − methylamine	$CH_3NH_2{-}CH_3NH_2$	0.56	0.79	0.86	0.90	0.92	0.95	1.47	0.92
methylamine − methanol	$CH_3NH_2{-}CH_3OH$	0.42	0.59	0.59	0.70	0.74	0.74	1.03	0.73
methylamine − peptide	$CH_3NH_2{-}C_6H_7NO_2$	0.92	1.10	1.26	1.17	1.19	1.31	1.73	1.17
methylamine − pyridine	$CH_3NH_2{-}C_5H_5N$	0.78	0.94	1.13	1.17	1.30	1.47	1.51	1.29
methylamine − water	$CH_3NH_2{-}H_2O$	0.32	0.87	0.84	1.03	1.07	1.01	1.48	0.99
methanol − methylamine	$CH_3OH{-}CH_3NH_2$	0.36	0.82	0.81	1.16	1.06	0.98	1.43	1.07
methanol − methanol	$CH_3OH{-}CH_3OH$	0.23	0.48	0.52	0.52	0.70	0.70	0.96	0.70
methanol − peptide	$CH_3OH{-}CH_3OH$	0.52	0.72	0.79	0.82	0.82	0.82	1.34	0.82
methanol − pyridine	$CH_3OH{-}C_5H_5N$	0.14	0.59	0.61	0.92	1.10	1.03	1.14	1.14
methanol − water	$CH_3OH{-}H_2O$	0.32	0.38	0.42	0.40	0.46	0.46	0.91	0.46
neopentane − neopentane	$C_5H_{12}{-}C_5H_{12}$	1.15	1.37	1.29	1.73	1.60	1.69	1.90	1.54
neopentane − pentane	$C_5H_{12}{-}C_5H_{12}$	1.59	1.74	1.81	2.01	1.94	2.04	2.33	1.80
pentane − acetamide	$C_5H_{12}{-}CH_3CONH_2$	1.52	1.59	1.65	1.83	1.71	1.84	2.17	1.62
pentane − aceticacid	$C_5H_{12}{-}CH_3COOH$	1.43	1.58	1.57	1.68	1.72	1.86	1.88	1.57
pentane − pentane	$C_5H_{12}{-}C_5H_{12}$	2.24	2.39	2.61	2.70	2.57	2.62	3.13	2.35
peptide − ethene	$C_6H_7NO_2{-}CH_2H_4$	0.77	0.93	0.99	1.06	1.08	1.18	1.45	1.04
peptide − methylamine	$C_6H_7NO_2{-}CH_3NH_2$	0.57	1.16	1.26	1.53	1.50	1.57	1.75	1.50
peptide − methanol	$C_6H_7NO_2{-}CH_3OH$	0.42	0.88	0.99	1.00	1.11	1.23	1.44	1.11
peptide − pentane	$C_6H_7NO_2{-}C_5H_{12}$	1.95	2.12	2.24	2.25	2.31	2.49	2.66	2.21
peptide − peptide	$C_6H_7NO_2{-}C_6H_7NO_2$	0.76	1.01	1.12	1.42	1.39	1.43	1.62	1.35
peptide − water	$C_6H_7NO_2{-}H_2O$	0.39	0.53	0.56	0.57	0.66	0.72	1.04	0.63
pyridine − ethene	$C_5H_5N{-}C_2H_4$	0.96	1.02	1.35	1.47	1.22	1.41	1.57	1.29
pyridine − ethyne	$C_5H_5N{-}C_2H_2$	0.75	0.93	1.03	1.13	1.41	1.41	1.42	1.41
pyridine − pyridine_CH − N	$C_5H_5N{-}C_5H_5N$	0.32	0.77	0.80	0.80	1.32	1.45	1.51	1.38
pyridine − pyridine_π − π	$C_5H_5N{-}C_5H_5N$	1.62	1.97	2.45	2.47	2.65	3.10	2.12	2.78
pyridine − pyridine_TS	$C_5H_5N{-}C_5H_5N$	0.85	1.21	1.42	1.46	1.82	2.15	1.63	1.77
pyridine − uracil_π − π	$C_5H_5N{-}C_4H_4N_2O_2$	1.95	2.23	2.69	2.45	3.02	3.65	2.12	3.23
uracil − cyclopentane	$C_4H_4N_2O_2{-}C_5H_{10}$	1.85	2.05	2.19	2.32	2.52	2.87	2.20	2.40
uracil − ethene	$C_4H_4N_2O_2{-}C_2H_4$	1.01	1.17	1.42	1.47	1.42	1.66	1.55	1.44
uracil − ethyne	$C_4H_4N_2O_2{-}C_2H_2$	0.78	0.90	1.20	1.02	1.03	1.27	1.33	1.08
uracil − neopentane	$C_4H_4N_2O_2{-}C_5H_{12}$	1.55	1.77	1.87	2.05	2.09	2.38	1.96	1.90
uracil − pentane	$C_4H_4N_2O_2{-}C_5H_{12}$	2.15	2.31	2.47	2.64	2.71	3.09	2.50	2.59
uracil − uracil_BP	$C_4H_4N_2O_2{-}C_4H_4N_2O_2$	0.11	0.50	0.71	1.00	1.37	1.23	1.39	1.51
uracil − uracil_π − π	$C_4H_4N_2O_2{-}C_4H_4N_2O_2$	2.19	2.23	2.67	2.22	2.99	3.60	1.73	3.27
water − methylamine	$H_2O{-}CH_3NH_2$	0.35	0.80	0.76	1.09	1.03	0.91	1.39	0.95
water − methanol	$H_2O{-}CH_3OH$	0.23	0.52	0.53	0.56	0.71	0.70	1.09	0.65
water − peptide	$H_2O{-}C_6H_7NO_2$	0.48	0.75	0.76	0.77	0.77	0.71	1.45	0.70
water − pyridine	$H_2O{-}C_5H_5N$	0.20	0.68	0.64	0.96	1.11	1.02	1.28	1.04
water − water	$H_2O{-}H_2O$	0.34	0.43	0.45	0.46	0.50	0.49	1.03	0.48

SAPT-DFT

Table S64: Absolute deviations for SAPT-DFT. For SAPT-DFT method we used the B3LYP, PBE0 and ωB97X DFA's in conjunction with the aug-cc-pVDZ basis set. Data: 66 interaction energies. All values in kcal/mol.

Dimer	Formula	SAPT-B3LYP	SAPT-PBE0	SAPT-ωB97X
acetamide − acetamide	$CH_3CONH_2{-}CH_3CONH_2$	3.27	2.54	2.93
acetamide − uracil	$CH_3CONH_2{-}C_4H_4N_2O_2$	3.64	2.81	3.11
aceticacid − aceticacid	$CH_3COOH{-}CH_3COOH$	4.73	3.66	3.62
aceticacid − uracil	$CH_3COOH{-}C_4H_4N_2O_2$	4.27	3.36	3.42
benzene − acetamide_NH − π	$C_6H_6{-}CH_3CONH_2$	0.77	0.50	0.90
benzene − aceticacid	$C_6H_6{-}CH_3COOH$	0.60	0.50	0.96
benzene − aceticacid_OH − π	$C_6H_6{-}CH_3COOH$	1.04	0.67	0.99
benzene − benzene_π − π	$C_6H_6{-}C_6H_6$	0.04	0.15	0.83
benzene − benzene_TS	$C_6H_6{-}C_6H_6$	0.40	0.31	0.73
benzene − cyclopentane	$C_6H_6{-}C_5H_{10}$	0.46	0.48	1.01
benzene − ethene	$C_6H_6{-}C_2H_4$	0.05	0.18	0.74
benzene − ethyne_CH − π	$C_6H_6{-}C_2H_2$	0.44	0.23	0.50
benzene − methylamine_NH − π	$C_6H_6{-}CH_3NH_2$	0.59	0.48	0.87
benzene − methanol_OH − π	$C_6H_6{-}CH_3OH$	0.74	0.51	1.02
benzene − neopentane	$C_6H_6{-}C_5H_{12}$	0.32	0.32	0.81
benzene − peptide_NH − π	$C_6H_6{-}C_6H_7NO_2$	0.91	0.66	1.06
benzene − pyridine_π − π	$C_6H_6{-}C_5H_5N$	0.15	0.22	0.82
benzene − pyridine_TS	$C_6H_6{-}C_5H_5N$	0.50	0.37	0.70
benzene − uracil_π − π	$C_6H_6{-}C_4H_4N_2O_2$	0.88	0.88	1.61
benzene − water_OH − π	$C_6H_6{-}H_2O$	0.68	0.45	0.71
cyclopentane − cyclopentane	$C_5H_{10}{-}C_5H_{10}$	0.64	0.67	1.01
cyclopentane − neopentane	$C_5H_{10}{-}C_5H_{12}$	0.49	0.52	0.89
ethene − pentane	$C_2H_4{-}C_5H_{12}$	0.37	0.39	0.80
ethyne − aceticacid_OH − π	$C_2H_2{-}CH_3COOH$	1.04	0.80	1.01
ethyne − ethyne_TS	$C_2H_2{-}C_2H_2$	0.09	0.04	0.22
ethyne − pentane	$C_2H_2{-}C_5H_{12}$	0.17	0.22	0.60
ethyne − water_CH − O	$C_2H_2{-}H_2O$	0.31	0.20	0.40
methylamine − methylamine	$CH_3NH_2{-}CH_3NH_2$	1.02	0.87	1.12
methylamine − methanol	$CH_3NH_2{-}CH_3OH$	0.66	0.58	0.85
methylamine − peptide	$CH_3NH_2{-}C_6H_7NO_2$	1.20	1.04	1.23
methylamine − pyridine	$CH_3NH_2{-}C_5H_5N$	0.73	0.67	0.99
methylamine − water	$CH_3NH_2{-}H_2O$	1.50	1.17	1.46
methanol − methylamine	$CH_3OH{-}CH_3NH_2$	1.54	1.15	1.61
methanol − methanol	$CH_3OH{-}CH_3OH$	1.28	1.09	1.36
methanol − peptide	$CH_3OH{-}CH_3OH$	1.81	1.51	1.73
methanol − pyridine	$CH_3OH{-}C_5H_5N$	1.33	1.12	1.39
methanol − water	$CH_3OH{-}H_2O$	0.94	0.77	1.03
neopentane − neopentane	$C_5H_{12}{-}C_5H_{12}$	0.27	0.33	0.64
neopentane − pentane	$C_5H_{12}{-}C_5H_{12}$	0.57	0.60	0.91
pentane − acetamide	$C_5H_{12}{-}CH_3CONH_2$	0.69	0.68	1.19
pentane − aceticacid	$C_5H_{12}{-}CH_3COOH$	0.66	0.65	0.99
pentane − pentane	$C_5H_{12}{-}C_5H_{12}$	0.89	0.97	1.26
peptide − ethene	$C_6H_7NO_2{-}CH_2H_4$	0.54	0.48	0.82
peptide − methylamine	$C_6H_7NO_2{-}CH_3NH_2$	1.62	1.26	1.55
peptide − methanol	$C_6H_7NO_2{-}CH_3OH$	1.24	1.05	1.33
peptide − pentane	$C_6H_7NO_2{-}C_5H_{12}$	1.12	1.11	1.40
peptide − peptide	$C_6H_7NO_2{-}C_6H_7NO_2$	1.59	1.31	1.78
peptide − water	$C_6H_7NO_2{-}H_2O$	0.83	0.67	0.88
pyridine − ethene	$C_5H_5N{-}C_2H_4$	0.10	0.23	0.75
pyridine − ethyne	$C_5H_5N{-}C_2H_2$	0.59	0.47	0.55
pyridine − pyridine_CH − N	$C_5H_5N{-}C_5H_5N$	0.88	0.76	0.80
pyridine − pyridine_π − π	$C_5H_5N{-}C_5H_5N$	0.21	0.27	0.75
pyridine − pyridine_TS	$C_5H_5N{-}C_5H_5N$	0.60	0.48	0.74
pyridine − uracil_π − π	$C_5H_5N{-}C_4H_4N_2O_2$	0.83	0.88	1.41
uracil − cyclopentane	$C_4H_4N_2O_2{-}C_5H_{10}$	0.91	0.92	1.35
uracil − ethene	$C_4H_4N_2O_2{-}C_2H_4$	0.51	0.58	1.09
uracil − ethyne	$C_4H_4N_2O_2{-}C_2H_2$	0.51	0.58	1.00
uracil − neopentane	$C_4H_4N_2O_2{-}C_5H_{12}$	0.65	0.67	1.15
uracil − pentane	$C_4H_4N_2O_2{-}C_5H_{12}$	1.12	1.11	1.55
uracil − uracil_BP	$C_4H_4N_2O_2{-}C_4H_4N_2O_2$	3.60	2.84	3.03
uracil − uracil_π − π	$C_4H_4N_2O_2{-}C_4H_4N_2O_2$	1.31	1.14	1.93
water − methylamine	$H_2O{-}CH_3NH_2$	1.41	1.09	1.35
water − methanol	$H_2O{-}CH_3OH$	1.25	1.07	1.28
water − peptide	$H_2O{-}C_6H_7NO_2$	1.62	1.37	1.74
water − pyridine	$H_2O{-}C_5H_5N$	1.25	1.09	1.22
water − water	$H_2O{-}H_2O$	0.92	0.77	0.97

6.2 aug-cc-pVTZ basis set data.

6.2.1 Ionization Potentials and HOMO values for SAPT-DFT calculations

Table S65: Experimental ionization potentials (IP) of molecules comprising dimers in the S66 database. IP obtained from NIST (https://webbook.nist.gov/chemistry). Kohn-Sham energies of HOMO orbitals were calculated at the DFA/aug-cc-pVTZ level of theory (DFA=B3LYP, PBE0, and ωB97X). Ionization energies in ev and HOMO energies in atomic units.

			HOMO energies (eV)			$\Delta_{XC} = \epsilon_{HOMO} - (-IP)$		
Molecule	Formula	IP	B3LYP	PBE0	ωB97X	B3LYP	PBE0	ωB97X
acetamide	CH_3CONH_2	10.00	-7.05	-7.38	-10.70	0.11	0.10	0.03
aceticacid	CH_3COOH	10.65	-7.87	-8.20	-9.87	0.10	0.09	0.03
benzene	C_6H_6	9.24	-6.99	-7.30	-9.47	0.08	0.07	0.01
cyclopentane	C_5H_{10}	10.33	-8.47	-8.73	-11.20	0.07	0.06	0.03
ethene	C_2H_4	10.51	-7.56	-7.89	-10.30	0.11	0.10	0.01
ethyne	C_2H_2	11.40	-8.07	-8.40	-10.85	0.12	0.11	0.02
methylamide	CH_3NH_2	8.90	-6.64	-6.95	-9.46	0.08	0.07	0.02
methanol	CH_3OH	10.84	-7.67	-7.99	-10.52	0.12	0.10	0.01
neopentane	C_5H_{12}	10.90	-8.70	-8.98	-11.42	0.08	0.07	0.02
pentane	C_5H_{12}	10.28	-8.56	-8.86	-11.27	0.06	0.05	0.04
peptide	C_2H_5NO	9.70	-6.93	-7.26	-9.72	0.10	0.09	0.00
pyridine	C_5H_5N	9.26	-7.16	-7.50	-9.83	0.08	0.06	0.02
uracil	$C_4H_4N_2O_2$	9.20	-7.23	-7.52	-9.71	0.07	0.06	0.02
water	H_2O	12.62	-8.74	-9.08	-11.70	0.14	0.13	0.03

6.2.2 Evaluation of mean absolute deviation in a grid of C_{OS} and C_{SS} values

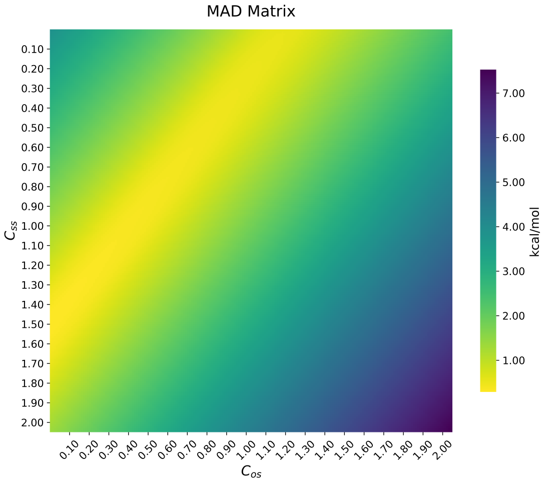

(a)

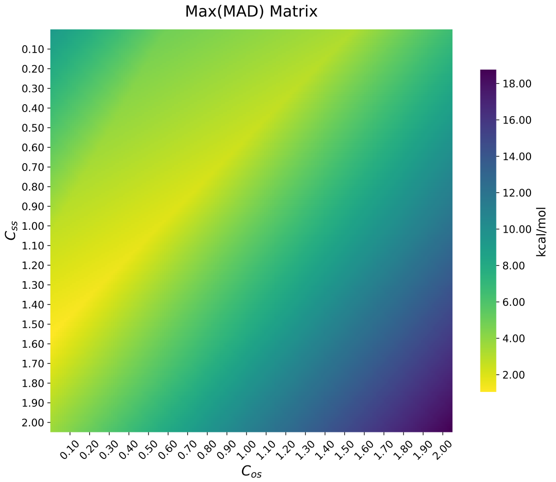

(b)

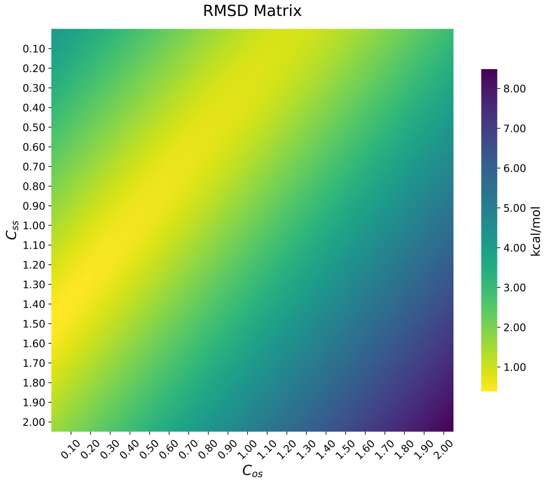

(c)

Figure S14: Evaluation of MADs (S14a), Max(MADs) (S14b) and RMSDs (S14c) in grid of values C_{OS} and C_{SS}. The optimal coefficients are C_{OS}=0.27 and C_{SS}=1.38. The theory level used is RIJK-MP2/aug-cc-pVDZ.

6.2.3 Interaction energies

MP2 with C_{OS} y C_{SS} values

Table S66: Interaction energies for CCSD(T)/CBS, MP2, SCS-MP2, SCS(MI)-MP2, SCSN-MP2, SCS-MP2-vdW, RI-MP2, RI-SCS-MP2, RIJK-MP2, RIJK-SCS-MP2, RIJCOSX-MP2, RIJCOSX-SCS-MP2, SCS-MP2^{BWI-TZ}, RI-SCS-MP2^{BWI-TZ}, RIJK-SCS-MP2^{BWI-TZ}, RIJCOSX-SCS-MP2^{BWI-DZ} and SCS-MP2-hal^{G-XZ}. Data: 66 interaction energies. MP2: $C_{OS} = C_{SS}$ =1.00; SCS-MP2: C_{OS} =1.20, C_{SS} =0.33; SCS(MI)-MP2: C_{OS} =0.40, C_{SS} =1.29; SCSN-MP2: C_{OS} =0.00, C_{SS} =1.76; SCS-MP2-vdW: C_{OS} =1.28, C_{SS} =0.50; SCS-MP2^{BWI-TZ} RI-SCS-MP2^{BWI-TZ} , RIJK-SCS-MP2^{BWI-TZ} : C_{OS} =0.27, C_{SS} =1.38; RIJCOSX-SCS-MP2^{BWI-TZ} : C_{OS} =0.17, C_{SS} =1.59. All values in kcal/mol.

Dimer	Formula	CCSD(T)/CBS	MP2	SCS-MP2	SCS(MI)-MP2	SCSN-MP2	SCS-MP2-vdW	RI-MP2	RI-SCS-MP2	RIJK-MP2	RIJK-SCS-MP2	RIJCOSX-MP2	RIJCOSX-SCS-MP2	SCS-MP2^{BWI-TZ}	RI-SCS-MP2^{BWI-TZ}	RIJK-SCS-MP2^{BWI-TZ}	RIJCOSX-SCS-MP2^{BWI-TZ}	SCS-MP2-hal^{G-XZ}
acetamide − acetamide	CH_3CONH_2	-15.60	-15.78	-14.32	-15.05	-15.39	-15.04	-15.78	-14.32	-15.78	-14.32	-15.73	-14.27	-14.98	-14.98	-14.98	-14.96	-13.94
acetamide − uracil	CH_3CONH_2	-18.44	-18.76	-16.97	-18.22	-18.81	-17.76	-18.76	-16.97	-18.75	-16.97	-18.67	-16.89	-18.20	-18.20	-18.19	-18.16	-16.53
aceticacid − aceticacid	CH_3COOH	-18.39	-17.37	-15.62	-17.23	-18.00	-16.29	-17.37	-15.62	-17.36	-15.61	-17.32	-15.57	-17.29	-17.29	-17.27	-17.28	-15.22
aceticacid − uracil	CH_3COOH	-18.73	-18.47	-16.65	-18.18	-18.92	-17.38	-18.47	-16.65	-18.46	-16.64	-18.40	-16.57	-18.21	-18.21	-18.20	-18.18	-16.21
benzene − acetamide − NH − π	C_6H_6	-3.84	-5.52	-4.62	-4.57	-4.52	-5.19	-5.52	-4.62	-5.53	-4.63	-5.44	-4.54	-4.42	-4.42	-4.42	-4.35	-4.35
benzene − aceticacid	C_6H_6	-3.10	-5.64	-4.39	-4.23	-4.12	-5.20	-5.64	-4.39	-5.65	-4.40	-5.59	-4.34	-4.01	-4.01	-4.02	-3.98	-4.00
benzene − aceticacid − OH − π	C_6H_6	-4.12	-6.26	-5.25	-5.13	-5.05	-5.91	-6.26	-5.25	-6.28	-5.27	-6.21	-5.20	-4.96	-4.96	-4.97	-4.91	-4.94
benzene − benzene − π − π	C_6H_6	-1.76	-5.90	-4.20	-3.82	-3.58	-5.35	-5.90	-4.20	-5.93	-4.23	-5.86	-4.16	-3.48	-3.48	-3.50	-3.46	-3.66
benzene − benzene − TS	C_6H_6	-2.24	-4.91	-3.83	-3.55	-3.38	-4.57	-4.91	-3.83	-4.92	-3.84	-4.84	-3.76	-3.33	-3.33	-3.34	-3.27	-3.49
benzene − cyclopentane	C_6H_6	-2.67	-5.98	-4.48	-4.09	-3.86	-5.51	-5.98	-4.48	-5.99	-4.48	-5.92	-4.42	-3.78	-3.78	-3.78	-3.74	-3.99
benzene − ethene	C_6H_6	-0.83	-2.90	-1.92	-1.68	-1.54	-2.59	-2.90	-1.92	-2.90	-1.92	-2.87	-1.89	-1.49	-1.49	-1.48	-1.47	-1.60
benzene − ethyne − CH − π	C_6H_6	-2.49	-4.30	-3.55	-3.47	-3.41	-4.03	-4.30	-3.55	-4.31	-3.56	-4.25	-3.50	-3.34	-3.34	-3.35	-3.30	-3.31
benzene − methylamide − NH − π	C_6H_6	-2.65	-4.70	-3.68	-3.47	-3.35	-4.36	-4.70	-3.68	-4.72	-3.70	-4.66	-3.64	-3.28	-3.28	-3.29	-3.25	-3.36
benzene − methanol − OH − π	C_6H_6	-3.60	-5.69	-4.64	-4.48	-4.38	-5.33	-5.69	-4.64	-5.70	-4.66	-5.62	-4.58	-4.29	-4.29	-4.30	-4.24	-4.31
benzene − neopentane	C_6H_6	-2.18	-4.81	-3.66	-3.30	-3.09	-4.46	-4.81	-3.66	-4.82	-3.66	-4.74	-3.58	-3.05	-3.05	-3.06	-2.99	-3.28
benzene − peptide − NH − π	C_6H_6	-4.46	-7.58	-6.17	-5.85	-5.66	-7.12	-7.58	-6.17	-7.60	-6.19	-7.52	-6.11	-5.57	-5.57	-5.59	-5.53	-5.71
benzene − pyridine − π − π	C_6H_6	-2.34	-6.58	-4.79	-4.45	-4.24	-5.98	-6.58	-4.79	-6.60	-4.81	-6.55	-4.76	-4.11	-4.11	-4.13	-4.11	-4.22
benzene − pyridine − TS	C_6H_6	-2.70	-5.26	-4.17	-3.96	-3.83	-4.90	-5.26	-4.17	-5.28	-4.19	-5.21	-4.12	-3.75	-3.75	-3.77	-3.71	-3.83
benzene − uracil − π − π	C_6H_6	-4.28	-9.21	-6.92	-6.65	-6.46	-8.41	-9.21	-6.92	-9.23	-6.93	-9.18	-6.89	-6.25	-6.25	-6.26	-6.26	-6.21
benzene − water − OH − π	C_6H_6	-2.95	-4.20	-3.57	-3.46	-3.39	-3.99	-4.20	-3.57	-4.21	-3.59	-4.15	-3.53	-3.35	-3.35	-3.36	-3.31	-3.38
cyclopentane − cyclopentane	C_5H_{10}	-2.26	-4.21	-3.04	-2.76	-2.58	-3.84	-4.21	-3.04	-4.22	-3.06	-4.10	-2.93	-2.52	-2.52	-2.53	-2.43	-2.67
cyclopentane − neopentane	C_5H_{10}	-1.78	-3.45	-2.51	-2.19	-2.01	-3.17	-3.45	-2.51	-3.45	-2.50	-3.42	-2.47	-1.99	-1.99	-1.98	-1.96	-2.20
ethene − pentane	C_2H_4	-1.52	-2.75	-1.94	-1.78	-1.68	-2.48	-2.75	-1.94	-2.75	-1.94	2.27	1.98	-1.62	-1.62	-1.62	2.48	-1.69
ethyne − aceticacid − OH − π	C_2H_2	-4.52	-5.32	-4.61	-4.91	-5.05	-4.97	-5.32	-4.61	-5.32	-4.61	-5.31	-4.59	-4.86	-4.86	-4.86	-4.86	-4.41
ethyne − ethyne − TS	C_2H_2	-1.37	-1.95	-1.65	-1.67	-1.67	-1.83	-1.95	-1.65	-1.95	-1.65	-1.94	-1.64	-1.62	-1.62	-1.63	-1.62	-1.56
ethyne − pentane	C_2H_2	-1.34	-2.51	-1.79	-1.65	-1.57	-2.27	-2.51	-1.79	-2.51	-1.79	-2.48	-1.76	-1.52	-1.52	-1.51	-1.50	-1.56
ethyne − water − CH − O	C_2H_2	-2.73	-3.16	-2.87	-3.06	-3.16	-3.00	-3.16	-2.87	-3.16	-2.87	-3.15	-2.86	-3.06	-3.06	-3.06	-3.06	-2.79
methylamide − methylamide	CH_3NH_2	-3.75	-4.61	-3.85	-3.89	-3.90	-4.31	-4.61	-3.85	-4.60	-3.84	-4.56	-3.80	-3.78	-3.78	-3.78	-3.75	-3.62
methylamide − methanol	CH_3NH_2	-2.78	-3.40	-2.90	-2.92	-2.92	-3.21	-3.40	-2.90	-3.40	-2.90	-3.38	-2.88	-2.85	-2.85	-2.85	-2.83	-2.75
methylamide − peptide	CH_3NH_2	-4.89	-6.18	-5.22	-5.35	-5.40	-5.78	-6.18	-5.22	-6.17	-5.21	-6.14	-5.17	-5.23	-5.23	-5.22	-5.21	-4.94
methylamide − pyridine	CH_3NH_2	-3.36	-5.16	-4.09	-4.02	-3.95	-4.77	-5.16	-4.09	-5.15	-4.08	-5.11	-4.04	-3.84	-3.84	-3.83	-3.80	-3.76
methylamide − water	CH_3NH_2	-6.92	-7.75	-6.99	-7.14	-7.20	-7.42	-7.75	-6.99	-7.75	-6.99	-7.69	-6.93	-7.06	-7.06	-7.06	-7.02	-6.77
methanol − methylamide	CH_3OH	-7.09	-7.91	-7.00	-7.23	-7.33	-7.51	-7.91	-7.00	-7.90	-6.99	-7.86	-6.95	-7.14	-7.14	-7.13	-7.11	-6.74
methanol − methanol	CH_3OH	-5.45	-6.14	-5.50	-5.69	-5.77	-5.85	-6.14	-5.50	-6.13	-5.50	-6.10	-5.47	-5.63	-5.63	-5.62	-5.61	-5.32
methanol − peptide	CH_3OH	-7.79	-8.53	-7.62	-8.03	-8.21	-8.08	-8.53	-7.62	-8.53	-7.63	-8.52	-7.61	-7.97	-7.97	-7.97	-7.97	-7.38
methanol − pyridine	CH_3OH	-6.93	-8.03	-7.16	-7.29	-7.33	-7.67	-8.03	-7.16	-8.03	-7.16	-7.99	-7.12	-7.18	-7.18	-7.18	-7.15	-6.91
methanol − water	CH_3OH	-4.79	-5.23	-4.75	-5.00	-5.12	-4.98	-5.23	-4.75	-5.23	-4.75	-5.21	-4.73	-4.98	-4.98	-4.98	-4.96	-4.62
neopentane − neopentane	C_5H_{12}	-1.31	-2.52	-1.86	-1.57	-1.40	-2.35	-2.52	-1.86	-2.53	-1.87	-2.46	-1.79	-1.40	-1.40	-1.41	-1.34	-1.64
neopentane − pentane	C_5H_{12}	-1.97	-3.65	-2.64	-2.35	-2.18	-3.34	-3.65	-2.64	-3.65	-2.64	-3.56	-2.55	-2.13	-2.13	-2.13	-2.07	-2.31
pentane − acetamide	C_5H_{12}	-2.75	-4.65	-3.43	-3.23	-3.10	-4.23	-4.65	-3.43	-4.65	-3.43	-4.60	-3.37	-3.00	-3.00	-3.00	-2.98	-3.04
pentane − aceticacid	C_5H_{12}	-2.24	-3.99	-2.94	-2.73	-2.60	-3.64	-3.99	-2.94	-4.00	-2.94	-3.93	-2.88	-2.52	-2.52	-2.53	-2.48	-2.60
pentane − pentane	C_5H_{12}	-2.87	-5.17	-3.67	-3.35	-3.16	-4.67	-5.17	-3.67	-5.17	-3.67	-5.08	-3.57	-3.06	-3.06	-3.06	-3.00	-3.19
peptide − ethene	$C_6H_7NO_2$	-2.51	-3.72	-2.88	-2.90	-2.89	-3.40	-3.72	-2.88	-3.72	-2.88	-3.71	-2.87	-2.77	-2.77	-2.77	-2.77	-2.63
peptide − methylamide	$C_6H_7NO_2$	-6.89	-8.32	-7.37	-7.33	-7.28	-7.97	-8.32	-7.37	-8.32	-7.37	-8.27	-7.32	-7.17	-7.17	-7.17	-7.13	-7.08
peptide − methanol	$C_6H_7NO_2$	-5.78	-7.09	-6.35	-6.34	-6.32	-6.81	-7.09	-6.35	-7.09	-6.35	-7.05	-6.32	-6.23	-6.23	-6.23	-6.21	-6.13
peptide − pentane	$C_6H_7NO_2$	-3.32	-5.75	-4.22	-3.95	-3.78	-5.23	-5.75	-4.22	-5.75	-4.22	-5.67	-4.14	-3.66	-3.66	-3.66	-3.61	-3.73
peptide − peptide	$C_6H_7NO_2$	-8.03	-9.35	-8.22	-8.42	-8.50	-8.87	-9.35	-8.22	-9.35	-8.22	-9.29	-8.16	-8.29	-8.29	-8.29	-8.25	-7.89
peptide − water	$C_6H_7NO_2$	-4.89	-5.53	-5.02	-5.17	-5.23	-5.30	-5.53	-5.02	-5.54	-5.02	-5.52	-5.01	-5.12	-5.12	-5.12	-5.11	-4.87
pyridine − ethene	C_5H_5N	-1.23	-3.38	-2.32	-2.13	-2.01	-3.02	-3.38	-2.32	-3.38	-2.32	-3.36	-2.30	-1.93	-1.93	-1.93	-1.93	-1.98
pyridine − ethyne	C_5H_5N	-3.72	-4.73	-4.23	-4.33	-4.37	-4.51	-4.73	-4.23	-4.73	-4.23	-4.66	-4.16	-4.27	-4.27	-4.27	-4.21	-4.08
pyridine − pyridine − CH − N	C_5H_5N	-3.69	-5.00	-4.26	-4.20	-4.15	-4.74	-5.00	-4.26	-5.00	-4.26	-4.64	-3.90	-4.07	-4.07	-4.07	-3.72	-4.03
pyridine − pyridine − π − π	C_5H_5N	-2.77	-7.07	-5.22	-4.91	-4.71	-6.44	-7.07	-5.22	-7.09	-5.23	-7.04	-5.18	-4.56	-4.56	-4.58	-4.56	-4.63
pyridine − pyridine − TS	C_5H_5N	-2.89	-5.30	-4.17	-4.04	-3.94	-4.91	-5.30	-4.17	-5.31	-4.18	-5.22	-4.09	-3.83	-3.83	-3.84	-3.77	-3.82
pyridine − uracil − π − π	C_5H_5N	-5.41	-10.12	-7.80	-7.69	-7.58	-9.26	-10.12	-7.80	-10.12	-7.80	-10.10	-7.78	-7.31	-7.31	-7.31	-7.33	-7.09
uracil − cyclopentane	$C_4H_4N_2O_2$	-3.08	-6.35	-4.74	-4.32	-4.08	-5.84	-6.35	-4.74	-6.35	-4.74	-6.30	-4.69	-3.99	-3.99	-3.99	-3.98	-4.22
uracil − ethene	$C_4H_4N_2O_2$	-2.68	-4.80	-3.63	-3.59	-3.54	-4.37	-4.80	-3.63	-4.80	-3.63	-4.79	-3.61	-3.40	-3.40	-3.40	-3.40	-3.27
uracil − ethyne	$C_4H_4N_2O_2$	-3.12	-4.92	-3.81	-3.92	-3.95	-4.47	-4.92	-3.81	-4.91	-3.81	-4.91	-3.81	-3.77	-3.77	-3.76	-3.79	-3.49
uracil − neopentane	$C_4H_4N_2O_2$	-2.87	-5.37	-4.08	-3.80	-3.62	-4.96	-5.37	-4.08	-5.38	-4.09	-5.35	-4.06	-3.54	-3.54	-3.55	-3.54	-3.67
uracil − pentane	$C_4H_4N_2O_2$	-3.66	-7.17	-5.31	-4.94	-4.71	-6.55	-7.17	-5.31	-7.18	-5.32	-7.10	-5.24	-4.58	-4.58	-4.59	-4.54	-4.72
uracil − uracil − BP	$C_4H_4N_2O_2$	-16.29	-17.31	-15.36	-16.70	-17.34	-16.22	-17.31	-15.36	-17.30	-15.36	-16.96	-15.01	-16.68	-16.68	-16.67	-16.38	-14.87
uracil − uracil − π − π	$C_4H_4N_2O_2$	-8.12	-12.63	-9.89	-10.03	-10.04	-11.55	-12.63	-9.89	-12.65	-9.90	-12.60	-9.86	-9.64	-9.64	-9.65	-9.66	-9.07
water − methylamide	H_2O	-6.60	-7.30	-6.65	-6.80	-6.86	-7.02	-7.30	-6.65	-7.30	-6.65	-7.26	-6.61	-6.73	-6.73	-6.73	-6.70	-6.47
water − methanol	H_2O	-5.35	-5.98	-5.44	-5.57	-5.62	-5.74	-5.98	-5.44	-5.97	-5.43	-5.95	-5.41	-5.51	-5.51	-5.51	-5.49	-5.28
water − peptide	H_2O	-7.80	-8.31	-7.51	-7.95	-8.15	-7.90	-8.31	-7.51	-8.31	-7.51	-8.28	-7.48	-7.91	-7.91	-7.91	-7.90	-7.31
water − pyridine	H_2O	-6.51	-7.45	-6.80	-6.85	-6.86	-7.19	-7.45	-6.80	-7.45	-6.80	-7.40	-6.75	-6.76	-6.76	-6.75	-6.72	-6.61
water − water	H_2O	-4.75	-5.16	-4.76	-4.95	-5.05	-4.96	-5.16	-4.76	-5.16	-4.76	-5.13	-4.73	-4.93	-4.93	-4.93	-4.91	-4.65

DFT

Table S67: Interaction energies for CCSD(T)/CBS, B97M-V, ωB97X-V, ωB97M-V, ωB97X-D3, B2PLYP-D3BJ, DSD-BLYP-D3BJ, ωB97X-D4 and B2PLYP-D4. Data: 66 interaction energies. All values in kcal/mol.

Dimer	Formula	CCSD(T)/CBS	B97M-V	ωB97X-V	ωB97M-V	ωB97X-D3	B2PLYP-D3BJ	DSD-BLYP-D3BJ	ωB97X-D4	B2PLYP-D4
acetamide − acetamide	$CH_3CONH_2-CH_3CONH_2$	-15.60	-14.92	-15.11	-15.14	-15.47	-15.63	-15.53	-16.17	-15.70
acetamide − uracil	$CH_3CONH_2-C_4H_4N_2O_2$	-18.44	-17.53	-17.79	-17.84	-18.13	-18.47	-18.38	-18.69	-18.56
aceticacid − aceticacid	$CH_3COOH-CH_3COOH$	-18.39	-17.00	-17.53	-17.41	-17.91	-17.64	-17.45	-18.55	-17.70
aceticacid − uracil	$CH_3COOH-C_4H_4N_2O_2$	-18.73	-17.55	-17.92	-17.92	-18.29	-18.41	-18.28	-18.85	-18.48
benzene − acetamide_NH − π	$C_6H_6-CH_3CONH_2$	-3.84	-4.06	-4.32	-4.38	-4.52	-4.49	-4.63	-4.73	-4.44
benzene − aceticacid	$C_6H_6-CH_3COOH$	-3.10	-3.68	-3.96	-4.10	-4.14	-4.03	-4.23	-4.22	-3.95
benzene − aceticacid_OH − π	$C_6H_6-CH_3COOH$	-4.12	-4.45	-4.87	-4.98	-4.96	-4.89	-5.11	-5.01	-4.80
benzene − benzene_π − π	$C_6H_6-C_6H_6$	-1.76	-2.82	-2.89	-3.12	-3.40	-3.40	-3.62	-3.26	-3.52
benzene − benzene_TS	$C_6H_6-C_6H_6$	-2.24	-2.64	-2.86	-2.97	-3.18	-3.25	-3.46	-3.30	-3.19
benzene − cyclopentane	$C_6H_6-C_5H_{10}$	-2.67	-3.68	-3.75	-3.90	-4.20	-3.97	-4.15	-4.19	-3.88
benzene − ethene	$C_6H_6-C_2H_4$	-0.83	-1.50	-1.44	-1.58	-1.84	-1.61	-1.70	-1.99	-1.67
benzene − ethyne_CH − π	$C_6H_6-C_2H_2$	-2.49	-2.65	-2.95	-2.98	-3.01	-3.16	-3.34	-3.32	-3.03
benzene − methylamine_NH − π	$C_6H_6-CH_3NH_2$	-2.65	-3.05	-3.21	-3.33	-3.58	-3.39	-3.54	-3.67	-3.30
benzene − methanol_OH − π	$C_6H_6-CH_3OH$	-3.60	-3.98	-4.23	-4.35	-4.49	-4.35	-4.53	-4.58	-4.24
benzene − neopentane	$C_6H_6-C_5H_{12}$	-2.18	-2.94	-3.12	-3.19	-3.59	-3.16	-3.34	-3.57	-3.06
benzene − peptide_NH − π	$C_6H_6-C_6H_7NO_2$	-4.46	-4.99	-5.34	-5.48	-5.79	-5.59	-5.84	-5.74	-5.49
benzene − pyridine_π − π	$C_6H_6-C_5H_5N$	-2.34	-3.37	-3.46	-3.69	-3.88	-4.03	-4.26	-3.73	-4.13
benzene − pyridine_TS	$C_6H_6-C_5H_5N$	-2.70	-2.99	-3.31	-3.41	-3.57	-3.69	-3.89	-3.73	-3.61
benzene − uracil_π − π	$C_6H_6-C_4H_4N_2O_2$	-4.28	-5.54	-5.71	-6.08	-5.93	-6.19	-6.52	-5.71	-6.33
benzene − water_OH − π	$C_6H_6-H_2O$	-2.95	-3.12	-3.40	-3.45	-3.66	-3.41	-3.54	-3.76	-3.20
cyclopentane − cyclopentane	$C_5H_{10}-C_5H_{10}$	-2.26	-3.18	-3.21	-3.19	-3.48	-3.15	-3.17	-3.74	-3.06
cyclopentane − neopentane	$C_5H_{10}-C_5H_{12}$	-1.78	-2.60	-2.60	-2.56	-3.00	-2.54	-2.56	-3.18	-2.48
ethene − pentane	$C_2H_4-C_5H_{12}$	-1.52	-2.01	-2.06	-2.02	-2.23	-2.01	-2.05	-2.66	-1.90
ethyne − aceticacid_OH − π	$C_2H_2-CH_3COOH$	-4.52	-4.99	-5.06	-5.10	-5.06	-4.99	-5.03	-5.63	-4.95
ethyne − ethyne_TS	$C_2H_2-C_2H_2$	-1.37	-1.57	-1.59	-1.56	-1.59	-1.66	-1.70	-1.90	-1.62
ethyne − pentane	$C_2H_2-C_5H_{12}$	-1.34	-1.71	-1.80	-1.79	-1.95	-1.75	-1.80	-2.25	-1.63
ethyne − water_CH − O	$C_2H_2-H_2O$	-2.73	-2.92	-2.96	-2.92	-2.87	-3.01	-3.05	-3.34	-3.00
methylamine − methylamine	$CH_3NH_2-CH_3NH_2$	-3.75	-3.94	-4.01	-4.05	-4.15	-4.11	-4.14	-4.75	-4.09
methylamine − methanol	$CH_3NH_2-CH_3OH$	-2.78	-2.94	-3.01	-2.97	-3.12	-3.14	-3.14	-3.45	-3.13
methylamine − peptide	$CH_3NH_2-C_6H_7NO_2$	-4.89	-5.37	-5.40	-5.53	-5.47	-5.40	-5.49	-6.05	-5.39
methylamine − pyridine	$CH_3NH_2-C_5H_5N$	-3.36	-3.74	-3.78	-3.89	-4.00	-4.05	-4.16	-4.40	-4.03
methylamine − water	$CH_3NH_2-H_2O$	-6.92	-6.90	-7.32	-7.24	-7.48	-7.53	-7.50	-7.97	-7.44
methanol − methylamine	$CH_3OH-CH_3NH_2$	-7.09	-7.14	-7.44	-7.32	-7.77	-7.65	-7.58	-8.04	-7.64
methanol − methanol	CH_3OH-CH_3OH	-5.45	-5.52	-5.67	-5.68	-5.72	-5.91	-5.91	-6.18	-5.89
methanol − peptide	CH_3OH-CH_3OH	-7.79	-8.07	-8.16	-8.22	-8.25	-8.24	-8.23	-8.80	-8.23
methanol − pyridine	$CH_3OH-C_5H_5N$	-6.93	-7.00	-7.25	-7.18	-7.56	-7.71	-7.66	-7.81	-7.74
methanol − water	CH_3OH-H_2O	-4.79	-5.02	-5.02	-5.02	-5.04	-5.12	-5.12	-5.56	-5.11
neopentane − neopentane	$C_5H_{12}-C_5H_{12}$	-1.31	-1.89	-1.96	-1.83	-2.35	-1.80	-1.83	-2.43	-1.73
neopentane − pentane	$C_5H_{12}-C_5H_{12}$	-1.97	-2.92	-2.87	-2.83	-3.14	-2.69	-2.73	-3.40	-2.54
pentane − acetamide	$C_5H_{12}-CH_3CONH_2$	-2.75	-3.67	-3.64	-3.66	-3.87	-3.52	-3.59	-4.22	-3.42
pentane − aceticacid	$C_5H_{12}-CH_3COOH$	-2.24	-3.01	-3.10	-3.05	-3.21	-2.96	-3.04	-3.39	-2.83
pentane − pentane	$C_5H_{12}-C_5H_{12}$	-2.87	-4.23	-4.14	-4.18	-4.37	-3.89	-3.91	-4.81	-3.67
peptide − ethene	$C_6H_7NO_2-CH_2H_4$	-2.51	-2.87	-2.98	-2.99	-3.09	-3.01	-3.07	-3.47	-2.96
peptide − methylamine	$C_6H_7NO_2-CH_3NH_2$	-6.89	-6.93	-7.31	-7.33	-7.65	-7.53	-7.58	-7.88	-7.52
peptide − methanol	$C_6H_7NO_2-CH_3OH$	-5.78	-5.88	-6.19	-6.25	-6.27	-6.35	-6.47	-6.70	-6.34
peptide − pentane	$C_6H_7NO_2-C_5H_{12}$	-3.32	-4.45	-4.39	-4.42	-4.47	-4.22	-4.34	-4.89	-4.11
peptide − peptide	$C_6H_7NO_2-C_6H_7NO_2$	-8.03	-8.29	-8.38	-8.42	-8.74	-8.66	-8.69	-8.96	-8.62
peptide − water	$C_6H_7NO_2-H_2O$	-4.89	-5.01	-5.06	-5.06	-5.08	-5.17	-5.24	-5.55	-5.15
pyridine − ethene	$C_5H_5N-C_2H_4$	-1.23	-1.89	-1.81	-1.96	-2.17	-2.05	-2.16	-2.37	-2.11
pyridine − ethyne	$C_5H_5N-C_2H_2$	-3.72	-4.02	-4.07	-4.03	-4.24	-4.42	-4.43	-4.56	-4.42
pyridine − pyridine_CH − N	$C_5H_5N-C_5H_5N$	-3.69	-3.57	-3.86	-3.79	-3.85	-4.23	-4.34	-4.60	-4.23
pyridine − pyridine_π − π	$C_5H_5N-C_5H_5N$	-2.77	-3.72	-3.86	-4.08	-4.25	-4.47	-4.70	-4.12	-4.58
pyridine − pyridine_TS	$C_5H_5N-C_5H_5N$	-2.89	-3.17	-3.41	-3.50	-3.64	-3.82	-3.99	-3.88	-3.74
pyridine − uracil_π − π	$C_5H_5N-C_4H_4N_2O_2$	-5.41	-6.64	-6.74	-7.07	-6.93	-7.24	-7.56	-6.80	-7.44
uracil − cyclopentane	$C_4H_4N_2O_2-C_5H_{10}$	-3.08	-4.25	-4.32	-4.41	-4.60	-4.40	-4.57	-4.54	-4.30
uracil − ethene	$C_4H_4N_2O_2-C_2H_4$	-2.68	-3.27	-3.37	-3.51	-3.63	-3.51	-3.64	-3.78	-3.54
uracil − ethyne	$C_4H_4N_2O_2-C_2H_2$	-3.12	-3.61	-3.66	-3.83	-3.69	-3.74	-3.88	-4.07	-3.78
uracil − neopentane	$C_4H_4N_2O_2-C_5H_{12}$	-2.87	-3.76	-3.90	-3.96	-4.18	-3.87	-4.01	-4.11	-3.68
uracil − pentane	$C_4H_4N_2O_2-C_5H_{12}$	-3.66	-5.06	-5.06	-5.17	-5.37	-5.04	-5.23	-5.29	-4.91
uracil − uracil_BP	$C_4H_4N_2O_2-C_4H_4N_2O_2$	-16.29	-15.83	-16.01	-16.10	-16.35	-16.87	-16.78	-16.84	-16.96
uracil − uracil_π − π	$C_4H_4N_2O_2-C_4H_4N_2O_2$	-8.12	-9.44	-9.39	-9.81	-9.39	-9.83	-10.09	-9.07	-10.10
water − methylamine	$H_2O-CH_3NH_2$	-6.60	-6.67	-7.02	-6.92	-7.32	-7.29	-7.20	-7.65	-7.20
water − methanol	H_2O-CH_3OH	-5.35	-5.35	-5.60	-5.58	-5.62	-5.82	-5.82	-6.19	-5.76
water − peptide	$H_2O-C_6H_7NO_2$	-7.80	-7.98	-8.13	-8.12	-8.11	-8.19	-8.18	-8.85	-8.12
water − pyridine	$H_2O-C_5H_5N$	-6.51	-6.56	-6.92	-6.83	-7.19	-7.35	-7.29	-7.57	-7.27
water − water	H_2O-H_2O	-4.75	-4.95	-5.02	-5.01	-5.04	-5.12	-5.12	-5.63	-5.09

SAPT-DFT

Table S68: Interaction energies for CCSD(T)/CBS and SAPT-DFT. For SAPT-DFT method we used the B3LYP, PBE0 and ωB97X DFA's in conjunction with the aug-cc-pVDZ basis set. Data: 66 interaction energies. All values in kcal/mol.

Dimer	Formula	CCSD(T)/CBS	SAPT-B3LYP	SAPT-PBE0	SAPT-ωB97X
acetamide − acetamide	$CH_3CONH_2-CH_3CONH_2$	-15.60	-13.26	-13.93	-13.41
acetamide − uracil	$CH_3CONH_2-C_4H_4N_2O_2$	-18.44	-15.81	-16.58	-16.14
aceticacid − aceticacid	$CH_3COOH-CH_3COOH$	-18.39	-15.00	-16.04	-15.85
aceticacid − uracil	$CH_3COOH-C_4H_4N_2O_2$	-18.73	-15.65	-16.52	-16.28
benzene − acetamide_NH − π	$C_6H_6-CH_3CONH_2$	-3.84	-3.48	-3.68	-3.24
benzene − aceticacid	$C_6H_6-CH_3COOH$	-3.10	-2.90	-2.96	-2.47
benzene − aceticacid_OH − π	$C_6H_6-CH_3COOH$	-4.12	-3.60	-3.93	-3.55
benzene − benzene_π − π	$C_6H_6-C_6H_6$	-1.76	-2.10	-1.96	-1.28
benzene − benzene_TS	$C_6H_6-C_6H_6$	-2.24	-2.11	-2.18	-1.74
benzene − cyclopentane	$C_6H_6-C_5H_{10}$	-2.67	-2.57	-2.54	-1.98
benzene − ethene	$C_6H_6-C_2H_4$	-0.83	-1.06	-0.93	-0.37
benzene − ethyne_CH − π	$C_6H_6-C_2H_2$	-2.49	-2.34	-2.54	-2.23
benzene − methylamine_NH − π	$C_6H_6-CH_3NH_2$	-2.65	-2.43	-2.52	-2.11
benzene − methanol_OH − π	$C_6H_6-CH_3OH$	-3.60	-3.32	-3.51	-2.97
benzene − neopentane	$C_6H_6-C_5H_{12}$	-2.18	-2.15	-2.14	-1.62
benzene − peptide_NH − π	$C_6H_6-C_6H_7NO_2$	-4.46	-4.00	-4.21	-3.78
benzene − pyridine_π − π	$C_6H_6-C_5H_5N$	-2.34	-2.57	-2.46	-1.86
benzene − pyridine_TS	$C_6H_6-C_5H_5N$	-2.70	-2.49	-2.59	-2.24
benzene − uracil_π − π	$C_6H_6-C_4H_4N_2O_2$	-4.28	-4.01	-3.96	-3.20
benzene − water_OH − π	$C_6H_6-H_2O$	-2.95	-2.64	-2.82	-2.51
cyclopentane − cyclopentane	$C_5H_{10}-C_5H_{10}$	-2.26	-1.96	-1.92	-1.55
cyclopentane − neopentane	$C_5H_{10}-C_5H_{12}$	-1.78	-1.57	-1.55	-1.14
ethene − pentane	$C_2H_4-C_5H_{12}$	-1.52	-1.44	-1.40	-0.97
ethyne − aceticacid_OH − π	$C_2H_2-CH_3COOH$	-4.52	-4.08	-4.32	-4.00
ethyne − ethyne_TS	$C_2H_2-C_2H_2$	-1.37	-1.43	-1.49	-1.27
ethyne − pentane	$C_2H_2-C_5H_{12}$	-1.34	-1.41	-1.36	-0.95
ethyne − water_CH − O	$C_2H_2-H_2O$	-2.73	-2.62	-2.71	-2.47
methylamine − methylamine	$CH_3NH_2-CH_3NH_2$	-3.75	-3.14	-3.28	-2.99
methylamine − methanol	$CH_3NH_2-CH_3OH$	-2.78	-2.39	-2.45	-2.14
methylamine − peptide	$CH_3NH_2-C_6H_7NO_2$	-4.89	-4.17	-4.29	-4.07
methylamine − pyridine	$CH_3NH_2-C_5H_5N$	-3.36	-3.00	-3.03	-2.69
methylamine − water	$CH_3NH_2-H_2O$	-6.92	-5.98	-6.27	-5.88
methanol − methylamine	$CH_3OH-CH_3NH_2$	-7.09	-6.10	-6.47	-5.93
methanol − methanol	CH_3OH-CH_3OH	-5.45	-4.60	-4.76	-4.43
methanol − peptide	CH_3OH-CH_3OH	-7.79	-6.61	-6.88	-6.57
methanol − pyridine	$CH_3OH-C_5H_5N$	-6.93	-6.07	-6.29	-5.94
methanol − water	CH_3OH-H_2O	-4.79	-4.24	-4.38	-4.08
neopentane − neopentane	$C_5H_{12}-C_5H_{12}$	-1.31	-1.23	-1.17	-0.83
neopentane − pentane	$C_5H_{12}-C_5H_{12}$	-1.97	-1.71	-1.67	-1.33
pentane − acetamide	$C_5H_{12}-CH_3CONH_2$	-2.75	-2.50	-2.50	-1.94
pentane − aceticacid	$C_5H_{12}-CH_3COOH$	-2.24	-1.95	-1.95	-1.56
pentane − pentane	$C_5H_{12}-C_5H_{12}$	-2.87	-2.39	-2.32	-1.98
peptide − ethene	$C_6H_7NO_2-CH_2H_4$	-2.51	-2.31	-2.34	-1.97
peptide − methylamine	$C_6H_7NO_2-CH_3NH_2$	-6.89	-5.75	-6.08	-5.72
peptide − methanol	$C_6H_7NO_2-CH_3OH$	-5.78	-4.97	-5.13	-4.79
peptide − pentane	$C_6H_7NO_2-C_5H_{12}$	-3.32	-2.72	-2.71	-2.38
peptide − peptide	$C_6H_7NO_2-C_6H_7NO_2$	-8.03	-6.98	-7.22	-6.68
peptide − water	$C_6H_7NO_2-H_2O$	-4.89	-4.39	-4.53	-4.27
pyridine − ethene	$C_5H_5N-C_2H_4$	-1.23	-1.42	-1.29	-0.76
pyridine − ethyne	$C_5H_5N-C_2H_2$	-3.72	-3.34	-3.46	-3.32
pyridine − pyridine_CH − N	$C_5H_5N-C_5H_5N$	-3.69	-3.07	-3.17	-3.09
pyridine − pyridine_π − π	$C_5H_5N-C_5H_5N$	-2.77	-2.93	-2.84	-2.35
pyridine − pyridine_TS	$C_5H_5N-C_5H_5N$	-2.89	-2.56	-2.65	-2.37
pyridine − uracil_π − π	$C_5H_5N-C_4H_4N_2O_2$	-5.41	-5.14	-5.06	-4.50
uracil − cyclopentane	$C_4H_4N_2O_2-C_5H_{10}$	-3.08	-2.61	-2.58	-2.12
uracil − ethene	$C_4H_4N_2O_2-C_2H_4$	-2.68	-2.56	-2.49	-1.94
uracil − ethyne	$C_4H_4N_2O_2-C_2H_2$	-3.12	-3.04	-2.97	-2.51
uracil − neopentane	$C_4H_4N_2O_2-C_5H_{12}$	-2.87	-2.58	-2.55	-2.03
uracil − pentane	$C_4H_4N_2O_2-C_5H_{12}$	-3.66	-3.05	-3.05	-2.56
uracil − uracil_BP	$C_4H_4N_2O_2-C_4H_4N_2O_2$	-16.29	-13.67	-14.39	-14.08
uracil − uracil_π − π	$C_4H_4N_2O_2-C_4H_4N_2O_2$	-8.12	-7.58	-7.69	-6.84
water − methylamine	$H_2O-CH_3NH_2$	-6.60	-5.69	-5.98	-5.63
water − methanol	H_2O-CH_3OH	-5.35	-4.54	-4.67	-4.39
water − peptide	$H_2O-C_6H_7NO_2$	-7.80	-6.81	-7.02	-6.55
water − pyridine	$H_2O-C_5H_5N$	-6.51	-5.72	-5.88	-5.65
water − water	H_2O-H_2O	-4.75	-4.22	-4.34	-4.08

6.2.4 Absolute deviations of molecular systems.

MP2 with C_{OS} y C_{SS} values

Table S69: Absolute deviations for MP2, SCS-MP2, SCS(MI)-MP2, SCSN-MP2, SCS-MP2-vdW, RI-MP2, RI-SCS-MP2, RIJK-MP2, RIJK-SCS-MP2, RIJCOSX-MP2, RIJCOSX-SCS-MP2, SCS-MP2^{BWI-TZ}, RI-SCS-MP2^{BWI-TZ}, RIJK-SCS-MP2^{BWI-TZ}, RIJCOSX-SCS-MP2^{BWI-DZ} and SCS-MP2-hal^{G-XZ}. Data: 66 interaction energies. MP2: C_{OS} = C_{SS} =1.00; SCS-MP2: C_{OS} =1.20, C_{SS} =0.33; SCS(MI)-MP2: C_{OS} =0.40, C_{SS} =1.29; SCSN-MP2: C_{OS} =0.00, C_{SS} =1.76; SCS-MP2-vdW: C_{OS} =1.28, C_{SS} =0.50; SCS-MP2^{BWI-TZ} RI-SCS-MP2^{BWI-TZ} , RIJK-SCS-MP2^{BWI-TZ} : C_{OS} =0.27, C_{SS} =1.38; RIJCOSX-SCS-MP2^{BWI-TZ} : C_{OS} =0.17, C_{SS} =1.59. All values in kcal/mol.

Dimer	Formula	MP2	SCS-MP2	SCS(MI)-MP2	SCSN-MP2	SCS-MP2-vdW	RI-MP2	RI-SCS-MP2	RIJK-MP2	RIJK-SCS-MP2	RIJCOSX-MP2	RIJCOSX-SCS-MP2	SCS-MP2^{BWI-TZ}	RI-SCS-MP2^{BWI-TZ}	RIJK-SCS-MP2^{BWI-TZ}	RIJCOSX-SCS-MP2^{BWI-TZ}	SCS-MP2-hal^{G-XZ}
acetamide − acetamide	CH_3CONH_2	0.18	1.28	0.55	0.21	0.56	0.18	1.28	0.18	1.28	0.13	1.33	0.62	0.62	0.62	0.64	1.66
acetamide − uracil	CH_3CONH_2	0.32	1.47	0.22	0.37	0.68	0.32	1.47	0.31	1.47	0.23	1.55	0.24	0.24	0.25	0.28	1.91
aceticacid − aceticacid	CH_3COOH	1.02	2.77	1.16	0.39	2.10	1.02	2.77	1.03	2.78	1.07	2.82	1.10	1.10	1.12	1.11	3.17
aceticacid − uracil	CH_3COOH	0.26	2.08	0.55	0.19	1.35	0.26	2.08	0.27	2.09	0.33	2.16	0.52	0.52	0.53	0.55	2.52
benzene − acetamide − NH − π	C_6H_6	1.68	0.78	0.73	0.68	1.35	1.68	0.78	1.69	0.79	1.60	0.70	0.58	0.58	0.58	0.51	0.51
benzene − aceticacid	C_6H_6	2.54	1.29	1.13	1.02	2.10	2.54	1.29	2.55	1.30	2.49	1.24	0.91	0.91	0.92	0.88	0.90
benzene − aceticacid − OH − π	C_6H_6	2.14	1.13	1.01	0.93	1.79	2.14	1.13	2.16	1.15	2.09	1.08	0.84	0.84	0.85	0.79	0.82
benzene − benzene − π − π	C_6H_6	4.14	2.44	2.06	1.82	3.59	4.14	2.44	4.17	2.47	4.10	2.40	1.72	1.72	1.74	1.70	1.90
benzene − benzene − TS	C_6H_6	2.67	1.59	1.31	1.14	2.33	2.67	1.59	2.68	1.60	2.60	1.52	1.09	1.09	1.10	1.03	1.25
benzene − cyclopentane	C_6H_6	3.31	1.81	1.42	1.19	2.84	3.31	1.81	3.32	1.81	3.25	1.75	1.11	1.11	1.11	1.07	1.32
benzene − ethene	C_6H_6	2.07	1.09	0.85	0.71	1.76	2.07	1.09	2.07	1.09	2.04	1.06	0.66	0.66	0.65	0.64	0.77
benzene − ethyne − CH − π	C_6H_6	1.81	1.06	0.98	0.92	1.54	1.81	1.06	1.82	1.07	1.76	1.01	0.85	0.85	0.86	0.81	0.82
benzene − methylamine − NH − π	C_6H_6	2.05	1.03	0.82	0.70	1.71	2.05	1.03	2.07	1.05	2.01	0.99	0.63	0.63	0.64	0.60	0.71
benzene − methanol − OH − π	C_6H_6	2.09	1.04	0.88	0.78	1.73	2.09	1.04	2.10	1.06	2.02	0.98	0.69	0.69	0.70	0.64	0.71
benzene − neopentane	C_6H_6	2.63	1.48	1.12	0.91	2.28	2.63	1.48	2.64	1.48	2.56	1.40	0.87	0.87	0.88	0.81	1.10
benzene − peptide − NH − π	C_6H_6	3.12	1.71	1.39	1.20	2.66	3.12	1.71	3.14	1.73	3.06	1.65	1.11	1.11	1.13	1.07	1.25
benzene − pyridine − π − π	C_6H_6	4.24	2.45	2.11	1.90	3.64	4.24	2.45	4.26	2.47	4.21	2.42	1.77	1.77	1.79	1.77	1.88
benzene − pyridine − TS	C_6H_6	2.56	1.47	1.26	1.13	2.20	2.56	1.47	2.58	1.49	2.51	1.42	1.05	1.05	1.07	1.01	1.13
benzene − uracil − π − π	C_6H_6	4.93	2.64	2.37	2.18	4.13	4.93	2.64	4.95	2.65	4.90	2.61	1.97	1.97	1.98	1.98	1.93
benzene − water − OH − π	C_6H_6	1.25	0.62	0.51	0.44	1.04	1.25	0.62	1.26	0.64	1.20	0.58	0.40	0.40	0.41	0.36	0.43
cyclopentane − cyclopentane	C_5H_{10}	1.95	0.78	0.50	0.32	1.58	1.95	0.78	1.96	0.80	1.84	0.67	0.26	0.26	0.27	0.17	0.41
cyclopentane − neopentane	C_5H_{10}	1.67	0.73	0.41	0.23	1.39	1.67	0.73	1.67	0.72	1.64	0.69	0.21	0.21	0.20	0.18	0.42
ethene − pentane	C_2H_4	1.23	0.42	0.26	0.16	0.96	1.23	0.42	1.23	0.42	3.79	3.50	0.10	0.10	0.10	4.00	0.17
ethyne − aceticacid − OH − π	C_2H_2	0.80	0.09	0.39	0.53	0.45	0.80	0.09	0.80	0.09	0.79	0.07	0.34	0.34	0.34	0.34	0.11
ethyne − ethyne − TS	C_2H_2	0.58	0.28	0.30	0.30	0.46	0.58	0.28	0.58	0.28	0.57	0.27	0.25	0.25	0.26	0.25	0.19
ethyne − pentane	C_2H_2	1.17	0.45	0.31	0.23	0.93	1.17	0.45	1.17	0.45	1.14	0.42	0.18	0.18	0.17	0.16	0.22
ethyne − water − CH − O	C_2H_2	0.43	0.14	0.33	0.43	0.27	0.43	0.14	0.43	0.14	0.42	0.13	0.33	0.33	0.33	0.33	0.06
methylamine − methylamine	CH_3NH_2	0.86	0.10	0.14	0.15	0.56	0.86	0.10	0.85	0.09	0.81	0.05	0.03	0.03	0.03	0.00	0.13
methylamine − meOH	CH_3NH_2	0.62	0.12	0.14	0.14	0.43	0.62	0.12	0.62	0.12	0.60	0.10	0.07	0.07	0.07	0.05	0.03
methylamine − peptide	CH_3NH_2	1.29	0.33	0.46	0.51	0.89	1.29	0.33	1.28	0.32	1.25	0.28	0.34	0.34	0.33	0.32	0.05
methylamine − pyridine	CH_3NH_2	1.80	0.73	0.66	0.59	1.41	1.80	0.73	1.79	0.72	1.75	0.68	0.48	0.48	0.47	0.44	0.40
methylamine − water	CH_3NH_2	0.83	0.07	0.22	0.28	0.50	0.83	0.07	0.83	0.07	0.77	0.01	0.14	0.14	0.14	0.10	0.15
methanol − methylamine	CH_3OH	0.82	0.09	0.14	0.24	0.42	0.82	0.09	0.81	0.10	0.77	0.14	0.05	0.05	0.04	0.02	0.35
methanol − methanol	CH_3OH	0.69	0.05	0.24	0.32	0.40	0.69	0.05	0.68	0.05	0.65	0.02	0.18	0.18	0.17	0.16	0.13
methanol − peptide	CH_3OH	0.74	0.17	0.24	0.42	0.29	0.74	0.17	0.74	0.16	0.73	0.18	0.18	0.18	0.18	0.18	0.41
methanol − pyridine	CH_3OH	1.10	0.23	0.36	0.40	0.74	1.10	0.23	1.10	0.23	1.06	0.19	0.25	0.25	0.25	0.22	0.02
methanol − water	CH_3OH	0.44	0.04	0.21	0.33	0.19	0.44	0.04	0.44	0.04	0.42	0.06	0.19	0.19	0.19	0.17	0.17
neopentane − neopentane	C_5H_{12}	1.21	0.55	0.26	0.09	1.04	1.21	0.55	1.22	0.56	1.15	0.48	0.09	0.09	0.10	0.03	0.33
neopentane − pentane	C_5H_{12}	1.68	0.67	0.38	0.21	1.37	1.68	0.67	1.68	0.67	1.59	0.58	0.16	0.16	0.16	0.10	0.34
pentane − acetamide	C_5H_{12}	1.90	0.68	0.48	0.35	1.48	1.90	0.68	1.90	0.68	1.85	0.62	0.25	0.25	0.25	0.23	0.29
pentane − aceticacid	C_5H_{12}	1.75	0.70	0.49	0.36	1.40	1.75	0.70	1.76	0.70	1.69	0.64	0.28	0.28	0.29	0.24	0.36
pentane − pentane	C_5H_{12}	2.30	0.80	0.48	0.29	1.80	2.30	0.80	2.30	0.80	2.21	0.70	0.19	0.19	0.19	0.13	0.32
peptide − ethene	$C_6H_7NO_2$	1.21	0.37	0.39	0.38	0.89	1.21	0.37	1.21	0.37	1.20	0.36	0.26	0.26	0.26	0.26	0.12
peptide − methylamine	$C_6H_7NO_2$	1.43	0.48	0.44	0.39	1.08	1.43	0.48	1.43	0.48	1.38	0.43	0.28	0.28	0.28	0.24	0.19
peptide − methanol	$C_6H_7NO_2$	1.31	0.57	0.56	0.54	1.03	1.31	0.57	1.31	0.57	1.27	0.54	0.45	0.45	0.45	0.43	0.35
peptide − pentane	$C_6H_7NO_2$	2.43	0.90	0.63	0.46	1.91	2.43	0.90	2.43	0.90	2.35	0.82	0.34	0.34	0.34	0.29	0.41
peptide − peptide	$C_6H_7NO_2$	1.32	0.19	0.39	0.47	0.84	1.32	0.19	1.32	0.19	1.26	0.13	0.26	0.26	0.26	0.22	0.14
peptide − water	$C_6H_7NO_2$	0.64	0.13	0.28	0.34	0.41	0.64	0.13	0.65	0.13	0.63	0.12	0.23	0.23	0.23	0.22	0.02
pyridine − ethene	C_5H_5N	2.15	1.09	0.90	0.78	1.79	2.15	1.09	2.15	1.09	2.13	1.07	0.70	0.70	0.70	0.70	0.75
pyridine − ethyne	C_5H_5N	1.01	0.51	0.61	0.65	0.79	1.01	0.51	1.01	0.51	0.94	0.44	0.55	0.55	0.55	0.49	0.36
pyridine − pyridine − CH − N	C_5H_5N	1.31	0.57	0.51	0.46	1.05	1.31	0.57	1.31	0.57	0.95	0.21	0.38	0.38	0.38	0.03	0.34
pyridine − pyridine − π − π	C_5H_5N	4.30	2.45	2.14	1.94	3.67	4.30	2.45	4.32	2.46	4.27	2.41	1.79	1.79	1.81	1.79	1.86
pyridine − pyridine − TS	C_5H_5N	2.41	1.28	1.15	1.05	2.02	2.41	1.28	2.42	1.29	2.33	1.20	0.94	0.94	0.95	0.88	0.93
pyridine − uracil − π − π	C_5H_5N	4.71	2.39	2.28	2.17	3.85	4.71	2.39	4.71	2.39	4.69	2.37	1.90	1.90	1.90	1.92	1.68
uracil − cyclopentane	$C_4H_4N_2O_2$	3.27	1.66	1.24	1.00	2.76	3.27	1.66	3.27	1.66	3.22	1.61	0.91	0.91	0.91	0.90	1.14
uracil − ethene	$C_4H_4N_2O_2$	2.12	0.95	0.91	0.86	1.69	2.12	0.95	2.12	0.95	2.11	0.93	0.72	0.72	0.72	0.72	0.59
uracil − ethyne	$C_4H_4N_2O_2$	1.80	0.69	0.80	0.83	1.35	1.80	0.69	1.79	0.69	1.79	0.69	0.65	0.65	0.64	0.67	0.37
uracil − neopentane	$C_4H_4N_2O_2$	2.50	1.21	0.93	0.75	2.09	2.50	1.21	2.51	1.22	2.48	1.19	0.67	0.67	0.68	0.67	0.80
uracil − pentane	$C_4H_4N_2O_2$	3.51	1.65	1.28	1.05	2.89	3.51	1.65	3.52	1.66	3.44	1.58	0.92	0.92	0.93	0.88	1.06
uracil − uracil − BP	$C_4H_4N_2O_2$	1.02	0.93	0.41	1.05	0.07	1.02	0.93	1.01	0.93	0.67	1.28	0.39	0.39	0.38	0.09	1.42
uracil − uracil − π − π	$C_4H_4N_2O_2$	4.51	1.77	1.91	1.92	3.43	4.51	1.77	4.53	1.78	4.48	1.74	1.52	1.52	1.53	1.54	0.95
water − methylamine	H_2O	0.70	0.05	0.20	0.26	0.42	0.70	0.05	0.70	0.05	0.66	0.01	0.13	0.13	0.13	0.10	0.13
water − methanol	H_2O	0.63	0.09	0.22	0.27	0.39	0.63	0.09	0.62	0.08	0.60	0.06	0.16	0.16	0.16	0.14	0.07
water − peptide	H_2O	0.51	0.29	0.15	0.35	0.10	0.51	0.29	0.51	0.29	0.48	0.32	0.11	0.11	0.11	0.10	0.49
water − pyridine	H_2O	0.94	0.29	0.34	0.35	0.68	0.94	0.29	0.94	0.29	0.89	0.24	0.25	0.25	0.24	0.21	0.10
water − water	H_2O	0.41	0.01	0.20	0.30	0.21	0.41	0.01	0.41	0.01	0.38	0.02	0.18	0.18	0.18	0.16	0.10

DFT

Table S70: Absolute deviations for B97M-V, ωB97X-V, ωB97M-V, ωB97X-D3, B2PLYP-D3BJ, DSD-BLYP-D3BJ, ωB97X-D4 and B2PLYP-D4. Data: 66 interaction energies. All values in kcal/mol.

Dimer	Formula	B97M-V	ωB97X-V	ωB97M-V	ωB97X-D3	B2PLYP-D3BJ	DSD-BLYP-D3BJ	ωB97X-D4	B2PLYP-D4
acetamide − acetamide	$CH_3CONH_2{-}CH_3CONH_2$	0.68	0.49	0.46	0.13	0.03	0.06	0.57	0.10
acetamide − uracil	$CH_3CONH_2{-}C_4H_4N_2O_2$	0.91	0.65	0.61	0.32	0.03	0.06	0.25	0.12
aceticacid − aceticacid	$CH_3COOH{-}CH_3COOH$	1.39	0.85	0.97	0.47	0.75	0.93	0.17	0.69
aceticacid − uracil	$CH_3COOH{-}C_4H_4N_2O_2$	1.18	0.81	0.81	0.44	0.32	0.45	0.12	0.24
benzene − acetamide_NH − π	$C_6H_6{-}CH_3CONH_2$	0.22	0.48	0.54	0.68	0.65	0.79	0.89	0.60
benzene − aceticacid	$C_6H_6{-}CH_3COOH$	0.58	0.86	1.01	1.04	0.94	1.13	1.13	0.85
benzene − aceticacid_OH − π	$C_6H_6{-}CH_3COOH$	0.33	0.75	0.86	0.84	0.77	0.99	0.89	0.68
benzene − benzene_π − π	$C_6H_6{-}C_6H_6$	1.06	1.13	1.35	1.64	1.64	1.85	1.50	1.76
benzene − benzene_TS	$C_6H_6{-}C_6H_6$	0.40	0.62	0.74	0.94	1.01	1.22	1.06	0.95
benzene − cyclopentane	$C_6H_6{-}C_5H_{10}$	1.01	1.08	1.23	1.53	1.30	1.48	1.52	1.21
benzene − ethene	$C_6H_6{-}C_2H_4$	0.68	0.61	0.76	1.02	0.78	0.88	1.16	0.84
benzene − ethyne_CH − π	$C_6H_6{-}C_2H_2$	0.16	0.46	0.49	0.52	0.66	0.85	0.83	0.53
benzene − methylamine_NH − π	$C_6H_6{-}CH_3NH_2$	0.40	0.56	0.68	0.93	0.74	0.89	1.01	0.65
benzene − methanol_OH − π	$C_6H_6{-}CH_3OH$	0.38	0.63	0.75	0.89	0.75	0.93	0.97	0.63
benzene − neopentane	$C_6H_6{-}C_5H_{12}$	0.76	0.93	1.01	1.40	0.98	1.15	1.38	0.87
benzene − peptide_NH − π	$C_6H_6{-}C_6H_7NO_2$	0.53	0.88	1.02	1.33	1.13	1.38	1.28	1.03
benzene − pyridine_π − π	$C_6H_6{-}C_5H_5N$	1.03	1.12	1.35	1.54	1.69	1.92	1.39	1.79
benzene − pyridine_TS	$C_6H_6{-}C_5H_5N$	0.28	0.60	0.71	0.87	0.99	1.18	1.03	0.90
benzene − uracil_π − π	$C_6H_6{-}C_4H_4N_2O_2$	1.26	1.42	1.80	1.65	1.91	2.24	1.43	2.05
benzene − water_OH − π	$C_6H_6{-}H_2O$	0.18	0.46	0.51	0.72	0.46	0.59	0.82	0.26
cyclopentane − cyclopentane	$C_5H_{10}{-}C_5H_{10}$	0.92	0.95	0.93	1.22	0.89	0.91	1.48	0.80
cyclopentane − neopentane	$C_5H_{10}{-}C_5H_{12}$	0.82	0.82	0.77	1.22	0.76	0.78	1.39	0.70
ethene − pentane	$C_2H_4{-}C_5H_{12}$	0.49	0.54	0.49	0.71	0.48	0.52	1.14	0.38
ethyne − aceticacid_OH − π	$C_2H_2{-}CH_3COOH$	0.47	0.54	0.58	0.55	0.48	0.51	1.12	0.43
ethyne − ethyne_TS	$C_2H_2{-}C_2H_2$	0.20	0.22	0.19	0.21	0.29	0.32	0.53	0.25
ethyne − pentane	$C_2H_2{-}C_5H_{12}$	0.37	0.46	0.44	0.61	0.40	0.46	0.91	0.29
ethyne − water_CH − O	$C_2H_2{-}H_2O$	0.19	0.23	0.19	0.14	0.28	0.32	0.61	0.27
methylamine − methylamine	$CH_3NH_2{-}CH_3NH_2$	0.19	0.26	0.30	0.40	0.36	0.39	1.00	0.34
methylamine − methanol	$CH_3NH_2{-}CH_3OH$	0.16	0.24	0.19	0.34	0.36	0.36	0.68	0.35
methylamine − peptide	$CH_3NH_2{-}C_6H_7NO_2$	0.48	0.51	0.64	0.58	0.51	0.60	1.16	0.50
methylamine − pyridine	$CH_3NH_2{-}C_5H_5N$	0.38	0.42	0.53	0.64	0.69	0.80	1.04	0.67
methylamine − water	$CH_3NH_2{-}H_2O$	0.02	0.41	0.33	0.56	0.62	0.59	1.06	0.52
methanol − methylamine	$CH_3OH{-}CH_3NH_2$	0.05	0.35	0.23	0.68	0.56	0.49	0.95	0.55
methanol − methanol	$CH_3OH{-}CH_3OH$	0.07	0.22	0.23	0.27	0.46	0.46	0.73	0.45
methanol − peptide	$CH_3OH{-}CH_3OH$	0.28	0.37	0.43	0.46	0.45	0.45	1.01	0.44
methanol − pyridine	$CH_3OH{-}C_5H_5N$	0.07	0.32	0.25	0.63	0.79	0.73	0.88	0.81
methanol − water	$CH_3OH{-}H_2O$	0.23	0.23	0.23	0.25	0.33	0.33	0.77	0.32
neopentane − neopentane	$C_5H_{12}{-}C_5H_{12}$	0.58	0.65	0.52	1.04	0.49	0.52	1.12	0.42
neopentane − pentane	$C_5H_{12}{-}C_5H_{12}$	0.95	0.90	0.86	1.17	0.72	0.76	1.43	0.57
pentane − acetamide	$C_5H_{12}{-}CH_3CONH_2$	0.92	0.89	0.91	1.12	0.77	0.84	1.47	0.67
pentane − aceticacid	$C_5H_{12}{-}CH_3COOH$	0.77	0.86	0.81	0.97	0.72	0.80	1.15	0.59
pentane − pentane	$C_5H_{12}{-}C_5H_{12}$	1.36	1.27	1.31	1.50	1.03	1.05	1.94	0.80
peptide − ethene	$C_6H_7NO_2{-}CH_2H_4$	0.36	0.47	0.49	0.58	0.50	0.56	0.96	0.46
peptide − methylamine	$C_6H_7NO_2{-}CH_3NH_2$	0.04	0.43	0.44	0.76	0.64	0.70	0.99	0.63
peptide − methanol	$C_6H_7NO_2{-}CH_3OH$	0.10	0.41	0.47	0.49	0.57	0.69	0.93	0.56
peptide − pentane	$C_6H_7NO_2{-}C_5H_{12}$	1.13	1.07	1.10	1.15	0.90	1.02	1.57	0.79
peptide − peptide	$C_6H_7NO_2{-}C_6H_7NO_2$	0.26	0.35	0.39	0.71	0.63	0.66	0.93	0.59
peptide − water	$C_6H_7NO_2{-}H_2O$	0.12	0.18	0.17	0.20	0.29	0.36	0.67	0.27
pyridine − ethene	$C_5H_5N{-}C_2H_4$	0.65	0.57	0.73	0.94	0.82	0.92	1.13	0.87
pyridine − ethyne	$C_5H_5N{-}C_2H_2$	0.30	0.35	0.31	0.52	0.70	0.71	0.84	0.70
pyridine − pyridine_CH − N	$C_5H_5N{-}C_5H_5N$	0.11	0.17	0.10	0.16	0.54	0.66	0.91	0.55
pyridine − pyridine_π − π	$C_5H_5N{-}C_5H_5N$	0.95	1.09	1.31	1.48	1.70	1.93	1.35	1.81
pyridine − pyridine_TS	$C_5H_5N{-}C_5H_5N$	0.28	0.53	0.62	0.75	0.93	1.11	0.99	0.85
pyridine − uracil_π − π	$C_5H_5N{-}C_4H_4N_2O_2$	1.23	1.34	1.66	1.52	1.84	2.15	1.40	2.03
uracil − cyclopentane	$C_4H_4N_2O_2{-}C_5H_{10}$	1.17	1.24	1.33	1.52	1.32	1.50	1.47	1.22
uracil − ethene	$C_4H_4N_2O_2{-}C_2H_4$	0.59	0.68	0.83	0.95	0.83	0.96	1.10	0.86
uracil − ethyne	$C_4H_4N_2O_2{-}C_2H_2$	0.49	0.54	0.71	0.57	0.62	0.76	0.95	0.66
uracil − neopentane	$C_4H_4N_2O_2{-}C_5H_{12}$	0.89	1.04	1.09	1.31	1.00	1.14	1.24	0.81
uracil − pentane	$C_4H_4N_2O_2{-}C_5H_{12}$	1.40	1.41	1.51	1.72	1.39	1.58	1.63	1.25
uracil − uracil_BP	$C_4H_4N_2O_2{-}C_4H_4N_2O_2$	0.45	0.27	0.19	0.06	0.58	0.49	0.55	0.67
uracil − uracil_π − π	$C_4H_4N_2O_2{-}C_4H_4N_2O_2$	1.32	1.27	1.69	1.27	1.71	1.97	0.95	1.98
water − methylamine	$H_2O{-}CH_3NH_2$	0.08	0.42	0.33	0.72	0.70	0.61	1.06	0.61
water − methanol	$H_2O{-}CH_3OH$	0.00	0.24	0.23	0.27	0.47	0.47	0.84	0.41
water − peptide	$H_2O{-}C_6H_7NO_2$	0.18	0.33	0.32	0.31	0.39	0.38	1.05	0.32
water − pyridine	$H_2O{-}C_5H_5N$	0.05	0.41	0.32	0.68	0.84	0.78	1.06	0.77
water − water	$H_2O{-}H_2O$	0.19	0.27	0.26	0.29	0.37	0.36	0.88	0.34

SAPT-DFT

Table S71: Absolute deviations for SAPT-DFT. For SAPT-DFT method we used the B3LYP, PBE0 and ωB97X DFA's in conjunction with the aug-cc-pVTZ basis set. Data: 66 interaction energies. All values in kcal/mol.

Dimer	Formula	SAPT-B3LYP	SAPT-PBE0	SAPT-ωB97X
acetamide − acetamide	$CH_3CONH_2-CH_3CONH_2$	2.34	1.67	2.19
acetamide − uracil	$CH_3CONH_2-C_4H_4N_2O_2$	2.63	1.86	2.30
aceticacid − aceticacid	$CH_3COOH-CH_3COOH$	3.39	2.35	2.54
aceticacid − uracil	$CH_3COOH-C_4H_4N_2O_2$	3.08	2.21	2.45
benzene − acetamide_NH − π	$C_6H_6-CH_3CONH_2$	0.36	0.16	0.60
benzene − aceticacid	$C_6H_6-CH_3COOH$	0.19	0.14	0.62
benzene − aceticacid_OH − π	$C_6H_6-CH_3COOH$	0.52	0.20	0.58
benzene − benzene_π − π	$C_6H_6-C_6H_6$	0.34	0.20	0.49
benzene − benzene_TS	$C_6H_6-C_6H_6$	0.12	0.06	0.50
benzene − cyclopentane	$C_6H_6-C_5H_{10}$	0.10	0.13	0.69
benzene − ethene	$C_6H_6-C_2H_4$	0.24	0.10	0.46
benzene − ethyne_CH − π	$C_6H_6-C_2H_2$	0.15	0.04	0.27
benzene − methylamine_NH − π	$C_6H_6-CH_3NH_2$	0.22	0.14	0.55
benzene − methanol_OH − π	$C_6H_6-CH_3OH$	0.28	0.09	0.64
benzene − neopentane	$C_6H_6-C_5H_{12}$	0.03	0.04	0.56
benzene − peptide_NH − π	$C_6H_6-C_6H_7NO_2$	0.46	0.25	0.68
benzene − pyridine_π − π	$C_6H_6-C_5H_5N$	0.23	0.12	0.48
benzene − pyridine_TS	$C_6H_6-C_5H_5N$	0.21	0.11	0.46
benzene − uracil_π − π	$C_6H_6-C_4H_4N_2O_2$	0.27	0.32	1.09
benzene − water_OH − π	$C_6H_6-H_2O$	0.31	0.13	0.44
cyclopentane − cyclopentane	$C_5H_{10}-C_5H_{10}$	0.30	0.34	0.71
cyclopentane − neopentane	$C_5H_{10}-C_5H_{12}$	0.21	0.24	0.64
ethene − pentane	$C_2H_4-C_5H_{12}$	0.09	0.12	0.56
ethyne − aceticacid_OH − π	$C_2H_2-CH_3COOH$	0.44	0.20	0.52
ethyne − ethyne_TS	$C_2H_2-C_2H_2$	0.06	0.12	0.10
ethyne − pentane	$C_2H_2-C_5H_{12}$	0.07	0.02	0.39
ethyne − water_CH − O	$C_2H_2-H_2O$	0.11	0.02	0.26
methylamine − methylamine	$CH_3NH_2-CH_3NH_2$	0.61	0.47	0.76
methylamine − methanol	$CH_3NH_2-CH_3OH$	0.39	0.33	0.64
methylamine − peptide	$CH_3NH_2-C_6H_7NO_2$	0.72	0.60	0.82
methylamine − pyridine	$CH_3NH_2-C_5H_5N$	0.37	0.33	0.67
methylamine − water	$CH_3NH_2-H_2O$	0.93	0.64	1.03
methanol − methylamine	$CH_3OH-CH_3NH_2$	0.98	0.62	1.16
methanol − methanol	CH_3OH-CH_3OH	0.85	0.69	1.02
methanol − peptide	CH_3OH-CH_3OH	1.18	0.91	1.22
methanol − pyridine	$CH_3OH-C_5H_5N$	0.86	0.64	0.99
methanol − water	CH_3OH-H_2O	0.55	0.41	0.71
neopentane − neopentane	$C_5H_{12}-C_5H_{12}$	0.09	0.14	0.48
neopentane − pentane	$C_5H_{12}-C_5H_{12}$	0.26	0.30	0.64
pentane − acetamide	$C_5H_{12}-CH_3CONH_2$	0.25	0.25	0.81
pentane − aceticacid	$C_5H_{12}-CH_3COOH$	0.29	0.29	0.68
pentane − pentane	$C_5H_{12}-C_5H_{12}$	0.47	0.55	0.88
peptide − ethene	$C_6H_7NO_2-CH_2H_4$	0.20	0.17	0.54
peptide − methylamine	$C_6H_7NO_2-CH_3NH_2$	1.14	0.81	1.17
peptide − methanol	$C_6H_7NO_2-CH_3OH$	0.81	0.65	0.99
peptide − pentane	$C_6H_7NO_2-C_5H_{12}$	0.60	0.61	0.94
peptide − peptide	$C_6H_7NO_2-C_6H_7NO_2$	1.05	0.81	1.35
peptide − water	$C_6H_7NO_2-H_2O$	0.49	0.36	0.62
pyridine − ethene	$C_5H_5N-C_2H_4$	0.19	0.06	0.47
pyridine − ethyne	$C_5H_5N-C_2H_2$	0.38	0.26	0.40
pyridine − pyridine_CH − N	$C_5H_5N-C_5H_5N$	0.62	0.51	0.60
pyridine − pyridine_π − π	$C_5H_5N-C_5H_5N$	0.16	0.07	0.42
pyridine − pyridine_TS	$C_5H_5N-C_5H_5N$	0.33	0.24	0.52
pyridine − uracil_π − π	$C_5H_5N-C_4H_4N_2O_2$	0.26	0.35	0.91
uracil − cyclopentane	$C_4H_4N_2O_2-C_5H_{10}$	0.47	0.49	0.96
uracil − ethene	$C_4H_4N_2O_2-C_2H_4$	0.12	0.20	0.74
uracil − ethyne	$C_4H_4N_2O_2-C_2H_2$	0.08	0.15	0.61
uracil − neopentane	$C_4H_4N_2O_2-C_5H_{12}$	0.29	0.32	0.83
uracil − pentane	$C_4H_4N_2O_2-C_5H_{12}$	0.60	0.60	1.10
uracil − uracil_BP	$C_4H_4N_2O_2-C_4H_4N_2O_2$	2.61	1.90	2.21
uracil − uracil_π − π	$C_4H_4N_2O_2-C_4H_4N_2O_2$	0.54	0.43	1.29
water − methylamine	$H_2O-CH_3NH_2$	0.90	0.61	0.96
water − methanol	H_2O-CH_3OH	0.81	0.69	0.96
water − peptide	$H_2O-C_6H_7NO_2$	0.99	0.78	1.25
water − pyridine	$H_2O-C_5H_5N$	0.79	0.63	0.86
water − water	H_2O-H_2O	0.53	0.41	0.68

6.3 Mean calculation times

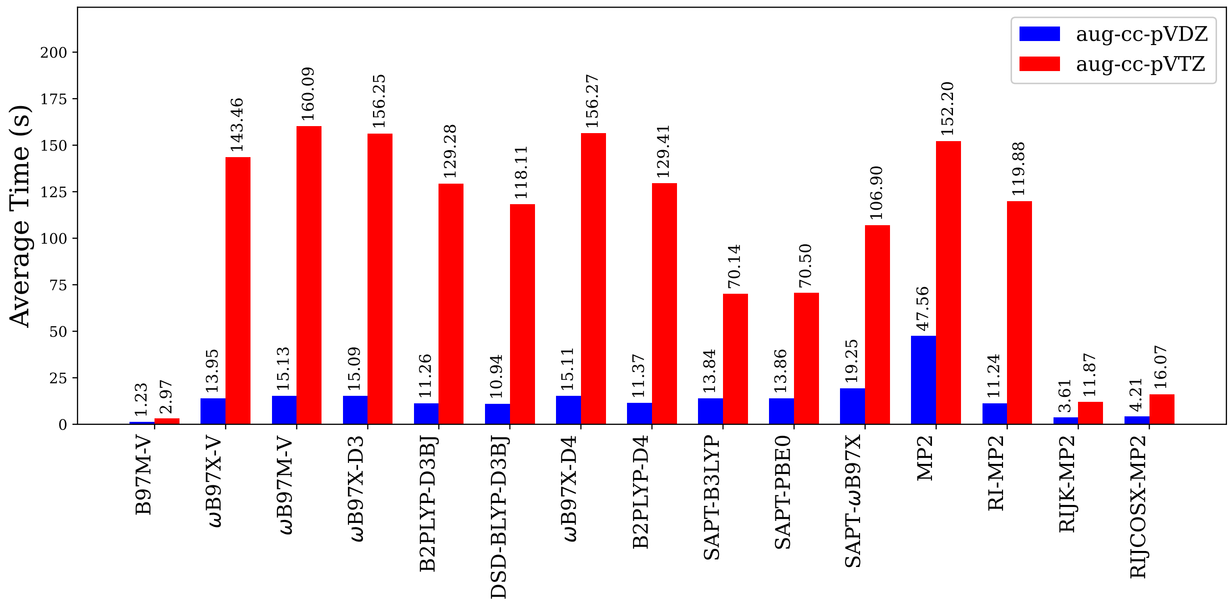

Figure S15: Comparison of computational times for different methods and basis sets in the dataset S66. All values in seconds.

7 X40* dataset

7.1 aug-cc-pVDZ basis set data.

7.1.1 Ionization Potentials and HOMO values for SAPT-DFT calculations

Table S72: Experimental ionization potentials (IP) of molecules comprising dimers in the X40* database. IP obtained from NIST (https://webbook.nist.gov/chemistry). Kohn-Sham energies of HOMO orbitals were calculated at the DFA/aug-cc-pVDZ level of theory (DFA=B3LYP, PBE0, and ωB97X). Ionization energies in ev and HOMO energies in atomic units.

			HOMO energies (eV)			$\Delta_{XC} = \epsilon_{HOMO} - (-IP)$		
Molecule	Formula	IP	B3LYP	PBE0	ωB97X	B3LYP	PBE0	ωB97X
acetone	C_3H_6O	9.69	-6.91	-7.23	-9.70	0.10	0.09	0.00
benzene	C_6H_6	9.24	-6.98	-7.29	-9.48	0.08	0.07	0.01
chlorobenzenezene	C_6H_5Cl	9.07	-6.86	-7.17	-9.34	0.08	0.07	0.01
chloromethane	CH_3Cl	11.28	-8.14	-8.50	-10.99	0.12	0.10	0.01
chlorine	Cl_2	11.48	-8.41	-8.78	-11.26	0.11	0.10	0.01
fluorine	F_2	15.70	-11.38	-11.79	-14.39	0.16	0.14	0.05
fluoromethane	CH_3F	12.54	-9.55	-9.89	-12.42	0.11	0.10	0.00
formaldehyde	CH_2O	10.88	-7.55	-7.85	-10.33	0.12	0.11	0.02
hydrogenchloride	HCl	12.79	-9.13	-9.50	-12.05	0.13	0.12	0.03
hexafluorobenzene	C_6F_6	9.90	-7.70	-7.99	-10.21	0.08	0.07	0.01
hydrogenfluoride	HF	16.06	-11.44	-11.84	-14.47	0.17	0.15	0.06
methane	CH_4	12.61	-10.67	-10.98	-9.26	0.07	0.06	0.12
methanethiol	CH_3SH	9.45	-6.51	-6.82	-13.58	0.11	0.10	0.15
methanol	CH_3OH	10.85	-7.65	-7.97	-10.50	0.12	0.11	0.01
methylamine	CH_3NH_2	8.90	-6.62	-6.93	-9.45	0.08	0.07	0.02
trichloromethane	$CHCl_3$	11.30	-8.55	-8.93	-11.40	0.10	0.09	0.00
tricholomethanol	CCl3OH	11.45	-8.54	-8.91	-11.40	0.11	0.09	0.00
trifluorobenzene	$C_6H_3F_3$	9.50	-7.41	-7.72	-9.93	0.08	0.07	0.02
trifluorochloromethane	CF_3Cl	13.08	-9.73	-10.11	-12.66	0.12	0.11	0.02
trifluoromethane	CHF_3	13.90	-11.10	-11.42	-13.98	0.10	0.09	0.00
trifluoromethanol	CF_3OH	13.08	-10.21	-10.59	-13.18	0.11	0.09	0.00
trimethylamine	$N(CH_3)_3$	7.80	-5.98	-6.27	-8.67	0.07	0.06	0.03

7.1.2 Evaluation of mean absolute deviation in a grid of C_{OS} and C_{SS} values

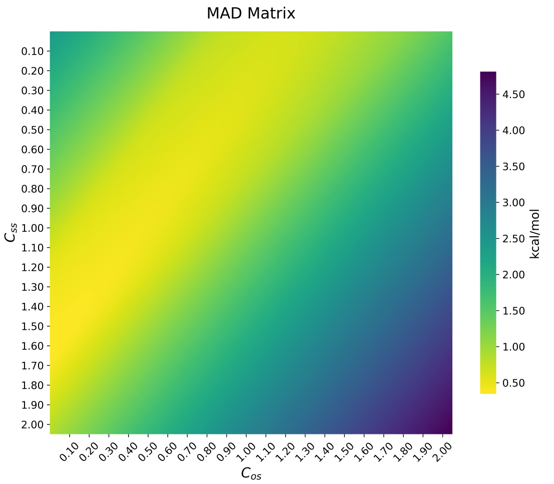

(a)

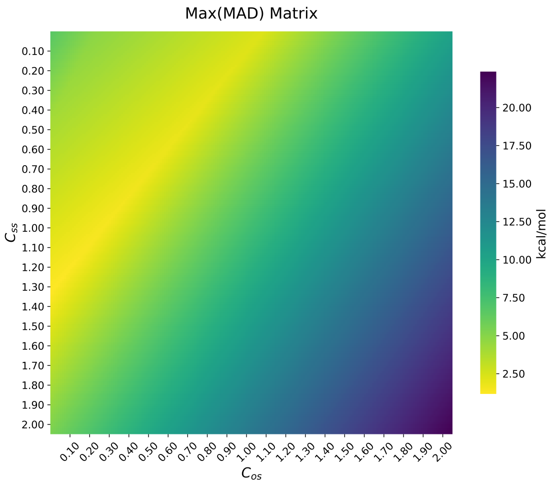

(b)

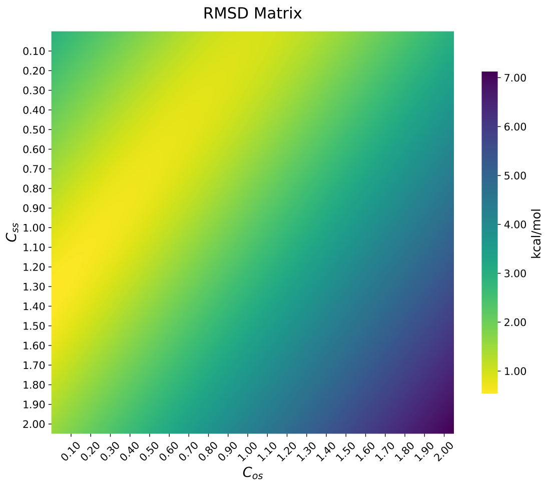

(c)

Figure S16: Evaluation of MADs (S16a), Max(MADs) (S16b) and RMSDs (S16c) in grid of values C_{OS} and C_{SS}. The optimal coefficients are C_{OS}=0.00 and C_{SS}=1.50. The theory level used is RIJK-MP2/aug-cc-pVDZ.

7.1.3 Interaction energies

MP2 with C_{OS} y C_{SS} values

Table S73: Interaction energies for CCSD(T)/CBS, MP2, SCS-MP2, SCS(MI)-MP2, SCSN-MP2, SCS-MP2-vdW, RI-MP2, RI-SCS-MP2, RIJK-MP2, RIJK-SCS-MP2, RIJCOSX-MP2, RIJCOSX-SCS-MP2, SCS-MP2^{BWI-DZ}, RI-SCS-MP2^{BWI-DZ}, RIJK-SCS-MP2^{BWI-DZ}, RIJCOSX-SCS-MP2^{BWI-DZ} and SCS-MP2-hal^{G-XZ}. Data: 22 interaction energies. MP2: $C_{OS} = C_{SS}$ =1.00; SCS-MP2: C_{OS} =1.20, C_{SS} =0.33; SCS(MI)-MP2: C_{OS} =0.40, C_{SS} =1.29; SCSN-MP2: C_{OS} =0.00, C_{SS} =1.76; SCS-MP2-vdW: C_{OS} =1.28, C_{SS} =0.50; SCS-MP2^{BWI-DZ} RI-SCS-MP2^{BWI-DZ} , RIJK-SCS-MP2^{BWI-DZ} : C_{OS} =0.00, C_{SS} =1.50; RIJCOSX-SCS-MP2^{BWI-DZ} : C_{OS} =0.00, C_{SS} =0.17. All values in kcal/mol.

Dimer	Formula	CCSD(T)/CBS	MP2	SCS-MP2	SCS(MI)-MP2	SCSN-MP2	SCS-MP2-vdW	RI-MP2	RI-SCS-MP2	RIJK-MP2	RIJK-SCS-MP2	RIJCOSX-MP2	RIJCOSX-SCS-MP2	SCS-MP2^{BWI-DZ}	RI-SCS-MP2^{BWI-DZ}	RIJK-SCS-MP2^{BWI-DZ}	RIJCOSX-SCS-MP2^{BWI-DZ}	SCS-MP2-hal^{G-XZ}
chlorobenzene − acetone	C_6H_5Cl	-1.49	-2.24	-1.74	-1.59	-1.51	-2.09	-2.25	-1.74	-2.24	-1.74	-2.25	-1.74	-1.18	-1.19	-1.19	-1.20	-1.32
chlorobenzene − trimethylamine	C_6H_5Cl	-2.11	-3.46	-2.76	-2.55	-2.43	-3.24	-3.46	-2.76	-3.46	-2.75	-3.44	-2.74	-1.97	-1.98	-1.98	-1.98	-2.16
chloromethane − chloromethane	CH_3Cl	-1.34	-1.78	-1.48	-1.41	-1.38	-1.68	-1.78	-1.48	-1.77	-1.47	-1.77	-1.47	-1.19	-1.19	-1.18	-1.19	-1.25
chloromethane − formaldehyde	CH_3Cl	-1.17	-1.43	-1.09	-1.03	-0.99	-1.32	-1.43	-1.09	-1.42	-1.08	-1.43	-1.09	-0.77	-0.78	-0.77	-0.78	-0.84
chloromethane − methane	CH_3Cl	-0.98	-1.62	-1.30	-1.14	-1.04	-1.54	-1.62	-1.30	-1.62	-1.30	-1.61	-1.29	-0.83	-0.83	-0.83	-0.83	-0.97
fluoromethane − fluoromethane	CH_3F	-1.65	-2.05	-1.88	-1.85	-1.84	-1.99	-2.06	-1.88	-2.05	-1.87	-2.04	-1.87	-1.73	-1.73	-1.72	-1.74	-1.75
fluoromethane − methane	CH_3F	-0.75	-1.29	-1.07	-0.91	-0.83	-1.25	-1.29	-1.07	-1.29	-1.06	-1.28	-1.06	-0.67	-0.67	-0.67	-0.67	-0.80
hydrogenchloride − metahnol	HCl	-6.30	-7.14	-6.34	-6.81	-7.02	-6.71	-7.14	-6.34	-7.13	-6.34	-7.11	-6.31	-6.61	-6.61	-6.60	-6.59	-6.29
hydrogenchloride − methylamine	HCl	-11.42	-12.20	-10.59	-11.36	-11.71	-11.39	-12.20	-10.59	-12.20	-10.59	-12.17	-10.56	-10.86	-10.85	-10.85	-10.85	-10.35
hexafuorobenzene − benzene	C_6F_6	-6.12	-13.66	-11.34	-10.40	-9.88	-13.01	-13.67	-11.35	-13.68	-11.37	-13.63	-11.31	-8.33	-8.34	-8.36	-8.36	-9.16
hydrogenfluoride − methanol	HF	-9.59	-9.72	-9.15	-9.58	-9.78	-9.39	-9.71	-9.15	-9.71	-9.15	-9.69	-9.13	-9.51	-9.50	-9.49	-9.48	-9.20
hydrogenfluoride − methylamine	HF	-14.32	-14.54	-13.63	-13.97	-14.12	-14.11	-14.53	-13.63	-14.52	-13.62	-14.51	-13.61	-13.62	-13.62	-13.61	-13.61	-13.41
methane − chlorine	CH_4	-1.08	-1.65	-1.30	-1.21	-1.15	-1.54	-1.65	-1.30	-1.64	-1.29	-1.66	-1.30	-0.92	-0.93	-0.92	-0.94	-1.01
methane − fluorine	CH_4	-0.49	-0.85	-0.69	-0.57	-0.51	-0.82	-0.85	-0.69	-0.85	-0.68	-0.85	-0.68	-0.39	-0.39	-0.39	-0.39	-0.49
methanol − chloromethane	CH_3OH	-3.78	-4.54	-3.99	-3.97	-3.95	-4.34	-4.54	-3.98	-4.54	-3.98	-4.53	-3.97	-3.61	-3.61	-3.60	-3.61	-3.65
methanol − fluoromethane	CH_3OH	-3.89	-4.45	-4.04	-4.11	-4.13	-4.28	-4.45	-4.04	-4.44	-4.03	-4.45	-4.04	-3.89	-3.89	-3.88	-3.89	-3.86
trichloromethane − methane	$CHCl_3$	-1.15	-1.98	-1.55	-1.37	-1.27	-1.86	-1.98	-1.55	-1.97	-1.54	-1.97	-1.53	-0.98	-0.99	-0.98	-0.99	-1.14
trichloromethanol − water	CCl_3OH	-10.41	-11.00	-9.99	-10.45	-10.66	-10.50	-11.00	-9.99	-10.99	-9.98	-10.98	-9.97	-10.12	-10.12	-10.10	-10.11	-9.81
trifluorobenzene − benzene	$C_6H_3F_3$	-4.41	-10.58	-8.53	-7.72	-7.26	-10.00	-10.59	-8.54	-10.60	-8.55	-10.58	-8.53	-5.90	-5.91	-5.92	-5.96	-6.61
trifluorochloromethane − formaldehyde	CF_3Cl	-2.25	-2.65	-2.27	-2.27	-2.27	-2.50	-2.65	-2.27	-2.64	-2.26	-2.65	-2.27	-2.04	-2.05	-2.03	-2.05	-2.05
trifluoromethane − methane	CHF_3	-0.69	-1.34	-1.13	-0.98	-0.90	-1.30	-1.35	-1.13	-1.34	-1.13	-1.34	-1.13	-0.75	-0.75	-0.74	-0.75	-0.87
trifluoromethanol − water	CF_3OH	-9.67	-10.14	-9.40	-9.79	-9.96	-9.76	-10.14	-9.40	-10.13	-9.40	-10.12	-9.39	-9.58	-9.57	-9.56	-9.56	-9.32

DFT

Table S74: Interaction energies for CCSD(T)/CBS B97M-V, ωB97X-V, ωB97M-V, ωB97X-D3, B2PLYP-D3BJ, DSD-BLYP-D3BJ, ωB97X-D4 and B2PLYP-D4. Data: 22 interaction energies. All values in kcal/mol.

Dimer	Formula	CCSD(T)/CBS	B97M-V	ωB97X-V	ωB97M-V	ωB97X-D3	B2PLYP-D3BJ	DSD-BLYP-D3BJ	ωB97X-D4	B2PLYP-D4
chlorobenzene − acetone	$C_6H_5Cl-C_3H_6O$	-1.49	-1.65	-1.56	-1.63	-1.40	-1.66	-1.77	-1.93	-1.72
chlorobenzene − trimethylamine	$C_6H_5Cl-N(CH_3)_3$	-2.11	-2.41	-2.35	-2.56	-2.40	-2.73	-2.81	-2.70	-2.78
chloromethane − chloromethane	CH_3Cl-CH_3Cl	-1.34	-1.34	-1.40	-1.33	-1.37	-1.45	-1.52	-1.71	-1.40
chloromethane − formaldehyde	CH_3Cl-CH_2O	-1.17	-1.29	-1.27	-1.30	-1.01	-1.25	-1.29	-1.65	-1.22
chloromethane − methane	CH_3Cl-CH_4	-0.98	-1.14	-1.33	-1.32	-1.46	-1.32	-1.34	-1.70	-1.25
fluoromethane − fluoromethane	CH_3F-CH_3F	-1.65	-1.72	-1.90	-1.85	-1.58	-1.86	-1.91	-1.91	-1.92
fluoromethane − methane	CH_3F-CH_4	-0.75	-0.97	-1.12	-1.07	-1.10	-1.09	-1.11	-1.32	-1.06
hydrogenchloride − methanol	$HCl-CH_3OH$	-6.30	-6.57	-6.76	-6.72	-6.98	-6.99	-6.94	-7.46	-6.92
hydrogenchloride − methylamine	$HCl-CH_3NH_2$	-11.42	-11.35	-11.70	-11.53	-12.24	-12.22	-11.93	-12.28	-12.08
hexafluorobenzene − benzene	$C_6F_6-C_6H_6$	-6.12	-7.32	-7.80	-8.32	-7.36	-8.74	-9.56	-6.75	-8.81
hydrogenfluoride − methanol	$HF-CH_3OH$	-9.59	-9.28	-9.81	-9.78	-9.86	-10.02	-9.92	-10.45	-9.96
hydrogenfluoride − methylamine	$HF-CH_3NH_2$	-14.32	-13.78	-14.83	-14.54	-14.90	-14.98	-14.72	-15.27	-14.88
methane − chlorine	CH_4-Cl_2	-1.08	-1.35	-1.25	-1.25	-1.24	-1.32	-1.38	-1.66	-1.21
methane − fluorine	CH_4-F_2	-0.49	-0.68	-0.55	-0.55	-0.30	-0.62	-0.67	-0.51	-0.58
methanol − chloromethane	CH_3OH-CH_3Cl	-3.78	-3.96	-3.99	-4.04	-3.83	-4.11	-4.19	-4.39	-4.08
methanol − fluoromethane	CH_3OH-CH_3F	-3.89	-4.03	-4.16	-4.17	-3.87	-4.25	-4.29	-4.48	-4.27
trichloromethane − methane	$CHCl_3-CH_4$	-1.15	-1.45	-1.59	-1.54	-1.82	-1.44	-1.50	-1.93	-1.37
trichloromethanol − water	CCl_3OH-H_2O	-10.41	-10.76	-10.55	-10.67	-10.96	-10.87	-10.76	-11.27	-10.84
trifluorobenzene − benzene	$C_6H_3F_3-C_6H_6$	-4.41	-5.37	-5.71	-6.23	-5.75	-6.41	-7.06	-5.32	-6.53
trifluorochloromethane − formaldehyde	CF_3Cl-CH_2O	-2.25	-2.47	-2.41	-2.50	-2.06	-2.46	-2.53	-2.82	-2.43
trifluoromethane − methane	CHF_3-CH_4	-0.69	-1.01	-1.11	-1.00	-0.91	-1.01	-1.07	-0.99	-1.04
trifluoromethanol − water	CF_3OH-H_2O	-9.67	-9.66	-9.95	-9.97	-10.00	-10.07	-10.03	-10.54	-10.09

SAPT-DFT

Table S75: Interaction energies for CCSD(T)/CBS SAPT-DFT. For SAPT-DFT method we used the B3LYP, PBE0 and ωB97X DFA's in conjunction with the aug-cc-pVDZ basis set. Data: 22 interaction energies. All values in kcal/mol.

Dimer	Formula	CCSD(T)/CBS	SAPT-B3LYP	SAPT-PBE0	SAPT-ωB97X
chlorobenzene − acetone	$C_6H_5Cl-C_3H_6O$	-1.49	-0.64	-0.66	-0.29
chlorobenzene − trimethylamine	$C_6H_5Cl-N(CH_3)_3$	-2.11	-0.76	-0.84	-0.56
chloromethane − chloromethane	CH_3Cl-CH_3Cl	-1.34	-0.92	-0.91	-0.69
chloromethane − formaldehyde	CH_3Cl-CH_2O	-1.17	-0.58	-0.59	-0.30
chloromethane − methane	CH_3Cl-CH_4	-0.98	-0.51	-0.49	-0.47
fluoromethane − fluoromethane	CH_3F-CH_3F	-1.65	-1.16	-1.12	-1.01
fluoromethane − methane	CH_3F-CH_4	-0.75	-0.36	-0.34	-0.34
hydrogenchloride − methanol	$HCl-CH_3OH$	-6.30	-4.78	-5.14	-4.91
hydrogenchloride − methylamine	$HCl-CH_3NH_2$	-11.42	-8.84	-10.06	-9.27
hexafluorobenzene − benzene	$C_6F_6-C_6H_6$	-6.12	-4.03	-4.04	-3.30
hydrogenfluoride − methanol	$HF-CH_3OH$	-9.59	-7.51	-7.71	-7.70
hydrogenfluoride − methylamine	$HF-CH_3NH_2$	-14.32	-11.43	-12.16	-11.60
methane − chlorine	CH_4-Cl_2	-1.08	-0.46	-0.48	-0.51
methane − fluorine	CH_4-F_2	-0.49	-0.12	-0.13	-0.20
methanol − chloromethane	CH_3OH-CH_3Cl	-3.78	-2.52	-2.59	-2.72
methanol − fluoromethane	CH_3OH-CH_3F	-3.89	-2.56	-2.65	-2.85
trichloromethane − methane	$CHCl_3-CH_4$	-1.15	-0.57	-0.53	-0.47
trichloromethanol − water	CCl_3OH-H_2O	-10.41	-7.90	-8.38	-7.96
trifluorobenzene − benzene	$C_6H_3F_3-C_6H_6$	-4.41	-2.76	-2.72	-2.04
trifluorochloromethane − formaldehyde	CF_3Cl-CH_2O	-2.25	-1.35	-1.37	-1.11
trifluoromethane − methane	CHF_3-CH_4	-0.69	-0.25	-0.24	-0.26
trifluoromethanol − water	CF_3OH-H_2O	-9.67	-7.56	-7.87	-7.61

7.1.4 Absolute deviations of molecular systems.

MP2 with C_{OS} y C_{SS} values

Table S76: Absolute deviations for MP2, SCS-MP2, SCS(MI)-MP2, SCSN-MP2, SCS-MP2-vdW, RI-MP2, RI-SCS-MP2, RIJK-MP2, RIJK-SCS-MP2, RIJCOSX-MP2, RIJCOSX-SCS-MP2, SCS-MP2^{BWI-DZ}, RI-SCS-MP2^{BWI-DZ}, RIJK-SCS-MP2^{BWI-DZ}, RIJCOSX-SCS-MP2^{BWI-DZ} and SCS-MP2-hal^{G-XZ}. Data: 22 interaction energies. MP2: C_{OS} = C_{SS} =1.00; SCS-MP2: C_{OS} =1.20, C_{SS} =0.33; SCS(MI)-MP2: C_{OS} =0.40, C_{SS} =1.29; SCSN-MP2: C_{OS} =0.00, C_{SS} =1.76; SCS-MP2-vdW: C_{OS} =1.28, C_{SS} =0.50; SCS-MP2^{BWI-DZ} RI-SCS-MP2^{BWI-DZ} , RIJK-SCS-MP2^{BWI-DZ} : C_{OS} =0.00, C_{SS} =1.50; RIJCOSX-SCS-MP2^{BWI-DZ} : C_{OS} =0.00, C_{SS} =0.17. All values in kcal/mol.

Dimer	Formula	MP2	SCS-MP2	SCS(MI)-MP2	SCSN-MP2	SCS-MP2-vdW	RI-MP2	RI-SCS-MP2	RIJK-MP2	RIJK-SCS-MP2	RIJCOSX-MP2	RIJCOSX-SCS-MP2	SCS-MP2^{BWI-DZ}	RI-SCS-MP2^{BWI-DZ}	RIJK-SCS-MP2^{BWI-DZ}	RIJCOSX-SCS-MP2^{BWI-DZ}	SCS-MP2-hal^{G-XZ}
chlorobenzene − acetone	C_6H_5Cl	0.75	0.25	0.10	0.02	0.60	0.76	0.25	0.75	0.25	0.76	0.25	0.31	0.30	0.30	0.29	0.17
chlorobenzene − trimethylamine	C_6H_5Cl	1.35	0.65	0.44	0.32	1.13	1.35	0.65	1.35	0.64	1.33	0.63	0.14	0.13	0.13	0.13	0.05
chloromethane − chloromethane	CH_3Cl	0.44	0.14	0.07	0.04	0.34	0.44	0.14	0.43	0.13	0.43	0.13	0.15	0.15	0.16	0.15	0.09
chloromethane − formaldehyde	CH_3Cl	0.26	0.08	0.14	0.18	0.15	0.26	0.08	0.25	0.09	0.26	0.08	0.40	0.39	0.40	0.39	0.33
chloromethane − methane	CH_3Cl	0.64	0.32	0.16	0.06	0.56	0.64	0.32	0.64	0.32	0.63	0.31	0.15	0.15	0.15	0.15	0.01
fluoromethane − fluoromethane	CH_3F	0.40	0.23	0.20	0.19	0.34	0.41	0.23	0.40	0.22	0.39	0.22	0.08	0.08	0.07	0.09	0.10
fluoromethane − methane	CH_3F	0.54	0.32	0.16	0.08	0.50	0.54	0.32	0.54	0.31	0.53	0.31	0.08	0.08	0.08	0.08	0.05
hydrogenchloride − metahnol	HCl	0.84	0.04	0.51	0.72	0.41	0.84	0.04	0.83	0.04	0.81	0.01	0.31	0.31	0.30	0.29	0.01
hydrogenchloride − methylamine	HCl	0.78	0.83	0.06	0.29	0.03	0.78	0.83	0.78	0.83	0.75	0.86	0.56	0.57	0.57	0.57	1.07
hexafuorobenzene − benzene	C_6F_6	7.54	5.22	4.28	3.76	6.89	7.55	5.23	7.56	5.25	7.51	5.19	2.21	2.22	2.24	2.24	3.04
hydrogenfluoride − methanol	HF	0.13	0.44	0.01	0.19	0.20	0.12	0.44	0.12	0.44	0.10	0.46	0.08	0.09	0.10	0.11	0.39
hydrogenfluoride − methylamine	HF	0.22	0.69	0.35	0.20	0.21	0.21	0.69	0.20	0.70	0.19	0.71	0.70	0.70	0.71	0.71	0.91
methane − chlorine	CH_4	0.57	0.22	0.13	0.07	0.46	0.57	0.22	0.56	0.21	0.58	0.22	0.16	0.15	0.16	0.14	0.07
methane − fluorine	CH_4	0.36	0.20	0.08	0.02	0.33	0.36	0.20	0.36	0.19	0.36	0.19	0.10	0.10	0.10	0.10	0.00
methanol − chloromethane	CH_3OH	0.76	0.21	0.19	0.17	0.56	0.76	0.20	0.76	0.20	0.75	0.19	0.17	0.17	0.18	0.17	0.13
methanol − fluoromethane	CH_3OH	0.56	0.15	0.22	0.24	0.39	0.56	0.15	0.55	0.14	0.56	0.15	0.00	0.00	0.01	0.00	0.03
trichloromethane − methane	$CHCl_3$	0.83	0.40	0.22	0.12	0.71	0.83	0.40	0.82	0.39	0.82	0.38	0.17	0.16	0.17	0.16	0.01
trichloromethanol − water	CCl_3OH	0.59	0.42	0.04	0.25	0.09	0.59	0.42	0.58	0.43	0.57	0.44	0.29	0.29	0.31	0.30	0.60
trifluorobenzene − benzene	$C_6H_3F_3$	6.17	4.12	3.31	2.85	5.59	6.18	4.13	6.19	4.14	6.17	4.12	1.49	1.50	1.51	1.55	2.20
trifluorochloromethane − formaldehyde	CF_3Cl	0.40	0.02	0.02	0.02	0.25	0.40	0.02	0.39	0.01	0.40	0.02	0.21	0.20	0.22	0.20	0.20
trifluoromethane − methane	CHF_3	0.65	0.44	0.29	0.21	0.61	0.66	0.44	0.65	0.44	0.65	0.44	0.06	0.06	0.05	0.06	0.18
trifluoromethano − lwater	CF_3OH	0.47	0.27	0.12	0.29	0.09	0.47	0.27	0.46	0.27	0.45	0.28	0.09	0.10	0.11	0.11	0.35

DFT

Table S77: Absolute deviations for B97M-V, ωB97X-V, ωB97M-V, ωB97X-D3, B2PLYP-D3BJ, DSD-BLYP-D3BJ, ωB97X-D4 and B2PLYP-D4. Data: 22 interaction energies. All values in kcal/mol.

Dimer	Formula	B97M-V	ωB97X-V	ωB97M-V	ωB97X-D3	B2PLYP-D3BJ	DSD-BLYP-D3BJ	ωB97X-D4	B2PLYP-D4
chlorobenzene − acetone	$C_6H_5Cl{-}C_3H_6O$	0.16	0.07	0.14	0.09	0.17	0.28	0.44	0.23
chlorobenzene − trimethylamine	$C_6H_5Cl{-}N(CH_3)_3$	0.30	0.24	0.44	0.29	0.61	0.69	0.59	0.67
chloromethane − chloromethane	$CH_3Cl{-}CH_3Cl$	0.01	0.06	0.00	0.03	0.11	0.18	0.37	0.06
chloromethane − formaldehyde	$CH_3Cl{-}CH_2O$	0.12	0.10	0.13	0.16	0.08	0.12	0.48	0.05
chloromethane − methane	$CH_3Cl{-}CH_4$	0.16	0.35	0.34	0.48	0.34	0.36	0.72	0.27
fluoromethane − fluoromethane	$CH_3F{-}CH_3F$	0.08	0.25	0.21	0.07	0.21	0.27	0.26	0.27
fluoromethane − methane	$CH_3F{-}CH_4$	0.22	0.37	0.32	0.34	0.34	0.36	0.57	0.31
hydrogenchloride − methanol	$HCl{-}CH_3OH$	0.27	0.46	0.42	0.68	0.69	0.64	1.16	0.62
hydrogenchloride − methylamine	$HCl{-}CH_3NH_2$	0.07	0.28	0.11	0.82	0.80	0.51	0.87	0.66
hexafluorobenzene − benzene	$C_6F_6{-}C_6H_6$	1.20	1.68	2.20	1.24	2.62	3.44	0.63	2.69
hydrogenfluoride − methanol	$HF{-}CH_3OH$	0.32	0.21	0.19	0.26	0.43	0.32	0.86	0.36
hydrogenfluoride − methylamine	$HF{-}CH_3NH_2$	0.54	0.51	0.22	0.58	0.66	0.41	0.96	0.56
methane − chlorine	$CH_4{-}Cl_2$	0.27	0.17	0.17	0.16	0.24	0.30	0.58	0.13
methane − fluorine	$CH_4{-}F_2$	0.19	0.06	0.06	0.19	0.13	0.18	0.02	0.09
methanol − chloromethane	$CH_3OH{-}CH_3Cl$	0.18	0.22	0.26	0.05	0.34	0.42	0.61	0.30
methanol − fluoromethane	$CH_3OH{-}CH_3F$	0.13	0.26	0.28	0.02	0.36	0.40	0.59	0.38
trichloromethane − methane	$CHCl_3{-}CH_4$	0.30	0.45	0.39	0.68	0.29	0.35	0.78	0.22
trichloromethanol − water	$CCl_3OH{-}H_2O$	0.36	0.15	0.27	0.56	0.46	0.36	0.87	0.44
trifluorobenzene − benzene	$C_6H_3F_3{-}C_6H_6$	0.96	1.31	1.83	1.35	2.01	2.65	0.92	2.13
trifluorochloromethane − formaldehyde	$CF_3Cl{-}CH_2O$	0.22	0.17	0.25	0.19	0.22	0.29	0.58	0.18
trifluoromethane − methane	$CHF_3{-}CH_4$	0.32	0.42	0.31	0.22	0.32	0.38	0.30	0.35
trifluoromethanol − water	$CF_3OH{-}H_2O$	0.01	0.28	0.30	0.34	0.40	0.36	0.87	0.42

SAPT-DFT

Table S78: Absolute deviations for CCSD(T)/CBS SAPT-DFT. For SAPT-DFT method we used the B3LYP, PBE0 and ωB97X DFA's in conjunction with the aug-cc-pVDZ basis set. Data: 22 interaction energies. All values in kcal/mol.

Dimer	Formula	SAPT-B3LYP	SAPT-PBE0	SAPT-ωB97X
chlorobenzene − acetone	$C_6H_5Cl{-}C_3H_6O$	0.85	0.83	1.20
chlorobenzene − trimethylamine	$C_6H_5Cl{-}N(CH_3)_3$	1.35	1.28	1.55
chloromethane − chloromethane	$CH_3Cl{-}CH_3Cl$	0.42	0.43	0.64
chloromethane − formaldehyde	$CH_3Cl{-}CH_2O$	0.59	0.58	0.87
chloromethane − methane	$CH_3Cl{-}CH_4$	0.47	0.49	0.51
fluoromethane − fluoromethane	$CH_3F{-}CH_3F$	0.49	0.53	0.64
fluoromethane − methane	$CH_3F{-}CH_4$	0.39	0.42	0.41
hydrogenchloride − methanol	$HCl{-}CH_3OH$	1.52	1.16	1.39
hydrogenchloride − methylamine	$HCl{-}CH_3NH_2$	2.58	1.36	2.15
hexafluorobenzene − benzene	$C_6F_6{-}C_6H_6$	2.09	2.08	2.82
hydrogenfluoride − methanol	$HF{-}CH_3OH$	2.08	1.88	1.90
hydrogenfluoride − methylamine	$HF{-}CH_3NH_2$	2.89	2.16	2.72
methane − chlorine	$CH_4{-}Cl_2$	0.62	0.60	0.57
methane − fluorine	$CH_4{-}F_2$	0.37	0.37	0.29
methanol − chloromethane	$CH_3OH{-}CH_3Cl$	1.26	1.19	1.06
methanol − fluoromethane	$CH_3OH{-}CH_3F$	1.33	1.25	1.04
trichloromethane − methane	$CHCl_3{-}CH_4$	0.58	0.62	0.67
trichloromethanol − water	$CCl_3OH{-}H_2O$	2.50	2.03	2.44
trifluorobenzene − benzene	$C_6H_3F_3{-}C_6H_6$	1.65	1.69	2.37
trifluorochloromethane − formaldehyde	$CF_3Cl{-}CH_2O$	0.89	0.88	1.14
trifluoromethane − methane	$CHF_3{-}CH_4$	0.44	0.45	0.43
trifluoromethanol − water	$CF_3OH{-}H_2O$	2.11	1.80	2.06

7.2 aug-cc-pVTZ basis set data.

7.2.1 Ionization Potentials and HOMO values for SAPT-DFT calculations

Table S79: Experimental ionization potentials (IP) of molecules comprising dimers in the X40* database. IP obtained from NIST (https://webbook.nist.gov/chemistry). Kohn-Sham energies of HOMO orbitals were calculated at the DFA/aug-cc-pVTZ level of theory (DFA=B3LYP, PBE0, and ωB97X). Ionization energies in ev and HOMO energies in atomic units.

			HOMO energies (eV)			$\Delta_{XC} = \epsilon_{HOMO} - (-IP)$		
Molecule	Formula	IP	B3LYP	PBE0	ωB97X	B3LYP	PBE0	ωB97X
acetone	C_3H_6O	9.69	-6.94	-7.25	-9.71	0.10	0.09	0.00
benzene	C_6H_6	9.24	-7.00	-7.31	-9.48	0.08	0.07	0.01
chloromethane	CH_3Cl	11.28	-8.15	-8.50	-10.98	0.12	0.10	0.01
chlorine	Cl_2	11.48	-8.40	-8.75	-11.21	0.11	0.10	0.01
fluorine	F_2	15.70	-11.37	-11.77	-14.33	0.16	0.14	0.05
fluoromethane	CH_3F	12.54	-9.57	-9.90	-12.42	0.11	0.10	0.00
formaldehyde	CH_2O	10.88	-7.59	-7.89	-10.35	0.12	0.11	0.02
hydrogenchloride	HCl	12.79	-9.15	-9.51	-12.04	0.13	0.12	0.03
hexafluorobenzene	C_6F_6	9.90	-7.70	-7.98	-10.17	0.08	0.07	0.01
hydrogenfluoride	HF	16.06	-11.45	-11.84	-14.45	0.17	0.16	0.06
methane	CH_4	12.61	-10.69	-11.00	-9.26	0.07	0.06	0.12
methanethiol	CH_3SH	9.45	-6.53	-6.84	-13.59	0.11	0.10	0.15
methanol	CH_3OH	10.85	-7.68	-8.00	-10.52	0.12	0.10	0.01
methylamine	CH_3NH_2	8.90	-6.65	-6.96	-9.46	0.08	0.07	0.02
trichloromethane	$CHCl_3$	11.30	-8.55	-8.92	-11.38	0.10	0.09	0.00
tricholomethanol	CCl_3OH	11.45	-8.55	-8.91	-11.38	0.11	0.09	0.00
trifluorobenzene	$C_6H_3F_3$	9.50	-7.42	-7.72	-9.91	0.08	0.07	0.02
trifluorochloromethane	CF_3Cl	13.08	-9.74	-10.11	-12.63	0.12	0.11	0.02
trifluoromethane	CHF_3	13.90	-11.12	-11.43	-13.96	0.10	0.09	0.00
trifluoromethanol	CF_3OH	13.08	-10.24	-10.62	-13.18	0.10	0.09	0.00
trimethylamine	$N(CH_3)_3$	7.80	-6.02	-6.30	-8.69	0.07	0.05	0.03

7.2.2 Evaluation of mean absolute deviation in a grid of C_{OS} and C_{SS} values

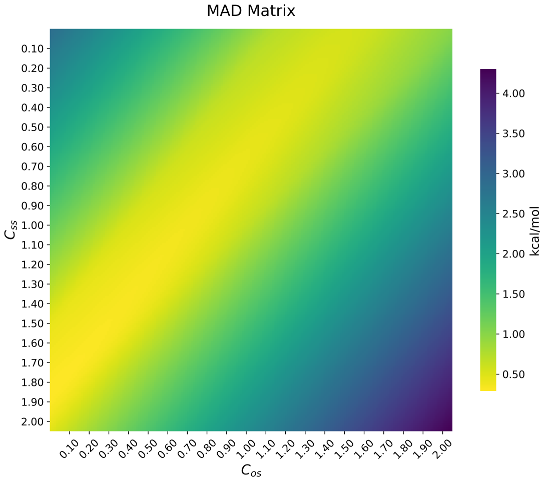

(a)

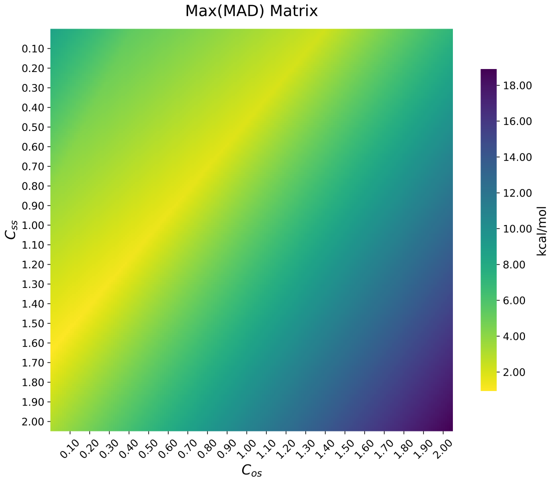

(b)

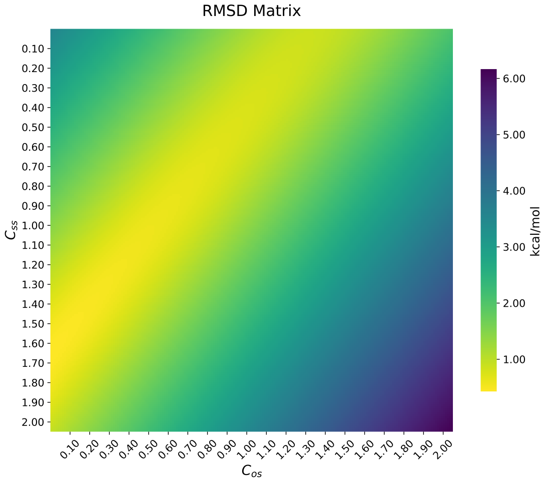

(c)

Figure S17: Evaluation of MADs (S17a), Max(MADs) (S17b) and RMSDs (S17c) in grid of values C_{OS} and C_{SS}. The optimal coefficients are C_{OS}=0.27 and C_{SS}=1.38. The theory level used is RIJK-MP2/aug-cc-pVDZ.

7.2.3 Interaction energies

MP2 with C_{OS} y C_{SS} values

Table S80: Interaction energies for CCSD(T)/CBS, MP2, SCS-MP2, SCS(MI)-MP2, SCSN-MP2, SCS-MP2-vdW, RI-MP2, RI-SCS-MP2, RIJK-MP2, RIJK-SCS-MP2, RIJCOSX-MP2, RIJCOSX-SCS-MP2, SCS-MP2^{BWI-TZ}, RI-SCS-MP2^{BWI-TZ}, RIJK-SCS-MP2^{BWI-TZ}, RIJCOSX-SCS-MP2^{BWI-DZ} and SCS-MP2-hal^{G-XZ}. Data: 22 interaction energies. MP2: $C_{OS} = C_{SS}$ =1.00; SCS-MP2: C_{OS} =1.20, C_{SS} =0.33; SCS(MI)-MP2: C_{OS} =0.40, C_{SS} =1.29; SCSN-MP2: C_{OS} =0.00, C_{SS} =1.76; SCS-MP2-vdW: C_{OS} =1.28, C_{SS} =0.50; SCS-MP2^{BWI-TZ} RI-SCS-MP2^{BWI-TZ} , RIJK-SCS-MP2^{BWI-TZ} : C_{OS} =0.27, C_{SS} =1.38; RIJCOSX-SCS-MP2^{BWI-TZ} : C_{OS} =0.17, C_{SS} =1.59. All values in kcal/mol.

Dimer	Formula	CCSD(T)/CBS	MP2	SCS-MP2	SCS(MI)-MP2	SCSN-MP2	SCS-MP2-vdW	RI-MP2	RI-SCS-MP2	RIJK-MP2	RIJK-SCS-MP2	RIJCOSX-MP2	RIJCOSX-SCS-MP2	SCS-MP2^{BWI-TZ}	RI-SCS-MP2^{BWI-TZ}	RIJK-SCS-MP2^{BWI-TZ}	RIJCOSX-SCS-MP2^{BWI-TZ}	SCS-MP2-hal^{G-XZ}
chlorobenzene − acetone	C_6H_5Cl	-1.49	-1.91	-1.38	-1.31	-1.27	-1.73	-1.91	-1.38	-1.91	-1.38	-1.79	-1.26	-1.22	-1.22	-1.21	-1.10	-1.22
chlorobenzene − trimethylamine	C_6H_5Cl	-2.11	-2.89	-2.16	-2.06	-1.99	-2.64	-2.89	-2.16	-2.89	-2.15	-2.65	-1.92	-1.93	-1.93	-1.92	-1.69	-1.93
chloromethane − chloromethane	CH_3Cl	-1.34	-1.58	-1.24	-1.25	-1.25	-1.45	-1.58	-1.24	-1.57	-1.24	-1.59	-1.25	-1.21	-1.21	-1.20	-1.22	-1.14
chloromethane − formaldehyde	CH_3Cl	-1.17	-1.39	-1.01	-0.98	-0.96	-1.25	-1.39	-1.01	-1.38	-1.00	-1.40	-1.01	-0.91	-0.91	-0.90	-0.93	-0.89
chloromethane − methane	CH_3Cl	-0.98	-1.24	-0.89	-0.82	-0.78	-1.12	-1.24	-0.89	-1.23	-0.89	-1.24	-0.90	-0.76	-0.76	-0.75	-0.76	-0.79
fluoromethane − fluoromethane	CH_3F	-1.65	-1.74	-1.55	-1.54	-1.54	-1.67	-1.74	-1.55	-1.73	-1.54	-1.72	-1.54	-1.51	-1.51	-1.51	-1.50	-1.49
fluoromethane − methane	CH_3F	-0.75	-0.90	-0.66	-0.60	-0.56	-0.82	-0.90	-0.66	-0.89	-0.65	-0.90	-0.66	-0.55	-0.55	-0.54	-0.55	-0.58
hydrogenchloride − metahnol	HCl	-6.30	-6.97	-6.11	-6.60	-6.82	-6.52	-6.97	-6.11	-6.96	-6.11	-6.93	-6.07	-6.56	-6.56	-6.56	-6.54	-5.89
hydrogenchloride − methylamine	HCl	-11.42	-11.42	-9.72	-10.55	-10.94	-10.56	-11.42	-9.72	-11.42	-9.71	-11.38	-9.67	-10.46	-10.46	-10.46	-10.45	-9.26
hexafuorobenzene − benzene	C_6F_6	-6.12	-11.05	-8.74	-8.11	-7.73	-10.33	-11.05	-8.74	-11.07	-8.76	-11.01	-8.70	-7.62	-7.62	-7.65	-7.62	-7.99
hydrogenfluoride − methanol	HF	-9.59	-9.85	-9.27	-9.67	-9.86	-9.53	-9.85	-9.27	-9.84	-9.26	-9.81	-9.23	-9.66	-9.66	-9.65	-9.63	-9.12
hydrogenfluoride − methylamine	HF	-14.32	-14.31	-13.40	-13.70	-13.83	-13.88	-14.31	-13.40	-14.30	-13.39	-14.26	-13.35	-13.62	-13.62	-13.61	-13.59	-13.14
methane − chloride	CH_4	-1.08	-1.46	-1.07	-1.03	-1.00	-1.32	-1.46	-1.07	-1.44	-1.06	-1.45	-1.06	-0.96	-0.96	-0.95	-0.96	-0.95
methane − fluoride	CH_4	-0.49	-0.71	-0.54	-0.45	-0.40	-0.67	-0.71	-0.54	-0.71	-0.54	-0.71	-0.54	-0.40	-0.40	-0.40	-0.40	-0.48
methanolchloro − methane	CH_3OH	-3.78	-4.27	-3.66	-3.70	-3.71	-4.03	-4.27	-3.66	-4.26	-3.66	-4.28	-3.68	-3.62	-3.62	-3.61	-3.64	-3.49
methanolfluoro − methane	CH_3OH	-3.89	-4.17	-3.74	-3.78	-3.79	-4.00	-4.17	-3.74	-4.16	-3.73	-4.16	-3.73	-3.72	-3.72	-3.71	-3.71	-3.62
trichloromethane − methane	$CHCl_3$	-1.15	-1.64	-1.18	-1.07	-1.00	-1.49	-1.64	-1.18	-1.63	-1.17	-1.63	-1.17	-0.97	-0.97	-0.97	-0.97	-1.03
trichloromethanol − water	CCl_3OH	-10.41	-10.49	-9.43	-9.97	-10.23	-9.95	-10.49	-9.43	-10.48	-9.42	-10.46	-9.40	-9.92	-9.92	-9.91	-9.91	-9.15
trifluorobenzene − benzene	$C_6H_3F_3$	-4.41	-8.43	-6.37	-5.88	-5.59	-7.76	-8.43	-6.37	-8.45	-6.39	-8.39	-6.33	-5.47	-5.47	-5.49	-5.46	-5.71
trifluorochloromethane − formaldehyde	CF_3Cl	-2.25	-2.48	-2.05	-2.06	-2.06	-2.31	-2.48	-2.05	-2.47	-2.04	-2.48	-2.05	-2.00	-2.00	-1.99	-2.01	-1.92
trifluoromethane − methane	CHF_3	-0.69	-0.91	-0.69	-0.62	-0.58	-0.84	-0.91	-0.69	-0.91	-0.68	-0.91	-0.69	-0.57	-0.57	-0.57	-0.58	-0.61
trifluoromethanol − water	CF_3OH	-9.67	-9.77	-9.00	-9.39	-9.57	-9.38	-9.77	-9.00	-9.76	-8.99	-9.74	-8.98	-9.35	-9.35	-9.34	-9.34	-8.80

DFT

Table S81: Interaction energies for CCSD(T)/CBS, B97M-V, ωB97X-V, ωB97M-V, ωB97X-D3, B2PLYP-D3BJ, DSD-BLYP-D3BJ, ωB97X-D4 and B2PLYP-D4. Data: 22 interaction energies. All values in kcal/mol.

Dimer	Formula	CCSD(T)/CBS	B97M-V	ωB97X-V	ωB97M-V	ωB97X-D3	B2PLYP-D3BJ	DSD-BLYP-D3BJ	ωB97X-D4	B2PLYP-D4
chlorobenzene − acetone	$C_6H_5Cl-C_3H_6O$	-1.49	-1.41	-1.27	-1.29	-1.03	-1.40	-1.47	-1.59	-1.42
chlorobenzene − trimethylamine	$C_6H_5Cl-N(CH_3)_3$	-2.11	-2.04	-1.84	-1.95	-1.79	-2.27	-2.31	-2.11	-2.25
chloromethane − chloromethane	CH_3Cl-CH_3Cl	-1.34	-1.15	-1.20	-1.11	-1.10	-1.27	-1.33	-1.47	-1.23
chloromethane − formaldehyde	CH_3Cl-CH_2O	-1.17	-1.12	-1.07	-1.08	-0.79	-1.11	-1.17	-1.44	-1.08
chloromethane − methane	CH_3Cl-CH_4	-0.98	-0.83	-0.96	-0.89	-1.05	-0.93	-0.96	-1.31	-0.87
fluoromethane − fluoromethane	CH_3F-CH_3F	-1.65	-1.41	-1.61	-1.57	-1.29	-1.51	-1.57	-1.64	-1.56
fluoromethane − methane	CH_3F-CH_4	-0.75	-0.66	-0.79	-0.71	-0.77	-0.69	-0.72	-1.00	-0.66
hydrogenchloride − methanol	$HCl-CH_3OH$	-6.30	-6.18	-6.31	-6.21	-6.48	-6.66	-6.65	-7.01	-6.59
hydrogenchloride − methylamine	$HCl-CH_3NH_2$	-11.42	-10.40	-10.54	-10.19	-11.01	-11.16	-10.94	-11.09	-11.00
hexafluorobenzene − benzene	$C_6F_6-C_6H_6$	-6.12	-6.03	-6.68	-7.05	-6.37	-7.37	-7.82	-5.99	-7.43
hydrogenfluoride − methanol	$HF-CH_3OH$	-9.59	-9.23	-9.74	-9.64	-9.73	-10.00	-9.93	-10.40	-9.92
hydrogenfluoride − methylamine	$HF-CH_3NH_2$	-14.32	-13.49	-14.39	-14.01	-14.48	-14.54	-14.35	-14.93	-14.43
methane − chlorine	CH_4-Cl_2	-1.08	-1.06	-0.95	-0.90	-0.85	-1.09	-1.16	-1.30	-0.98
methane − fluorine	CH_4-F_2	-0.49	-0.57	-0.49	-0.46	-0.22	-0.50	-0.54	-0.46	-0.46
methanol − chloromethane	CH_3OH-CH_3Cl	-3.78	-3.75	-3.69	-3.72	-3.53	-3.81	-3.89	-4.15	-3.79
methanol − fluoromethane	CH_3OH-CH_3F	-3.89	-3.70	-3.81	-3.82	-3.56	-3.90	-3.95	-4.20	-3.93
trichloromethane − methane	$CHCl_3-CH_4$	-1.15	-1.11	-1.26	-1.16	-1.44	-1.17	-1.21	-1.57	-1.09
trichloromethanol − water	CCl_3OH-H_2O	-10.41	-10.40	-10.13	-10.23	-10.56	-10.45	-10.31	-10.90	-10.42
trifluorobenzene − benzene	$C_6H_3F_3-C_6H_6$	-4.41	-4.43	-4.73	-5.05	-4.78	-5.30	-5.64	-4.60	-5.39
trifluorochloromethane − formaldehyde	CF_3Cl-CH_2O	-2.25	-2.10	-2.06	-2.10	-1.63	-2.21	-2.29	-2.42	-2.18
trifluoromethane − methane	CHF_3-CH_4	-0.69	-0.71	-0.88	-0.76	-0.75	-0.64	-0.69	-0.86	-0.66
trifluoromethanol − water	CF_3OH-H_2O	-9.67	-9.27	-9.58	-9.56	-9.61	-9.68	-9.64	-10.18	-9.70

SAPT-DFT

Table S82: Interaction energies for CCSD(T)/CBS and SAPT-DFT. For SAPT-DFT method we used the B3LYP, PBE0 and ωB97X DFA's in conjunction with the aug-cc-pVDZ basis set. Data: 22 interaction energies. All values in kcal/mol.

Dimer	Formula	CCSD(T)/CBS	SAPT-B3LYP	SAPT-PBE0	SAPT-ωB97X
chlorobenzene − acetone	$C_6H_5Cl{-}C_3H_6O$	-1.49	-0.90	-0.88	-0.49
chlorobenzene − trimethylamine	$C_6H_5Cl{-}N(CH_3)_3$	-2.11	-1.07	-1.09	-0.80
chloromethane − chloromethane	$CH_3Cl{-}CH_3Cl$	-1.34	-1.11	-1.09	-0.85
chloromethane − formaldehyde	$CH_3Cl{-}CH_2O$	-1.17	-0.87	-0.85	-0.54
chloromethane − methane	$CH_3Cl{-}CH_4$	-0.98	-0.69	-0.67	-0.63
fluoromethane − fluoromethane	$CH_3F{-}CH_3F$	-1.65	-1.35	-1.29	-1.14
fluoromethane − methane	$CH_3F{-}CH_4$	-0.75	-0.51	-0.48	-0.47
hydrogenchloride − methanol	$HCl{-}CH_3OH$	-6.30	-5.21	-5.53	-5.21
hydrogenchloride − methylamine	$HCl{-}CH_3NH_2$	-11.42	-9.56	-10.72	-9.73
hexafluorobenzene − benzene	$C_6F_6{-}C_6H_6$	-6.12	-4.62	-4.55	-3.77
hydrogenfluoride − methanol	$HF{-}CH_3OH$	-9.59	-8.19	-8.37	-8.18
hydrogenfluoride − methylamine	$HF{-}CH_3NH_2$	-14.32	-12.17	-12.87	-12.09
methane − chlorine	$CH_4{-}Cl_2$	-1.08	-0.71	-0.71	-0.72
methane − fluorine	$CH_4{-}F_2$	-0.49	-0.26	-0.25	-0.31
methanol − chloromethane	$CH_3OH{-}CH_3Cl$	-3.78	-3.00	-3.05	-3.12
methanol − fluoromethane	$CH_3OH{-}CH_3F$	-3.89	-2.86	-2.90	-3.09
trichloromethane − methane	$CHCl_3{-}CH_4$	-1.15	-0.79	-0.75	-0.66
trichloromethanol − water	$CCl_3OH{-}H_2O$	-10.41	-8.61	-9.03	-8.46
trifluorobenzene − benzene	$C_6H_3F_3{-}C_6H_6$	-4.41	-3.26	-3.16	-2.46
trifluorochloromethane − formaldehyde	$CF_3Cl{-}CH_2O$	-2.25	-1.64	-1.60	-1.30
trifluoromethane − methane	$CHF_3{-}CH_4$	-0.69	-0.42	-0.39	-0.40
trifluoromethanol − water	$CF_3OH{-}H_2O$	-9.67	-8.17	-8.45	-8.05

7.2.4 Absolute deviations of molecular systems.

MP2 with C_{OS} y C_{SS} values

Table S83: Absolute deviations for MP2, SCS-MP2, SCS(MI)-MP2, SCSN-MP2, SCS-MP2-vdW, RI-MP2, RI-SCS-MP2, RIJK-MP2, RIJK-SCS-MP2, RIJCOSX-MP2, RIJCOSX-SCS-MP2, SCS-MP2^{BWI-TZ}, RI-SCS-MP2^{BWI-TZ}, RIJK-SCS-MP2^{BWI-TZ}, RIJCOSX-SCS-MP2^{BWI-DZ} and SCS-MP2-hal^{G-XZ}. Data: 22 interaction energies. MP2: $C_{OS} = C_{SS}$ =1.00; SCS-MP2: C_{OS} =1.20, C_{SS} =0.33; SCS(MI)-MP2: C_{OS} =0.40, C_{SS} =1.29; SCSN-MP2: C_{OS} =0.00, C_{SS} =1.76; SCS-MP2-vdW: C_{OS} =1.28, C_{SS} =0.50; SCS-MP2^{BWI-TZ} RI-SCS-MP2^{BWI-TZ} , RIJK-SCS-MP2^{BWI-TZ} : C_{OS} =0.27, C_{SS} =1.38; RIJCOSX-SCS-MP2^{BWI-TZ} : C_{OS} =0.17, C_{SS} =1.59. All values in kcal/mol.

Dimer	Formula	MP2	SCS-MP2	SCS(MI)-MP2	SCSN-MP2	SCS-MP2-vdW	RI-MP2	RI-SCS-MP2	RIJK-MP2	RIJK-SCS-MP2	RIJCOSX-MP2	RIJCOSX-SCS-MP2	SCS-MP2^{BWI-TZ}	RI-SCS-MP2^{BWI-TZ}	RIJK-SCS-MP2^{BWI-TZ}	RIJCOSX-SCS-MP2^{BWI-TZ}	SCS-MP2-hal^{G-XZ}
chlorobenzene − acetone	C_6H_5Cl	0.42	0.11	0.18	0.22	0.24	0.42	0.11	0.42	0.11	0.30	0.23	0.27	0.27	0.28	0.39	0.27
chlorobenzene − trimethylamine	C_6H_5Cl	0.78	0.05	0.05	0.12	0.53	0.78	0.05	0.78	0.04	0.54	0.19	0.18	0.18	0.19	0.42	0.18
chloromethane − chloromethane	CH_3Cl	0.24	0.10	0.09	0.09	0.11	0.24	0.10	0.23	0.10	0.25	0.09	0.13	0.13	0.14	0.12	0.20
chloromethane − formaldehyde	CH_3Cl	0.22	0.16	0.19	0.21	0.08	0.22	0.16	0.21	0.17	0.23	0.16	0.26	0.26	0.27	0.24	0.28
chloromethane − methane	CH_3Cl	0.26	0.09	0.16	0.20	0.14	0.26	0.09	0.25	0.09	0.26	0.08	0.22	0.22	0.23	0.22	0.19
fluoromethane − fluoromethane	CH_3F	0.09	0.10	0.11	0.11	0.02	0.09	0.10	0.08	0.11	0.07	0.11	0.14	0.14	0.14	0.15	0.16
fluoromethane − methane	CH_3F	0.15	0.09	0.15	0.19	0.07	0.15	0.09	0.14	0.10	0.15	0.09	0.20	0.20	0.21	0.20	0.17
hydrogenchloride − metahnol	HCl	0.67	0.19	0.30	0.52	0.22	0.67	0.19	0.66	0.19	0.63	0.23	0.26	0.26	0.26	0.24	0.41
hydrogenchloride − methylamine	HCl	0.00	1.70	0.87	0.48	0.86	0.00	1.70	0.00	1.71	0.04	1.75	0.96	0.96	0.96	0.97	2.16
hexafuorobenzene − benzene	C_6F_6	4.93	2.62	1.99	1.61	4.21	4.93	2.62	4.95	2.64	4.89	2.58	1.50	1.50	1.53	1.50	1.87
hydrogenfluoride − methanol	HF	0.26	0.32	0.08	0.27	0.06	0.26	0.32	0.25	0.33	0.22	0.36	0.07	0.07	0.06	0.04	0.47
hydrogenfluoride − methylamine	HF	0.01	0.92	0.62	0.49	0.44	0.01	0.92	0.02	0.93	0.06	0.97	0.70	0.70	0.71	0.73	1.18
methane − chlorine	CH_4	0.38	0.01	0.05	0.08	0.24	0.38	0.01	0.36	0.02	0.37	0.02	0.12	0.12	0.13	0.12	0.13
methane − fluorine	CH_4	0.22	0.05	0.04	0.09	0.18	0.22	0.05	0.22	0.05	0.22	0.05	0.09	0.09	0.09	0.09	0.01
methanol − chloromethane	CH_3OH	0.49	0.12	0.08	0.07	0.25	0.49	0.12	0.48	0.12	0.50	0.10	0.16	0.16	0.17	0.14	0.29
methanol − fluoromethane	CH_3OH	0.28	0.15	0.11	0.10	0.11	0.28	0.15	0.27	0.16	0.27	0.16	0.17	0.17	0.18	0.18	0.27
trichloromethane − methane	$CHCl_3$	0.49	0.03	0.08	0.15	0.34	0.49	0.03	0.48	0.02	0.48	0.02	0.18	0.18	0.18	0.18	0.12
trichloromethanol − water	CCl_3OH	0.08	0.98	0.44	0.18	0.46	0.08	0.98	0.07	0.99	0.05	1.01	0.49	0.49	0.50	0.50	1.26
trifluorobenzene − benzene	$C_6H_3F_3$	4.02	1.96	1.47	1.18	3.35	4.02	1.96	4.04	1.98	3.98	1.92	1.06	1.06	1.08	1.05	1.30
trifluorochloromethane − formaldehyde	CF_3Cl	0.23	0.20	0.19	0.19	0.06	0.23	0.20	0.22	0.21	0.23	0.20	0.25	0.25	0.26	0.24	0.33
trifluoromethane − methane	CHF_3	0.22	0.00	0.07	0.11	0.15	0.22	0.00	0.22	0.01	0.22	0.00	0.12	0.12	0.12	0.11	0.08
trifluoromethanol − water	CF_3OH	0.10	0.67	0.28	0.10	0.29	0.10	0.67	0.09	0.68	0.07	0.69	0.32	0.32	0.33	0.33	0.87

DFT

Table S84: Absolute deviations for B97M-V, ωB97X-V, ωB97M-V, ωB97X-D3, B2PLYP-D3BJ, DSD-BLYP-D3BJ, ωB97X-D4 and B2PLYP-D4. Data: 22 interaction energies. All values in kcal/mol.

Dimer	Formula	B97M-V	ωB97X-V	ωB97M-V	ωB97X-D3	B2PLYP-D3BJ	DSD-BLYP-D3BJ	ωB97X-D4	B2PLYP-D4
chlorobenzene − acetone	$C_6H_5Cl{-}C_3H_6O$	0.08	0.22	0.20	0.46	0.09	0.01	0.10	0.07
chlorobenzene − trimethylamine	$C_6H_5Cl{-}N(CH_3)_3$	0.07	0.27	0.16	0.32	0.16	0.20	0.01	0.14
chloromethane − chloromethane	$CH_3Cl{-}CH_3Cl$	0.19	0.14	0.23	0.23	0.06	0.01	0.13	0.11
chloromethane − formaldehyde	$CH_3Cl{-}CH_2O$	0.05	0.10	0.09	0.38	0.06	0.00	0.27	0.09
chloromethane − methane	$CH_3Cl{-}CH_4$	0.15	0.02	0.09	0.07	0.05	0.02	0.33	0.11
fluoromethane − fluoromethane	$CH_3F{-}CH_3F$	0.23	0.04	0.08	0.36	0.14	0.08	0.01	0.09
fluoromethane − methane	$CH_3F{-}CH_4$	0.09	0.04	0.05	0.02	0.06	0.03	0.25	0.09
hydrogenchloride − methanol	$HCl{-}CH_3OH$	0.12	0.01	0.09	0.18	0.36	0.35	0.71	0.29
hydrogenchloride − methylamine	$HCl{-}CH_3NH_2$	1.02	0.88	1.22	0.41	0.26	0.48	0.33	0.41
hexafluorobenzene − benzene	$C_6F_6{-}C_6H_6$	0.09	0.56	0.92	0.24	1.25	1.70	0.13	1.31
hydrogenfluoride − methanol	$HF{-}CH_3OH$	0.36	0.15	0.05	0.14	0.40	0.34	0.81	0.32
hydrogenfluoride − methylamine	$HF{-}CH_3NH_2$	0.83	0.07	0.31	0.16	0.23	0.03	0.61	0.12
methane − chlorine	$CH_4{-}Cl_2$	0.02	0.13	0.18	0.23	0.02	0.09	0.22	0.10
methane − fluorine	$CH_4{-}F_2$	0.07	0.01	0.03	0.27	0.01	0.05	0.03	0.03
methanol − chloromethane	$CH_3OH{-}CH_3Cl$	0.03	0.09	0.06	0.25	0.03	0.11	0.37	0.01
methanol − fluoromethane	$CH_3OH{-}CH_3F$	0.19	0.08	0.08	0.34	0.01	0.06	0.30	0.03
trichloromethane − methane	$CHCl_3{-}CH_4$	0.04	0.11	0.02	0.29	0.03	0.06	0.42	0.05
trichloromethanol − water	$CCl_3OH{-}H_2O$	0.00	0.27	0.17	0.15	0.04	0.10	0.50	0.01
trifluorobenzene − benzene	$C_6H_3F_3{-}C_6H_6$	0.02	0.32	0.64	0.38	0.90	1.24	0.19	0.99
trifluorochloromethane − formaldehyde	$CF_3Cl{-}CH_2O$	0.15	0.19	0.15	0.62	0.03	0.04	0.18	0.06
trifluoromethane − methane	$CHF_3{-}CH_4$	0.02	0.19	0.07	0.06	0.06	0.01	0.17	0.03
trifluoromethanol − water	$CF_3OH{-}H_2O$	0.40	0.09	0.11	0.06	0.01	0.03	0.51	0.03

SAPT-DFT

Table S85: Absolute deviations for SAPT-DFT. For SAPT-DFT method we used the B3LYP, PBE0 and ωB97X DFA's in conjunction with the aug-cc-pVTZ basis set. Data: 22 interaction energies. All values in kcal/mol.

Dimer	Formula	SAPT-B3LYP	SAPT-PBE0	SAPT-ωB97X
chlorobenzene − acetone	$C_6H_5Cl{-}C_3H_6O$	0.59	0.61	1.00
chlorobenzene − trimethylamine	$C_6H_5Cl{-}N(CH_3)_3$	1.04	1.02	1.31
chloromethane − chloromethane	$CH_3Cl{-}CH_3Cl$	0.23	0.25	0.48
chloromethane − formaldehyde	$CH_3Cl{-}CH_2O$	0.30	0.32	0.63
chloromethane − methane	$CH_3Cl{-}CH_4$	0.29	0.31	0.35
fluoromethane − fluoromethane	$CH_3F{-}CH_3F$	0.30	0.36	0.51
fluoromethane − methane	$CH_3F{-}CH_4$	0.24	0.27	0.28
hydrogenchloride − methanol	$HCl{-}CH_3OH$	1.09	0.77	1.09
hydrogenchloride − methylamine	$HCl{-}CH_3NH_2$	1.86	0.70	1.69
hexafluorobenzene − benzene	$C_6F_6{-}C_6H_6$	1.50	1.58	2.35
hydrogenfluoride − methanol	$HF{-}CH_3OH$	1.40	1.23	1.41
hydrogenfluoride − methylamine	$HF{-}CH_3NH_2$	2.15	1.44	2.23
methane − chlorine	$CH_4{-}Cl_2$	0.37	0.37	0.36
methane − fluorine	$CH_4{-}F_2$	0.23	0.24	0.18
methanol − chloromethane	$CH_3OH{-}CH_3Cl$	0.77	0.72	0.65
methanol − fluoromethane	$CH_3OH{-}CH_3F$	1.03	0.99	0.80
trichloromethane − methane	$CHCl_3{-}CH_4$	0.35	0.40	0.48
trichloromethanol − water	$CCl_3OH{-}H_2O$	1.79	1.37	1.95
trifluorobenzene − benzene	$C_6H_3F_3{-}C_6H_6$	1.14	1.24	1.94
trifluorochloromethane − formaldehyde	$CF_3Cl{-}CH_2O$	0.61	0.65	0.94
trifluoromethane − methane	$CHF_3{-}CH_4$	0.27	0.30	0.29
trifluoromethanol − water	$CF_3OH{-}H_2O$	1.50	1.22	1.62

7.3 Mean calculation times

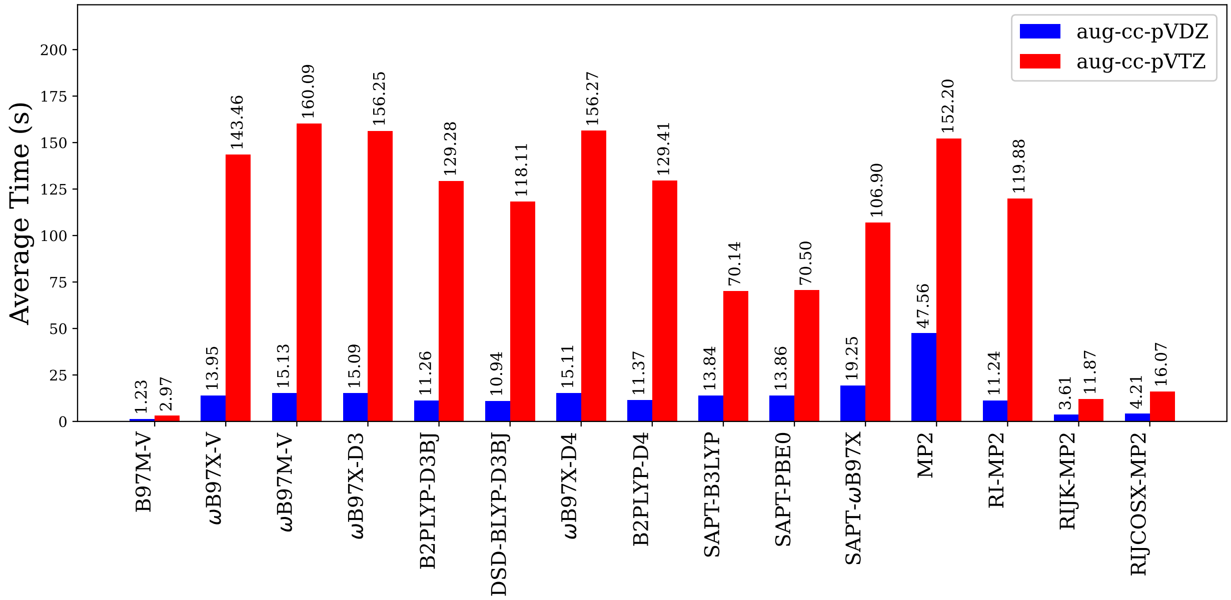

Figure S18: Comparison of computational times for different methods and basis sets in the dataset X40*. All values in seconds.

8 Orca input for RIJK-MP2/aug-cc-pVDZ calculations

$! \, UHF \; RI - MP2 \; aug - cc - pVDZ \; aug - cc - pVDZ/C \; RIJK \; def2/JK$

$! \, TightSCF$

$\%pal$

$nprocs \; 10$

end

$\%MaxCore \; 2000$

$\%scf$

$maxiter = 300$

end

$\%mp2$

$NatOrbs \; true$

$PrintLevel \; 3$

end

$\%output$

$PrintLevel \; Huge$

end

$* \; xyz \; 0 \; 1$

$Molecular \; structure$

$*$

9 Python script to minimize MAD

In the following python script the file all-mp2-aug-cc-pVDZ.csv contains the information of the Hartree-Fock energy, the opposite-spin and same-spin correlation energy, as well as the reference interaction energies (CCSD(T)/CBS) necessary to carry out the MAD minimization process.

```python
import os
import pandas as pd
import numpy as np
import matplotlib.pyplot as plt
import seaborn as sns
from scipy.optimize import minimize


class InteractionEnergyAnalysis:
    """Class to compute interaction energies, evaluate methods, and analyze errors."""

    HARTREE_TO_KCAL_MOL = 627.509  # Conversion factor from Hartree to kcal/mol

    def __init__(self, file_path):
        """
        Initialize the class with the data file path and set up the output folder.

        Args:
            file_path (str): Path to the CSV file containing the data.
        """
        self.file_path = file_path
        self.file_name = os.path.splitext(os.path.basename(file_path))[0]  # Extract file name without extension
        self.output_folder = f"{self.file_name}_results"  # Output folder for results
        os.makedirs(self.output_folder, exist_ok=True)  # Create output folder if it doesn't exist

        # Load data from the CSV file
        self.data = pd.read_csv(file_path)

        # Predefined methods with their (C_ss, C_so) coefficients
        self.methods = {
            "MP2": (1.0, 1.0),
```

```python
            "SCS-MP2": (0.33, 1.20),
            "SCS(MI)-MP2": (1.29, 0.40),
            "SCSN-MP2": (1.76, 0.0),
            "SCS-MP2-vdw": (0.50, 1.28)
        }

        # Grid search space for C_ss and C_so
        self.C_ss_values = np.arange(0.00, 2.05, 0.01)  # C_ss values from 0.0 to 2.0 with step 0.01
        self.C_so_values = np.arange(0.00, 2.05, 0.01)  # C_so values from 0.0 to 2.0 with step 0.01

        # Initialize matrices for error metrics
        shape = (len(self.C_ss_values), len(self.C_so_values))
        self.RMSE_matrix = np.zeros(shape)  # Root Mean Square Error matrix
        self.MAD_matrix = np.zeros(shape)  # Mean Absolute Deviation matrix
        self.MAD_max_matrix = np.zeros(shape)  # Maximum Absolute Deviation matrix
        self.MRPD_matrix = np.zeros(shape)  # Mean Relative Percentage Deviation matrix
        self.MAD_max_system_matrix = np.empty(shape, dtype=object)  # System responsible for MAD_max

        # Store the minimum MRPD value for plotting
        self.min_mrpd = None

    def compute_interaction_energy(self, C_ss, C_so):
        """
        Compute the interaction energy for given C_ss and C_so coefficients.

        Args:
            C_ss (float): Coefficient for same-spin interactions.
            C_so (float): Coefficient for opposite-spin interactions.

        Returns:
            np.ndarray: Interaction energies in kcal/mol.
        """
        # Compute interaction energy in atomic units (a.u.)
        E_au = (
            self.data['Ehf_dimer'] - (self.data['Ehf_monomer1'] + self.data['Ehf_monomer2'])
            + C_ss * (self.data['Ess_dimer'] - (self.data['Ess_monomer1'] + self.data['Ess_monomer2']))
            + C_so * (self.data['Eso_dimer'] - (self.data['Eso_monomer1'] + self.data['Eso_monomer2']))
        )
        # Convert to kcal/mol and return
        return E_au * self.HARTREE_TO_KCAL_MOL
```

```

    def compute_rmse(self, coefficients):
        """
        Compute the Root Mean Square Error (RMSE) for given C_ss and C_so.

        Args:
            coefficients (list): List containing C_ss and C_so.

        Returns:
            float: RMSE value.
        """
        C_ss, C_so = coefficients
        E_interaction = self.compute_interaction_energy(C_ss, C_so)
        errors = self.data['E_interaction_CC'] - E_interaction
        return np.sqrt(np.mean(errors ** 2))

    def compute_mad(self, coefficients):
        """
        Compute the Mean Absolute Deviation (MAD) for given C_ss and C_so.

        Args:
            coefficients (list): List containing C_ss and C_so.

        Returns:
            float: MAD value.
        """
        C_ss, C_so = coefficients
        E_interaction = self.compute_interaction_energy(C_ss, C_so)
        errors = self.data['E_interaction_CC'] - E_interaction
        return np.mean(np.abs(errors))

    def compute_mad_max(self, coefficients):
        """
        Compute the Maximum Absolute Deviation (MAD_max) for given C_ss and C_so.

        Args:
            coefficients (list): List containing C_ss and C_so.

        Returns:
            float: MAD_max value.
```

```
        """
        C_ss, C_so = coefficients
        E_interaction = self.compute_interaction_energy(C_ss, C_so)
        errors = self.data['E_interaction_CC'] - E_interaction
        return np.max(np.abs(errors))

    def compute_mrpd(self, coefficients):
        """
        Compute the Mean Relative Percentage Deviation (MRPD) for given C_ss and C_so.

        Args:
            coefficients (list): List containing C_ss and C_so.

        Returns:
            float: MRPD value.
        """
        C_ss, C_so = coefficients
        E_interaction = self.compute_interaction_energy(C_ss, C_so)
        mrpd_values = np.abs((self.data['E_interaction_CC'] - E_interaction) / self.data['E_interaction_CC'])
        ↪  * 100
        return np.mean(mrpd_values)

    def compute_all_metrics(self, C_ss, C_so):
        """
        Compute all deviation metrics (RMSE, MAD, MAD_max, MRPD) for given C_ss and C_so.

        Args:
            C_ss (float): Coefficient for same-spin interactions.
            C_so (float): Coefficient for opposite-spin interactions.

        Returns:
            dict: Dictionary containing RMSE, MAD, MAD_max, MRPD, and the system responsible for MAD_max.
        """
        E_interaction = self.compute_interaction_energy(C_ss, C_so)
        errors = self.data['E_interaction_CC'] - E_interaction

        rmse = np.sqrt(np.mean(errors ** 2))
        mad = np.mean(np.abs(errors))
        mad_max = np.max(np.abs(errors))
        mrpd = np.mean(np.abs((self.data['E_interaction_CC'] - E_interaction) /
        ↪  self.data['E_interaction_CC']) * 100)
```

```python

        return {
            "RMSE": rmse,
            "MAD": mad,
            "MAD_max": mad_max,
            "MRPD": mrpd,
            "System": self.data.loc[np.abs(errors).idxmax(), 'System']
        }

    def find_minimums(self):
        """
        Find the coefficients minimizing RMSE, MAD, MAD_max, and MRPD using optimization.

        Returns:
            dict: Dictionary containing optimized coefficients and their corresponding metrics.
        """
        # Bounds for C_ss and C_so
        bounds = [(0.0, 2.5), (0.0, 2.5)]  # C_ss and C_so bounds

        # Initial guess for C_ss and C_so
        initial_guess = [1.0, 1.0]

        # Minimize RMSE
        rmse_result = minimize(self.compute_rmse, initial_guess, bounds=bounds)
        # Minimize MAD
        mad_result = minimize(self.compute_mad, initial_guess, bounds=bounds)
        # Minimize MAD_max
        mad_max_result = minimize(self.compute_mad_max, initial_guess, bounds=bounds)
        # Minimize MRPD
        mrpd_result = minimize(self.compute_mrpd, initial_guess, bounds=bounds)

        return {
            "RMSE": {
                "C_ss": rmse_result.x[0],
                "C_so": rmse_result.x[1],
                "Value": rmse_result.fun,
            },
            "MAD": {
                "C_ss": mad_result.x[0],
                "C_so": mad_result.x[1],
```

```
                "Value": mad_result.fun,
            },
            "MAD_max": {
                "C_ss": mad_max_result.x[0],
                "C_so": mad_max_result.x[1],
                "Value": mad_max_result.fun,
                "System": self.data.loc[np.argmax(np.abs(
                    self.data['E_interaction_CC'] - self.compute_interaction_energy(mad_max_result.x[0],
                    ↪  mad_max_result.x[1]))
                ), 'System']
            },
            "MRPD": {
                "C_ss": mrpd_result.x[0],
                "C_so": mrpd_result.x[1],
                "Value": mrpd_result.fun,
            }
        }

    def compute_matrices(self):
        """Compute RMSE, MAD, MAD_max, and MRPD matrices across C_ss and C_so values."""
        # Compute MRPD values for each combination of C_ss and C_so
        mrpd_values_list = []

        for i, C_ss in enumerate(self.C_ss_values):
            for j, C_so in enumerate(self.C_so_values):
                E_interaction = self.compute_interaction_energy(C_ss, C_so)
                errors = self.data['E_interaction_CC'] - E_interaction

                # Compute error metrics
                self.RMSE_matrix[i, j] = np.sqrt(np.mean(errors ** 2))
                self.MAD_matrix[i, j] = np.mean(np.abs(errors))
                self.MAD_max_matrix[i, j] = np.max(np.abs(errors))

                # Compute MRPD for this specific combination
                mrpd_values = np.abs(
                    (self.data['E_interaction_CC'] - E_interaction) / self.data['E_interaction_CC']) * 100
                mrpd_values_list.append(mrpd_values)

                # Compute mean MRPD
                self.MRPD_matrix[i, j] = np.mean(mrpd_values)
```

```python

                # Track system responsible for max absolute error
                self.MAD_max_system_matrix[i, j] = self.data.loc[np.abs(errors).idxmax(), 'System']

        # Find the minimum MRPD value across all combinations
        mrpd_values_array = np.array(mrpd_values_list)
        self.min_mrpd = np.min(mrpd_values_array)

    def evaluate_methods(self):
        """Evaluate predefined methods and save interaction energies and statistical metrics to an Excel file."""
        results = {'System': self.data['System']}
        error_metrics = {'Metric': ['RMSE', 'MAD', 'MAD_max', 'MRPD']}
        mad_max_dimer = {'Metric': ['-', '-', 'MAD_max_dimer', '-']}  # Extra row for responsible dimer

        for method, (C_ss, C_so) in self.methods.items():
            E_interaction = self.compute_interaction_energy(C_ss, C_so)
            results[f'E_interaction_{method}'] = E_interaction

            # Compute error metrics
            errors = self.data['E_interaction_CC'] - E_interaction
            RMSE = np.sqrt(np.mean(errors ** 2))
            MAD = np.mean(np.abs(errors))
            MAD_max = np.max(np.abs(errors))
            MRPD = np.mean(
                np.abs((self.data['E_interaction_CC'] - E_interaction) / self.data['E_interaction_CC']) * 100)
            system_responsible = self.data.loc[np.abs(errors).idxmax(), 'System']

            error_metrics[method] = [RMSE, MAD, MAD_max, MRPD]
            mad_max_dimer[method] = ['-', '-', system_responsible, '-']  # Only fill MAD_max row

        results['E_interaction_CC'] = self.data['E_interaction_CC']

        # Convert dictionaries to DataFrames
        results_df = pd.DataFrame(results)
        error_metrics_df = pd.DataFrame(error_metrics)
        mad_max_dimer_df = pd.DataFrame(mad_max_dimer)

        # Combine error metrics and MAD_max dimer into one table
```

```
        final_error_metrics_df = pd.concat([error_metrics_df, mad_max_dimer_df.iloc[:, 1:]],
        ↪  ignore_index=True)

        # Compute metrics for optimized coefficients
        optimized_metrics = self.compute_optimized_metrics()
        optimized_metrics_df = pd.DataFrame(optimized_metrics)

        # Save results to Excel
        excel_file = os.path.join(self.output_folder, f'{self.file_name}_methods_analysis.xlsx')
        with pd.ExcelWriter(excel_file) as writer:
            results_df.to_excel(writer, sheet_name='Interaction_Energies', index=False)
            final_error_metrics_df.to_excel(writer, sheet_name='Error_Metrics', index=False)
            optimized_metrics_df.to_excel(writer, sheet_name='Optimized_Metrics', index=False)

    def compute_optimized_metrics(self):
        """Compute all deviation metrics for the optimized coefficients."""
        mins = self.find_minimums()
        optimized_metrics = {}

        for metric, result in mins.items():
            C_ss = result['C_ss']
            C_so = result['C_so']
            metrics = self.compute_all_metrics(C_ss, C_so)
            optimized_metrics[metric] = {
                "C_ss": C_ss,
                "C_so": C_so,
                "RMSE": metrics["RMSE"],
                "MAD": metrics["MAD"],
                "MAD_max": metrics["MAD_max"],
                "MRPD": metrics["MRPD"],
                "System": metrics["System"]
            }

        # Convert to DataFrame
        return pd.DataFrame(optimized_metrics).T

    def save_results(self):
        """Save computed matrices and best coefficient results to an Excel file."""
        excel_file = os.path.join(self.output_folder, f'{self.file_name}_output_matrices.xlsx')
        txt_file = os.path.join(self.output_folder, f'{self.file_name}_min_coefficients_results.txt')
```

```

        with pd.ExcelWriter(excel_file) as writer:
            for name, matrix in zip(['RMSE', 'MAD', 'MAD_max', 'MRPD', 'MAD_max_system'],
                                    [self.RMSE_matrix, self.MAD_matrix, self.MAD_max_matrix,
                                     self.MRPD_matrix, self.MAD_max_system_matrix]):
                pd.DataFrame(matrix, index=self.C_ss_values, columns=self.C_so_values).to_excel(writer,
                ↪  sheet_name=name)

        # Save minimum RMSE, MAD, MAD_max, and MRPD to a text file
        mins = self.find_minimums()
        with open(txt_file, 'w') as file:
            for metric in ['RMSE', 'MAD', 'MAD_max', 'MRPD']:
                file.write(f"Minimum {metric}:\n")
                if metric == 'MAD_max':
                    file.write(
                        f"C_ss: {mins[metric]['C_ss']}, C_so: {mins[metric]['C_so']}, "
                        f"Value: {mins[metric]['Value']:.6f}, "
                        f"System: {mins[metric]['System']}\n\n")
                else:
                    file.write(
                        f"C_ss: {mins[metric]['C_ss']}, C_so: {mins[metric]['C_so']}, Value:
                        ↪  {mins[metric]['Value']:.6f}\n\n")

    def plot_matrices(self):
        """Generate heatmaps for RMSD, MAD, MRPD, and Max(MAD) matrices using a reversed viridis colormap."""
        matrices = {
            'RMSD': self.RMSE_matrix,
            'MAD': self.MAD_matrix,
            'Max(MAD)': self.MAD_max_matrix,
            'MRPD': self.MRPD_matrix
        }

        # Define x-axis ticks at multiples of 0.1 (from 0.1 to 1.0)
        x_tick_values = np.arange(0.10, 2.10, 0.10)  # Successive multiples of 0.1
        x_tick_positions = [np.argmin(np.abs(self.C_so_values - val)) for val in x_tick_values]
        x_tick_labels = [f"{val:.2f}" for val in x_tick_values]

        # Define y-axis ticks at multiples of 0.1 (from 0.1 to 1.0)
        y_tick_values = np.arange(0.1, 2.1, 0.1)  # Successive multiples of 0.1
        y_tick_positions = [np.argmin(np.abs(self.C_ss_values - val)) for val in y_tick_values]
```

```python
    y_tick_labels = [f"{val:.2f}" for val in y_tick_values]

    for name, matrix in matrices.items():
        plt.figure(figsize=(10, 8))

        # Handle MRPD-specific modifications
        if name == 'MRPD':
            # Clip values greater than 100 to 100
            matrix = np.clip(matrix, None, 100)
            # Bound the colormap between the minimum value and 100
            norm = plt.Normalize(vmin=np.min(matrix), vmax=100)
            label = '%'
        else:
            label = 'kcal/mol'
            norm = None

        ax = sns.heatmap(matrix, annot=False, cmap='viridis_r',
                         norm=norm,
                         cbar_kws={'label': label, 'format': '%.2f', 'shrink': 0.8},
                         xticklabels=False,  # Disable default x-tick labels
                         yticklabels=False,  # Disable default y-tick labels
                         square=True)

        # Set x-axis ticks at multiples of 0.1
        plt.xticks(ticks=x_tick_positions, labels=x_tick_labels, rotation=45, fontsize=12)  # Increased
        ↪  font size

        # Set y-axis ticks at multiples of 0.1
        plt.yticks(ticks=y_tick_positions, labels=y_tick_labels, rotation=0, fontsize=12)  # Increased
        ↪  font size

        # Set title and axis labels with increased font size
        plt.title(f"{name} Matrix", fontsize=18, pad=15)  # Increased font size
        plt.xlabel(r'$C_{os}$', fontsize=16)  # Increased font size
        plt.ylabel(r'$C_{ss}$', fontsize=16)  # Increased font size

        # Adjust color bar font size
        cbar = ax.collections[0].colorbar
        cbar.ax.tick_params(labelsize=12)  # Increased font size for color bar ticks
        cbar.set_label(label, fontsize=14)  # Increased font size for color bar label
```

```

            plt.tight_layout()

            # Save the plot
            plt.savefig(os.path.join(self.output_folder, f'{self.file_name}_{name.lower()}_matrix_plot.png'),
                        dpi=600)
            # plt.show()  # Uncomment if visualization is needed


# Example Usage
file_path1 = 'all-rijcos-aug-cc-pVTZ.csv'  # Replace with actual file path
analysis1 = InteractionEnergyAnalysis(file_path1)
# Perform analysis
analysis1.compute_matrices()
analysis1.save_results()
analysis1.plot_matrices()
analysis1.evaluate_methods()

# Example Usage
#file_path2 = 'bauza-dz.csv'  # Replace with actual file path
#analysis2 = InteractionEnergyAnalysis(file_path2)
# Perform analysis
#analysis2.compute_matrices()
#analysis2.save_results()
#analysis2.plot_matrices()
#analysis2.evaluate_methods()

# Example Usage
#file_path3 = 'marco-test2.csv'  # Replace with actual file path
#analysis3 = InteractionEnergyAnalysis(file_path3)
# Perform analysis
#analysis3.compute_matrices()
#analysis3.save_results()
#analysis3.plot_matrices()
#analysis3.evaluate_methods()
```
